# Burden of disease among the world’s poorest billion people: An expert-informed secondary analysis of Global Burden of Disease estimates

**DOI:** 10.1371/journal.pone.0253073

**Published:** 2021-08-16

**Authors:** Matthew M. Coates, Majid Ezzati, Gisela Robles Aguilar, Gene F. Kwan, Daniel Vigo, Ana O. Mocumbi, Anne E. Becker, Julie Makani, Adnan A. Hyder, Yogesh Jain, D. Cristina Stefan, Neil Gupta, Andrew Marx, Gene Bukhman

**Affiliations:** 1 Program in Global Noncommunicable Diseases and Social Change, Harvard Medical School, Boston, Massachusetts, United States of America; 2 Division of Global Health Equity, Brigham and Women’s Hospital, Boston, Massachusetts, United States of America; 3 MRC-PHE Centre for Environment and Health, Imperial College London, London, United Kingdom; 4 Department of Epidemiology and Biostatistics, School of Public Health, Imperial College London, London, United Kingdom; 5 WHO Collaborating Centre on NCD Surveillance and Epidemiology, Imperial College London, London, United Kingdom; 6 Oxford GBD Group, University of Oxford, Oxford, United Kingdom; 7 Section of Cardiovascular Medicine, Boston University School of Medicine, Boston, Massachusetts, United States of America; 8 Partners In Health, Boston, Massachusetts, United States of America; 9 Department of Global Health and Social Medicine, Harvard Medical School, Boston, Massachusetts, United States of America; 10 Department of Psychiatry, University of British Columbia, Vancouver, British Columbia, Canada; 11 Instituto Nacional de Saúde, Ministério da Saúde, Maputo, Mozambique; 12 Faculdade de Medicina, Universidade Eduardo Mondlane, Maputo, Mozambique; 13 Department of Psychiatry, Harvard Medical School, Boston, Massachusetts, United States of America; 14 Sickle Cell Programme, Muhimbili University of Health & Allied Sciences, Dar-es-Salaam, Tanzania; 15 Department of Haematology & Blood Transfusion, Muhimbili University of Health and Allied Sciences, Dar-es-Salaam, Tanzania; 16 George Washington University Milken Institute School of Public Health, Washington, DC, United States of America; 17 Jan Swasthya Sahyog, Bilaspur, Chhattisgarh, India; 18 African Medical Research and Innovation Institute, Cape Town, South Africa; 19 SingHealth Duke-NUS Global Health Institute (SDGHI), Duke-NUS Medical School, Singapore, Singapore; 20 Division of Cardiovascular Medicine, Brigham and Women’s Hospital, Boston, Massachusetts, United States of America; Sciensano, BELGIUM

## Abstract

**Background:**

The health of populations living in extreme poverty has been a long-standing focus of global development efforts, and continues to be a priority during the Sustainable Development Goal era. However, there has not been a systematic attempt to quantify the magnitude and causes of the burden in this specific population for almost two decades. We estimated disease rates by cause for the world’s poorest billion and compared these rates to those in high-income populations.

**Methods:**

We defined the population in extreme poverty using a multidimensional poverty index. We used national-level disease burden estimates from the 2017 Global Burden of Disease Study and adjusted these to account for within-country variation in rates. To adjust for within-country variation, we looked to the relationship between rates of extreme poverty and disease rates across countries. In our main modeling approach, we used these relationships when there was consistency with expert opinion from a survey we conducted of disease experts regarding the associations between household poverty and the incidence and fatality of conditions. Otherwise, no within-country variation was assumed. We compared results across multiple approaches for estimating the burden in the poorest billion, including aggregating national-level burden from the countries with the highest poverty rates. We examined the composition of the estimated disease burden among the poorest billion and made comparisons with estimates for high-income countries.

**Results:**

The composition of disease burden among the poorest billion, as measured by disability-adjusted life years (DALYs), was 65% communicable, maternal, neonatal, and nutritional (CMNN) diseases, 29% non-communicable diseases (NCDs), and 6% injuries. Age-standardized DALY rates from NCDs were 44% higher in the poorest billion (23,583 DALYs per 100,000) compared to high-income regions (16,344 DALYs per 100,000). Age-standardized DALY rates were 2,147% higher for CMNN conditions (32,334 DALYs per 100,000) and 86% higher for injuries (4,182 DALYs per 100,000) in the poorest billion, compared to high-income regions.

**Conclusion:**

The disease burden among the poorest people globally compared to that in high income countries is highly influenced by demographics as well as large disparities in burden from many conditions. The comparisons show that the largest disparities remain in communicable, maternal, neonatal, and nutritional diseases, though NCDs and injuries are an important part of the “unfinished agenda” of poor health among those living in extreme poverty.

## Introduction

Levels and patterns of causes of disease burden across countries are highly associated with differences in country gross domestic product (GDP) per capita [1]. Sanitation, public works projects, vaccination, improved nutrition, improved housing, and better access to higher quality health care are factors linked to economic growth that may shape relationships observed between national economic productivity and life expectancy over time [2, 3]. Economic growth and lower prevalence and intensity of poverty can lead to more available resources to invest on these types of factors, and public investment in intersectoral public health policy and health system intervention implementation can in turn lead to better health, economic growth, and poverty reduction. Evidence suggests that concerted public action to implement policy is necessary to translate economic growth into improvements in health [3]. Within low- and middle-income countries (LMICs), absolute socioeconomic deprivation and socioeconomic gradients among populations are both important determinants of health [4]. Structural mechanisms related to the political and socioeconomic context—such as governance, societal values, and economic, social, and public policies—underlie individuals’ socioeconomic positions [5]. An individual’s socioeconomic status acts as a cause of health inequities by patterning access to the flexible resources of knowledge, money, power, prestige, and social connections which allow action to avoid health risks and minimize the impact of poor health [6]. The combination of cross-country socioeconomic and political factors and within-country socioeconomic stratification shapes the health of people living in extreme poverty. Differences between population health outcomes among populations in high-income countries and populations in LMICs living in extreme poverty are suggestive of the disease burden that could be averted through economic growth and concerted policy and implementation efforts.

The *Lancet* Commission on Reframing Noncommunicable Diseases and Injuries (NCDIs) for the Poorest Billion (*Lancet* NCDI Poverty Commission) hypothesized that levels of extreme poverty are likely to affect the overall levels of NCDI burden and the composition of that burden [7]. The disease burden in low- and lower-middle-income countries (LLMICs) has often been characterized as primarily due to communicable diseases as well as maternal and child illness [8]. Non-communicable diseases (NCDs) in the LLMIC context are often described in the context of transitions involving population aging, urbanization, and higher-income lifestyles [9]. Nonetheless, age-standardized rates of disability-adjusted life years (DALYs) from NCDs and injuries are higher in LLMICs than in high-income countries (HICs), and a growing body of work has characterized factors, including risks associated with the NCD burden that disproportionately affect populations in LLMICs [10–16]. There has been growing evidence about and interest in the socioeconomic patterning of risk factors for NCDs within these countries [17]. Quantifying the disease burden specifically in the poorest populations is important, as using the aggregate global burden to set priorities risks widening disparities in health between rich and poor [18, 19].

There is a body of literature on people living in extreme poverty using the terms “bottom billion” or “poorest billion.” Definitions of the population in question have varied depending on the poverty measure used, and there has been some variation in the precise number of people referred to as the poorest billion [19–23]. For instance, Gwatkin and colleagues examined burden disease among the global poorest billion by aggregating the national-level burden in the poorest countries [18, 19]. There is increasing evidence in LLMICs—from large household survey series, health and demographic surveillance sites, and reviews of literature—on associations between individual or household socioeconomic status and both risk factors exposures and health outcomes related to NCDs [17, 24–27]. Yet, disease burden estimates are often constrained to national level or subnational administrative units by available data. Here, we compare multiple strategies for defining the global poorest billion and for estimating and aggregating their disease burden, including assumptions about how disease burden might be associated with poverty within countries. We describe the estimated disease burden in terms of both the underlying epidemiological and demographic differences from higher-income populations. Results specific to NCDIs from the analysis described here were a component of the evidence underlying the *Lancet* NCDI Poverty Commission Report [20].

## Methods

### Disease burden data

We downloaded publicly available data from the GBD Study 2017. The available data contain population, incidence, prevalence, deaths, years of life lost (YLLs), years lived with disability (YLDs), and disability-adjusted life years (DALYs) by causes of morbidity and mortality for 23 age groups, by sex, and in 195 countries and territories [1, 28, 29]. We used the estimates for the year 2017.

### Poverty data

For poverty estimates, we used a set of eight household-level indicators from the Multidimensional Poverty Index (MPI) created by the Oxford Poverty and Human Development Initiative (OPHI). Typically, the OPHI MPI is estimated by aggregating ten indicators in three dimensions including education, living standards, and health [30]. We excluded the health indicators because our analysis examined health as an outcome. We defined the poorest billion as those living in households deprived in five or more of the eight categories in our poverty index, which include child school attendance, highest educational attainment, electricity, sanitation, safe water, floor material, cooking fuel, and a set of assets (details in S1 Appendix, p 3). The data to classify populations according to these indicators came from representative country surveys (S1 Appendix, pp 4–11), and more detail on this multidimensional definition of poverty can be found elsewhere [31]. The prevalence of people in the poorest billion was estimated by sex and five-year age groups, ending with and including a group for those 80 years and older. This non-monetary approach had advantages in terms of capturing multiple dimensions of poverty, being derived from microdata that allowed for age- and sex-specific estimates, and being available across a broad set of countries [20]. The poverty indicators have clear theoretical links by which to influence disease—use of biomass fuels is associated with household air pollution, lack of sanitation and safe drinking water is linked to diarrhea and malnutrition, dirt floors provide environments for particular pathogens, maternal and childhood education have well established links to mortality, and certain household assets are linked with general resources and wealth, access to information, and mobility to access health care [20].

Prevalence of people in the poorest billion could be estimated in 105 countries from surveys since 2005. Ninety-one of these surveys (87%) were from 2010 or after. For countries with surveys prior to 2010, only Madagascar, Somalia, and Bolivia had more than one million people estimated in the poorest billion, based on 2017 populations. In addition, there were two low-income, seven lower-middle-income countries, and 22 upper-middle-income countries, according to the World Bank list of economies (calendar year 2017), in which surveys to create the poverty index were not available and in which disease burden data existed (S1 Appendix, pp 4–11) [32]. From these countries without surveys, we assumed that the prevalence of people with five or more deprivations was equal to the age- and sex-specific average by country income group among countries with surveys. This constituted less than 5% of the total population in the poorest billion. We defined the populations in high-income countries to be entirely outside the poorest billion.

To define populations consistent with the GBD demographic and disease burden estimates, we applied the proportions of people in the poorest billion from the survey data in each age, sex, and location to the corresponding age-, sex-, and location-specific populations from the GBD study for the year 2017. We used the proportion of under-5-year-olds in the poorest billion as the proportion for the early neonatal (0–6 days), late neonatal (7–27 days), post neonatal (28 days to under 1 year), and 1–4 year old age groups from GBD, as well as the proportion of 80 year-olds and over in the poorest billion for the 80–84, 85–89, 90–94, 95 and older age groups. We found 838 million people with 5 or more deprivations on our poverty index across these countries. Including the low- and middle-income countries without surveys, we added 34 million additional people. In total, our “poorest billion” population contained 873 million people. Though this population was not precisely one billion, we refer to this population of interest as the poorest billion to remain consistent with literature describing this population.

### Analysis

To create disease burden estimates for the poorest billion, we utilized GBD 2017 estimates. We employed five strategies to aggregate the burden for the world’s poorest populations. We report results from one method and the range across methods in the main text, and we show comparisons in the S1 Appendix (pp 37–119).

We called the main approach presented in the results the Selective Ecological approach. The population in the poorest billion was defined as described using the household survey poverty data. Rather than using country-level burden estimates for both the poorest and non-poorest within each country (an approach we also took, S1 Appendix pp 12–13), we sought to inform estimates using ecological relationships between disease burden and poverty across countries. By age, sex, and cause, we conducted mixed-effects linear regressions predicting rates of death and YLDs across countries, using the prevalence of poorest billion population as a covariate and including a random effect for region to better isolate associations with poverty prevalence from other regional differences. We then made predictions from these models for hypothetical groups in which either 100% of the population was in the poorest billion or 0% was in the poorest billion. We scaled these estimates to the national-level GBD estimates in each country, such that the population-weighted average rate for the poorest and non-poorest in each country was consistent with the national-level estimate. We called this the Full Ecological approach (presented as one of the five approaches, S1 Appendix pp 12–15).

Cross-country associations between disease rates and poverty are not necessarily consistent with the within-country associations. To understand how rates of disease may vary by socioeconomic status within LMICs to the best of our knowledge, we conducted a survey of perceived relationships between poverty and disease in LMICs among 97 health practitioners and researchers with a broad range of disease expertise and experience working in or researching health in LMICs. The S1 Appendix (pp 16–17) characterizes the participants in greater detail. Participants answered questions about their perception of relationships between rates of diseases and poverty within LMICs, indicating whether they thought occurrence (defined in survey instructions as incidence) rates and fatality (defined as case fatality) rates were (1) much higher in the non-poorest, (2) higher in the non-poorest, (3) not different, (4) higher in the poorest, or (5) much higher in the poorest. The survey also asked respondents how confident they were in their selection.

To choose conditions to model using this Selective Ecological approach, we found the set of conditions with differences in rates between poorest billion and non-poorest from the expert perception survey and which showed agreement with the direction of the association in the ecological model in over half of age groups (S1 Appendix pp 16–19). To determine conditions that experts thought varied between the poorest billion and non-poorest, we treated the answers on the expert survey as ordinal and used a non-parametric one-sample two-sided sign test to test for a difference in the median from the “No Difference” response. We modeled each of these selected conditions except for those in which the number of countries with non-zero rates in a given age and sex group was too small (fewer than 10 countries) to create stable results. We used the national-level death and YLD rates for both the poorest and non-poorest within countries for the remaining conditions that did not have alignment between the expert survey and the observed ecological relationships. We conducted analysis at the lowest levels of the GBD hierarchies (most specific conditions and ages, and by sex) and aggregated to create internally consistent results.

### Presentation of results

We report results for the poorest billion from the Selective Ecological approach, though we show results for other approaches in the S1 Appendix (pp 37–119) and discuss differences. These other approaches primarily differed in relation to assumptions about within-country burden gradients (assuming none or using ecological relationships to assume a gradient) and the definition of the poorest billion population (population in poorest countries or the household survey population definition). Uncertainty bounds are available on the online GBD results tool; however, random draws from the underlying distributions are used to propagate uncertainty. Without these draws, we were unable to propagate uncertainty when aggregating our results. In many cases, we present the resulting point estimate from the Selective Ecological approach and the range for all 5 approaches. While we conducted our analysis for the most specific age groups within the GBD up to the age group 95 and older, we group the ages above 80 when we present age-specific results because such a small proportion of the population in the poorest billion is above that age. We report rates, rate differences, and rate ratios of YLLs, YLDs, and DALYs to describe the overall levels as well as absolute and relative differences in morbidity and mortality between populations. We used the GBD 2017 age standard for age-standardized results [28].

Analyses were conducted using R version 3.3.1, 3.3.3, and 3.6.1 [33]. The study was submitted to the Harvard Faculty of Medicine IRB office and found exempt from further review (IRB17-0615).

## Results

**Fig 1:**
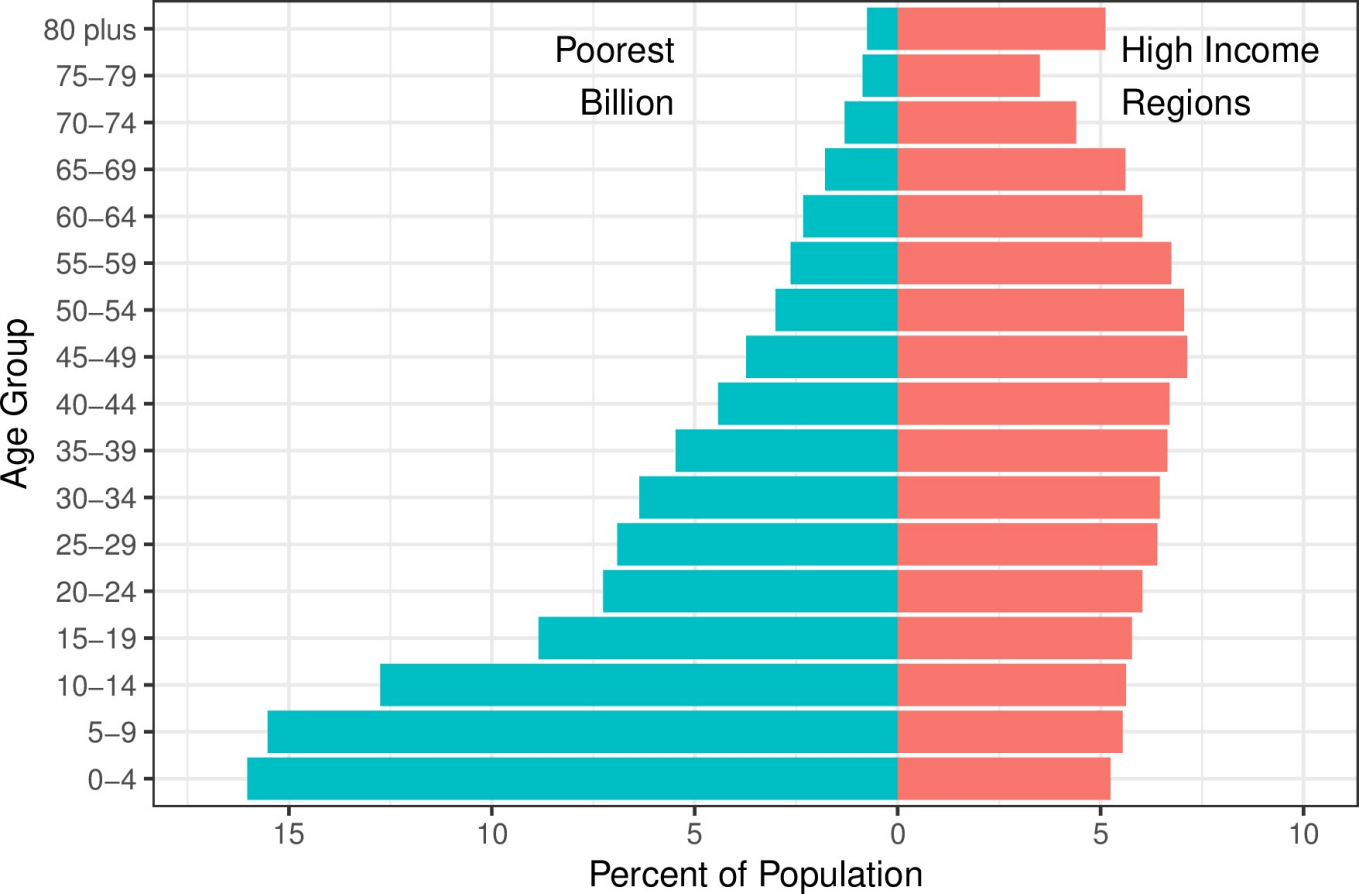
Percent of population in each age group, for the poorest billion versus high-income regions. High-income regions defined as Western Europe, high-income North America, high-income Asia-Pacific, Australia, and New Zealand from the GBD 2017. Percentages shown within each group: percentages in the poorest billion total to 100% and in high-income regions total to 100%.

The population in the poorest billion was considerably younger than in high-income regions (HIRs) (Fig 1). The age structure within countries was younger in poorest billion populations, and the age structure in the poorest billion globally also varied by the income level of the countries in which they live, with the populations living in middle-income counties older than those in low-income countries (S1 Appendix, pp 20–24). We found that the majority of the poorest billion population was living in Sub-Saharan Africa (56·7%), while 31·6% resided in South Asia, leaving only 11·7% in other regions, including Southeast Asia, East Asia, Latin America, and the Middle East and North Africa.

**Fig 2:**
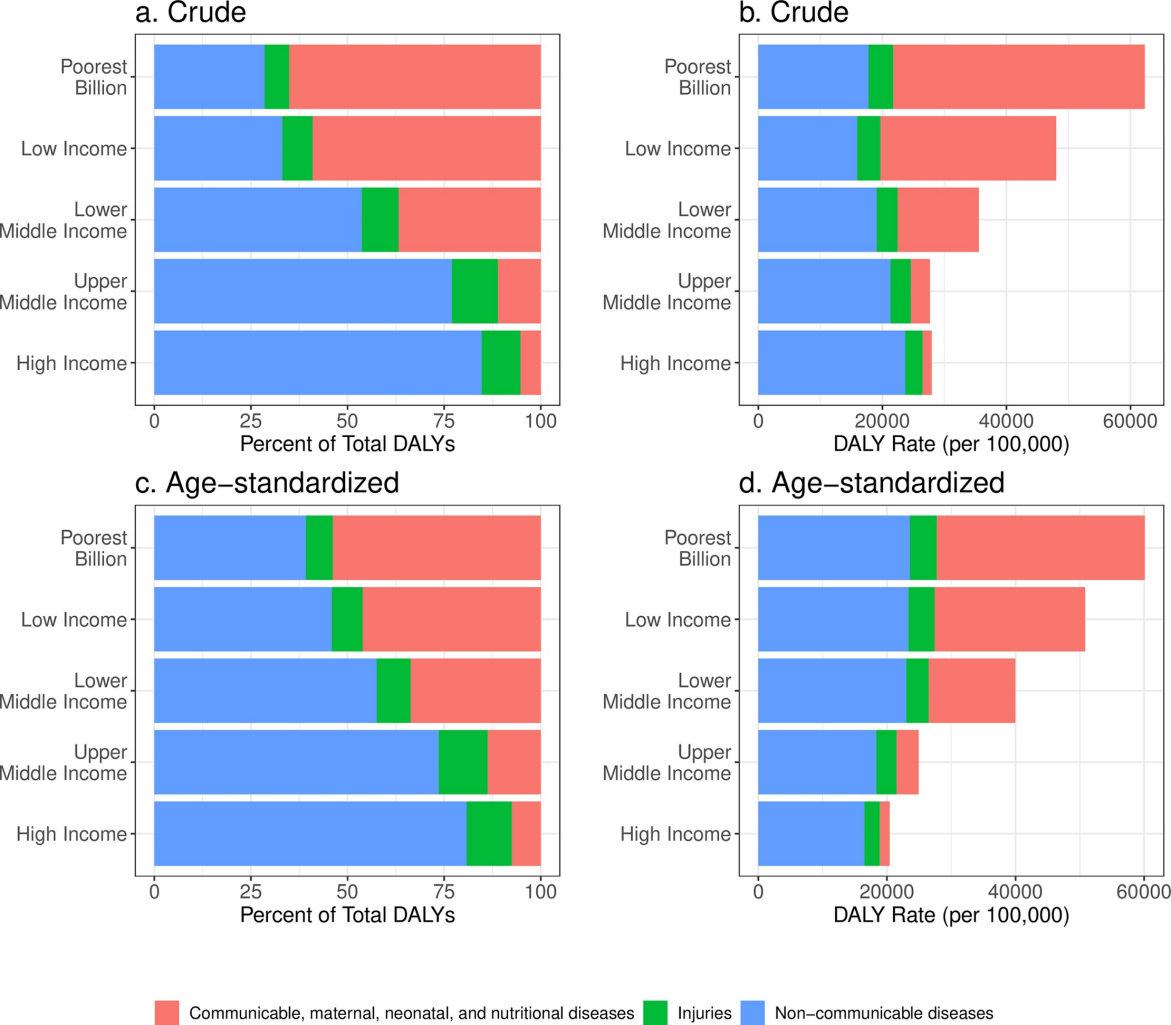
Proportion of DALYs by broad cause groups, crude (a) and age-standardized (b); Rates of DALYs by broad cause groups, crude (c) and age-standardized (d), for the poorest billion and World Bank income groups. Age-standardized results were standardized using the GBD 2017 population standard [28].

Communicable, maternal, neonatal, and nutritional (CMNN) diseases accounted for 65·1% (approaches ranged 55·0%-65·1%) of the DALYs in the poorest billion, compared to 28·5% (28·5%-37·4%) from NCDs and 6·4% from injuries (6·4%-7·8%). Fig 2 shows proportions and rates of broad causes of age-standardized and crude DALYs for the poorest billion as well as World Bank income groups. The difference in cause distribution between the poorest billion and high-income countries was stark, with 5·2% of DALYs from CMNN causes, 84·7% from NCDs, and 10·0% from injuries in high-income countries.

**Fig 3:**
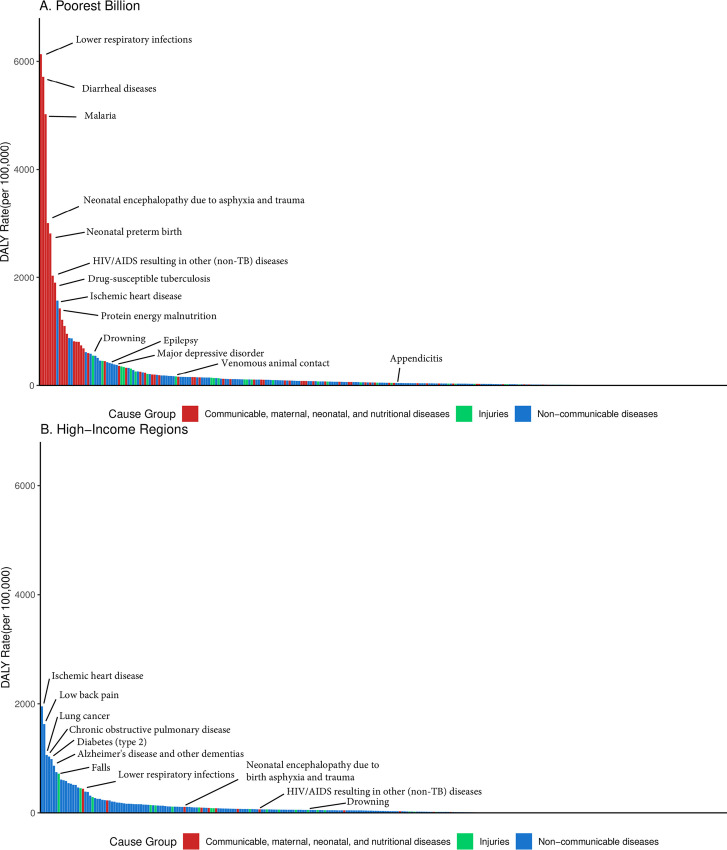
Crude DALY rate by condition in the poorest billion (a) and in high-income regions (b). High-income regions defined as Western Europe, high-income North America, high-income Asia-Pacific, Australia, and New Zealand from the GBD 2017.

Several major CMNN conditions—neonatal disorders, lower respiratory infections, diarrheal diseases, HIV/AIDs, malaria, and tuberculosis—make up 71·5% (71·4–72·9%) of the CMNN DALYs in the poorest billion and 46·5% (40·1–46·5%) of the overall DALYs (Fig 3). In HIRs, only 3·8% of DALYs overall were from these causes. Out of the 293 most specific conditions, 274 (272–274) each contributed less than 1% percent of the disease burden in the poorest billion, but collectively made up 38·2% (38·0%-42·3%) of DALYs. Similarly, 271 conditions contributing less than 1% of the disease burden in HIRs collectively made up 44·4% of DALYs.

Standardizing the age structure lessened the disparity in the rate of DALYs from CMNN causes between poor and wealthier populations, showing that part of the difference in observed burden is the younger age structure of the poorest billion (Fig 2C and 2D). However, the age-standardized DALY rate from CMNN conditions in the poorest billion was still 21 times higher (approaches range 13–22 times higher) than that in high-income countries. Standardizing by age also showed higher rates of NCD burden among the poor (23,583 DALYs per 100,000 [range 22,232–25,231]) compared to those in high-income countries (16,510 DALYs per 100,000), the opposite direction of the crude difference.

**Fig 4:**
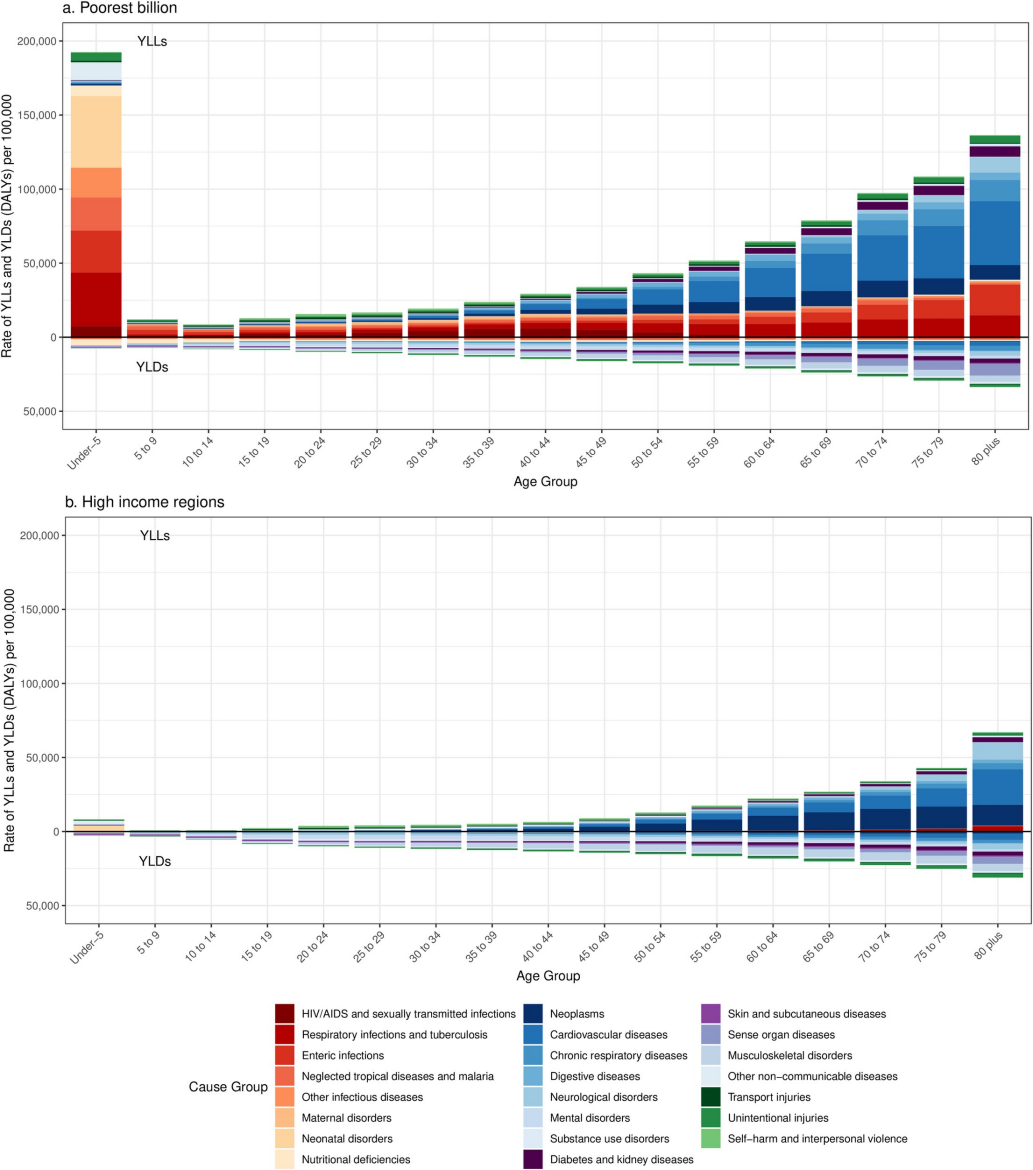
YLL and YLD (DALY) rates by age in the poorest billion (a) and high-income regions (b). Approaches described in detail in Methods. Results for other approaches in S1 Appendix (pp 38–44). High-income regions defined as Western Europe, high-income North America, high-income Asia-Pacific, Australia, and New Zealand from the GBD 2017.

YLL rates were highest under five years of age, declined through childhood, and then increased through adulthood (Fig 4). In the poorest billion, high rates of malaria, diarrheal diseases, lower respiratory infections, and neonatal conditions contributed to an overall under-five YLL rate of 192,506 (approaches range 123,141 to 204,672) per 100,000 people, compared to 8,215 per 100,000 in HIRs. Rates of YLLs from NCDs overtook rates of YLLs from CMNN conditions at older ages in the poor, though CMNN YLL rates remained high at older ages in the poor compared to HIRs, largely from diarrheal and lower respiratory diseases. Rates of YLLs were higher in the poorest billion compared to those in HIRs in every age group. Age-specific YLL rates were higher in almost every broad category of illness in the poorest billion, though cancer notably had higher age-specific YLL rates in HIRs after age 60. Mental and substance use disorders had higher YLL rates in HIRs in some ages, though deaths from mental disorders in the GBD only included eating disorders.

The absolute disparity in YLD rates was much smaller. In both the poorest billion and in HIRs, rates of YLDs increased with age, largely due to NCDs and injuries. Mental disorders had higher YLD rates in HIRs than in the poorest billion from ages 5 to 54 but higher YLD rates in the poorest billion after age 50. There were higher YLD rates from substance use disorders in HIRs in almost every age group. YLDs comprised a smaller proportion of the DALY rates than YLLs across every age group in the poorest billion, but in HIRs, YLDs made up more than half of the DALY burden in older children through middle age. In contrast to the YLL burden, much of the YLD burden was from NCDs and injuries, even in the poorest billion (66·9% [66·8%-74·0%]).

**Fig 5:**
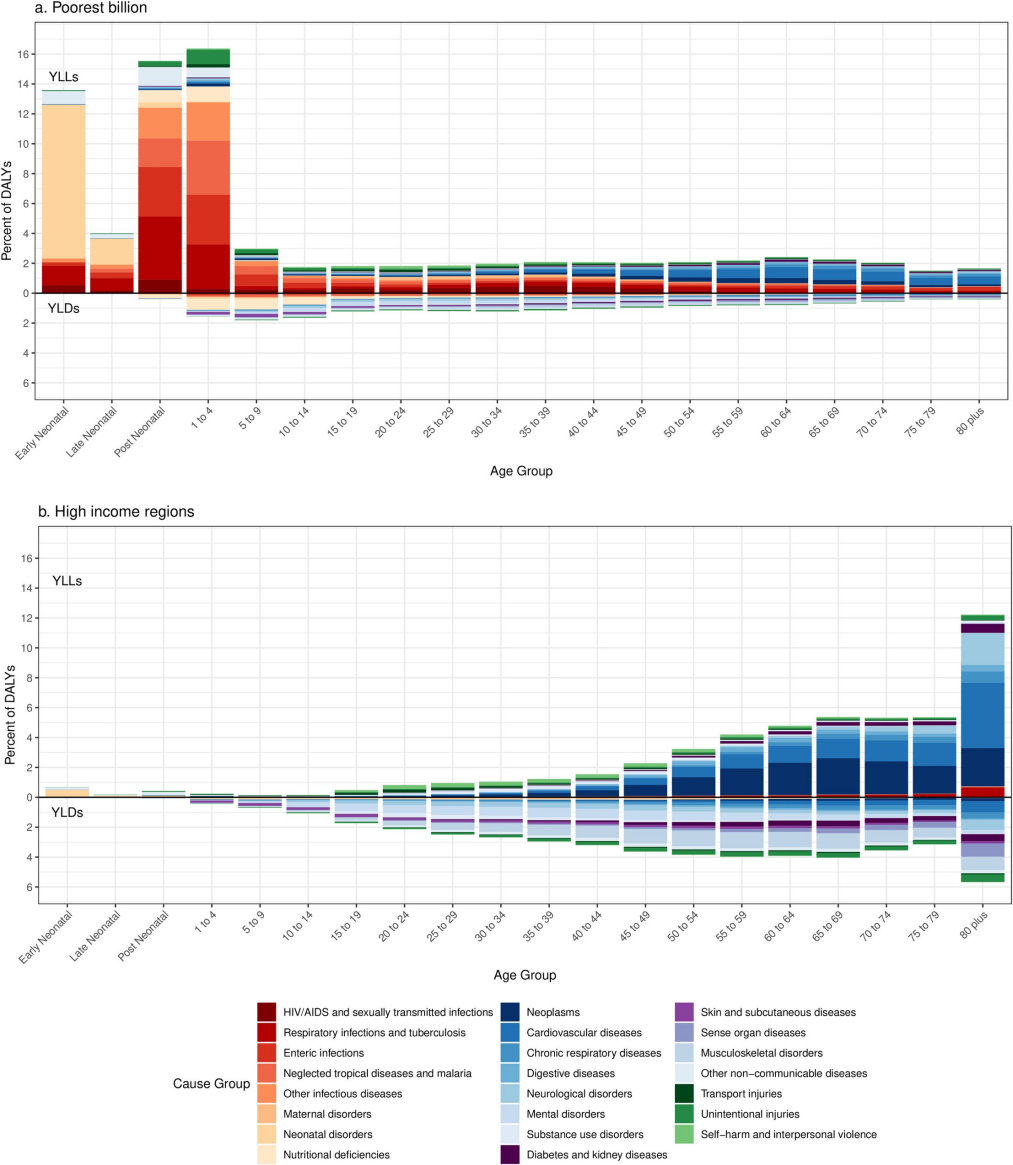
Age-specific YLLs and YLDs as a proportion of all-age DALYS in the poorest billion (a) high-income regions (b). Approaches described in detail in Methods. Results for other approaches in S1 Appendix (pp 45–51). High-income regions defined as Western Europe, high-income North America, high-income Asia-Pacific, Australia, and New Zealand from the GBD 2017.

The high contribution of mortality to disease burden in the poor was magnified in total counts of YLLs because the population in the poorest billion was much younger than those in high-income or even middle-income countries. Fig 5 shows the proportion of total DALYs in terms of age-specific YLLs and YLDs for the poorest billion and HIRs. In the poorest billion, 60·3% (55·6%-60·8%) of YLLs and 51·5% (45·4%-50·8%) of DALYs occurred under the age of 5, compared to 3·0% YLLs and 2·0% of DALYs in HIRs. The five methods estimated that between 70·7% and 77·9% of the DALYs in the poorest billion occur before age 40, compared to 20·6% in HIRs, both because of the younger population and the disproportionately higher DALY rates under age five. Many of these DALYs in children in the poorest billion were from CMNN conditions such as pneumonia, diarrheal disease, and neonatal conditions, but congenital anomalies and injuries additionally contributed substantial burden.

**Fig 6:**
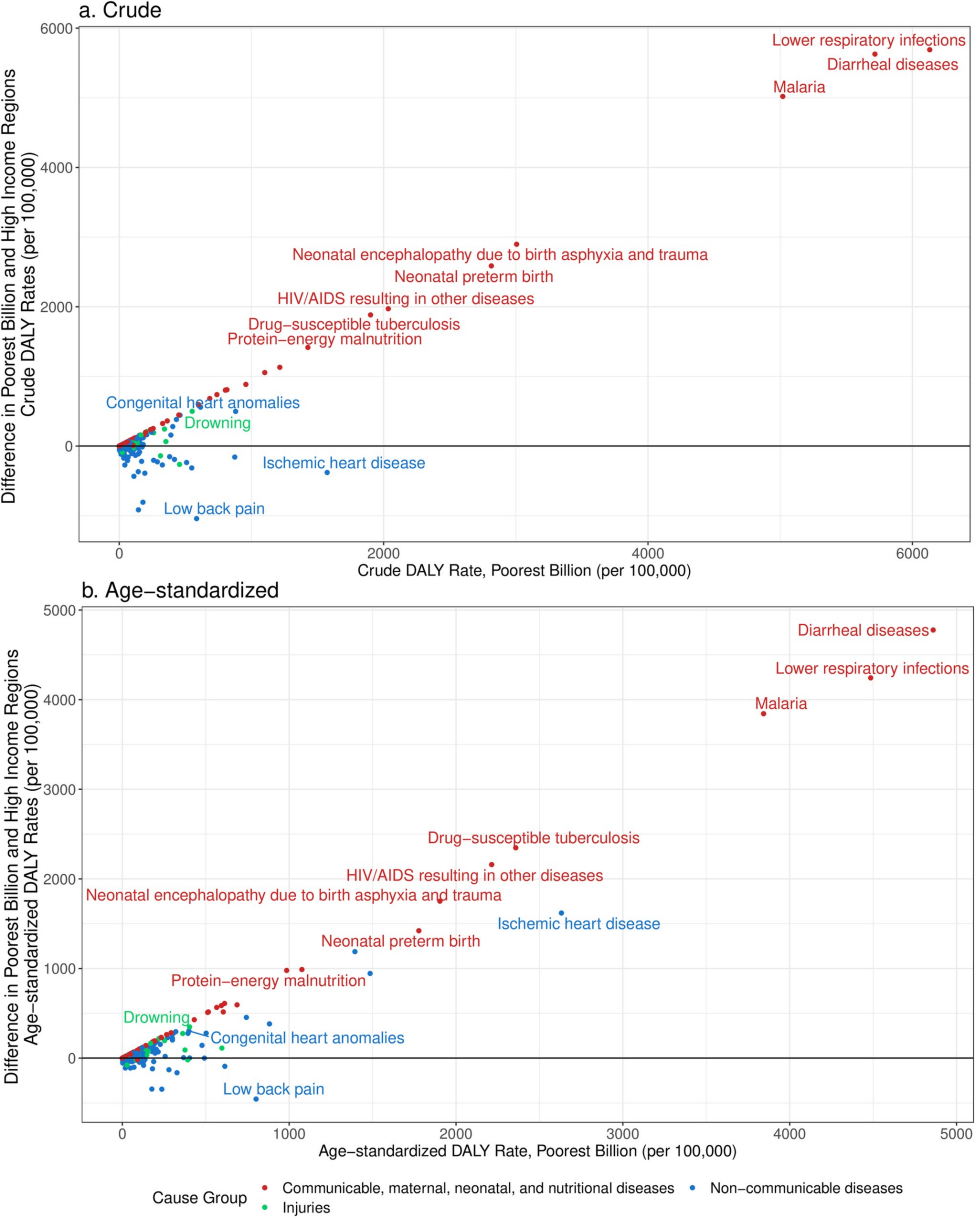
Crude DALY rate in the poorest billion and difference between crude DALY rate in poorest billion and high-income regions (a) and difference between age-standardized DALY rate in poorest billion and high-income regions (b). Approaches described in detail in Methods, results for other approaches and more focused graphs on NCDs and injuries in S1 Appendix (pp 69–78). Results for other approaches in S1 Appendix (pp 52–59). High-income regions defined as Western Europe, high-income North America, high-income Asia-Pacific, Australia, and New Zealand from the GBD 2017. Age-standardized results were standardized using the GBD 2017 population standard [28].

Fig 6 shows the gaps in crude and age-standardized DALY rates between the poorest billion and HIRs, by specific condition. The largest differences in both crude and age-standardized DALY rates were lower respiratory diseases, diarrheal diseases, and malaria, followed by other notable CMNN conditions. Almost all CMNN conditions had higher crude and age-standardized rates among the poorest billion compared to HIRs. By comparison, there were 55 (50–56) NCDs that had lower crude DALY rates among the poorest billion compared to HIRs but higher age-standardized rates compared with HIRs. The largest swings in these rate differences were in ischemic heart disease and chronic obstructive pulmonary disease. Diseases like intracerebral hemorrhage, congenital heart anomalies and other congenital birth defects, epilepsy, asthma, and rheumatic heart disease had large crude and age-standardized disparities with higher DALY rates among the poor. Among non-communicable diseases, the largest gaps in crude DALY rates were in congenital heart anomalies, intracerebral hemorrhage, neural tube defects, and epilepsy. Meanwhile, the largest gaps in age-standardized DALY rates were observed in ischemic heart disease, intracerebral hemorrhage, chronic obstructive pulmonary disease, ischemic stroke, and type 2 diabetes. There were diverse patterns across cancer types. Some age-standardized DALY rates were substantially higher in the poorest billion, like cervical cancer, esophageal cancer, stomach cancer, and liver cancer from hepatitis B, while others were higher in HIRs, such as lung cancer, colorectal cancer, and pancreatic cancer. For injuries, the conditions with the largest gaps in age-standardized DALY rates were from drowning, pedestrian road injuries, fire, heat, and hot substances, and venomous animal contact. The injury categories with the largest differences in crude rates were similar. The main injury categories with higher age-standardized DALY rates in high-income countries involved self-harm or firearm violence. The crude DALY rate from falls was higher in HIRs, whereas the age-standardized rate was higher in the poorest billion.

**Fig 7:**
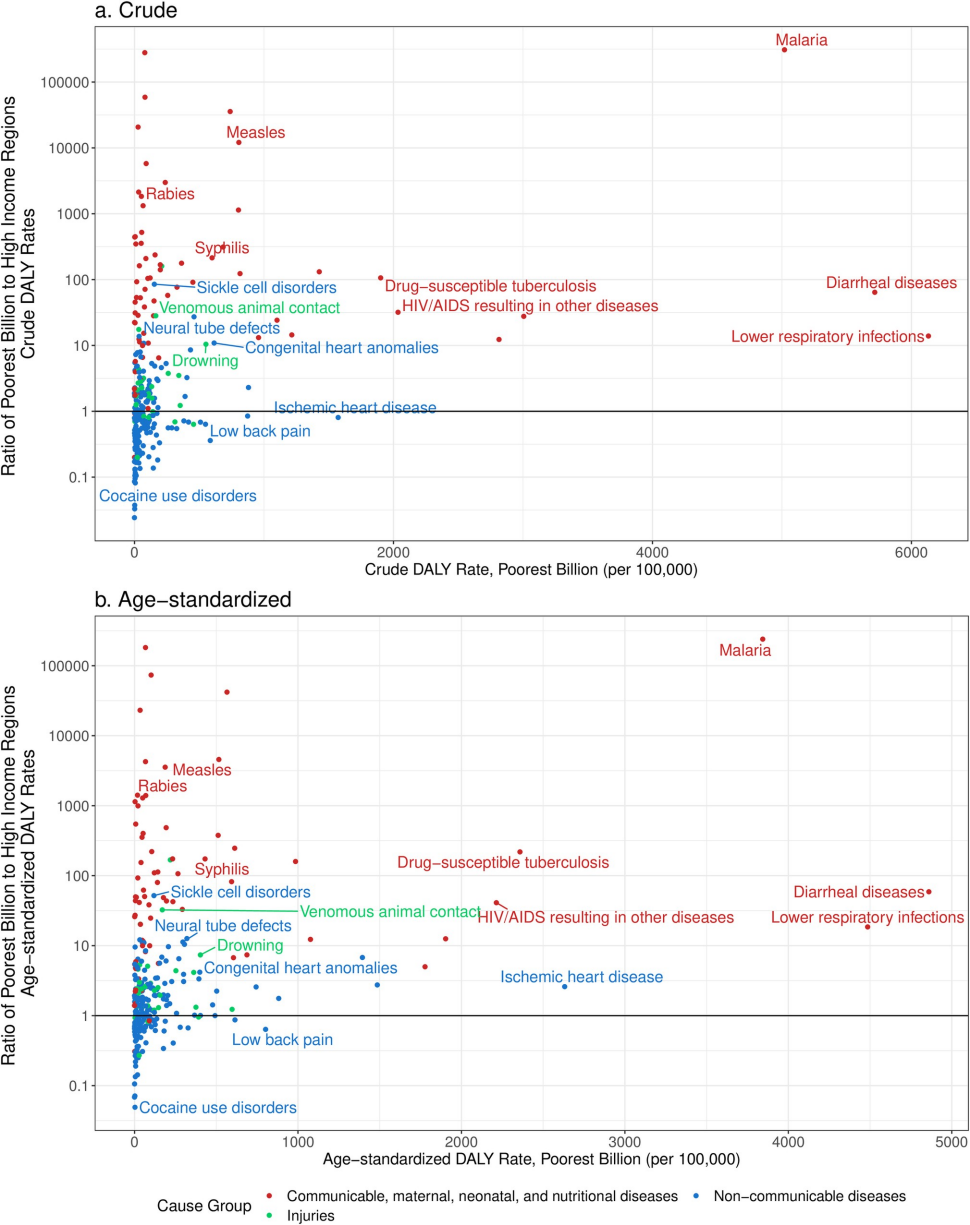
Crude DALY rate in the poorest billion and ratio of crude DALY rate in poorest billion and high-income regions (a) and ratio of age-standardized DALY rate in poorest billion and high-income regions (b). Approaches described in detail in Methods. High-income regions defined as Western Europe, high-income North America, high-income Asia-Pacific, Australia, and New Zealand from the GBD 2017. There are infinite ratios (zero DALYs in high income regions) for the following causes- African trypanosomiasis, Guinea worm, Onchocerciasis, Schistosomiasis, Vitamin A deficiency, Yellow fever). Ratios on y-axis expressed on a logarithmic scale. Age-standardized results were standardized using the GBD 2017 population standard [28].

While the largest absolute differences in DALY rates came mostly in the causes of the largest burden, the largest relative disparities in DALY rates often came in other conditions (Fig 7). Communicable diseases such as HIV/AIDS, lower respiratory infections, and diarrheal diseases remained highly ranked in terms of rate ratios between age-standardized DALY rates between the poorest billion and HIRs, but neglected tropical diseases almost exclusively found in lower-income countries, malaria, and certain vaccine-preventable illnesses like yellow fever, measles, tetanus, and pertussis, had higher rate ratios. All these conditions had age-standardized DALY rate ratios over 100. Sickle cell disorders (age-standardized DALY rate ratio ranging 51·9–85·2 across approaches), rheumatic heart disease (5·6–12·7), neural tube defects (7·1–11·3), appendicitis (11·0–16·7), and cirrhosis due to hepatitis B (8·3–11·3) had the highest age-standardized DALY rate ratios of NCDs. Some of the largest NCDs in terms of DALYs ranked lower in terms of rate ratios when comparing to HIRs, such as ischemic stroke (rate ratio of 2·6 [2·5–3·1]), ischemic heart disease (2·6 [2·1–2·8]), and type 2 diabetes (1·8 [1·4–1·9]). Tracheal, bronchus, and lung cancer had higher age-standardized DALY rates in HIRs (0·4 [0·3–1·0]). Causes of injury with the highest age-standardized rate ratios included conflict and terrorism, venomous animal contact, and exposure to forces of nature (ratios all over 10).

## Discussion

Our analysis found substantial gaps in rates of death and disability between the populations in high-income countries and the poorest populations. Demographic and epidemiological factors linked to poverty both played a substantial role in these gaps.

The largest gaps between the poorest billion and wealthier populations were in rates of CMNN conditions, largely because of gross inequalities in rates of morbidity and mortality, though heightened by the younger population of the poorest billion. Substantial progress has been made in reducing health burdens associated with certain clinical conditions, like diarrheal diseases, lower respiratory infections, maternal mortality, and malaria, with age-standardized DALY rates from each declining over 50% in low-income countries from 1990 to 2017 and age-standardized DALY rates for HIV returning to pre-1990 levels after peaking in the early 2000s [10]. However, it is clear almost 20 years after the study by Gwatkin and colleagues, that there is still a large unfinished agenda with respect to CMNN conditions. This agenda receives continued support from efforts like the *Lancet* Commission on Investing in Health and the Countdown to 2015, which has renewed efforts towards improving women’s, children’s, and adolescents’ health by forming Countdown to 2030 [34–36].

At the same time as an enduring focus is needed to continue to drive down global disparities in disease burden from CMNN conditions, there are growing concerns about the rise of NCDs in LLMICs with aging populations and growing exposures to cardiometabolic risk factors [37]. In LLMICs, obesity, blood lipids, diabetes, and blood pressure are rising, but dietary risk factors are more complex, and some of the rising exposure, such as blood pressure, tend to plateau and even decrease at a certain level of country economic development [14]. Our finding that NCDs and injuries make up over 35% of DALYs in the poorest billion shows that they are already an important part of the disease burden. This burden comes from a diverse set of conditions, including cardiovascular disease, chronic respiratory disease, cancer, and diabetes, but also importantly more than half also comes from other conditions such as neurological disorders, mental health conditions, congenital anomalies, hemoglobinopathies, sensory diseases, kidney and liver diseases, and skin and oral conditions. From the perspective of health system planning, as the burden from CMNN conditions declines and the population ages, there is evidence that the NCD burden will be a growing challenge for many LMICs to address, and many of these countries are not well prepared, given current spending and health system capacity [38]. From an equity perspective, some have noted that age-standardized rates of DALYs from NCDs are higher in many low-income countries than in higher income populations [11]. While the age-standardized DALY rates from NCDs are declining for all World Bank income groups, the gap between low-income countries and high-income countries has actually grown from 1990 to 2016 [39].

We found that both crude and age-standardized DALY rates from injuries were higher among the poor. Conditions like drowning, pedestrian road injuries, and venomous animal contact were all higher among the poor and have well-documented interventions that could save many lives [40, 41]. For injuries that require emergency and surgical care, poor access to care compounds the burden in many countries with substantial populations in the poorest billion [42, 43].

### Limitations

Ideally, disease burden estimates could be generated using inputs already stratified by SES within countries to create consistent results rather than relying on either grouping populations from whole countries or examining ecological relationships. Although we were able to examine poverty at a household level, the use of country-level GBD estimates and our ecological analysis proved insightful but inherently limited results. Qualitatively, the different approaches to disease burden estimation led to a similar picture of disease burden in the poorest billion. There were variations in geography, population structure, and epidemiology of particular conditions across the methods that are further shown in the S1 Appendix (pp 37–119). Increasingly available subnational estimates of disease burden have the potential to continue to improve the characterizations of disease in high-poverty areas within countries [44]. Poverty is associated with poor health across country-income status; life expectancy differs by about 20 years between the counties in the United States with the highest and lowest life expectancies, with socioeconomic factors explaining a large proportion of county-to-county variation [45]. Our analysis focused on absolute poverty in LMICs rather than relative poverty gradients.

Although estimates from the GBD Study are the most comprehensive available, they are limited by data availability and methodologic assumptions. The estimates for causes of death in lower-income countries rely on limited verbal autopsy data and the effects of covariates and geographic trends through modeling, given the absence of vital registration in these countries. These data limitations could have effects on death estimates in the poorest billion from causes like ischemic heart disease, for example [25, 46]. Although an analysis comparing country-level GBD cause-specific death rates to corresponding death rates in health and demographic surveillance sites found reasonable concordance, the estimates of death rates in adults from ischemic heart disease in the GBD were systematically higher than those from acute cardiac events in the sites [47]. Analysis of data from the Million Death Study in India also found substantially lower rates of death from IHD compared to GBD and higher rates of death from stroke [48]. Differences in large causes of death such as IHD can have large impacts on overall results. In the GBD results, the lack of data is reflected partially through the wider uncertainty intervals around estimates in countries without data. We were unable to propagate this uncertainty in our analysis given the publicly available estimates. The distribution of severity of illness from the GBD is also often reliant on data from high-income countries [49]. For example, the average disability weight, calculated by dividing YLD rates by prevalence and standardizing by age, for a person with major depression in low-income countries is 0.20, while that in high-income countries is also 0.20, despite much higher availability of treatment in high-income countries that one might expect to shift the distribution of severity. This type of limitation is relevant to a range of conditions within the GBD for which there are disparities in treatment access and quality.

There are also limitations to each approach we took to quantify the burden. The era of the Sustainable Development Goals has brought an important focus on inequality within countries in addition to across them [50, 51]. Our findings that 54% of the people in the poorest billion by our poverty index live in middle-income countries suggests that using data based solely on national estimates will be inadequate to prioritize the worse off. We were able to utilize evidence from our expert survey; however, given the lack of specific and representative data in LMICs for the burden from many health conditions, the experts had to rely on their own experiences and expertise in addition to data in answering the survey. A larger number of experts surveyed and alternative methods to estimate the degree of the associations between poverty and health outcomes, such as those used in expert elicitation for climate research, could have allowed for more complex estimates from the expert survey, such as probability distributions [52, 53]. The survey results suggested that assuming no association between burden and poverty within countries would be inaccurate. Yet, associations between disease burden and poverty across countries do not necessarily approximate household-level associations. For example, despite evidence that conditions like mental disorders in LMICs are more common among the poor, the ecological relationships did not show a positive association between common mental health disorders and country-level poverty [54]. Though the use of ecological relationships rather than within-country gradients is inherently limited, using ecological relationships, constrained by the plausibility from expert perceptions and bounded by national-level GBD estimates, provided a useful perspective, which we were able to compare with other perspectives (see S1 Appendix, pp 37–119).

## Additional evidence and conclusions

Multi-country survey series can sometimes be analyzed according to household socioeconomic factors, contributing evidence on risk factors and disease burden by SES. These types of analyses have typically found higher mortality rates among poorer groups [55]. Among studies using verbal autopsy at health and demographic surveillance sites in low and lower-middle-income countries, higher death rates have been documented in the poor from malaria, communicable disease generally, HIV, TB, and childhood illness such as diarrhea and acute respiratory infection, though there has been some variation [56–61]. Studies have found conflicting evidence about death rates from NCDs in different settings [59, 62]. Studies examining proportions of deaths, rather than rates, have often shown higher proportions of deaths from various communicable conditions in poorer groups, conflicting evidence about drowning in children, lower proportions of deaths from road traffic injuries, and lower proportions from circulatory diseases [57, 59, 63–66]. A recent multi-site study found higher rates of death from NCDs overall among poorer groups in several sites; however, population sizes and verbal autopsy classification methods limit how narrowly groups of diseases can be examined [67]. There is an opportunity to incorporate measures of SES into more data collection and to systematically include these types of data into modeled estimates of disease burden, ensuring that we adequately monitor inequalities so as to more effectively redress them [68].

The largest disparities in disease burden persist among CMNN conditions. In addition, our finding that age-specific DALY rates in the poorest billion are higher than those in high-income regions for NCDs and injuries shows that NCDs and injuries should be considered part of the “unfinished agenda” of preventing and treating illness in populations living in extreme poverty.

## Supporting information

S1 Appendix Methods and results appendix. Appendix containing additional methodological information and results.
(PDF)

S1 Table Results table. Table containing results.
(XLSX)
