# Supplementary material for: Burden of disease among the world’s poorest billion people: An expert-informed secondary analysis of Global Burden of Disease estimates
Source: PLoS One. 2021 Aug 16;16(8):e0253073. doi: 10.1371/journal.pone.0253073 (PMC8366975; doi:10.1371/journal.pone.0253073)
Supplement: S1 Appendix — Appendix containing additional methodological information and results. (PDF) [file pone.0253073.s001.pdf]

Supplementary Appendix

Appendix Table 1: Poverty indicators used to define the poorest billion

Appendix Table 2: Country poverty estimates and inclusion in burden aggregation methods

Analytic Approaches

Appendix Table 3: Characteristics of analytical burden groups

Survey Methods and Acknowledgment

Appendix Table 4: Characteristics of expert survey respondents

Appendix Figure 1: Age and sex distributions of various populations

Appendix Table 5: Percent of the population below specified ages in various groups

Survey of Expert Perceptions Results

Appendix Figure 2: Summary of expert perceptions of disease incidence and case fatality among the poorest billion compared to non-poorest in low- and middle-income countries, by disease groups

Appendix Table 6: Summary results from expert survey on perceptions of disease occurrence and fatality

Appendix Figure 3: Proportion of DALYs by broad cause groups, crude (a) and age-standardized (b); Rates of DALYs by broad cause groups, crude (c) and age-standardized (d), for five approaches to estimating the burden in the poorest billion and World Bank income groups

Appendix Figure 4: Cause-specific DALY Rates by Age

Appendix Figure 5: Percent of Cause-Specific DALYs by Age

Appendix Figure 6: Difference between DALY Rates in Poorest Billion and High-income Regions

Appendix Figure 7: Age-specific Percent of DALYs by Cause

Appendix Figure 8: Difference between DALY Rates in Poorest Billion and High-income Regions (NCDs and Injuries)

Appendix Figure 9: Difference between YLL Rates in Poorest Billion and High-income Regions

Appendix Figure 10: Difference between YLD Rates in Poorest Billion and High-income Regions

Appendix Figure 11: Difference between YLL Rates in Poorest Billion and High-income Regions (NCDs and Injuries)

Appendix Figure 12: Difference between YLD Rates in Poorest Billion and High-income Regions (NCDs and Injuries)

Comparison of Estimation Methods

Example Analysis

Appendix Table 7: Regression example results

Appendix Figure 13: Analysis example regression results

Appendix Table 1: Poverty indicators used to define the poorest billion

**Education**	Schooling	No household member has completed five years of schooling
	Attendance	Any school-aged child in the household is not attending school up to class 8
**Standard of living**	Electricity	The household has no electricity
	Sanitation	The household's sanitation facility is categorized as unimproved or it is shared with other households
	Water	The household does not have access to safe drinking water or safe water is more than a 30-minute walk, round trip
	Floor	The household has a dirt, sand, or dung floor
	Cooking fuel	The household cooks with dung, wood, or charcoal
	Assets	The household does not own more than one of the following: radio, TV, telephone, bike, motorbike, or refrigerator; however, if the household owns a car or truck, it does not count as deprived in this category
**Health***	Child mortality	Any child has died in the family in the five-year period preceding the survey
	Malnutrition	Any adult under 70 years of age is undernourished in terms of BMI, or any child for whom there is nutritional information is undernourished in terms of weight-for-age.

*Health indicators included in MPI from OPHI but excluded from our assessment. We defined the poorest billion as those living in households counted as deprived in five or more of the eight indicators. Surveys defined people in the household as "usual residents" in almost all cases (see appendix for exceptions).

Appendix Table 2: Country poverty estimates and inclusion in burden aggregation methods

Location	World Bank Income Group (2017)	GBD Burden Estimate Availability	Population in 2017 (GBD 2017)	Survey	Year	Population in Poorest Billion	Proportion of Population in Poorest Billion	Used in Country Aggregation Approach	Used in Poorest Billion Aggregation and Ecological Approaches
**Afghanistan**	LIC	Y	32854767	DHS	2016	8250698	25.10%	F	T
**Albania**	U-MIC	Y	2766162	DHS	2009	44	<0.1%	F	T
**Algeria**	U-MIC	Y	40463698	MICS	2013	107067	0.30%	F	T
**American Samoa**	U-MIC	Y	55631	None		1130*	2.0%*	F	T
**Andorra**	HIC	Y	79966	None		^	^	F	F
**Angola**	L-MIC	Y	28202299	None		4629653*	16.4%*	F	T
**Antigua and Barbuda**	HIC	Y	88957	None		^	^	F	F
**Argentina**	HIC	Y	44265909	None		^	^	F	F
**Armenia**	U-MIC	Y	3027231	DHS	2010	0	<0.1%	F	T
**Aruba**	HIC	N		None		^	^	F	F
**Australia**	HIC	Y	23943082	None		^	^	F	F
**Austria**	HIC	Y	8793125	None		^	^	F	F
**Azerbaijan**	U-MIC	Y	10225021	DHS	2006	15050	0.20%	F	T
**Bahrain**	HIC	Y	1470373	None		^	^	F	F
**Bangladesh**	L-MIC	Y	156981002	DHS	2014	39593478	25.20%	F	T
**Barbados**	HIC	Y	295884	MICS	2012	0	<0.1%	F	T
**Belarus**	U-MIC	Y	9491040	MICS	2005	0	<0.1%	F	T
**Belgium**	HIC	Y	11319411	None		^	^	F	F
**Belize**	U-MIC	Y	394958	MICS	2011	4004	1.00%	F	T
**Benin**	LIC	Y	11585301	DHS	2012	5147069	44.40%	T	T
**Bermuda**	HIC	Y	65886	None		^	^	F	F
**Bhutan**	L-MIC	Y	957410	MICS	2010	105601	11.00%	F	T
**Bolivia**	L-MIC	Y	11542995	DHS	2008	1672854	14.50%	F	T
**Bosnia and Herzegovina**	U-MIC	Y	3399239	MICS	2012	525	<0.1%	F	T
**Botswana**	U-MIC	Y	2281843	None		44967*	2.0%*	F	T
**Brazil**	U-MIC	Y	211812707	PNAD	2014	512627	0.20%	F	T

**British Virgin Islands**	HIC	N		None		^	^	F	F
**Brunei**	HIC	Y	432451	None		^	^	F	F
**Bulgaria**	U-MIC	Y	7052339	None		158759*	2.2%*	F	T
**Burkina Faso**	LIC	Y	21121966	DHS	2010	13655378	64.70%	T	T
**Burundi**	LIC	Y	10905413	DHS	2010	7937451	72.80%	T	T
**Cambodia**	L-MIC	Y	16122383	DHS	2014	2187088	13.60%	F	T
**Cameroon**	L-MIC	Y	27769738	DHS	2011	10101590	36.40%	T	T
**Canada**	HIC	Y	35982918	None		^	^	F	F
**Cape Verde**	L-MIC	Y	545806	None		86283*	15.8%*	F	T
**Cayman Islands**	HIC	N		None		^	^	F	F
**Central African Republic**	LIC	Y	4622515	MICS	2010	3459994	74.80%	T	T
**Chad**	LIC	Y	15222086	DHS	2015	12578760	82.60%	T	T
**Channel Islands**	HIC	N		None		^	^	F	F
**Chile**	HIC	Y	17918235	None		^	^	F	F
**China**	U-MIC	Y	1412480390	CFPS	2014	1107398	0.10%	F	T
**Colombia**	U-MIC	Y	50606157	DHS	2010	968253	1.90%	F	T
**Comoros**	LIC	Y	718322	DHS-MICS	2012	164201	22.90%	F	T
**Congo**	L-MIC	Y	4913072	DHS	2012	1545506	31.50%	F	T
**Costa Rica**	U-MIC	Y	4653734	None		94250*	2.0%*	F	T
**Cote d'Ivoire**	L-MIC	Y	24965279	DHS	2012	7346601	29.40%	F	T
**Croatia**	HIC	Y	4275496	None		^	^	F	F
**Cuba**	U-MIC	Y	11376669	None		241571*	2.1%*	F	T
**Curaçao**	HIC	N		None		^	^	F	F
**Cyprus**	HIC	Y	1262632	None		^	^	F	F
**Czech Republic**	HIC	Y	10592732	None		^	^	F	F
**Democratic Republic of the Congo**	LIC	Y	80884671	DHS	2014	55750504	68.90%	T	T
**Denmark**	HIC	Y	5732278	None		^	^	F	F
**Djibouti**	L-MIC	Y	1113100	MICS	2006	175698	15.80%	F	T
**Dominica**	U-MIC	Y	68933	None		1444*	2.1%*	F	T
**Dominican Republic**	U-MIC	Y	10451750	MICS	2014	100245	1.00%	F	T

**Ecuador**	U-MIC	Y	16686189	ECV	2014	118784	0.70%	F	T
**Egypt**	L-MIC	Y	96484009	DHS	2014	14539	<0.1%	F	T
**El Salvador**	L-MIC	Y	6086947	MICS	2014	153499	2.50%	F	T
**Equatorial Guinea**	U-MIC	Y	1345031	None		27277*	2.0%*	F	T
**Eritrea**	LIC	Y	5859329	None		2883986*	49.2%*	T	T
**Estonia**	HIC	Y	1314543	None		^	^	F	F
**Ethiopia**	LIC	Y	102883938	DHS	2011	83839933	81.50%	T	T
**Faroe Islands**	HIC	N		None		^	^	F	F
**Federated States of Micronesia**	L-MIC	Y	103926	None		16542*	15.9%*	F	T
**Fiji**	U-MIC	Y	906939	None		18245*	2.0%*	F	T
**Finland**	HIC	Y	5517978	None		^	^	F	F
**France**	HIC	Y	65712599	None		^	^	F	F
**French Polynesia**	HIC	N		None		^	^	F	F
**Gabon**	U-MIC	Y	1702720	DHS	2012	119855	7.00%	F	T
**Georgia**	L-MIC	Y	3691322	MICS	2005	2585	0.10%	F	T
**Germany**	HIC	Y	83294524	None		^	^	F	F
**Ghana**	L-MIC	Y	30205292	DHS	2014	3543527	11.70%	F	T
**Gibraltar**	HIC	N		None		^	^	F	F
**Greece**	HIC	Y	10402319	None		^	^	F	F
**Greenland**	HIC	Y	56155	None		^	^	F	F
**Grenada**	U-MIC	Y	110879	None		2351*	2.1%*	F	T
**Guam**	HIC	Y	167837	None		^	^	F	F
**Guatemala**	U-MIC	Y	16924208	DHS	2015	1668125	9.90%	F	T
**Guinea**	LIC	Y	11819987	DHS-MICS	2012	6532382	55.30%	T	T
**Guinea-Bissau**	LIC	Y	1855540	MICS	2014	973096	52.40%	T	T
**Guyana**	U-MIC	Y	742302	MICS	2014	13476	1.80%	F	T
**Haiti**	LIC	Y	11824835	DHS	2012	4612763	39.00%	T	T
**Honduras**	L-MIC	Y	9498815	DHS	2012	511853	5.40%	F	T
**Hong Kong SAR, China**	HIC	Burden in China		None		^	^	F	F
**Hungary**	HIC	Y	9727364	None		^	^	F	F

**Iceland**	HIC	Y	337471	None		^	^	F	F
**India**	L-MIC	Y	1380560359	IHDS	2012	197494714	14.30%	F	T
**Indonesia**	L-MIC	Y	258134673	DHS	2012	9185428	3.60%	F	T
**Iran**	U-MIC	Y	82176111	None		1614551*	2.0%*	F	T
**Iraq**	U-MIC	Y	43304442	MICS	2011	297650	0.70%	F	T
**Ireland**	HIC	Y	4860552	None		^	^	F	F
**Isle of Man**	HIC	N		None		^	^	F	F
**Israel**	HIC	Y	8949198	None		^	^	F	F
**Italy**	HIC	Y	60597480	None		^	^	F	F
**Jamaica**	U-MIC	Y	2779264	JSLC	2012	12847	0.50%	F	T
**Japan**	HIC	Y	128363257	None		^	^	F	F
**Jordan**	U-MIC	Y	10648463	DHS	2012	224	<0.1%	F	T
**Kazakhstan**	U-MIC	Y	17904334	MICS	2015	0	<0.1%	F	T
**Kenya**	L-MIC	Y	48326776	DHS	2014	18788645	38.90%	T	T
**Kiribati**	L-MIC	Y	118241	None		18991*	16.1%*	F	T
**Kosovo**	L-MIC	N		None		*	*	F	F
**Kuwait**	HIC	Y	4262200	None		^	^	F	F
**Kyrgyzstan**	L-MIC	Y	6368576	MICS	2014	1815	<0.1%	F	T
**Laos**	L-MIC	Y	6970176	MICS/DHS	2012	1164406	16.70%	F	T
**Latvia**	HIC	Y	1945427	None		^	^	F	F
**Lebanon**	U-MIC	Y	8511980	None		170166*	2.0%*	F	T
**Lesotho**	L-MIC	Y	1947578	DHS	2014	489549	25.10%	F	T
**Liberia**	LIC	Y	4722638	DHS	2013	2280072	48.30%	T	T
**Libya**	U-MIC	Y	6908740	PAPFAM	2007	981	<0.1%	F	T
**Liechtenstein**	HIC	N		None		^	^	F	F
**Lithuania**	HIC	Y	2847647	None		^	^	F	F
**Luxembourg**	HIC	Y	590547	None		^	^	F	F
**Macao SAR, China**	HIC	Burden in China		None		^	^	F	F
**Macedonia**	U-MIC	Y	2174828	MICS	2011	852	<0.1%	F	T
**Madagascar**	LIC	Y	26108785	DHS	2009	17017274	65.20%	T	T

**Malawi**	LIC	Y	17191217	DHS	2016	8329662	48.50%	T	T
**Malaysia**	U-MIC	Y	30639828	None		606871*	2.0%*	F	T
**Maldives**	U-MIC	Y	458601	DHS	2009	66	<0.1%	F	T
**Mali**	LIC	Y	20253727	DHS	2013	11757744	58.00%	T	T
**Malta**	HIC	Y	434472	None		^	^	F	F
**Marshall Islands**	U-MIC	Y	56311	None		1121*	2.0%*	F	T
**Mauritania**	L-MIC	Y	3913922	MICS	2011	1684605	43.00%	T	T
**Mauritius**	U-MIC	Y	1272112	None		25940*	2.0%*	F	T
**Mexico**	U-MIC	Y	126569720	MICS	2015	303957	0.20%	F	T
**Moldova**	L-MIC	Y	3721820	MICS	2012	10649	0.30%	F	T
**Monaco**	HIC	N		None		^	^	F	F
**Mongolia**	L-MIC	Y	3251426	MICS	2013	408585	12.60%	F	T
**Montenegro**	U-MIC	Y	626318	MICS	2013	573	0.10%	F	T
**Morocco**	L-MIC	Y	35488915	PAPFAM	2011	1836369	5.20%	F	T
**Mozambique**	LIC	Y	30035304	DHS	2011	19563037	65.10%	T	T
**Myanmar**	L-MIC	Y	52795004	DHS	2016	6803003	12.90%	F	T
**Namibia**	U-MIC	Y	2353503	DHS	2013	596001	25.30%	F	T
**Nauru**	U-MIC	N		None		*	*	F	F
**Nepal**	LIC	Y	29891524	MICS	2014	3779226	12.60%	F	T
**Netherlands**	HIC	Y	17029098	None		^	^	F	F
**New Caledonia**	HIC	N		None		^	^	F	F
**New Zealand**	HIC	Y	4448375	None		^	^	F	F
**Nicaragua**	L-MIC	Y	6396570	DHS	2012	726171	11.30%	F	T
**Niger**	LIC	Y	21375946	DHS	2012	16599772	77.70%	T	T
**Nigeria**	L-MIC	Y	206087947	DHS	2013	69834303	33.90%	T	T
**North Korea**	LIC	Y	25716644	None		12663055*	49.2%*	T	T
**Northern Mariana Islands**	HIC	Y	44878	None		^	^	F	F
**Norway**	HIC	Y	5263178	None		^	^	F	F
**Oman**	HIC	Y	4535815	None		^	^	F	F
**Pakistan**	L-MIC	Y	214287443	DHS	2013	35051746	16.40%	F	T
**Palau**	HIC	N		None		^	^	F	F

**Palestine**	L-MIC	Y	4852097	MICS	2014	0	<0.1%	F	T
**Panama**	HIC	Y	3921083	None		^	^	F	F
**Papua New Guinea**	L-MIC	Y	9227551	None		1473694*	16.0%*	F	T
**Paraguay**	U-MIC	Y	6931175	None		140074*	2.0%*	F	T
**Peru**	U-MIC	Y	33219612	DHS-Cont	2012	2779379	8.40%	F	T
**Philippines**	L-MIC	Y	103470619	DHS	2013	3380988	3.30%	F	T
**Poland**	HIC	Y	38393061	None		^	^	F	F
**Portugal**	HIC	Y	10681712	None		^	^	F	F
**Puerto Rico**	HIC	Y	3665882	None		^	^	F	F
**Qatar**	HIC	Y	2747311	None		^	^	F	F
**Romania**	U-MIC	Y	19433766	None		429249*	2.2%*	F	T
**Russian Federation**	U-MIC	Y	146189867	None		3123509*	2.1%*	F	T
**Rwanda**	LIC	Y	12554172	DHS	2015	6170616	49.10%	T	T
**Saint Lucia**	U-MIC	Y	176474	MICS	2012	316	0.20%	F	T
**Saint Vincent and the Grenadines**	U-MIC	Y	114112	None		2361*	2.1%*	F	T
**Samoa**	U-MIC	Y	198918	None		4127*	2.1%*	F	T
**San Marino**	HIC	N		None		^	^	F	F
**Sao Tome and Principe**	L-MIC	Y	200206	MICS	2014	17492	8.70%	F	T
**Saudi Arabia**	HIC	Y	34444054	None		^	^	F	F
**Senegal**	LIC	Y	14688034	DHS	2015	4526503	30.80%	F	T
**Serbia**	U-MIC	Y	8874098	MICS	2014	8619	0.10%	F	T
**Seychelles**	HIC	Y	100907	None		^	^	F	F
**Sierra Leone**	LIC	Y	7829749	DHS	2013	4871383	62.20%	T	T
**Singapore**	HIC	Y	5568481	None		^	^	F	F
**Sint Maarten (Dutch part)**	HIC	N		None		^	^	F	F
**Slovakia**	HIC	Y	5419210	None		^	^	F	F
**Slovenia**	HIC	Y	2068856	None		^	^	F	F
**Solomon Islands**	L-MIC	Y	637607	None		102922*	16.1%*	F	T
**Somalia**	LIC	Y	16880386	MICS	2006	12875624	76.30%	T	T
**South Africa**	U-MIC	Y	54952860	NIDS	2015	1314104	2.40%	F	T
**South Korea**	HIC	Y	52670741	None		^	^	F	F

**South Sudan**	LIC	Y	9941010	MICS	2010	8819371	88.70%	T	T
**Spain**	HIC	Y	46389213	None		^	^	F	F
**Sri Lanka**	L-MIC	Y	21596437	None		3465702*	16.1%*	F	T
**St. Kitts and Nevis**	HIC	N		None		^	^	F	F
**St. Martin (French part)**	HIC	N		None		^	^	F	F
**Sudan**	L-MIC	Y	40255551	MICS	2014	18536578	46.00%	T	T
**Suriname**	U-MIC	Y	572463	MICS	2010	12875	2.20%	F	T
**Swaziland**	L-MIC	Y	1124436	MICS	2014	102401	9.10%	F	T
**Sweden**	HIC	Y	10044996	None		^	^	F	F
**Switzerland**	HIC	Y	8593087	None		^	^	F	F
**Syria**	LIC	Y	18131194	PAPFAM	2009	12364	0.10%	F	T
**Taiwan**	HIC	Y	23583126	None		^	^	F	F
**Tajikistan**	LIC	Y	9243717	DHS	2012	84087	0.90%	F	T
**Tanzania**	LIC	Y	53973136	DHS	2016	30353223	56.20%	T	T
**Thailand**	U-MIC	Y	70626063	MICS	2012	34427	<0.1%	F	T
**The Bahamas**	HIC	Y	375441	None		^	^	F	F
**The Gambia**	LIC	Y	2132510	DHS	2013	503255	23.60%	F	T
**Timor-Leste**	L-MIC	Y	1287486	DHS	2010	659356	51.20%	T	T
**Togo**	LIC	Y	7516030	DHS	2014	2344458	31.20%	F	T
**Tonga**	U-MIC	Y	102838	None		2126*	2.1%*	F	T
**Trinidad and Tobago**	HIC	Y	1391805	MICS	2006	503	<0.1%	F	T
**Tunisia**	L-MIC	Y	11442183	MICS	2012	22155	0.20%	F	T
**Turkey**	U-MIC	Y	80456851	None		1635775*	2.0%*	F	T
**Turkmenistan**	U-MIC	Y	4976883	MICS	2016	0	<0.1%	F	T
**Turks and Caicos Islands**	HIC	N		None		^	^	F	F
**Tuvalu**	U-MIC	N		None		*	*	F	F
**Uganda**	LIC	Y	39078382	DHS	2011	22831657	58.40%	T	T
**Ukraine**	L-MIC	Y	44689084	MICS	2012	4751	<0.1%	F	T
**United Arab Emirates**	HIC	Y	9734100	None		^	^	F	F
**United Kingdom**	HIC	Y	66635515	None		^	^	F	F
**United States**	HIC	Y	324839024	None		^	^	F	F

**Uruguay**	HIC	Y	3421532	None		^	^	F	F
**Uzbekistan**	L-MIC	Y	32236895	MICS	2006	23459	0.10%	F	T
**Vanuatu**	L-MIC	Y	287568	MICS	2007	79805	27.80%	F	T
**Venezuela**	U-MIC	Y	30831741	None		620769*	2.0%*	F	T
**Vietnam**	L-MIC	Y	96140732	MICS	2014	817839	0.80%	F	T
**Virgin Islands, U.S.**	HIC	Y	104952	None		^	^	F	F
**Yemen**	LIC	Y	30449159	DHS	2013	6310934	20.70%	F	T
**Zambia**	L-MIC	Y	17364088	DHS	2014	8113994	46.70%	T	T
**Zimbabwe**	LIC	Y	14713754	DHS	2015	4204692	28.60%	F	T

^ High income country, no survey data, assumed 0% in the poorest billion
* LMIC, no survey data, used age- and sex-specific average across countries in income category

Following the methodology used by the Oxford Poverty and Human Development Initiative, on almost every survey, the members of the household counted are “usual residents,” except for the following three cases. On the Jamaica JSLC, people who spent the night prior to the interview were considered household members (de facto population). In the case of Ecuador ECV, individuals listed in the household except for 'pensioners' and 'other non-relatives' were considered members of the household. In the case of India IHDS, people interviewed who 'live and take meals in this house' were considered members of the household.

Analytic Approaches

A summary of the different analytic approaches is given in Appendix Table 3. Details on the methods are presented below, and differences in results are discussed in the discussion section of the main text and appendix. Results presented in the main text are from the Selective Ecological approach.

Appendix Table 3: Characteristics of analytical burden groups

Method Name	Countries Included	Total Population Included	Description of Population	Burden Methods Notes
**Low-income**	34	725 million	Low-income countries by World Bank per capita income definition	Sum of country-level burden
**Country Aggregation**	31	873 million	Poorest countries ranked by extreme poverty prevalence on poverty index	Sum of country-level burden
**Poorest Billion Aggregation**	139	873 million	Population in 139 countries living in households deprived in five of eight indicators on the poverty index	Age- and sex-specific rates assumed homogeneous across wealthy/poor within countries, but burden aggregated using fractions of country population in poorest billion by our survey definition
**Full Ecological**	139	873 million	Population in 139 countries living in households deprived in five of eight indicators on the poverty index	Predictions of age- and sex-specific rates based on cross-country ecological relationships for those in extreme poverty and not in extreme poverty, scaled to country-level GBD estimates
**Selective Ecological**	139	873 million	Population in 139 countries living in households deprived in five of eight indicators on the poverty index	Same as Full Ecological approach, but ecological predictions only used for conditions that show concordance between ecological relationships and expert perceptions of within-country relationships between poverty and disease. Poorest Billion Aggregation approach used for other conditions.

As described in the main text, each of these approaches utilized national-level estimates of disease burden from the Global Burden of Disease Study 2017.[1–3]

Low-Income Approach

For the Low-Income approach, we used the publicly available GBD estimates to aggregate the disease burden in countries defined as low-income by the World Bank 2017 calendar year income groups.[4] The size of the population in low-income countries (725 million) was not the same as for our other definitions, but it serves as a familiar group of reference. We use the disease burden in other World Bank income groups (lower-middle-income, upper-middle-income, high-income) for comparison. In addition, we show results for “high-income regions,” which we aggregated from the following GBD regions: Western Europe, high-income North America, high-income Asia-Pacific, and Australasia (Australia and New

Zealand). The main distinction from the World Bank high-income countries was the exclusion of some of the countries whose per capita wealth is driven largely by oil and whose health patterns less closely match other high-income countries. About 54% of the population in the poorest billion as defined by our poverty index lived outside of countries classified as Low Income.

Country Aggregation Approach

Like the 1999 Gwatkin analysis, we defined the population in the poorest billion for the Country Aggregation approach without using poverty measures to disaggregate populations within countries.[5] Gwatkin and colleagues defined the global poor as the people living in the poorest countries by per capita gross domestic product (GDP), aggregated by rank to cumulatively sum to 20% of the global population, close to 1 billion people at the time. In contrast, we used national prevalence of the poorest billion from our household poverty index definition to define country ranking. To reach the same total population as in our poorest billion as defined by our poverty index (873 million), we took the first 30 countries from this list, as well as 82% of the population and burden (for each age and sex group) of the 31st country, Nigeria. The set of countries in the Country Aggregation approach and Low-Income approach were largely congruent, but there were seven countries in the Country Aggregation approach that are not present in the Low-Income approach and ten countries present in the Low-Income approach that were not present in the Country Aggregation approach because of differences in per capita GDP and prevalence of extreme poverty on our poverty index. The low-income countries not included in the Country Aggregation approach were Afghanistan, Comoros, Nepal, Senegal, Syria, Tajikistan, The Gambia, Togo, Yemen, and Zimbabwe. Meanwhile, the countries in the Country Aggregation approach that were not included in the set of low-income countries are Cameroon, Kenya, Mauritania, Nigeria, Sudan, Timor-Leste, and Zambia. The Low-Income and Country Aggregation approaches defined the poorest billion in a manner that allowed the use of more established country-level disease burden estimates; however, they did not fully utilize information about where the poorest live from our poverty index. About 43% of the population in the poorest billion defined by our poverty index lived outside of the countries included in the country aggregation approach.

Poorest Billion Aggregation Approach

For the Poorest Billion Aggregation approach, we used the country-level rates of burden as the rates for both the poorest billion and non-poorest within the country. The aggregate poorest billion burden was created by multiplying age- and sex-specific rates of the relevant measures (deaths, YLLs, YLDs, DALYs) by the corresponding age- and sex-specific populations in the poorest billion, by location. For example the total number for a given measure, m, in age a, and sex s in location l is given by:

$$Count_{m,a,s,l}^{Poorest\ Billion} = Rate_{m,a,s,l}^{National\ GBD} * Population_{a,s,l}^{Poorest\ Billion}$$

The total of any measure of disease for the poorest billion was then created by aggregating the burden across locations. This strategy did not distinguish the within-country differences in burden between the poorest billion and the non-poorest, only accounting for the diverse geographic locations in which the poorest billion live. In effect, this assumed homogeneous burden across poverty levels within countries should bias results towards showing less difference in disease burden between the wealthy and the poorest. Some approximation of the within-country differences would be necessary to accurately estimate the disease burden.

Full Ecological Analysis Approach

Comprehensive empirical data describing rates of disease by individual poverty status within LMICs are largely unavailable. For the Ecological Analysis approaches we used the relationships of disease rates across countries with respect to the prevalence of the poorest billion to make assumptions about possible

within-country differences in disease rates by poverty. We modeled the log of the death and YLD rates in location, l, on the percent of population in the poorest billion, PPB, with a random intercept on the region, r, to better isolate the associations with poverty prevalence rather than regional differences (for each age and sex group separately):

$$\log(Measure\ rate_l) = \beta_0 + \beta_1 PPB_l + \gamma_r + \varepsilon$$

We limited the set of countries included in this model to those for which we had survey estimates of extreme poverty and with prevalence of poorest billion populations of at least 2% in the given age-sex group so that variations in other factors among countries with no poverty prevalence did not drive the association. We then made predictions for the rate of the deaths and YLDs for hypothetical groups in which 100% of the population is in the poorest billion and 0% of the population is in the poorest billion by substituting these for PPB_l. These predicted rates were then rescaled using the poorest billion and non-poorest populations in each of the 139 countries to aggregate to the national GBD estimates. The below equation shows how a scalar was derived to rescale the rates for the poorest billion and non-poorest within each country such that they aggregate to the national estimates, where *pb* indicates poorest billion and *npb* indicates non-poorest for a given location, *l*, age, *a*, sex, *s*, and cause of death or disability, *c*.

$$k\left(Rate_{l,a,s,c}^{pb} * Pop_{l,a,s}^{pb} + Rate_{l,a,s,c}^{npb} * Pop_{l,a,s}^{npb}\right) = Rate_{l,a,s,c}^{GBD} * Pop_{l,a,s}^{GBD}$$

After solving for the scalar *k*, it was multiplied by the predicted rates among the poorest billion and non-poorest to calculate rates in the poorest billion and non-poorest populations in the given age and sex group. This method preserved the same ratio of poorest billion to non-poor rates across countries but ensured that the age- and sex-specific results for the poorest billion and non-poorest aggregate to the national GBD results. For the Full Ecological Analysis approach, we modeled every condition except for those in which the number of countries with non-zero rates in a given age and sex group was too small (fewer than 10 countries) to create stable results. We used the national-level death and YLD rates for both the poorest and non-poorest for these conditions.

Selective Ecological Analysis Approach

In the Selective Ecological Analysis approach, we used the methods described for the Full Ecological Analysis Approach but chose to model only conditions for which the ecological relationship might plausibly represent the relationship between poverty and disease within countries. To choose the conditions for which we used the results from the ecological model, we first conducted a survey of perceived relationships between poverty and disease in LMICs among 97 health practitioners and researchers with a broad range of disease expertise and experience working in or researching health in LMICs. Respondents came from three broad categories: (1) commissioners on the *Lancet* NCDI Poverty Commission, (2) members of the 11 national NCDI Poverty Commissions and Groups associated with the *Lancet* Commission, and (3) experts identified in specific disease areas to ensure coverage of the diverse set of diseases in the GBD. We acknowledge each of the participants in the appendix. Descriptive characteristics of the respondents are included later in the appendix, including region of residence, region of expertise, areas of work (clinical, research), and experience in LMICs. Participants answered questions about their perceived relationships between rates of diseases and poverty within LMICs, indicating whether they thought occurrence (defined in survey instructions as incidence) rates and fatality (defined as case fatality) rates were (1) much higher in the non-poorest, (2) higher in the non-poorest, (3) not different, (4) higher in the poorest, or (5) much higher in the poorest. The survey also asked respondents how confident they were in their selection.

For graphical representation of the results, we display the range of responses from “Much Higher in the Non-Poor” to “Much Higher in the Poor” on a continuous scale with each answer one unit apart (i.e. “Much Higher in the Non-Poor” = -2, “Higher in the Non-Poor” = -1, “No Difference” = 0, “Higher in the

Poor" = 1, "Much Higher in the Poor" = 2). For statistical analysis, we treated these answers as ordinal and used the non-parametric one sample, two-sided sign test to test for a difference in the median from the "No Difference" response. We described uncertainty using the spread of the responses and the self-reported certainty about each answer.

To choose conditions to model using the Selective Ecological Approach, we found the set of conditions with significant differences in rates between poorest billion and non-poorest from the expert perception survey and which showed agreement with the direction of the association in the ecological model in over half of age groups. For those conditions, we used the estimates from the Full Ecological Analysis. For conditions without agreement between expert opinion and the ecological associations, we used the national-level death and YLD rates for both the poorest and non-poorest.

Methodological Consistency

To ensure consistency in how we aggregated conditions for the ecological analysis approaches, we conducted these regressions and predictions at the lowest-level causes within the GBD hierarchy and aggregated up levels (e.g. ischemic heart disease aggregated to cardiovascular diseases, then to NCDs). We also wanted to ensure consistency in the measures of burden. In GBD, YLLs are calculated using age-sex-specific differences between ages of death and a standard life expectancy. In instances in which we used the ecological predictions, we created YLLs directly from our predicted deaths by multiplying the country-level age-, sex-, and cause-specific YLL to death ratios by the predicted deaths. We created DALYs by adding YLDs and YLLs. To ensure consistency in aggregate age groups or for both sexes combined, we created aggregate results by adding the most specific age and sex groupings. We created age-standardized results using the GBD 2017 population age standard.[2]

References

1 Kyu HH, Abate D, Abate KH, *et al.* Global, regional, and national disability-adjusted life-years (DALYs) for 359 diseases and injuries and healthy life expectancy (HALE) for 195 countries and territories, 1990–2017: a systematic analysis for the Global Burden of Disease Study 2017. *The Lancet* 2018; **392**: 1859–922.

2 Roth GA, Abate D, Abate KH, *et al.* Global, regional, and national age-sex-specific mortality for 282 causes of death in 195 countries and territories, 1980–2017: a systematic analysis for the Global Burden of Disease Study 2017. *The Lancet* 2018; **392**: 1736–88.

3 James SL, Abate D, Abate KH, *et al.* Global, regional, and national incidence, prevalence, and years lived with disability for 354 diseases and injuries for 195 countries and territories, 1990–2017: a systematic analysis for the Global Burden of Disease Study 2017. *The Lancet* 2018; **392**: 1789–858.

4 The World Bank. World Bank Country and Lending Groups. 2019. https://datahelpdesk.worldbank.org/knowledgebase/articles/906519-world-bank-country-and-lending-groups (accessed June 11, 2020).

5 Gwatkin DR, Guillot M. The Burden of Disease among the Global Poor: Current Situation, Future Trends, and Implications for Strategy. Washington (DC): The World Bank and Global Forum for Health Research, 1999.

Survey Methods and Acknowledgment

Survey participants were recruited from three broad categories: (1) commissioners on the *Lancet* NCDI Poverty Commission, (2) members of the 11 national NCDI Poverty Groups associated with the *Lancet* Commission, and (3) experts identified in specific disease areas to ensure coverage of the diverse set of diseases in the GBD. Recruitment was via email. The survey was conducted June 7, 2017 to September 30, 2017.

We deeply appreciate the time and contributions that each of the experts made in participating in the survey, listed below.

Abdul Qadir Qadir, Abha Shrestha, Abraham Haileamlak, Adamson Muula, Agnes Binagwaho, Allison Linden, Amelia (Mia) Crampin, Ann Miller, Aruyaru Stanley Mwenda, Benjamin Massenburg, Bhagawan Koirala, Bhaskar Pant, Bistra Zheleva, Bongani Mayosi, Crispin Gishoma, D Cristina Stefan, Dagnaw Wubaye Walelgne, Damian Hoy, Daniel Vigo, Dinesh Neupane, Mariam Kalomo, Cyprian Kamau, Eleana Stoufi, Eugene Richardson, Farhad Farewar, Ferozuddin Feroz, Fred Amegashie, Gedeon Ngoga, Gertrudes Machatine, Gladwell Gathecha, Hanna Kaade, Hema Magge, Hideki Higashi, Homa Akseer, Jacques Clerville, Jane Barrow, Jason Beste, Jean Pascal Blaise Uhagaze, Jennifer Furin, John Chipolombwe , John G Meara, John H. Kempen, Joia Mukherjee, Jonathan Steer-Massaro, Jones Masiye, Joseph Mucumbitsi, Judith Lindert, Julia von Oettingen, Julie Makani, Katie Cundale, Kerry Vaughan, Krishna Kumar Aryal, Leo Masamba, Liesl Zuhlke, Lillian Gondwe-Chunda, Loise Nyanjau, Luckson Dullie, Luke L. Bawo, Marie Nancy Charles Larco, Michael Udedi, Mieraf Taddesse Tolla, Muhimpundu Marie Aimee, Munirat Ogunlayi, Natasha Archer, Neil Gupta, Nelya Melnitchouk, Nicholas Campain, Nobhojit Roy, Oriol Mitjà, Paul Heidekrueger, Piet Noë, Rachel Koch, Rachel Nugent, Roderick Hay, Roger Jean-Charles, Sabrina Juran, Sam Patel, Sam Slewion, Samuel Oti, Sanctus Musafiri, Sarah Averbach, Saroj Ojha, Shada Rouhani, Sonya Shin, Thomas Koestler, Thomas Randall, Tim Walker, Valeria Macias, Valerie Luyckx, Wilnique Pierre, Yassir Turki, Yogesh Jain, Yogeshwar Kalkonde, Yoseph Mamo Azmera

The survey was administered online using the web browser-based Qualtrics interface. The survey typically took respondents between 10 and 45 minutes, though respondents were allowed to pause and return to the survey at a later time. The survey consisted of questions about the respondent's geographic background, training, and areas of expertise, followed by questions about the relationship between disease rates and poverty in disease areas with which the respondent self-identified as having experience. Some respondents had clinical experience, while others had epidemiological or research experience with the conditions. Appendix Table 1 describes the characteristics of the respondents in more detail.

Before participants answered questions on poverty and disease, they were presented with descriptions of the multidimensional definition of poverty that we used. They were given context for percentages of the population in a range of countries in the poorest billion. They were then asked to answer the questions with regard to those in the poorest billion versus the non-poorest within an LMIC. They were asked questions about both occurrence (incidence) and fatality (case fatality) of the diseases in the categories for which they had experience, expressing both their perception of the relationship between the disease and poverty, as well as their confidence in their response. We chose qualitative categorizations because we received feedback from pilot surveys that asking for quantitative estimates about these relationships was too difficult. The categories that respondents could choose were "Much Higher in the Non-Poor", "Higher in the Non-Poor", "No Difference", "Higher in the Poor", and "Much Higher in the Poor."

We also identified four out of the total 97 respondents who had either internally inconsistent responses or whose responses did not match the content of the survey and removed those outliers from the results.

Appendix Table 4: Characteristics of expert survey respondents

	**Category**	**Number of Respondents (Percentage)**
**Gender**	Female	40 (41.2%)
**Region of Nationality**	High-Income	37 (38.1%)
	Latin America and Caribbean	6 (6.2%)
	Central Europe, Eastern Europe, Central Asia	2 (2.1%)
	North Africa and Middle East	2 (2.1%)
	South Asia	13 (13.7%)
	Sub-Saharan Africa	37 (38.1%)
**Region of Residence**	High-Income	33 (34.0%)
	Latin America and Caribbean	4 (4.1%)
	North Africa and Middle East	1 (1.0%)
	South Asia	13 (13.4%)
	Southeast Asia, East Asia, and Oceania	1 (1.0%)
	Sub-Saharan Africa	45 (46.4%)
**Regions of Expertise (can be more than one)**	East Asia and Pacific	13 (13.4%)
	Europe and Central Asia	8 (8.2%)
	Latin America and Caribbean	24 (24.7%)
	Middle East and North Africa	6 (6.2%)
	South Asia	19 (19.6%)
	Sub-Saharan Africa	69 (71.1%)
**Areas of Work (can be more than one)**	Clinical	67 (69.1%)
	Epidemiology/Health Metrics	34 (35.1%)
	Health Systems/Health Policy	52 (53.6%)
	Other (demography, ethics, social science, education, health communication)	9 (9.3%)
**Training (can be more than one)**	Medical Doctor, Nurse, or equivalent	78 (80.4%)
	PhD or equivalent	24 (24.7%)
	Master’s in Public Health or Science	44 (45.4%)
	None of the Above	0 (0%)
**Years of Experience on Health in LMICs**	<2 years	4 (4.1%)
	2-4 years	9 (9.3%)
	5-9 years	20 (20.6%)
	10-19 years	36 (37.1%)
	20+ years	28 (28.9%)

To inform which conditions we modeled for the Selective Ecological Approach, we identified conditions for which experts perceived significant differences in either fatality or occurrence between the poorest and non-poorest. The underlying assumptions are that YLDs follow incidence and that increases in either incidence or case fatality lead to increases in death rates. We chose to model only conditions for which there was relative certainty based on expert responses. The decisions to model can be summarized as follows, where the default decision is not to use the ecological model:

For purely nonfatal conditions in the GBD study:

If there was a significant difference in occurrence based on the expert survey AND the direction of that difference was the same as the direction for YLDs from the ecological model, we used the modeled results

For conditions that can be fatal in the GBD study:

If there was a significant difference in fatality or occurrence based on the expert survey AND the direction of the expert perceptions for fatality and occurrence were not in opposite directions from each other AND the direction of the result from the expert survey was in the same direction of the death rate from the ecological model, we used the ecological model.

Since the death rate affects the prevalence and therefore YLDs, if we used the model for the death rate, we used the model for YLDs as well. For some conditions, the direction of the associations in the ecological analysis varied by age. In some cases, that makes logical sense- higher death rates at younger ages can lead to lower death rates at older ages if it leads to fewer people with the condition. We chose to model conditions if the above rules were met in at least 50% of age groups and in both sexes (or, for sex-specific diseases like maternal disorders, then for that one sex). If criteria for using the ecological relationship were not met, we assumed no difference in rates between the poorest and non-poorest within countries (see methods description for Selective Ecological Approach).

The initial survey was conducted using a cause list from a previous version of the GBD Study—some conditions were mapped between the survey and the GBD 2017 cause list for assessment.

The list of conditions for which we used the ecological associations in the Selective Ecological Approach is below.

Acute hepatitis A, Acute hepatitis B, Acute hepatitis C, Acute hepatitis E, African trypanosomiasis, Ascariasis, Cervical cancer, Chlamydial infection, Cirrhosis and other chronic liver diseases due to alcohol use, Cirrhosis and other chronic liver diseases due to hepatitis B, Cirrhosis and other chronic liver diseases due to hepatitis C, Coal workers pneumoconiosis, Cutaneous and mucocutaneous leishmaniasis, Cystic echinococcosis, Cysticercosis, Diarrheal diseases, Dietary iron deficiency, Diphtheria, Drowning, Drug-susceptible tuberculosis, Ectopic pregnancy, Environmental heat and cold exposure, Epilepsy, Esophageal cancer, Falls, Fire, heat, and hot substances, Foreign body in eyes, Fungal skin diseases, Genital herpes, Gonococcal infection, Gout, H influenzae type B meningitis, HIV/AIDS-Drug-susceptible Tuberculsosis, HIV/AIDS resulting in other diseases, Hookworm disease, Indirect maternal deaths, Intracerebral hemorrhage, Invasive Non-typhoidal Salmonella (iNTS), Iodine deficiency, Late maternal deaths, Leprosy, Liver cancer due to alcohol use, Liver cancer due to hepatitis B, Liver cancer due to hepatitis C, Lower respiratory infections, Lymphatic filariasis, Malaria, Maternal abortion and

miscarriage, Maternal deaths aggravated by HIV/AIDS, Maternal hemorrhage, Maternal hypertensive disorders, Maternal obstructed labor and uterine rupture, Maternal sepsis and other maternal infections, Measles, Meningococcal meningitis, Multidrug-resistant tuberculosis without extensive drug resistance, Neonatal encephalopathy due to birth asphyxia and trauma, Neonatal preterm birth, Neonatal sepsis and other neonatal infections, Neural tube defects, Non-venomous animal contact, Onchocerciasis, Other meningitis, Other pneumoconiosis, Otitis media, Paratyphoid fever, Peptic ulcer disease, Pneumococcal meningitis, Protein-energy malnutrition, Rabies, Rheumatic heart disease, Scabies, Schistosomiasis, Subarachnoid hemorrhage, Syphilis, Tetanus, Trachoma, Trichomoniasis, Trichuriasis, Typhoid fever, Upper respiratory infections, Varicella and herpes zoster, Venomous animal contact, Visceral leishmaniasis, Vitamin A deficiency, Whooping cough, Yellow fever

Appendix Figure 1: Age and sex distributions of various populations

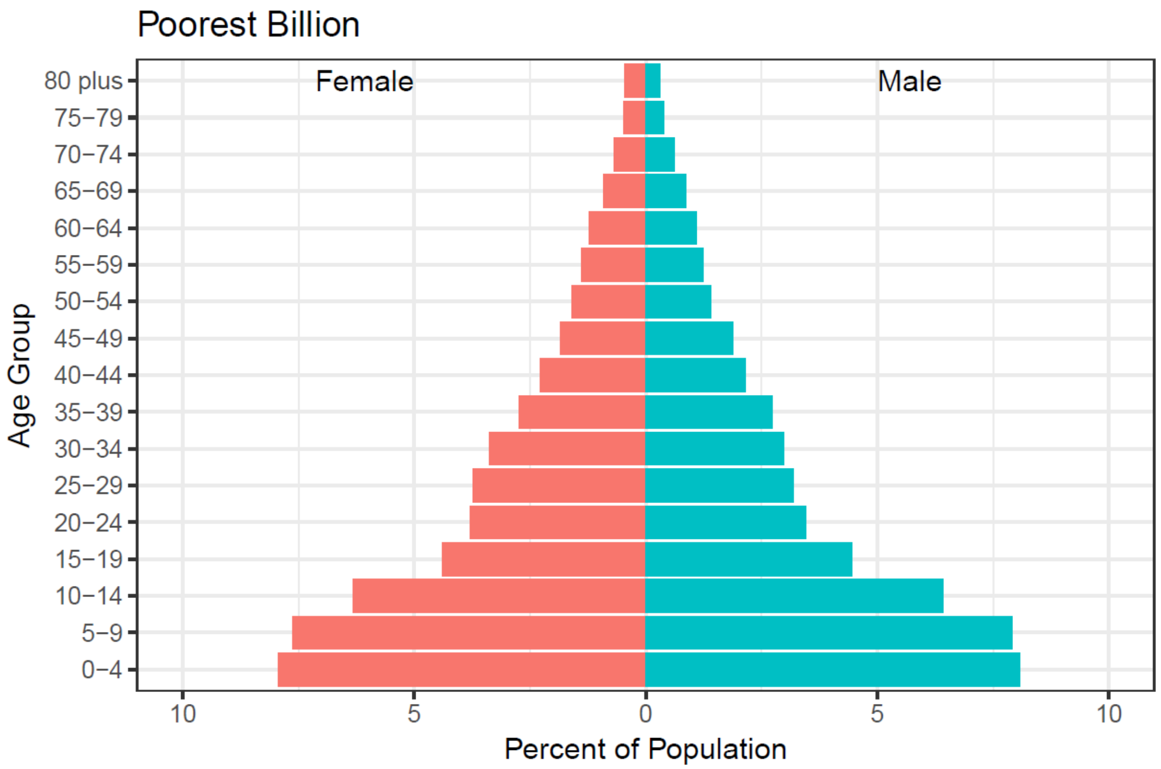

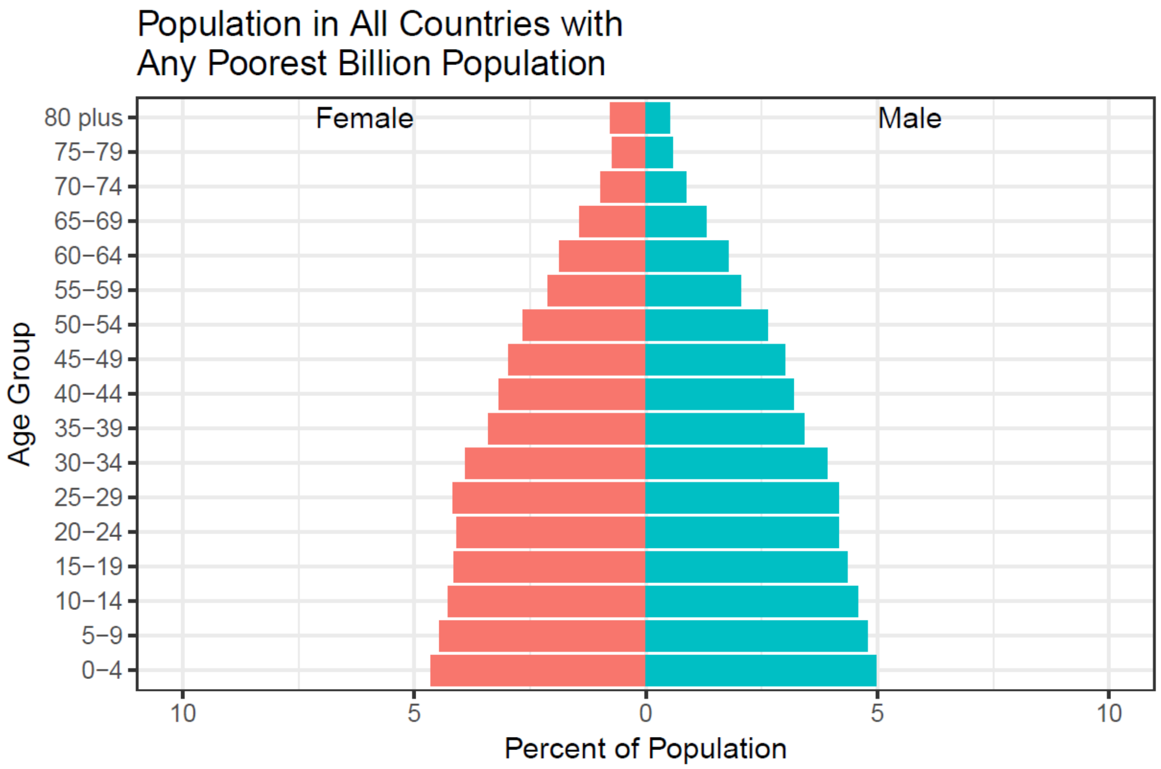

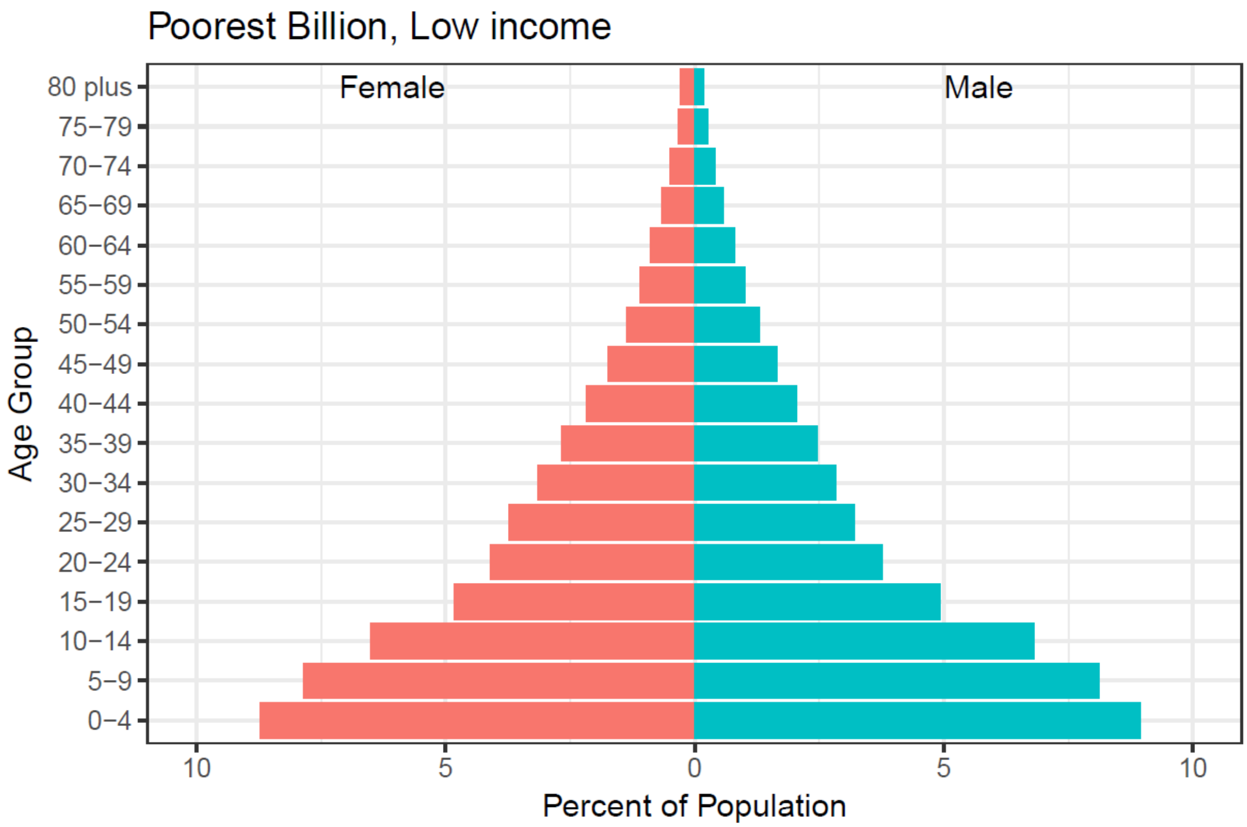
Poorest Billion, Low income
Female
Male
80 plus
75−79
70−74
65−69
60−64
55−59
50−54
45−49
40−44
35−39
30−34
25−29
20−24
15−19
10−14
5−9
0−4
Age Group
10
5
0
5
10
Percent of Population

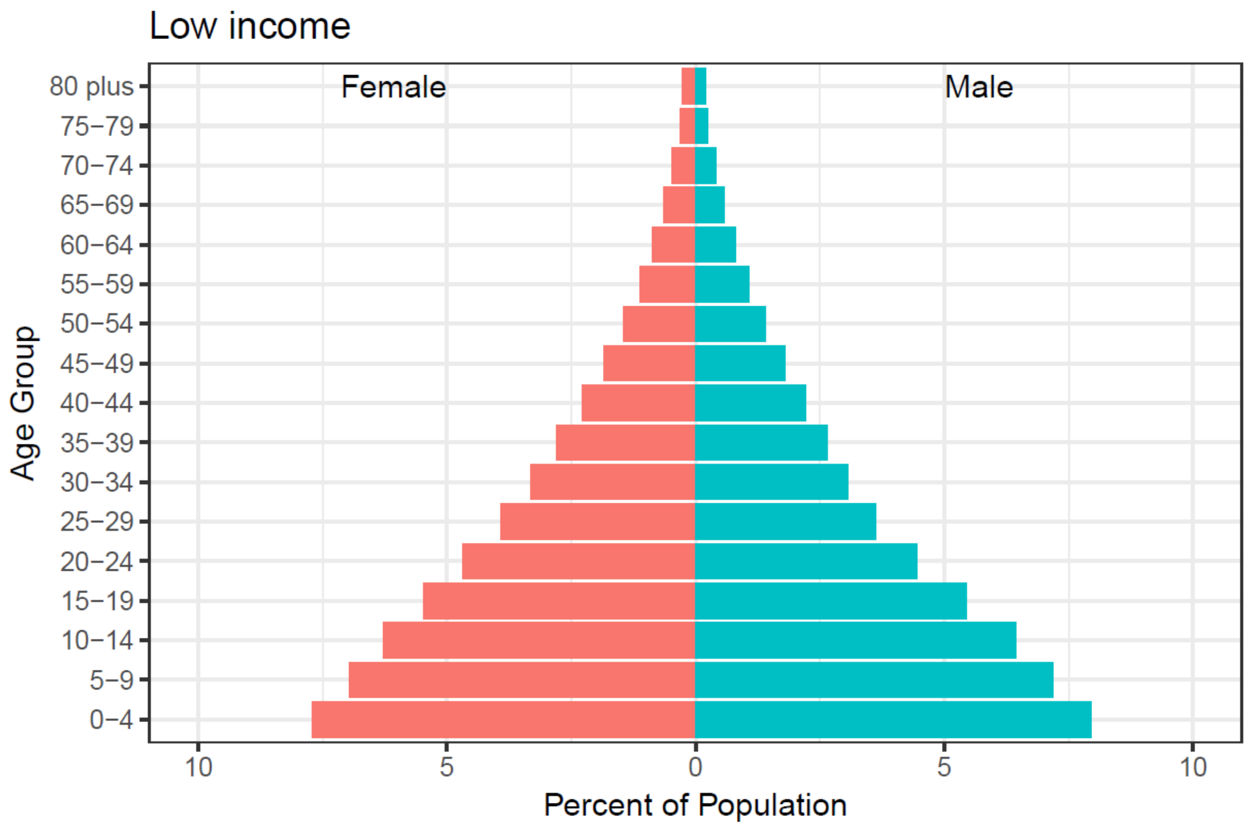
Low income
Female
Male
80 plus
75−79
70−74
65−69
60−64
55−59
50−54
45−49
40−44
35−39
30−34
25−29
20−24
15−19
10−14
5−9
0−4
Age Group
10
5
0
5
10
Percent of Population

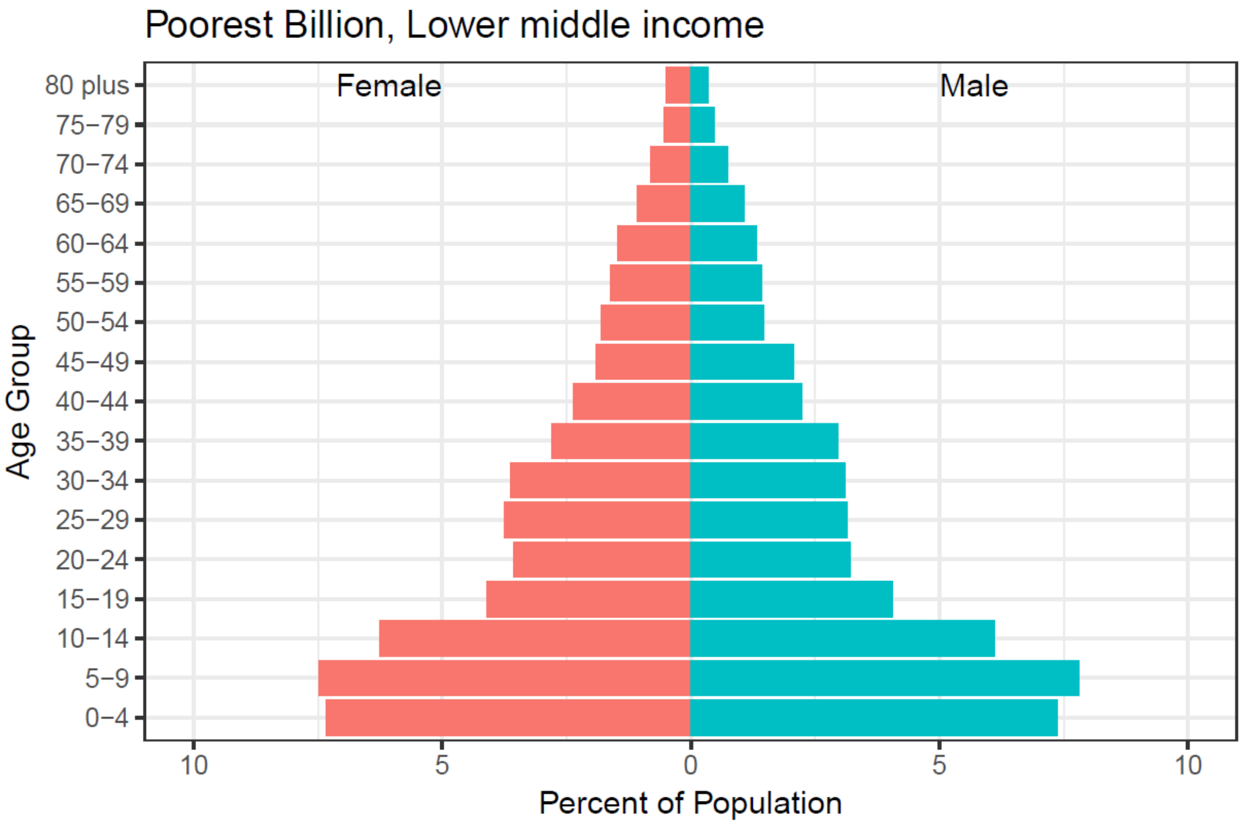
Poorest Billion, Lower middle income
Female
Male
80 plus
75−79
70−74
65−69
60−64
55−59
50−54
45−49
40−44
35−39
30−34
25−29
20−24
15−19
10−14
5−9
0−4
Age Group
10
5
0
5
10
Percent of Population

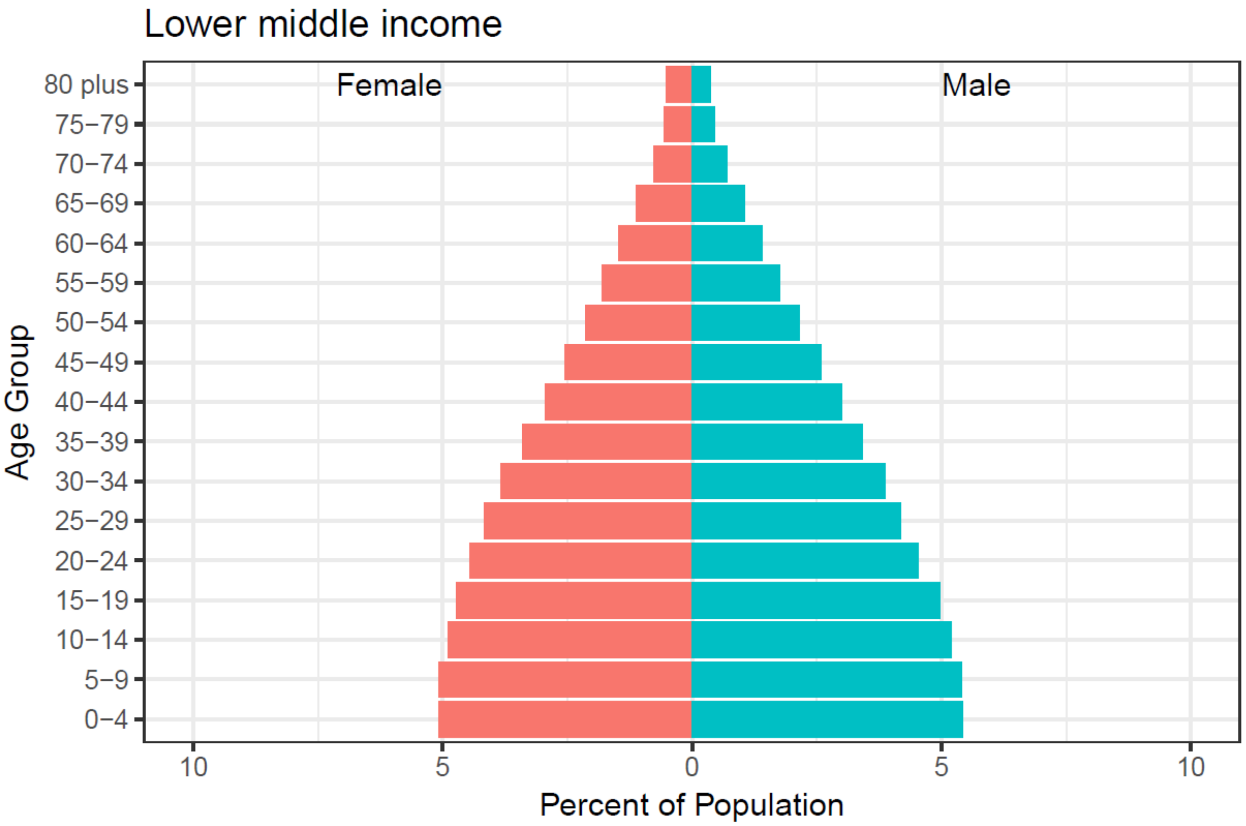
Lower middle income
Female
Male
80 plus
75−79
70−74
65−69
60−64
55−59
50−54
45−49
40−44
35−39
30−34
25−29
20−24
15−19
10−14
5−9
0−4
Age Group
10
5
0
5
10
Percent of Population

Poorest Billion, Upper middle income

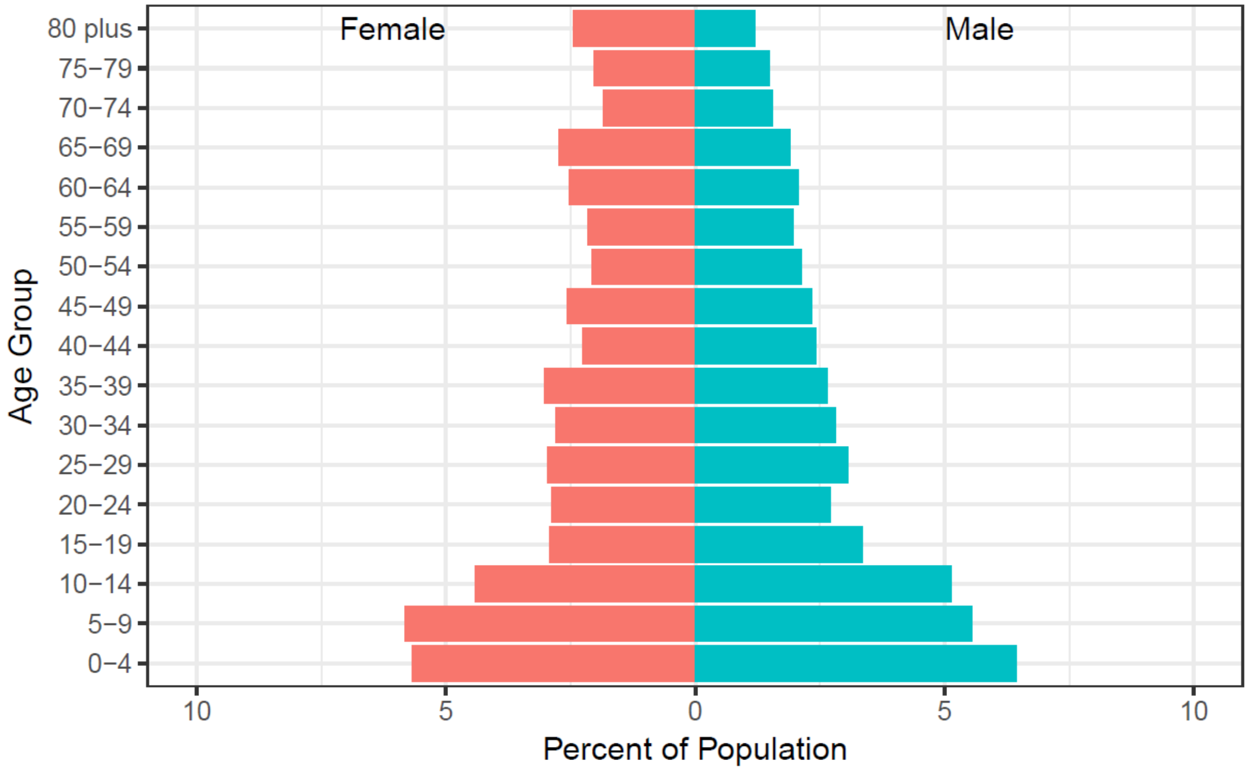

Upper middle income

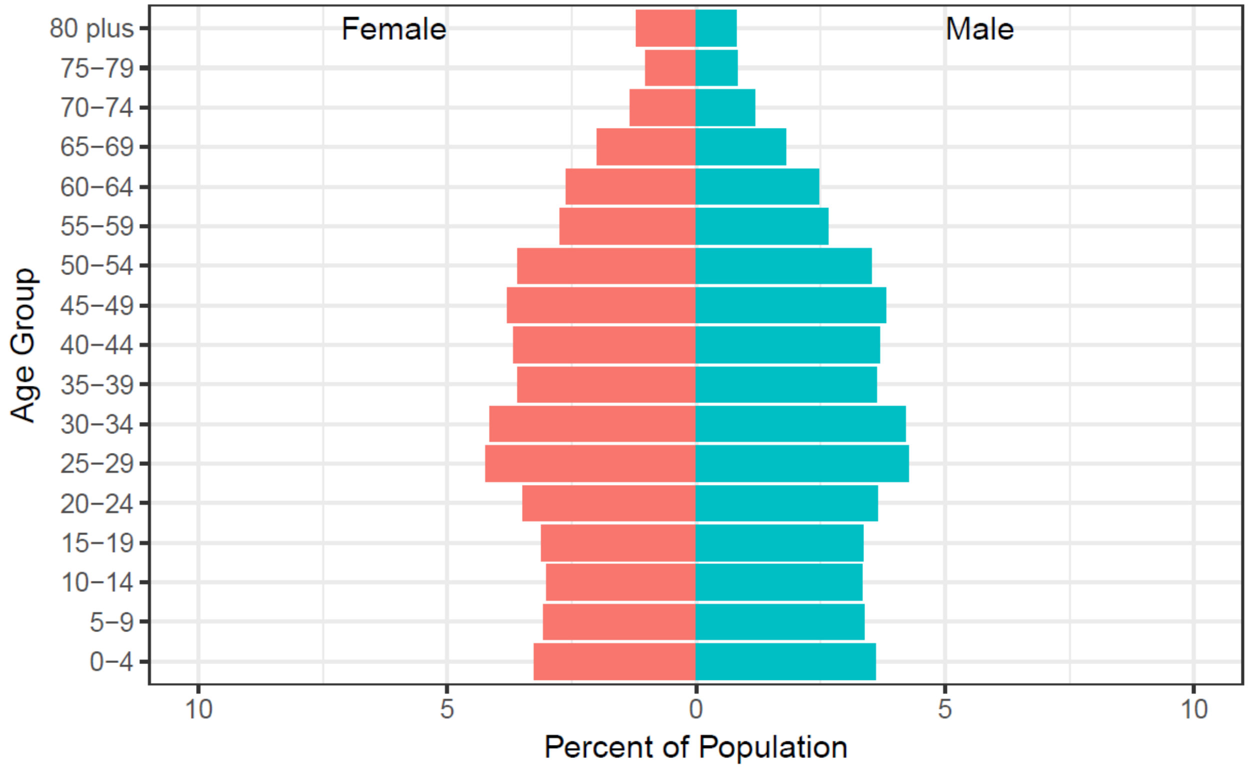

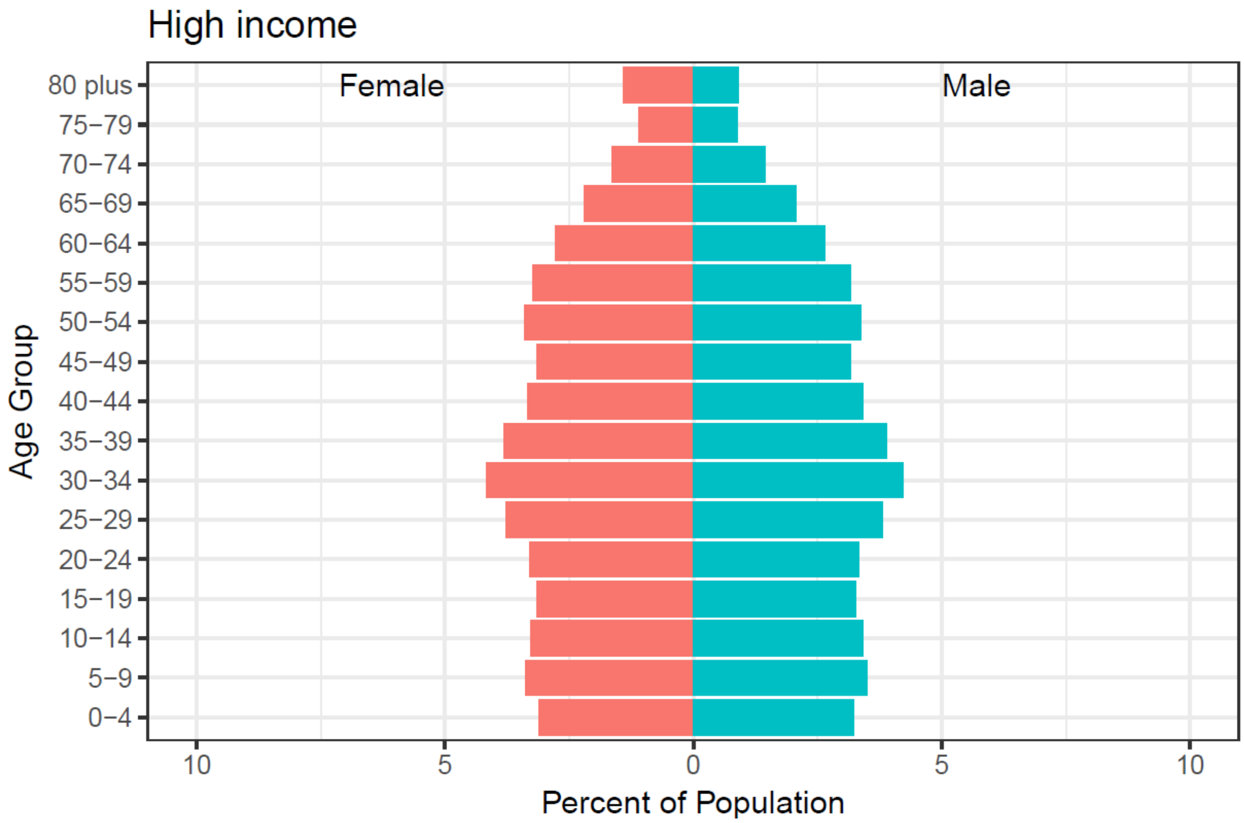

Appendix Table 5. Percent of the population below specified ages in various groups

	Under 5	Under 20	Under 30	Under 40	Under 50	Under 75
**Poorest Billion**	16.0	53.1	67.3	79.1	87.3	98.4
**Countries with any Poorest Billion Population**	9.6	36.2	52.8	67.4	79.7	97.4
**Poorest Billion, Low income**	17.7	56.7	71.5	82.7	90.3	98.9
**Low income**	15.7	53.5	70.1	82.0	90.1	99.0
**Poorest Billion, Lower middle income**	14.7	50.5	64.3	76.7	85.3	98.1
**Lower middle income**	10.5	40.8	58.1	72.7	83.8	98.1
**Poorest Billion, Upper middle income**	12.1	39.3	50.9	62.3	71.9	92.8
**Upper middle income**	6.9	26.1	41.7	57.3	72.2	96.1
**High income**	6.4	26.4	40.6	56.7	69.7	95.7

Survey of Expert Perceptions Results

Our survey showed perceived differences in incidence between the poorest billion and non-poorest within LMICs in all 61 communicable, maternal, neonatal, and nutritional diseases (CMNNs), 39 of 143 NCDs, and 13 of 19 injuries (Fig 2). Most of these conditions with perceived differences between the poorest and non-poorest ($p < 0.05$) were thought to occur more often in the poorest (104 of 113), although ischemic heart disease, type 2 diabetes, eating disorders, gout, and several substance use disorders were thought to occur more often among the non-poorest. Of the 164 conditions we included that can be fatal according to GBD, respondents perceived 153 to be more highly fatal among the poorest than the less poor; the others showed no difference.

Respondents generally expressed confidence in their answers. Over 80% of experts were confident or very confident of their answers about incidence for 71 conditions, while there were only six conditions for which over 50% of respondents were uncertain or very uncertain in their responses about incidence. The spread of responses showed a variety of certainty levels in the responses in aggregate. For incidence, there were 22 conditions (10%) for which respondents answered using only two categories, showing relative certainty, and 27 conditions (13%) for which respondents' answers spanned all five options from "Much Higher in the Non-Poorest" to "Much Higher in the Poorest," showing a higher degree of uncertainty across experts.

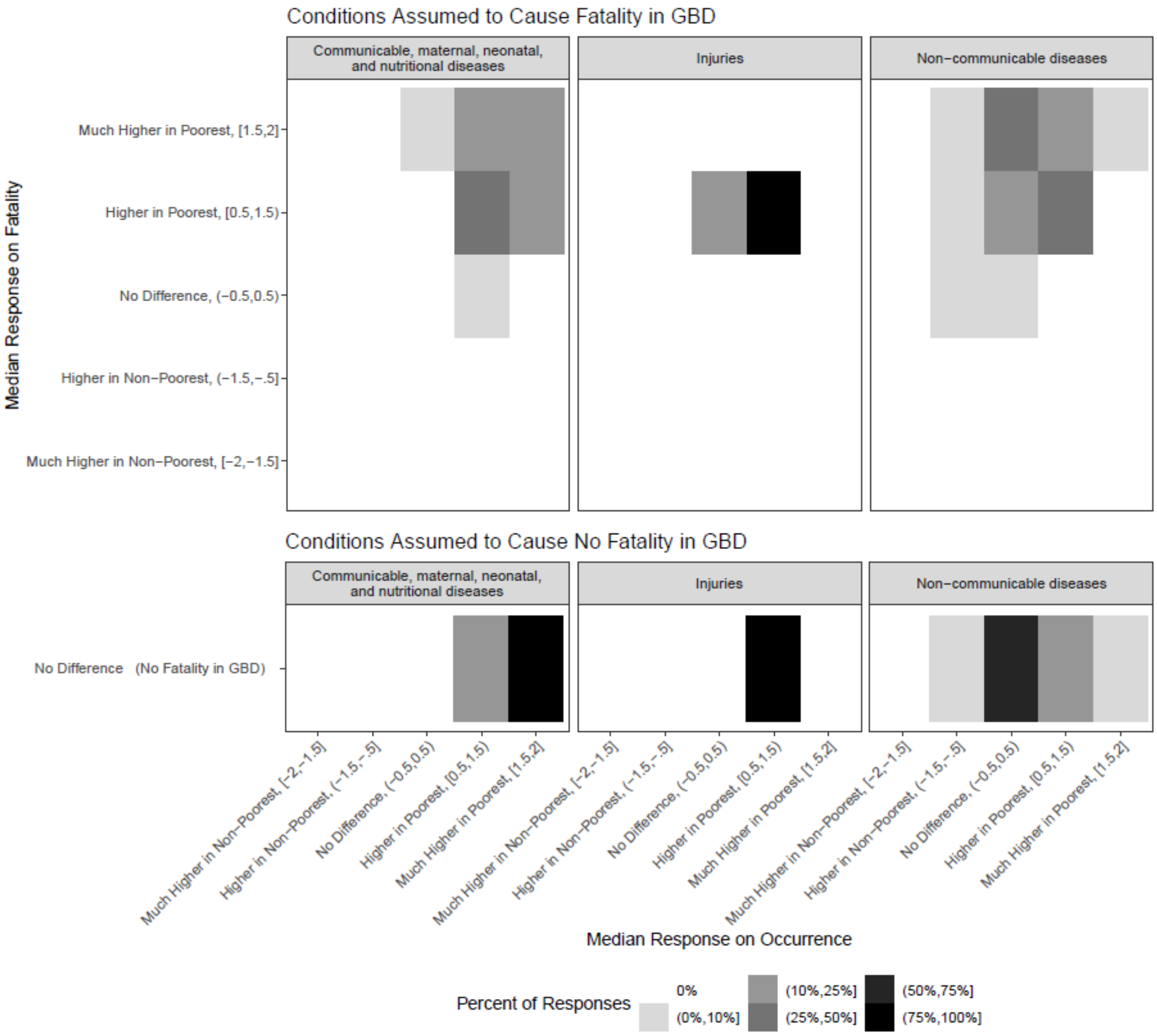

To plot spread, the axes are converted to a continuous -2 (Much Higher in the Non-Poorest) to 2 (Much Higher in the Poorest) scale to calculate medians. Percentages are shown within disease categories.

Appendix Figure 2: Summary of expert perceptions of disease incidence and case fatality among the poorest billion compared to non-poorest in low- and middle-income countries, by disease groups

For more disease-specific results, see table below or appendix in:

Bukhman G, Mocumbi AO, Atun R, Becker AE, Bhutta Z, Binagwaho A, et al. The Lancet NCDI Poverty Commission: bridging a gap in universal health coverage for the poorest billion. The Lancet. 2020;396: 991–1044. doi:10.1016/S0140-6736(20)31907-3

Appendix Table 6. Summary results from expert survey on perceptions of disease occurrence and fatality

-2 = Much Higher in the Non-Poorest, -1 = Higher in the Non-Poorest, 0 = No Difference, 1 = Higher in the Poorest, 2 = Much Higher in the Poorest

P-values based on sign test for the median being different than "No Difference between Poorest and Non-Poorest" (0)

Condition	Measure	Median	Min	Q1	Q3	Max	p-value	Number of Respondents
**Communicable, maternal, neonatal, and nutritional diseases**								
Acute hepatitis A	fatality	1	0	1	1	2	<0.001	33
Acute hepatitis A	occurrence	1	-1	1	2	2	<0.001	33
Acute hepatitis E	fatality	1	0	1	2	2	<0.001	19
Acute hepatitis E	occurrence	1	0	1	2	2	<0.001	20
African trypanosomiasis	fatality	2	1	1	2	2	<0.001	19
African trypanosomiasis	occurrence	2	0	1	2	2	<0.001	20
Ascariasis	fatality	1	0	0	2	2	0.001	19
Ascariasis	occurrence	2	1	1	2	2	<0.001	20
Chagas disease	fatality	1	0	1	2	2	<0.001	19
Chagas disease	occurrence	1	-2	1	2	2	0.001	20
Chlamydial infection	fatality	0.5	0	0	1	2	<0.001	42
Chlamydial infection	occurrence	1	-1	1	1	2	<0.001	42
Cutaneous and mucocutaneous leishmaniasis	occurrence	2	1	1	2	2	<0.001	19
Cystic echinococcosis	fatality	1	0	1	1.5	2	<0.001	19
Cystic echinococcosis	occurrence	1	0	1	2	2	<0.001	20
Cysticercosis	fatality	1	0	1	2	2	<0.001	20
Cysticercosis	occurrence	1	-2	1	2	2	<0.001	21
Dengue	fatality	1	0	1	2	2	<0.001	19
Dengue	occurrence	1	-1	1	2	2	<0.001	20
Diarrheal diseases	fatality	2	1	1.5	2	2	<0.001	19
Diarrheal diseases	occurrence	2	1	2	2	2	<0.001	20
Diphtheria	fatality	2	0	1	2	2	<0.001	19
Diphtheria	occurrence	2	0	1	2	2	<0.001	20
Ebola	fatality	1.5	0	1	2	2	<0.001	20
Ebola	occurrence	2	0	1	2	2	<0.001	21
Encephalitis	fatality	1	0	1	2	2	<0.001	19
Encephalitis	occurrence	1	0	1	1.25	2	<0.001	20
Food-borne trematodiases	occurrence	2	1	1	2	2	<0.001	20
Genital herpes	fatality	0	0	0	1	2	<0.001	42
Genital herpes	occurrence	1	-1	0	1	2	<0.001	43
Gonococcal infection	fatality	1	0	0	1.75	2	<0.001	42
Gonococcal infection	occurrence	1	-1	1	1	2	<0.001	43
Hemolytic disease and other neonatal jaundice	fatality	2	0	1	2	2	0.001	15
Hemolytic disease and other neonatal jaundice	occurrence	0	0	0	1	2	0.015	15

Hepatitis B	fatality	1	0	1	2	2	<0.001	19
Hepatitis B	occurrence	1	-1	0.5	1	2	0.003	19
Hepatitis C	fatality	1	0	1	2	2	<0.001	19
Hepatitis C	occurrence	1	-1	0	1	2	0.003	20
HIV/AIDS - Tuberculosis	fatality	2	0	1	2	2	<0.001	33
HIV/AIDS - Tuberculosis	occurrence	1.5	0	1	2	2	<0.001	34
HIV/AIDS resulting in other diseases	fatality	2	-1	1	2	2	<0.001	33
HIV/AIDS resulting in other diseases	occurrence	1	-2	1	2	2	<0.001	34
Hookworm disease	occurrence	2	1	1	2	2	<0.001	20
Indirect maternal deaths	occurrence	2	0	1	2	2	0.001	15
Iodine deficiency	fatality	1	0	1	2	2	0.007	11
Iodine deficiency	occurrence	2	0	1	2	2	0.005	11
Iron-deficiency anemia	fatality	2	1	1	2	2	0.003	11
Iron-deficiency anemia	occurrence	2	1	1.5	2	2	0.002	11
Late maternal deaths	occurrence	2	0	1	2	2	<0.001	15
Leprosy	occurrence	2	0	1	2	2	<0.001	22
Lower respiratory infections	fatality	2	0	1	2	2	<0.001	19
Lower respiratory infections	occurrence	1	-1	1	2	2	<0.001	20
Lymphatic filariasis	occurrence	2	-1	1	2	2	<0.001	20
Malaria	fatality	2	1	1	2	2	<0.001	21
Malaria	occurrence	1	1	1	2	2	<0.001	22
Maternal abortion, miscarriage, and ectopic pregnancy	fatality	2	1	1	2	2	0.003	11
Maternal abortion, miscarriage, and ectopic pregnancy	occurrence	1	0	0.5	1	2	0.008	11
Maternal deaths aggravated by HIV/AIDS	occurrence	1	-1	1	2	2	<0.001	17
Maternal hemorrhage	fatality	2	1	1	2	2	0.002	12
Maternal hemorrhage	occurrence	1	0	1	1	2	0.003	12
Maternal hypertensive disorders	fatality	1	1	1	2	2	0.002	12
Maternal hypertensive disorders	occurrence	1	-1	0	1	1	0.023	12
Maternal obstructed labor and uterine rupture	fatality	2	1	1.5	2	2	0.002	11
Maternal obstructed labor and uterine rupture	occurrence	2	1	1	2	2	0.003	11
Maternal sepsis and other maternal infections	fatality	2	1	1	2	2	0.002	12
Maternal sepsis and other maternal infections	occurrence	1	1	1	2	2	0.002	12
Measles	fatality	1.5	1	1	2	2	<0.001	20
Measles	occurrence	2	0	1	2	2	<0.001	21
Meningitis	fatality	1.5	1	1	2	2	<0.001	20
Meningitis	occurrence	1	0	1	2	2	<0.001	21
Neonatal encephalopathy due to birth asphyxia and trauma	fatality	2	0	2	2	2	<0.001	17
Neonatal encephalopathy due to birth asphyxia and trauma	occurrence	1	-1	1	2	2	0.001	17
Neonatal preterm birth complications	fatality	2	1	1.75	2	2	<0.001	16
Neonatal preterm birth complications	occurrence	1	-1	1	2	2	0.001	16

Neonatal sepsis and other neonatal infections	fatality	2	1	1	2	2	<0.001	16
Neonatal sepsis and other neonatal infections	occurrence	1	-1	1	2	2	<0.001	16
Onchocerciasis	occurrence	2	0	1	2	2	<0.001	21
Otitis media	fatality	1	0	0	1.5	2	0.002	19
Otitis media	occurrence	1	0	1	2	2	<0.001	20
Paratyphoid fever	fatality	1	0	1	2	2	<0.001	19
Paratyphoid fever	occurrence	1	0	1	2	2	<0.001	19
Protein-energy malnutrition	fatality	2	1	2	2	2	<0.001	15
Protein-energy malnutrition	occurrence	2	1	2	2	2	<0.001	15
Rabies	fatality	1	0	0.5	2	2	<0.001	19
Rabies	occurrence	1	0	1	2	2	<0.001	20
Schistosomiasis	fatality	1	0	1	2	2	<0.001	20
Schistosomiasis	occurrence	2	1	1	2	2	<0.001	21
Syphilis	fatality	1	0	1	2	2	<0.001	42
Syphilis	occurrence	1	-2	1	2	2	<0.001	43
Tetanus	fatality	1	0	1	2	2	<0.001	19
Tetanus	occurrence	2	1	1	2	2	<0.001	20
Trachoma	occurrence	2	0	2	2	2	<0.001	21
Trichomoniasis	occurrence	1	-2	1	1	2	<0.001	43
Trichuriasis	occurrence	2	0	1	2	2	<0.001	19
Tuberculosis	fatality	2	1	1.75	2	2	<0.001	40
Tuberculosis	occurrence	2	1	1.75	2	2	<0.001	40
Typhoid fever	fatality	2	0	1	2	2	<0.001	19
Typhoid fever	occurrence	2	0	1	2	2	<0.001	20
Upper respiratory infections	fatality	1	0	1	2	2	<0.001	19
Upper respiratory infections	occurrence	1	0	0	1.25	2	0.002	20
Varicella and herpes zoster	fatality	1	0	1	1	2	<0.001	19
Varicella and herpes zoster	occurrence	1	0	0.75	2	2	<0.001	20
Visceral leishmaniasis	fatality	1	0	1	2	2	<0.001	18
Visceral leishmaniasis	occurrence	2	0	1	2	2	<0.001	19
Vitamin A deficiency	occurrence	2	1	2	2	2	<0.001	14
Whooping cough	fatality	1	0	1	1.5	2	<0.001	19
Whooping cough	occurrence	1	0	1	2	2	<0.001	20
Yellow fever	fatality	1	0	1	2	2	<0.001	18
Yellow fever	occurrence	1	-1	1	2	2	<0.001	20
**Injuries**								
Adverse effects of medical treatment	fatality	1	-1	1	2	2	0.002	18
Adverse effects of medical treatment	occurrence	1	-1	-0.25	1	2	0.078	20
Assault by firearm	fatality	1	-1	1	2	2	<0.001	19
Assault by firearm	occurrence	0.5	-1	-0.25	1	2	0.132	20
Assault by other means	fatality	1	-1	1	2	2	<0.001	19
Assault by other means	occurrence	1	-1	0	1	2	0.005	20

Assault by sharp object	fatality	1	-1	1	2	2	<0.001	19
Assault by sharp object	occurrence	1	-1	0	1	2	0.003	20
Collective violence and legal intervention	fatality	1	-1	1	1.75	2	<0.001	18
Collective violence and legal intervention	occurrence	1	-1	1	2	2	<0.001	19
Drowning	fatality	1	0	1	1	2	<0.001	18
Drowning	occurrence	1	0	1	2	2	<0.001	20
Environmental heat and cold exposure	fatality	1	-1	1	1.75	2	<0.001	18
Environmental heat and cold exposure	occurrence	1	0	1	2	2	<0.001	20
Exposure to forces of nature	fatality	1	-1	1	2	2	<0.001	18
Exposure to forces of nature	occurrence	1	-1	0.75	2	2	<0.001	20
Falls	fatality	1	-1	0.25	1.75	2	0.002	18
Falls	occurrence	1	0	0	1	2	0.002	20
Fire, heat, and hot substances	fatality	1	-1	1	2	2	<0.001	18
Fire, heat, and hot substances	occurrence	1	-2	1	2	2	0.001	20
Foreign body in eyes	occurrence	1	0	0	1	2	0.002	20
Non-venomous animal contact	fatality	1	-1	0	2	2	0.003	17
Non-venomous animal contact	occurrence	1	-1	1	1	2	<0.001	19
Poisonings	fatality	1	-1	1	2	2	<0.001	18
Poisonings	occurrence	1	0	0	1.25	2	<0.001	20
Pulmonary aspiration and foreign body in airway	fatality	1	-1	1	1	2	0.001	17
Pulmonary aspiration and foreign body in airway	occurrence	0	0	0	0.5	2	0.053	19
Road injuries	fatality	1	-2	1	2	2	0.005	19
Road injuries	occurrence	1	-2	-1	1	2	0.253	21
Self-harm	fatality	1	-1	0	1	2	0.009	17
Self-harm	occurrence	0	-2	-1	0	1	0.308	19
Unintentional firearm injuries	fatality	1	-1	1	2	2	0.001	17
Unintentional firearm injuries	occurrence	1	-2	-1	1	2	0.607	20
Unintentional suffocation	fatality	1	-1	0	1	2	0.004	17
Unintentional suffocation	occurrence	1	-1	0	1	2	0.023	19
Venomous animal contact	fatality	1	0	1	2	2	0.001	16
Venomous animal contact	occurrence	1	0	0.5	1	2	<0.001	19
**Non-communicable diseases**								
Acne vulgaris	occurrence	0	-1	0	0	1	1	8
Acute glomerulonephritis	fatality	1	0	1	1.5	2	0.001	15
Acute glomerulonephritis	occurrence	0	-1	0	1	2	0.071	15
Acute lymphoid leukemia	fatality	2	0	1	2	2	<0.001	24
Acute lymphoid leukemia	occurrence	0	-1	0	0	2	1	25
Acute myeloid leukemia	fatality	2	0	1	2	2	<0.001	24
Acute myeloid leukemia	occurrence	0	-1	-0.25	0	2	0.608	24
Alcohol use disorders	fatality	1	-1	0	1	2	0.012	17
Alcohol use disorders	occurrence	1	-2	0	1	2	0.158	17

Alopecia areata	occurrence	0	-1	-0.5	0.5	1	1	7
Alzheimer disease and other dementias	fatality	1	-1	1	1	2	0.015	10
Alzheimer disease and other dementias	occurrence	-0.5	-2	-1	0	1	0.12	10
Amphetamine use disorders	fatality	0	-1	0	1	2	0.158	14
Amphetamine use disorders	occurrence	-1	-2	-1	-1	1	0.008	15
Anorexia nervosa	fatality	0	-1	-0.75	0.75	2	0.821	14
Anorexia nervosa	occurrence	-1	-2	-2	-1	0	0.002	14
Anxiety disorders	occurrence	0	-2	-1.25	1	2	0.774	16
Aortic aneurysm	occurrence	0	-2	-1	0.75	1	0.419	30
Appendicitis	fatality	2	1	1	2	2	0.012	8
Appendicitis	occurrence	0	0	0	0	2	1	9
Asbestosis	fatality	1	0	1	2	2	0.001	16
Asbestosis	occurrence	1	-2	0	1	2	0.071	16
Asperger syndrome and other autistic spectrum disorders	occurrence	0	-1	0	0	2	0.824	13
Asthma	fatality	1	0	1	2	2	<0.001	18
Asthma	occurrence	0.5	-2	0	1	1	0.212	18
Atrial fibrillation and flutter	fatality	2	0	1	2	2	<0.001	29
Atrial fibrillation and flutter	occurrence	0	-2	-1	1	2	0.712	29
Attention-deficit/hyperactivity disorder	occurrence	0	-2	-1	0	1	0.152	14
Autism	occurrence	0	-2	0	0	2	1	14
Benign prostatic hyperplasia	occurrence	0	-1	0	0	1	0.773	11
Bipolar disorder	occurrence	0	-1	0	0.75	2	0.374	14
Bladder cancer	fatality	2	0	1	2	2	<0.001	21
Bladder cancer	occurrence	0	-1	0	1	1	0.039	22
Brain and nervous system cancer	fatality	2	-1	1	2	2	<0.001	26
Brain and nervous system cancer	occurrence	0	-2	0	0	1	0.374	27
Breast cancer	fatality	2	1	1	2	2	<0.001	20
Breast cancer	occurrence	0	-1	-1	0	1	0.802	21
Bulimia nervosa	fatality	0	-1	-1	0	2	0.821	14
Bulimia nervosa	occurrence	-1	-2	-2	-1	0	0.002	14
Cannabis use disorders	occurrence	-1	-2	-1	0.75	2	0.549	18
Cardiomyopathy and myocarditis	fatality	2	1	1	2	2	<0.001	28
Cardiomyopathy and myocarditis	occurrence	1	-1	0	1	2	0.003	28
Cataract	occurrence	1.5	0	1	2	2	0.053	6
Cellulitis	fatality	1	0	1	1	2	0.026	7
Cellulitis	occurrence	1	0	0	1	1	0.072	7
Cervical cancer	fatality	2	1	1	2	2	<0.001	24
Cervical cancer	occurrence	1	0	1	2	2	<0.001	25
Chronic kidney disease due to diabetes mellitus	fatality	1	1	1	2	2	<0.001	16
Chronic kidney disease due to diabetes mellitus	occurrence	1	-1	1	1.25	2	0.005	16
Chronic kidney disease due to glomerulonephritis	fatality	1	0	1	2	2	0.001	15

Chronic kidney disease due to glomerulonephritis	occurrence	1	0	0	1	1	0.002	15
Chronic kidney disease due to hypertension	fatality	1	0	1	2	2	<0.001	17
Chronic kidney disease due to hypertension	occurrence	1	-1	1	1	2	0.003	17
Chronic kidney disease due to other causes	fatality	1	0	1	1.25	2	0.001	16
Chronic kidney disease due to other causes	occurrence	0.5	-1	0	1	2	0.025	16
Chronic lymphoid leukemia	fatality	1.5	0	1	2	2	<0.001	24
Chronic lymphoid leukemia	occurrence	0	-1	-1	0	1	0.066	25
Chronic myeloid leukemia	fatality	1.5	0	1	2	2	<0.001	24
Chronic myeloid leukemia	occurrence	0	-1	-1	0	2	0.308	25
Chronic obstructive pulmonary disease	fatality	1	0	1	2	2	<0.001	16
Chronic obstructive pulmonary disease	occurrence	1	-2	0	1	2	0.061	16
Cirrhosis and other chronic liver diseases due to alcohol use	fatality	1.5	1	1	2	2	0.005	10
Cirrhosis and other chronic liver diseases due to alcohol use	occurrence	1	0	0.25	1	2	0.018	10
Cirrhosis and other chronic liver diseases due to hepatitis B	fatality	2	1	1	2	2	0.007	9
Cirrhosis and other chronic liver diseases due to hepatitis B	occurrence	1	0	1	2	2	0.012	9
Cirrhosis and other chronic liver diseases due to hepatitis C	fatality	1.5	0	1	2	2	0.007	10
Cirrhosis and other chronic liver diseases due to hepatitis C	occurrence	1	0	0.25	1.75	2	0.019	10
Cirrhosis and other chronic liver diseases due to other causes	fatality	1	0	1	2	2	0.012	9
Cirrhosis and other chronic liver diseases due to other causes	occurrence	0	-1	0	1	2	0.572	9
Cleft lip and cleft palate	fatality	1	1	1	1.5	2	0.018	7
Cleft lip and cleft palate	occurrence	1	0	0	1	2	0.089	7
Coal workers pneumoconiosis	fatality	1	1	1	2	2	<0.001	15
Coal workers pneumoconiosis	occurrence	1	0	1	1.5	2	0.001	15
Cocaine use disorders	fatality	0	-1	-0.75	1	2	0.437	14
Cocaine use disorders	occurrence	-1	-2	-1	1	1	0.143	14
Colon and rectum cancer	fatality	2	1	1	2	2	<0.001	26
Colon and rectum cancer	occurrence	0	-2	-1	0	1	0.065	27
Conduct disorder	occurrence	0	-1	0	1	2	0.124	14
Congenital heart anomalies	fatality	2	1	2	2	2	<0.001	27
Congenital heart anomalies	occurrence	0	-1	0	0.5	2	0.042	27
Deciduous caries	occurrence	1	-2	0	1	2	0.71	5
Decubitus ulcer	fatality	1	1	1	1.5	2	0.018	7
Decubitus ulcer	occurrence	1	-2	0.5	1	2	0.33	7
Dermatitis	occurrence	1	-1	0	1	2	0.24	7
Down syndrome	fatality	2	0	2	2	2	0.008	9
Down syndrome	occurrence	0	-1	0	0.75	1	0.766	10
Dysthymia	occurrence	0	-1	0	1	2	0.105	13
Edentulism and severe tooth loss	occurrence	2	1	2	2	2	0.048	5
Endocarditis	fatality	2	1	1	2	2	<0.001	29

Endocarditis	occurrence	1	-2	0	1	2	<0.001	29
Endometriosis	fatality	0	0	0	0.25	1	0.346	8
Endometriosis	occurrence	0	-1	0	0	0	1	8
Epilepsy	fatality	1	1	1	2	2	0.007	9
Epilepsy	occurrence	1	1	1	1	2	0.005	9
Esophageal cancer	fatality	2	0	1	2	2	<0.001	26
Esophageal cancer	occurrence	1	-1	0	1	2	0.001	27
Female infertility	occurrence	1	-1	0	1	1	0.299	9
Fungal skin diseases	occurrence	1	1	1	1.5	2	0.018	7
G6PD deficiency	fatality	1	0	0.75	1.25	2	0.031	8
G6PD deficiency	occurrence	0	-1	0	1	2	0.345	8
G6PD trait	occurrence	0	-2	-0.25	0.25	2	1	8
Gallbladder and biliary diseases	fatality	1	0	1	1	1	0.006	9
Gallbladder and biliary diseases	occurrence	0	0	0	0	1	1	9
Gallbladder and biliary tract cancer	fatality	2	0	1	2	2	<0.001	26
Gallbladder and biliary tract cancer	occurrence	0	-1	-1	0	1	0.023	27
Gastritis and duodenitis	fatality	1	0	1	1	2	0.008	10
Gastritis and duodenitis	occurrence	0.5	-1	0	1	2	0.12	10
Genital prolapse	fatality	0.5	0	0	1	2	0.089	8
Genital prolapse	occurrence	1	1	1	1	1	0.006	8
Glaucoma	occurrence	0.5	0	0	1.75	2	0.174	6
Gout	occurrence	-1	-2	-2	0	0	0.007	14
Hemorrhagic stroke	fatality	2	-1	1	2	2	<0.001	31
Hemorrhagic stroke	occurrence	1	-2	-0.5	1	2	0.039	31
Hodgkin lymphoma	fatality	1.5	0	1	2	2	<0.001	24
Hodgkin lymphoma	occurrence	0	-1	0	0.75	2	0.308	26
Hypertensive heart disease	fatality	2	-2	1	2	2	<0.001	30
Hypertensive heart disease	occurrence	0	-2	-1	1	2	0.438	30
Idiopathic developmental intellectual disability	occurrence	0	-1	0	1	1	0.073	13
Inflammatory bowel disease	fatality	1	1	1	1	2	0.003	10
Inflammatory bowel disease	occurrence	-0.5	-1	-1	0	2	0.374	10
Inguinal, femoral, and abdominal hernia	fatality	1	0	0.75	2	2	0.032	8
Inguinal, femoral, and abdominal hernia	occurrence	1	0	0	1	1	0.02	9
Interstitial nephritis and urinary tract infections	fatality	1	0	1	2	2	0.001	15
Interstitial nephritis and urinary tract infections	occurrence	1	0	0	1	2	0.004	16
Ischemic heart disease	fatality	2	1	1	2	2	<0.001	29
Ischemic heart disease	occurrence	-1	-2	-1	1	2	0.05	29
Ischemic stroke	fatality	2	-1	1	2	2	<0.001	31
Ischemic stroke	occurrence	0	-2	-1	1	2	0.729	31
Kidney cancer	fatality	1	-1	1	2	2	<0.001	28
Kidney cancer	occurrence	0	-1	0	0	1	0.299	29

Klinefelter syndrome	occurrence	0	-1	0	0	0	1	9
Larynx cancer	fatality	1.5	-1	1	2	2	<0.001	30
Larynx cancer	occurrence	0	-1	0	1	2	0.188	32
Lip and oral cavity cancer	fatality	1	-1	1	2	2	<0.001	24
Lip and oral cavity cancer	occurrence	1	-1	0	1	1	0.063	25
Liver cancer due to alcohol use	fatality	1	0	1	2	2	<0.001	26
Liver cancer due to alcohol use	occurrence	1	-2	0	1	2	0.046	27
Liver cancer due to hepatitis B	fatality	2	0	1	2	2	<0.001	26
Liver cancer due to hepatitis B	occurrence	1	0	1	1.5	2	<0.001	27
Liver cancer due to hepatitis C	fatality	1.5	0	1	2	2	<0.001	26
Liver cancer due to hepatitis C	occurrence	1	0	1	1	2	<0.001	27
Liver cancer due to other causes	fatality	1	0	1	2	2	<0.001	26
Liver cancer due to other causes	occurrence	0	-1	0	1	2	0.05	27
Low back pain	occurrence	0	-2	-1	1	1	0.356	16
Macular degeneration	occurrence	0	-1	0	0.75	2	0.586	6
Major depressive disorder	occurrence	0	-1	-1	1	2	0.83	16
Male infertility	occurrence	0	-1	0	0	1	1	7
Malignant skin melanoma	fatality	1	-1	1	2	2	<0.001	23
Malignant skin melanoma	occurrence	0	-2	-1	1	2	0.562	24
Medication overuse headache	occurrence	-1	-1	-1	-0.75	0	0.02	8
Mesothelioma	fatality	2	-1	1	2	2	<0.001	29
Mesothelioma	occurrence	0	-1	0	1	1	0.301	29
Migraine	occurrence	0	-1	-1	0	1	0.424	9
Motor neuron disease	fatality	1	-1	0.75	1.25	2	0.065	8
Motor neuron disease	occurrence	0	-1	-1	0	0	0.149	8
Multiple myeloma	fatality	1.5	0	1	2	2	<0.001	24
Multiple myeloma	occurrence	0	-1	0	0	1	0.565	25
Multiple sclerosis	fatality	1	-1	0.75	1.25	2	0.065	8
Multiple sclerosis	occurrence	-0.5	-1	-1	0	0	0.072	8
Nasopharynx cancer	fatality	1	-1	1	2	2	<0.001	24
Nasopharynx cancer	occurrence	0	-1	0	1	2	0.308	25
Neck pain	occurrence	0	-2	-1	0	1	0.275	14
Neural tube defects	fatality	1	1	1	1.75	2	0.031	6
Neural tube defects	occurrence	1	1	1	1.75	2	0.031	6
Non-Hodgkin lymphoma	fatality	2	1	1	2	2	<0.001	24
Non-Hodgkin lymphoma	occurrence	0	-1	0	1	2	0.042	24
Non-melanoma skin cancer (basal-cell carcinoma)	fatality	1	-1	1	2	2	<0.001	23
Non-melanoma skin cancer (basal-cell carcinoma)	occurrence	0	-2	-1	0	1	0.212	24
Non-melanoma skin cancer (squamous-cell carcinoma)	fatality	1	0	1	2	2	<0.001	22
Non-melanoma skin cancer (squamous-cell carcinoma)	occurrence	0	-2	-1	1	1	0.66	24
Opioid use disorders	fatality	0	-1	-0.75	1	1	0.565	14
Opioid use disorders	occurrence	-1	-2	-2	-1	1	0.011	15

Osteoarthritis	occurrence	0	-2	-1	0	1	0.158	14
Other pneumoconiosis	fatality	1	1	1	2	2	<0.001	15
Other pneumoconiosis	occurrence	1	-1	0.5	1	2	0.006	15
Ovarian cancer	fatality	2	0	1	2	2	<0.001	22
Ovarian cancer	occurrence	0	-1	0	0.5	1	0.182	23
Pancreatic cancer	fatality	1.5	0	1	2	2	<0.001	26
Pancreatic cancer	occurrence	0	-1	-0.5	0	1	0.11	27
Pancreatitis	fatality	1	0	1	1	1	0.006	9
Pancreatitis	occurrence	0	-1	0	0	1	0.773	9
Paralytic ileus and intestinal obstruction	fatality	1.5	1	1	2	2	0.012	8
Paralytic ileus and intestinal obstruction	occurrence	1	-1	0	1	2	0.12	9
Parkinson disease	fatality	1	0	1	1	2	0.01	9
Parkinson disease	occurrence	0	-1	-1	0	1	0.424	9
Peptic ulcer disease	fatality	1	0	0.75	2	2	0.032	8
Peptic ulcer disease	occurrence	1	-1	0	1	2	0.105	9
Periodontal diseases	occurrence	1	-1	1	1	2	0.203	5
Peripheral vascular disease	fatality	2	1	1	2	2	<0.001	28
Peripheral vascular disease	occurrence	0	-2	-1	1	2	0.475	28
Permanent caries	occurrence	1	-1	-1	2	2	0.408	5
Polycystic ovarian syndrome	fatality	0	0	0	0	0	NaN	9
Polycystic ovarian syndrome	occurrence	0	-1	-1	0	0	0.072	9
Premenstrual syndrome	occurrence	0	-1	-0.25	0	1	0.773	8
Prostate cancer	fatality	1	0	1	2	2	<0.001	22
Prostate cancer	occurrence	0	-1	0	0	1	0.777	24
Pruritus	occurrence	1	0	0	1	2	0.089	7
Psoriasis	occurrence	0	-2	-0.5	1	1	1	7
Pyoderma	fatality	1	1	1	1	2	0.015	7
Pyoderma	occurrence	1	0	0.5	1.5	2	0.053	7
Refraction and accommodation disorders	occurrence	0	-1	0	0.75	1	0.773	6
Rheumatic heart disease	fatality	2	1	2	2	2	<0.001	27
Rheumatic heart disease	occurrence	2	0	2	2	2	<0.001	26
Rheumatoid arthritis	fatality	1	0	1	1	2	0.003	13
Rheumatoid arthritis	occurrence	0	-1	0	0	1	1	14
Scabies	occurrence	2	0	1	2	2	0.018	8
Schizophrenia	fatality	1	0	1	2	2	0.003	14
Schizophrenia	occurrence	0	-1	0	1	2	0.24	15
Sickle cell disorders	fatality	2	0	1	2	2	0.018	9
Sickle cell disorders	occurrence	0	0	0	1	2	0.089	9
Sickle cell trait	occurrence	0	0	0	1	2	0.174	9
Silicosis	fatality	1	0	1	2	2	0.001	16
Silicosis	occurrence	1	-2	0	1	2	0.056	16
Stomach cancer	fatality	1	0	1	2	2	<0.001	27

Stomach cancer	occurrence	0	-1	0	1	1	0.208	28
Sudden infant death syndrome	occurrence	0	-1	0	1	2	0.124	16
Tension-type headache	occurrence	0	-2	-1	1	1	0.824	8
Testicular cancer	fatality	1	0	1	2	2	<0.001	22
Testicular cancer	occurrence	0	-1	0	0	1	0.773	23
Thalassemias	fatality	1	0	0.75	2	2	0.032	8
Thalassemias	occurrence	0	0	0	0.25	2	0.371	8
Thalassemias trait	occurrence	0	-1	0	1	2	0.345	8
Thyroid cancer	fatality	1	-1	1	2	2	<0.001	21
Thyroid cancer	occurrence	0	-1	-0.75	0	2	0.824	22
Tracheal, bronchus, and lung cancer	fatality	2	-1	1	2	2	<0.001	29
Tracheal, bronchus, and lung cancer	occurrence	0	-2	0	1	1	0.209	30
Turner syndrome	occurrence	0	-1	0	0	0	0.346	9
Type 1 diabetes mellitus	fatality	2	0	1	2	2	<0.001	33
Type 1 diabetes mellitus	occurrence	0	-2	0	0	2	1	34
Type 2 diabetes mellitus	fatality	2	0	1	2	2	<0.001	32
Type 2 diabetes mellitus	occurrence	-1	-2	-1	0.75	1	0.009	34
Urolithiasis	fatality	1	0	0	1	2	0.174	5
Urolithiasis	occurrence	0.5	-1	-0.75	1	1	0.766	6
Urticaria	occurrence	0	0	0	1	1	0.149	8
Uterine cancer	fatality	2	0	1	2	2	<0.001	22
Uterine cancer	occurrence	0	-1	0	0.5	1	0.802	23
Uterine fibroids	fatality	1	0	0	1	2	0.026	9
Uterine fibroids	occurrence	0	-1	0	0	1	0.773	9
Vascular intestinal disorders	fatality	1	0	0	1	1	0.037	8
Vascular intestinal disorders	occurrence	0	-1	-1	0	0	0.072	9
Viral skin diseases	occurrence	1	-1	0	1	1	0.233	7

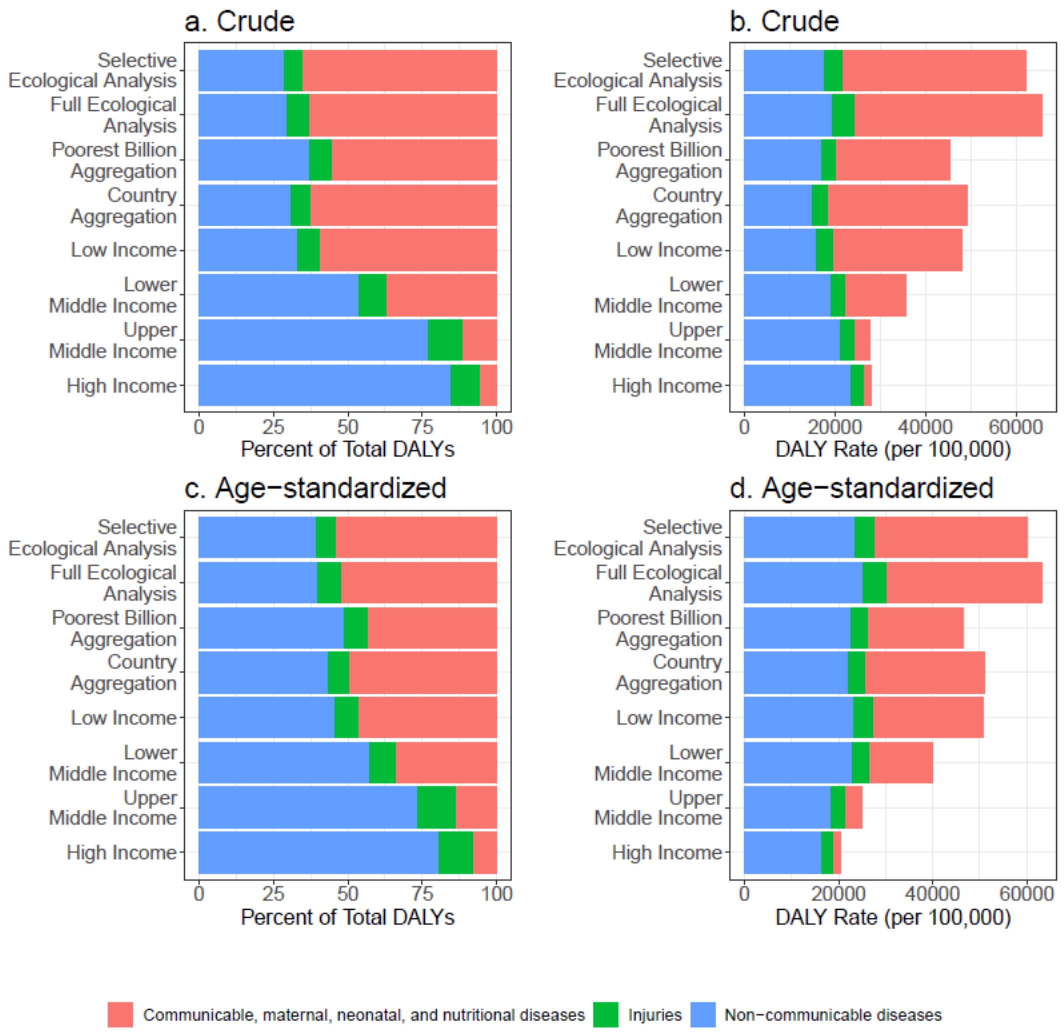

Appendix Figure 3: Proportion of DALYs by broad cause groups, crude (a) and age-standardized (b); Rates of DALYs by broad cause groups, crude (c) and age-standardized (d), for five approaches to estimating the burden in the poorest billion and World Bank income groups

Appendix Figure 4a. Cause−specific DALY Rates by Age, Full Ecological Approach

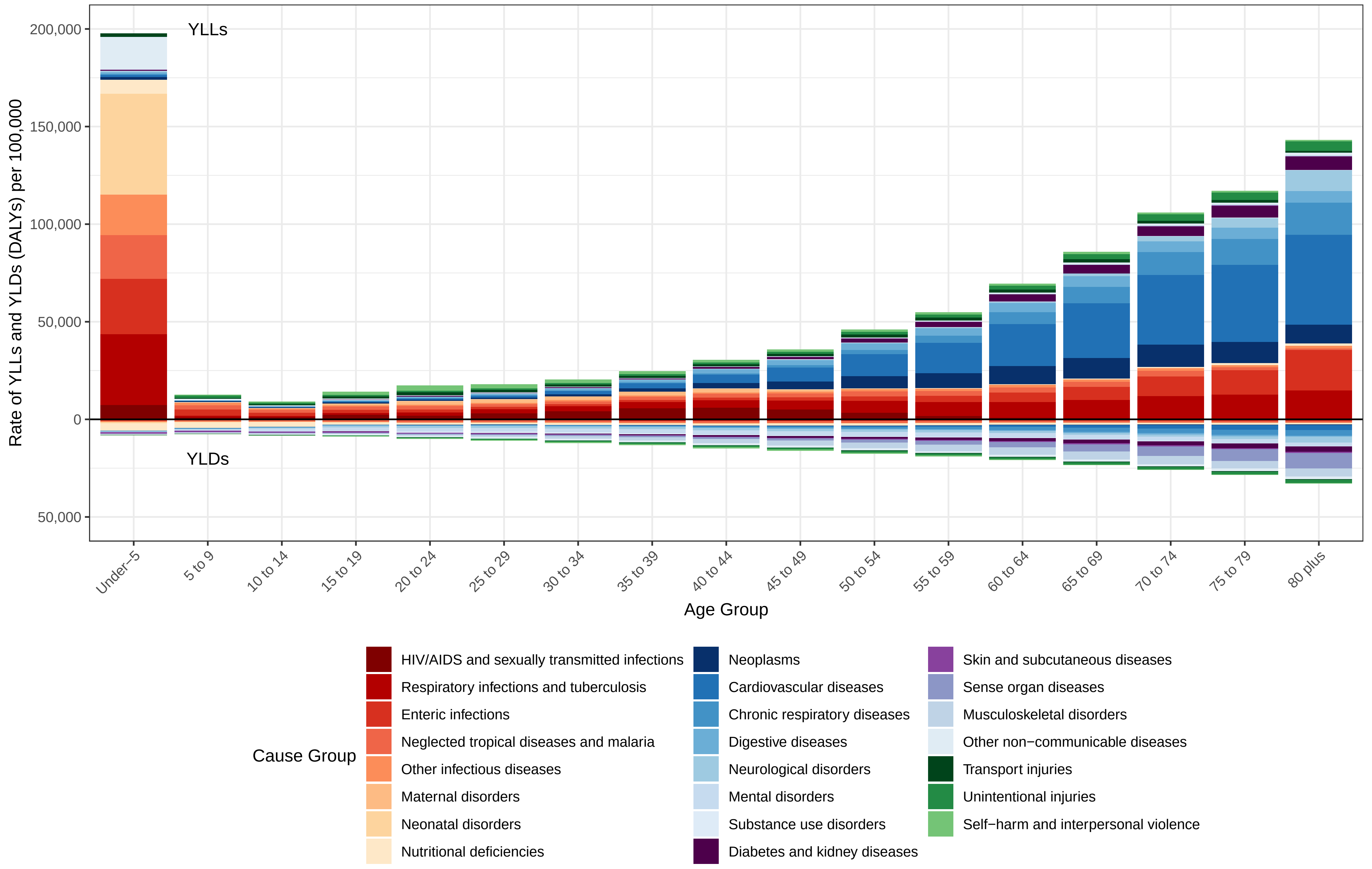

Appendix Figure 4b. Cause−specific DALY Rates by Age, Poorest Billion Aggregation

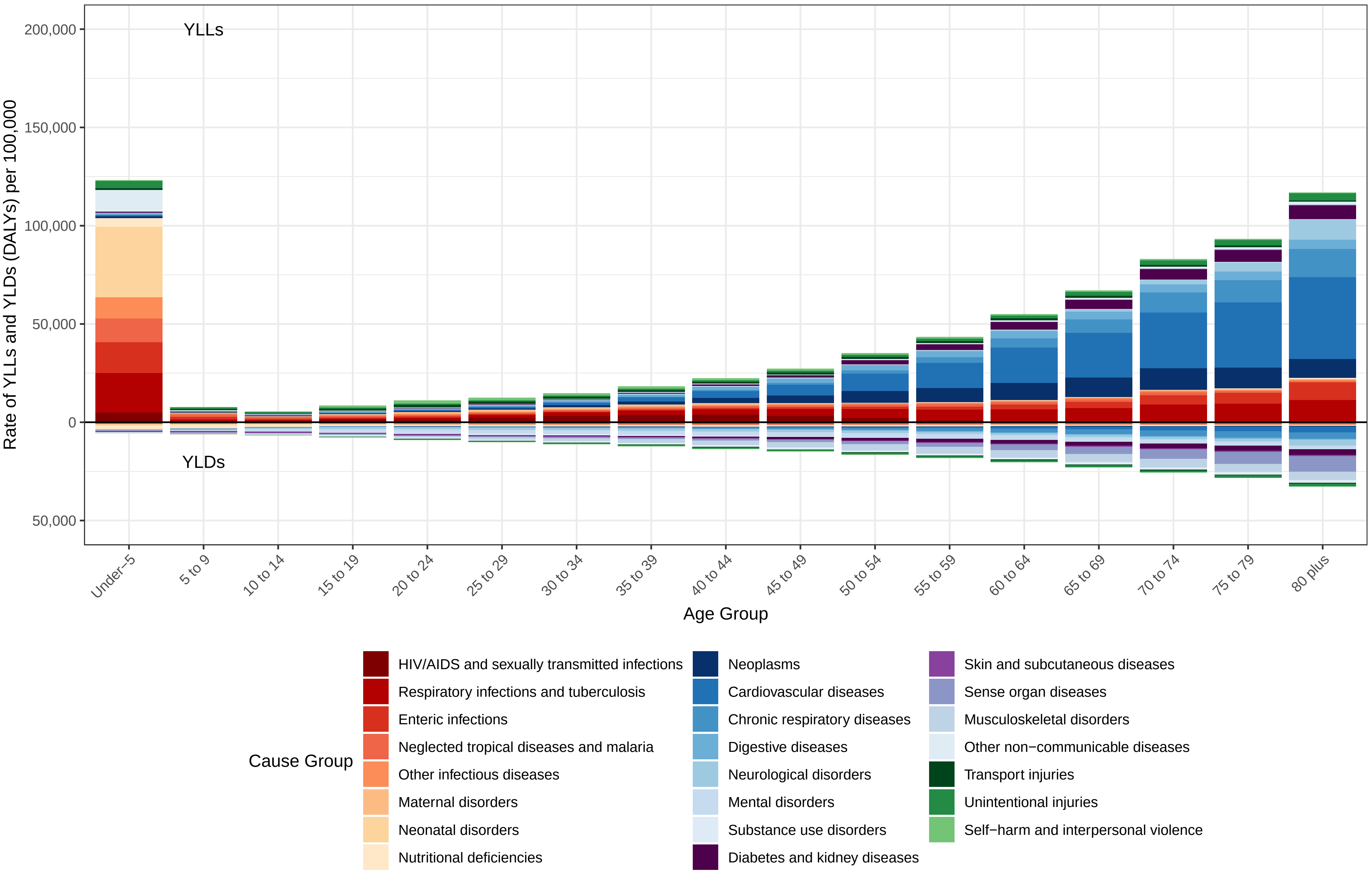

Appendix Figure 4c. Cause−specific DALY Rates by Age, Country Aggregation

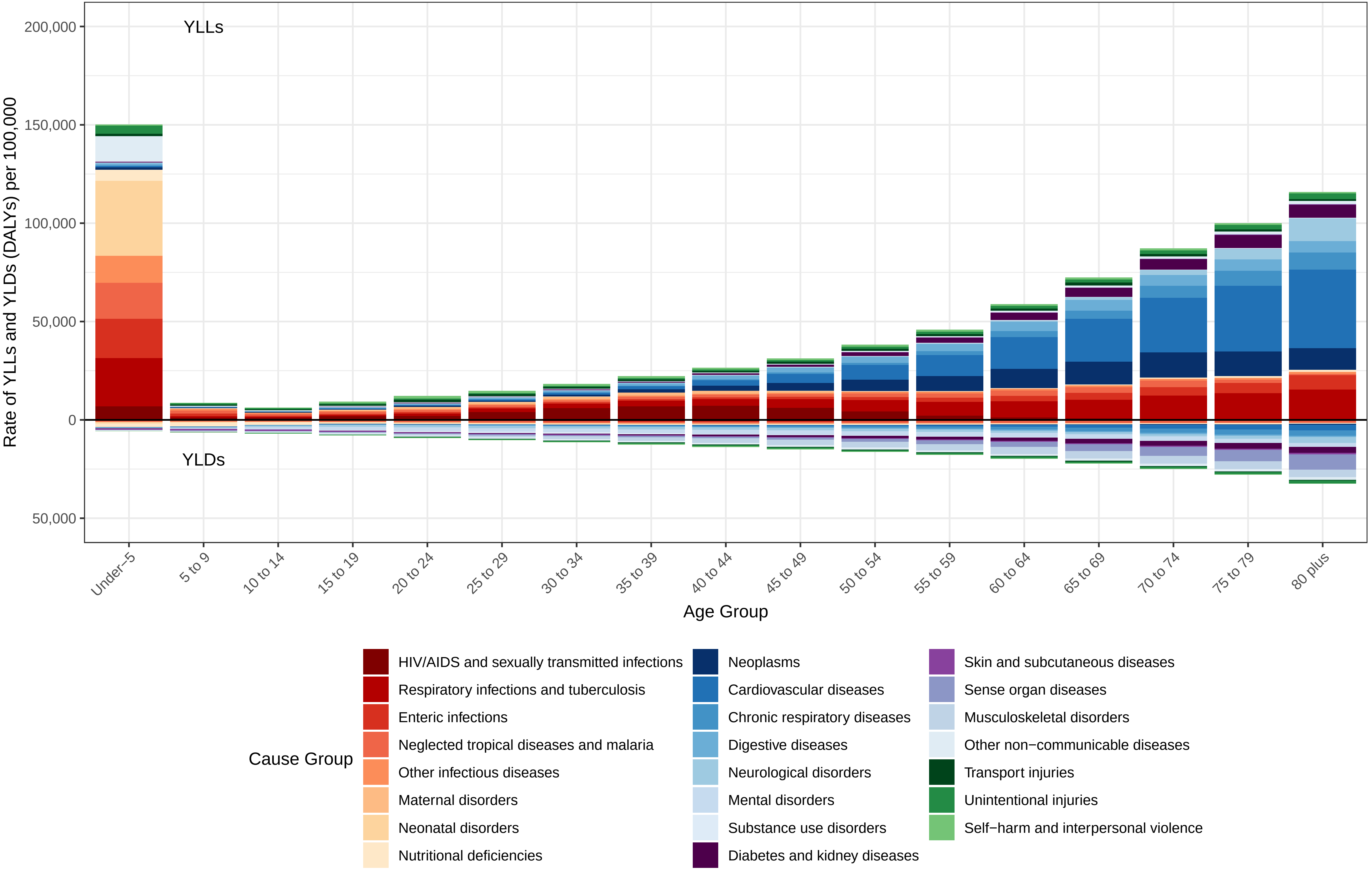

Appendix Figure 4d. Cause−specific DALY Rates by Age, Low−income Countries

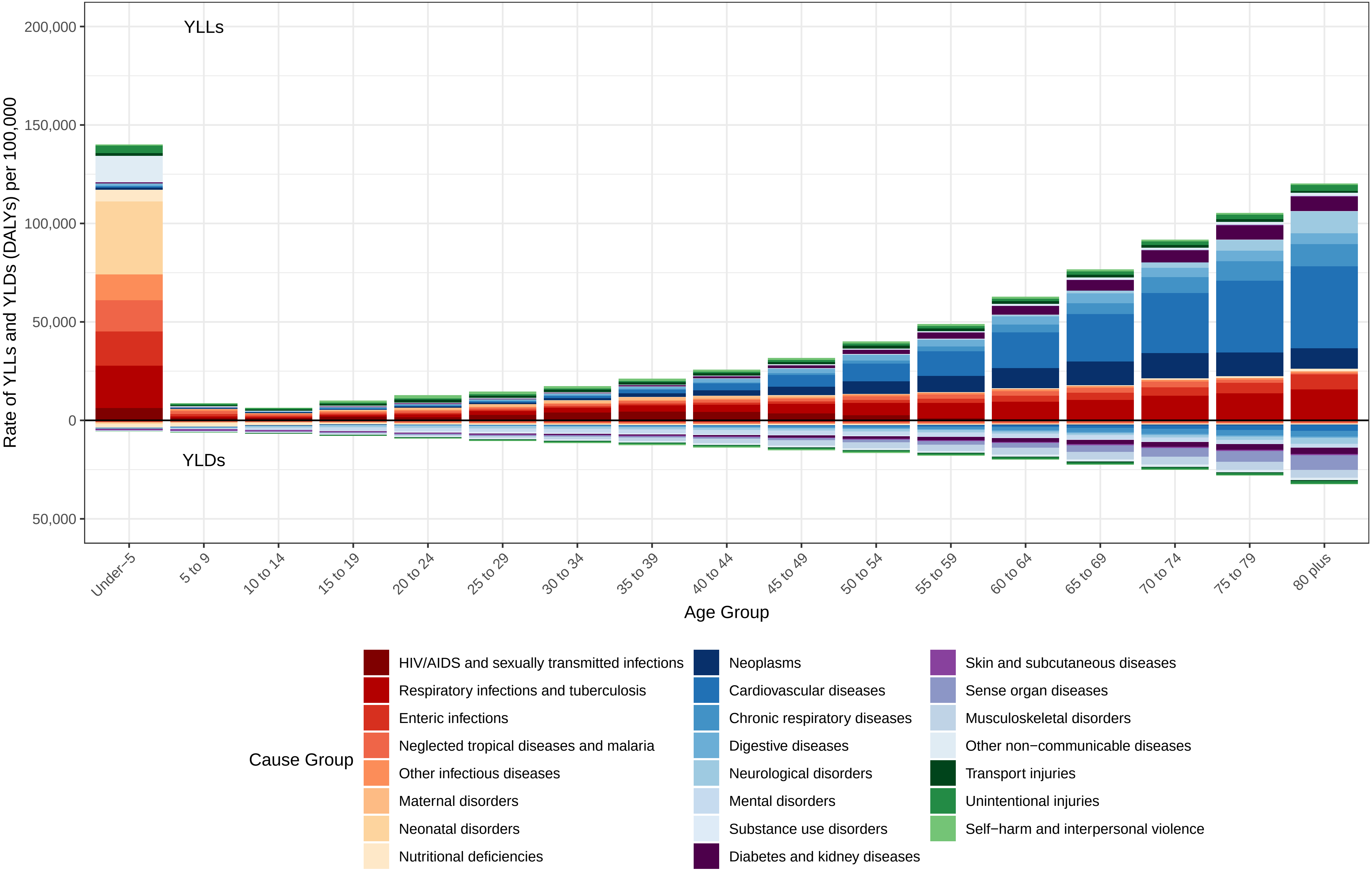

Appendix Figure 4e. Cause−specific DALY Rates by Age, Lower−middle−income Countries

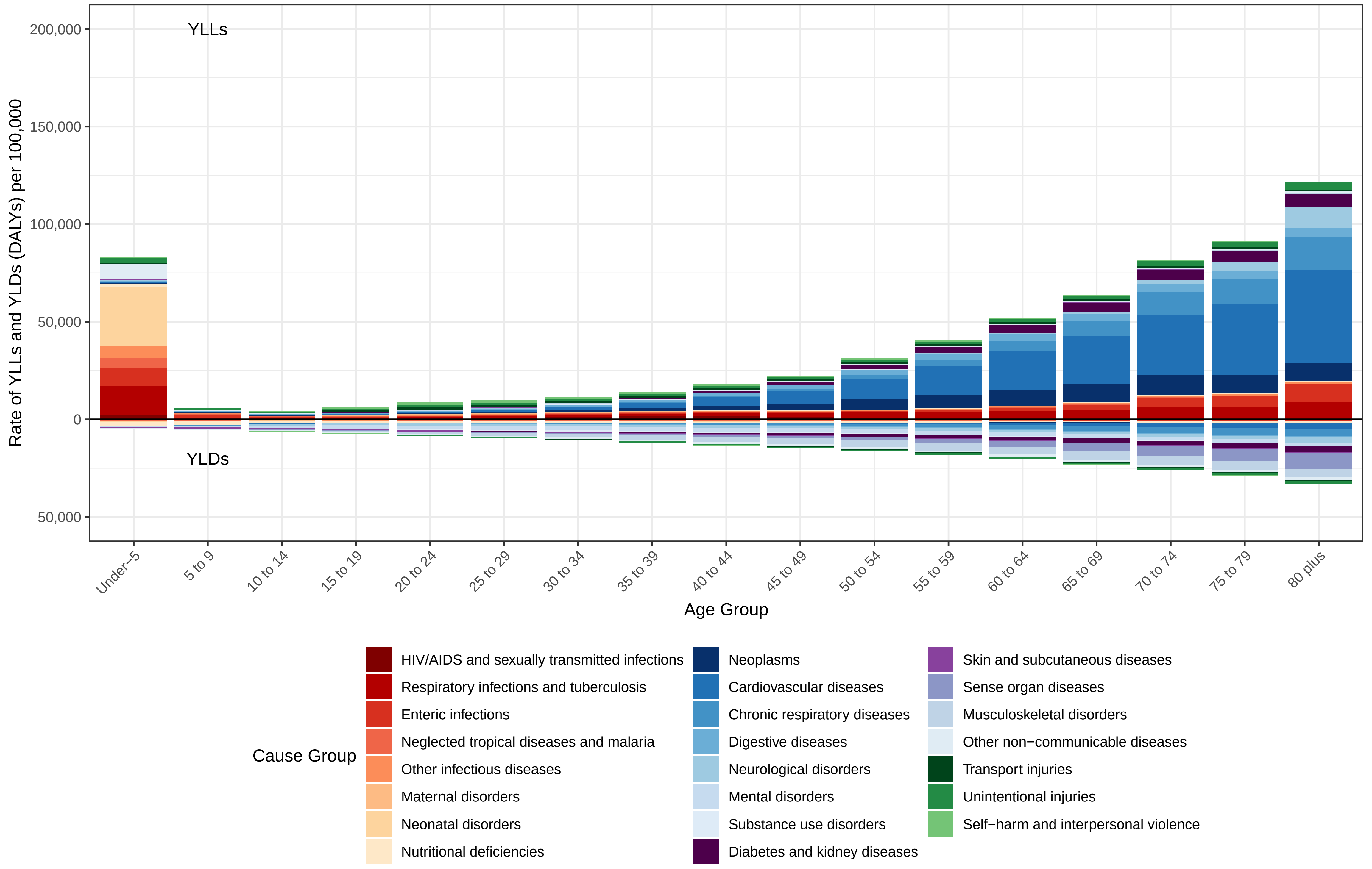

Appendix Figure 4f. Cause−specific DALY Rates by Age, Upper−middle−income Countries

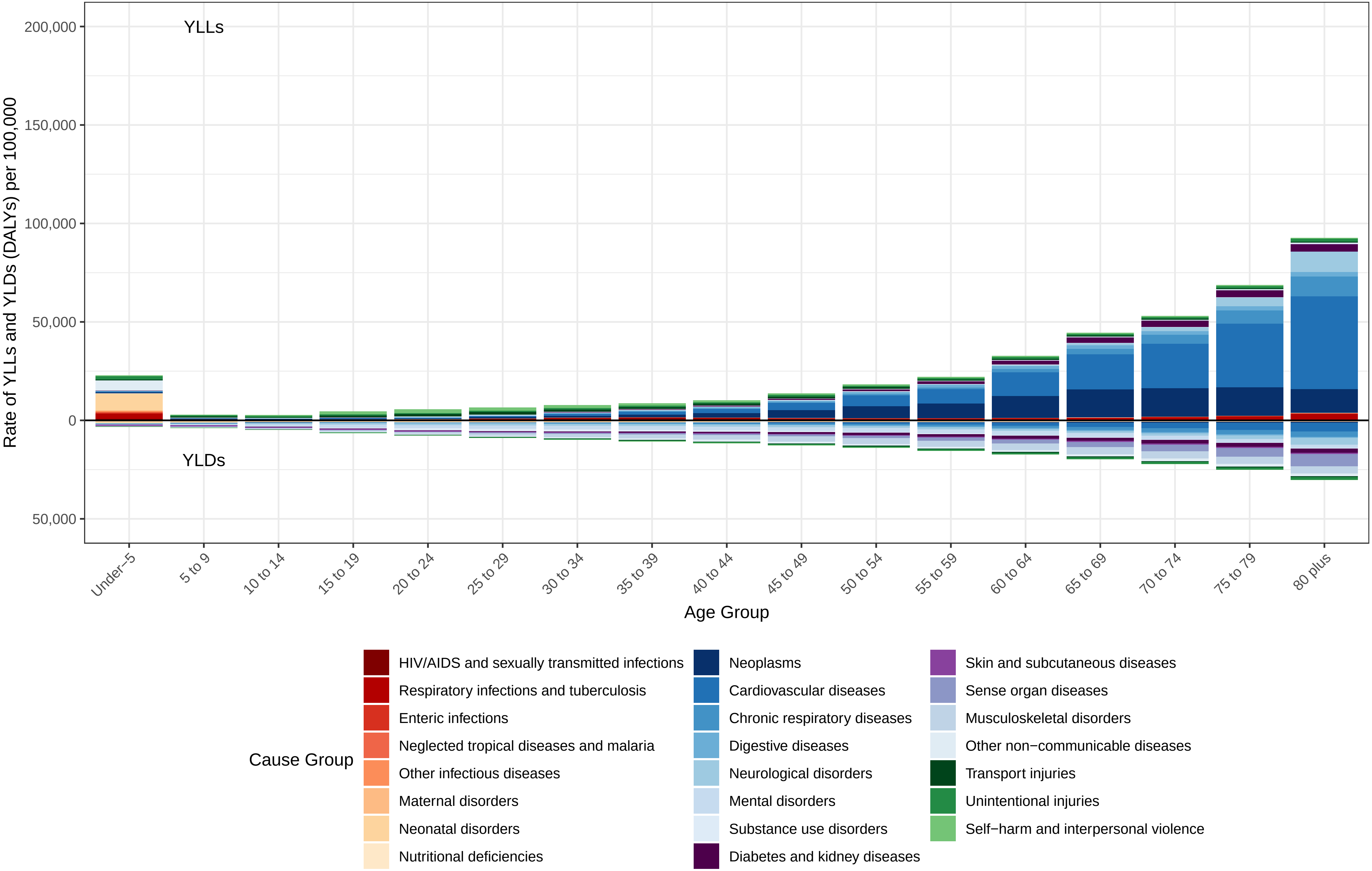

Appendix Figure 4g. Cause−specific DALY Rates by Age, High−income Countries

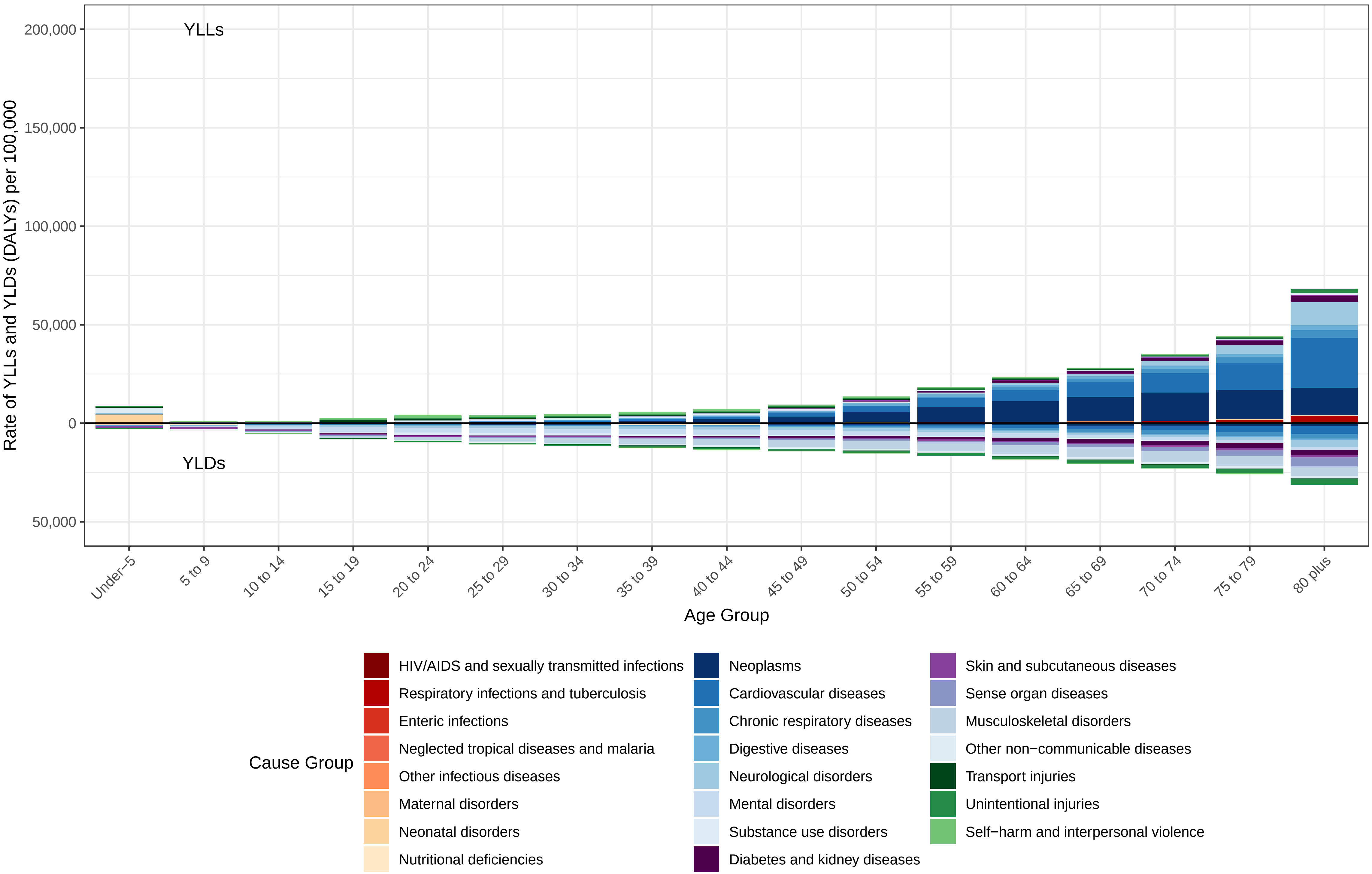

Appendix Figure 5a. Percent of Cause−Specific DALYs by Age, Full Ecological Approach

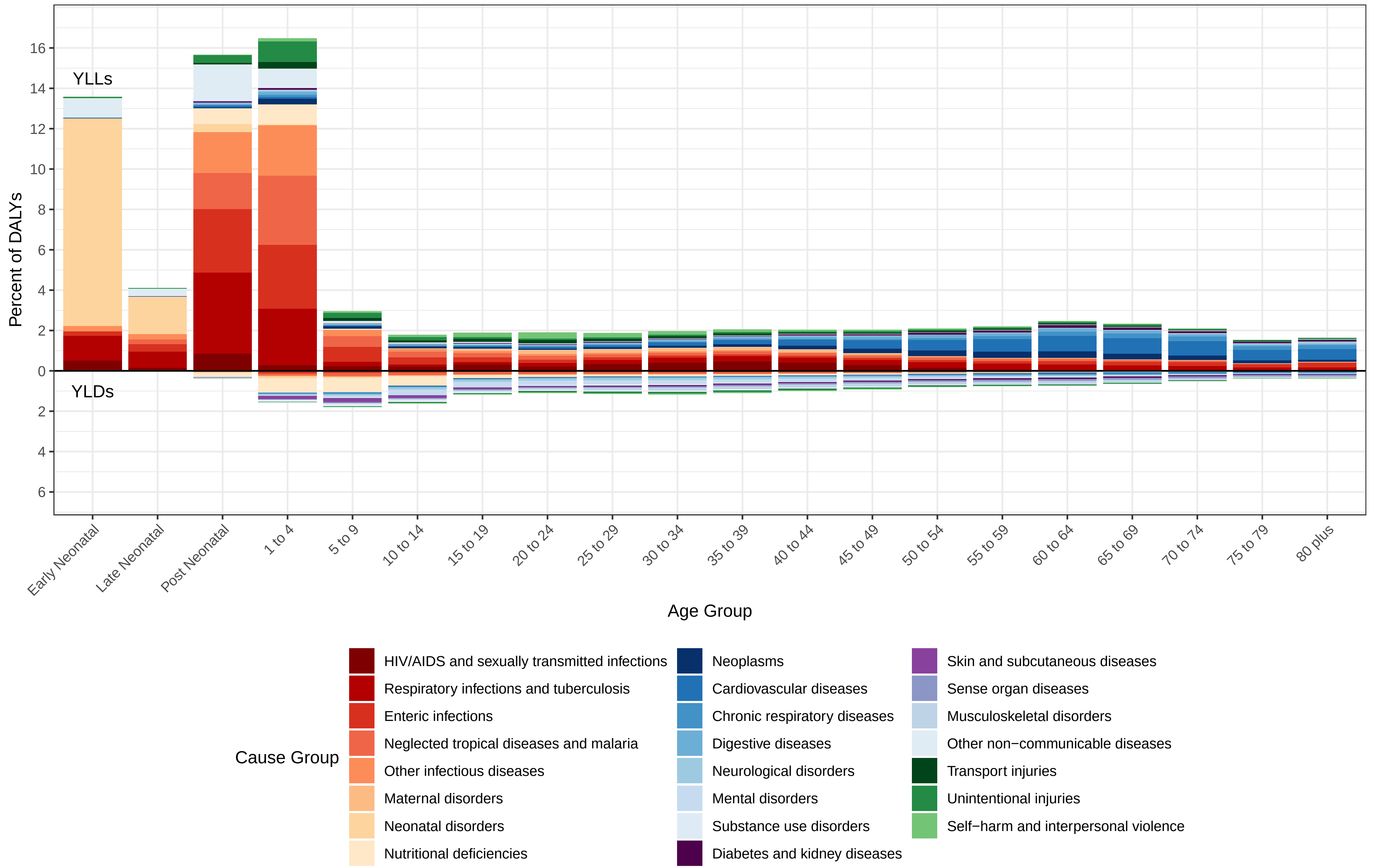

Appendix Figure 5b. Percent Cause−Specific of DALYs by Age, Poorest Billion Aggregation

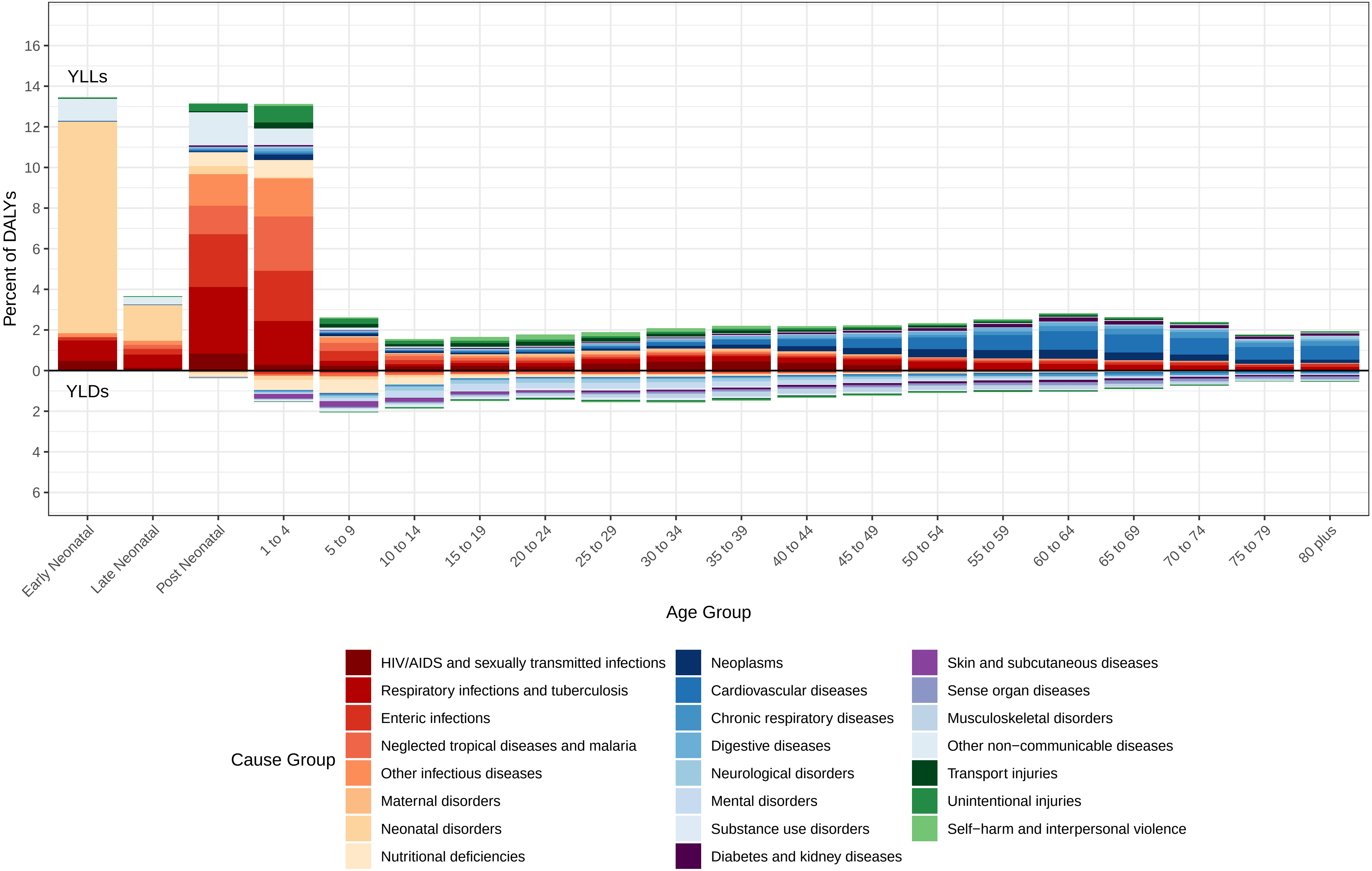

Appendix Figure 5c. Percent Cause−Specific of DALYs by Age, Country Aggregation

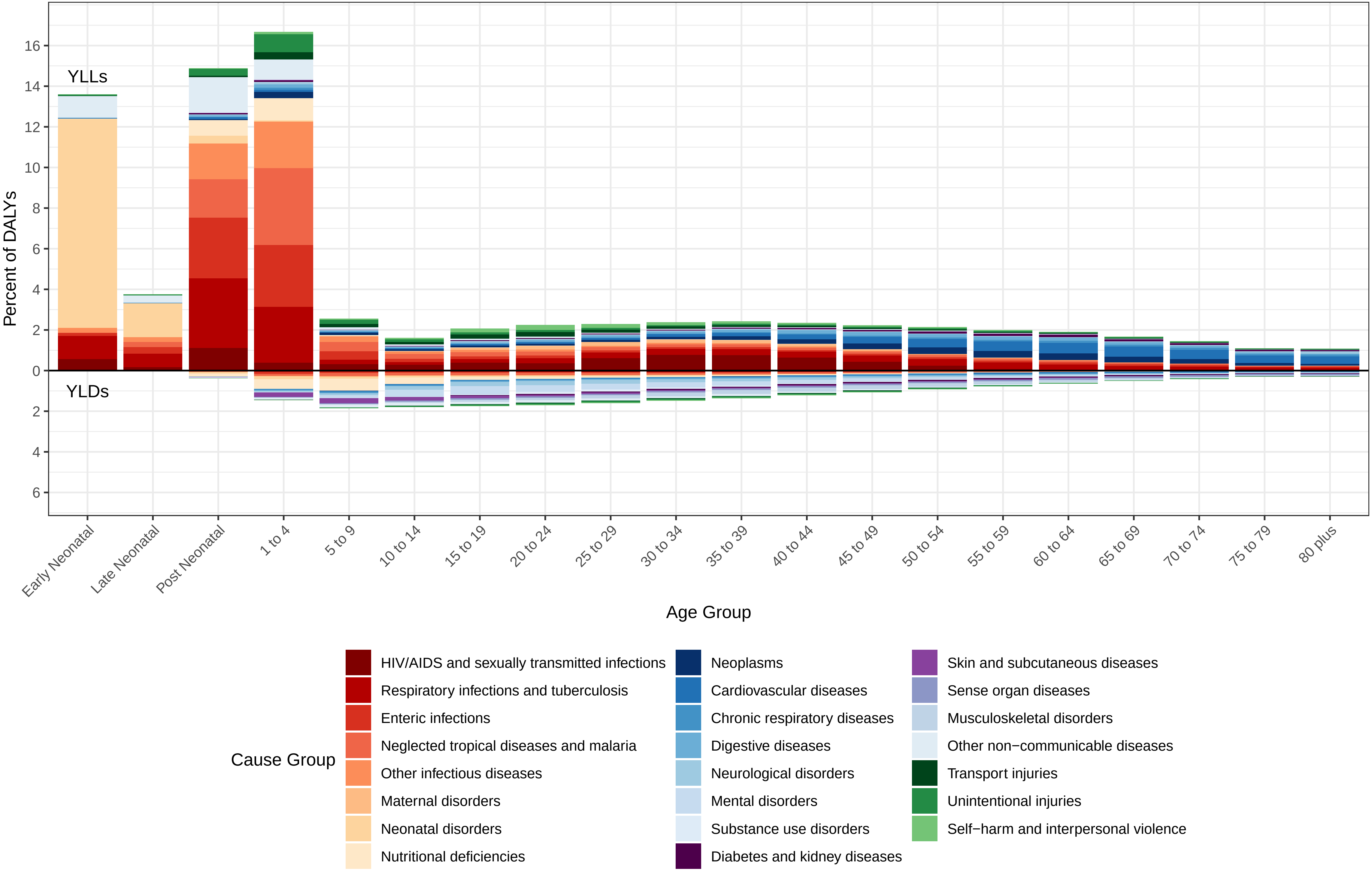

Appendix Figure 5d. Percent of Cause−Specific DALYs by Age, Low−income Countries

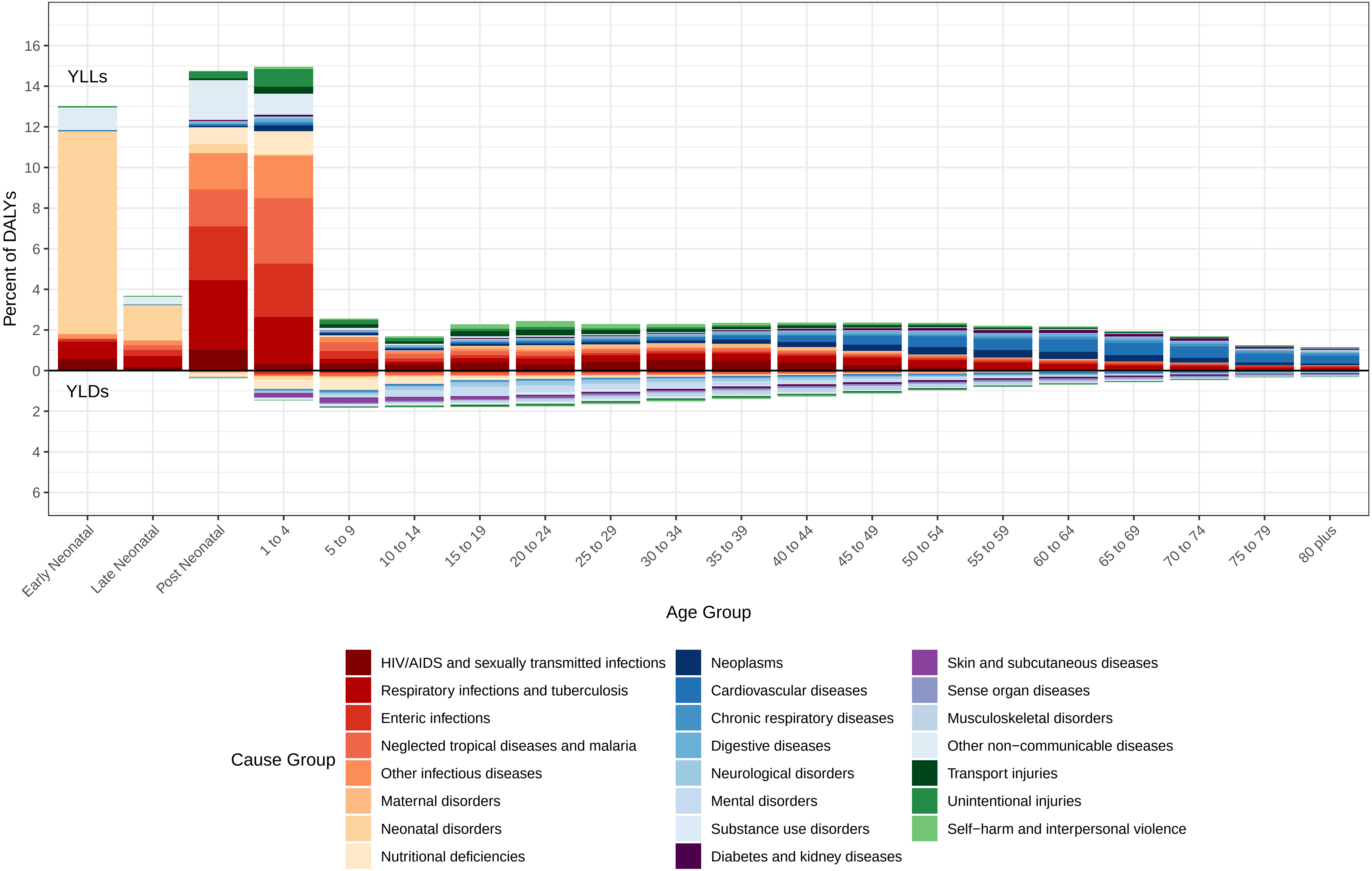

Appendix Figure 5e. Percent of Cause−Specific DALYs by Age, Lower−middle−income Countries

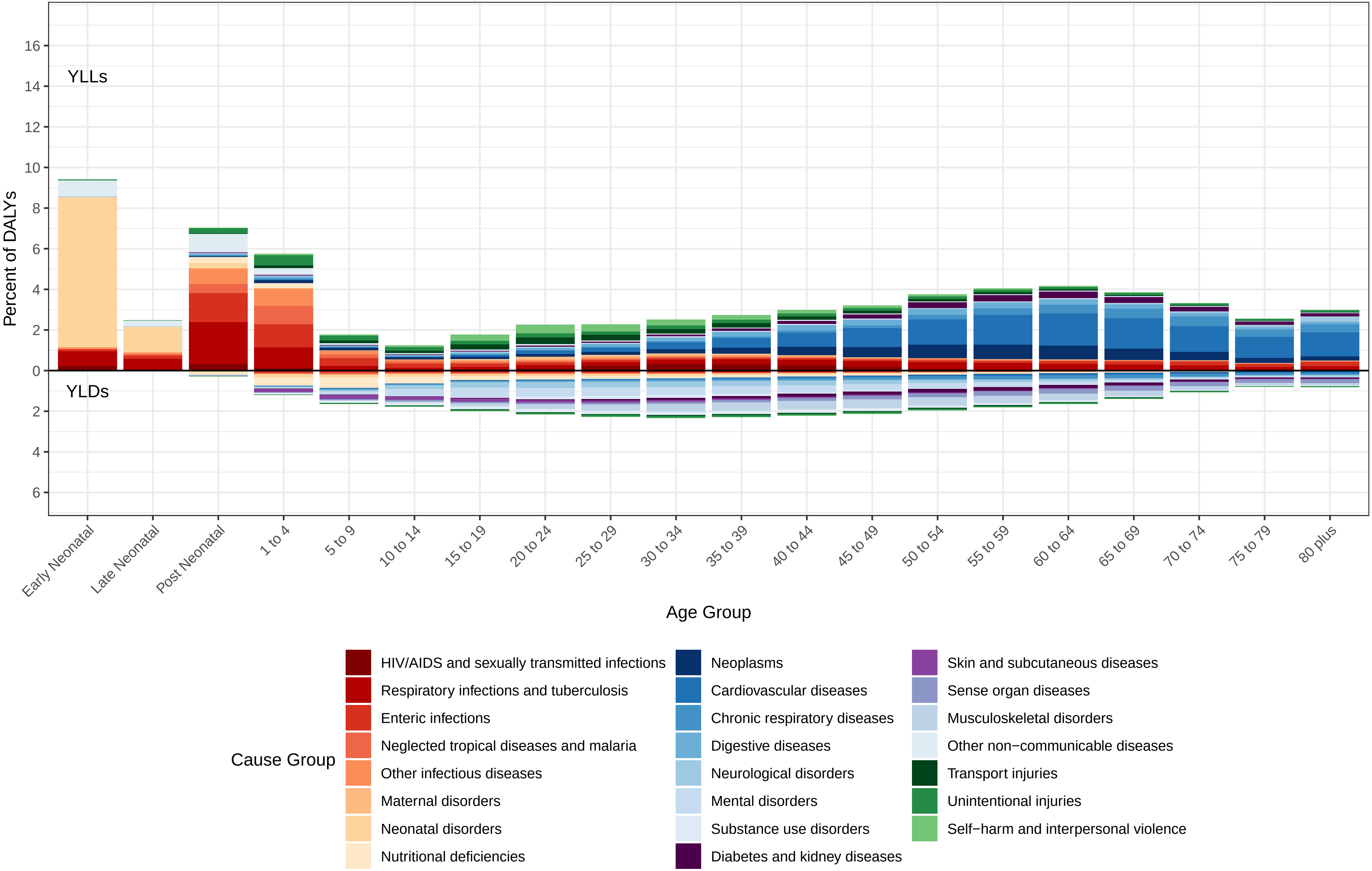

Appendix Figure 5f. Percent of Cause−Specific DALYs by Age, Upper−middle−income Countries

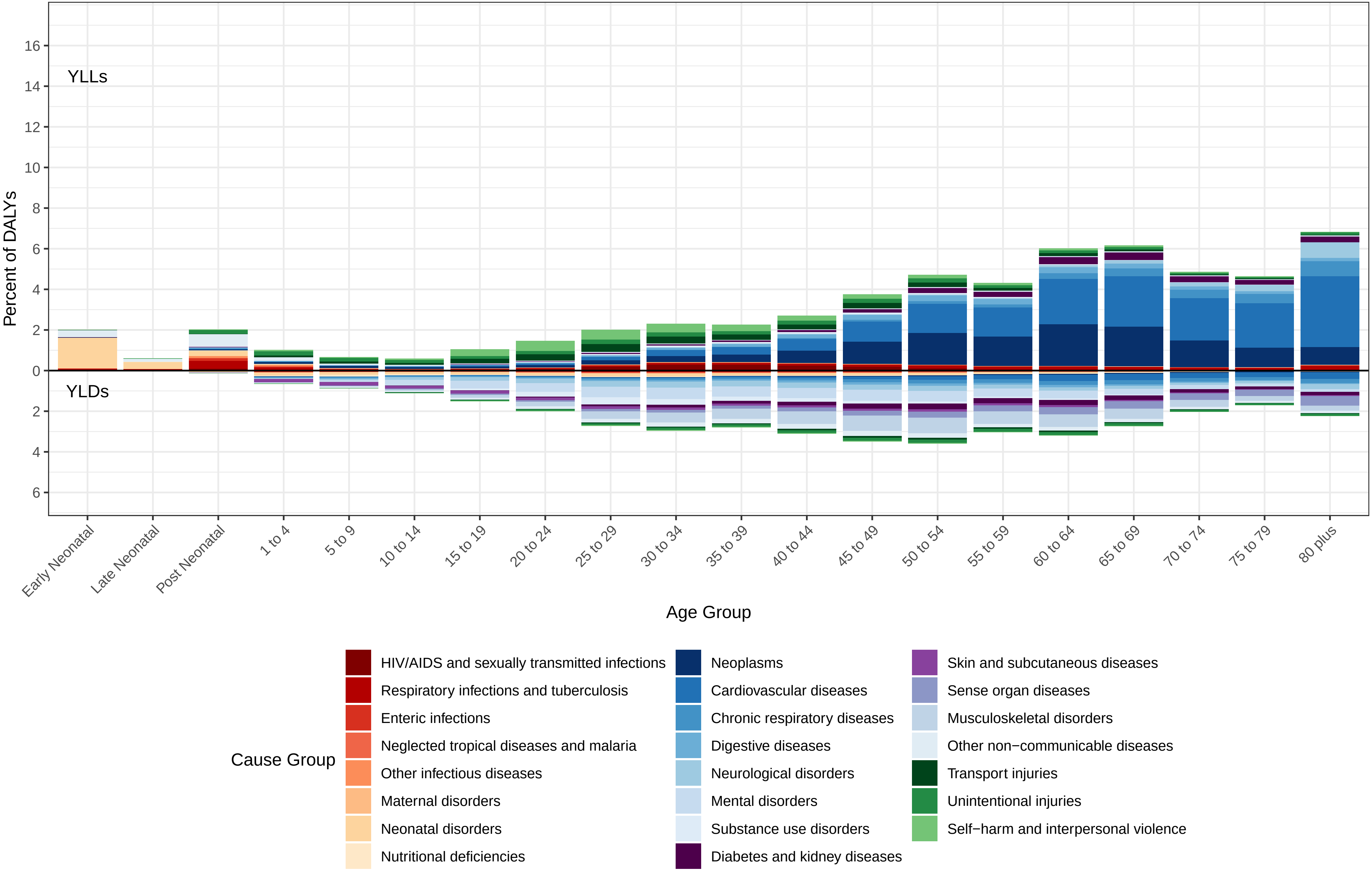

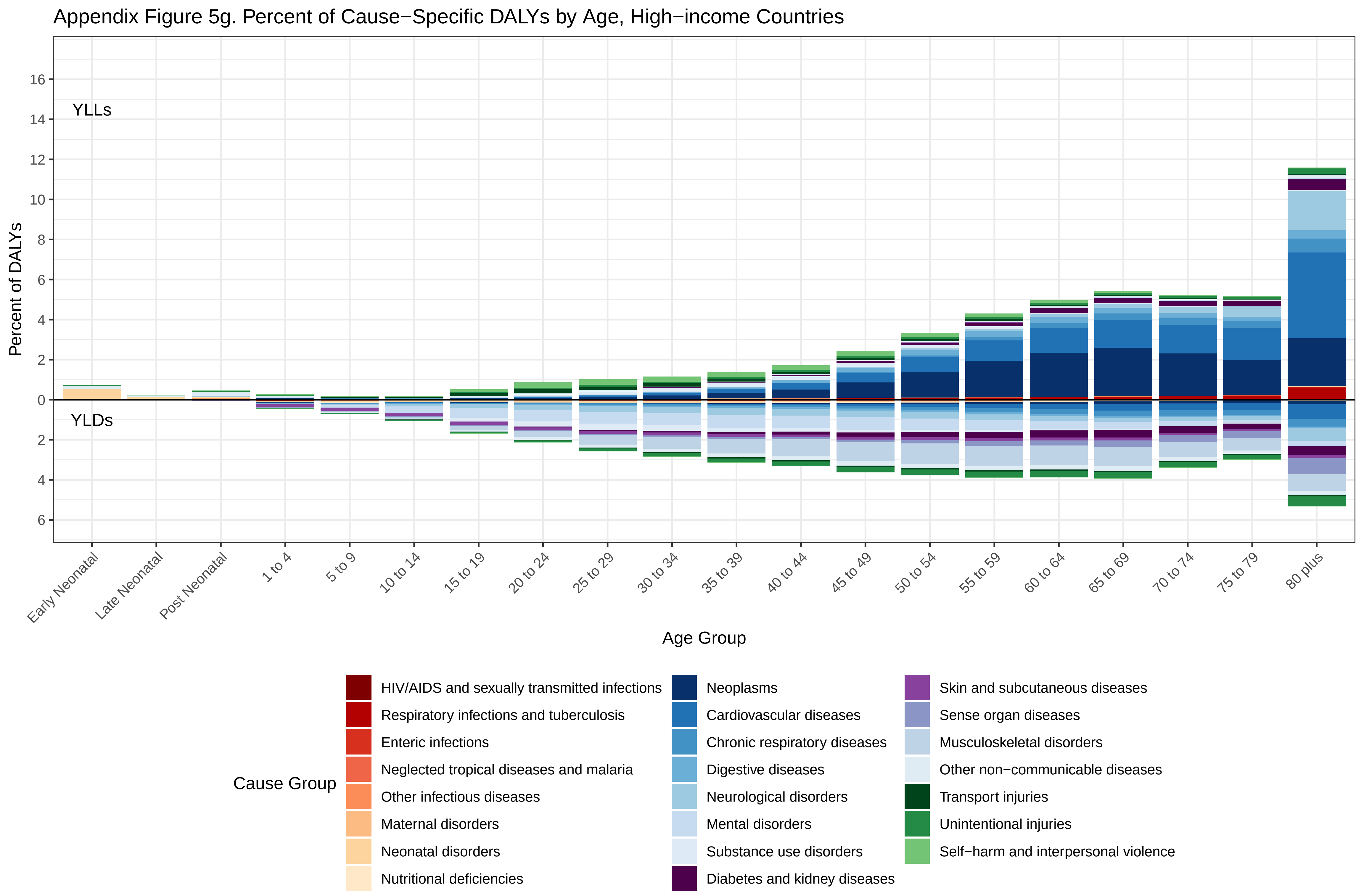
Appendix Figure 5g. Percent of Cause−Specific DALYs by Age, High−income Countries
YLLs
YLDs
Percent of DALYs
16
14
12
10
8
6
4
2
0
2
4
6
Early Neonatal
Late Neonatal
Post Neonatal
1 to 4
5 to 9
10 to 14
15 to 19
20 to 24
25 to 29
30 to 34
35 to 39
40 to 44
45 to 49
50 to 54
55 to 59
60 to 64
65 to 69
70 to 74
75 to 79
80 plus
Age Group
Cause Group
HIV/AIDS and sexually transmitted infections
Respiratory infections and tuberculosis
Enteric infections
Neglected tropical diseases and malaria
Other infectious diseases
Maternal disorders
Neonatal disorders
Nutritional deficiencies
Neoplasms
Cardiovascular diseases
Chronic respiratory diseases
Digestive diseases
Neurological disorders
Mental disorders
Substance use disorders
Diabetes and kidney diseases
Skin and subcutaneous diseases
Sense organ diseases
Musculoskeletal disorders
Other non−communicable diseases
Transport injuries
Unintentional injuries
Self−harm and interpersonal violence

Appendix Figure 6a. Difference between Crude DALY Rates in Poorest Billion and High−income Regions, Full Ecological Approach

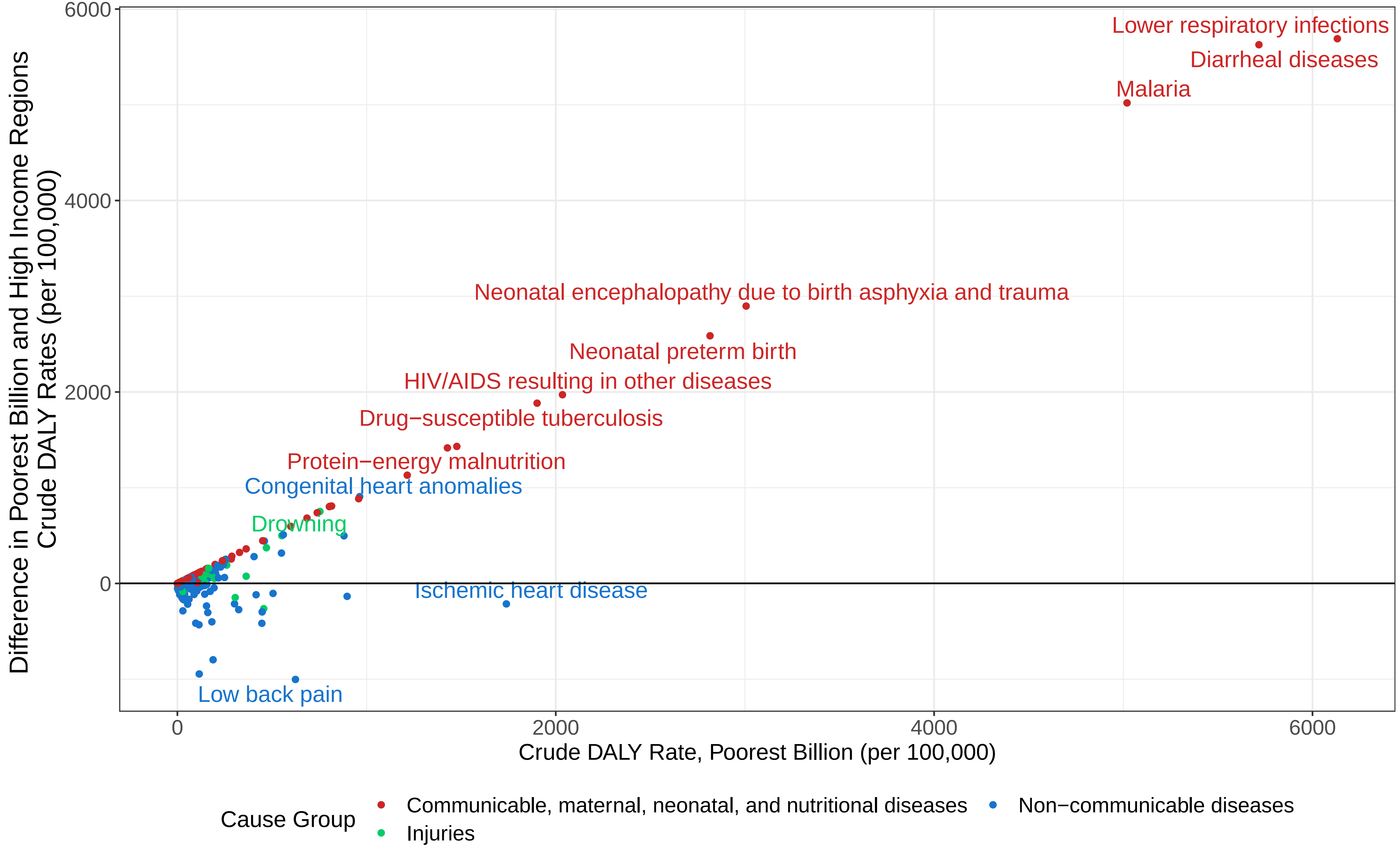

Appendix Figure 6b. Difference between Age−standardized DALY Rates in Poorest Billion and High−income Regions, Full Ecological Approach

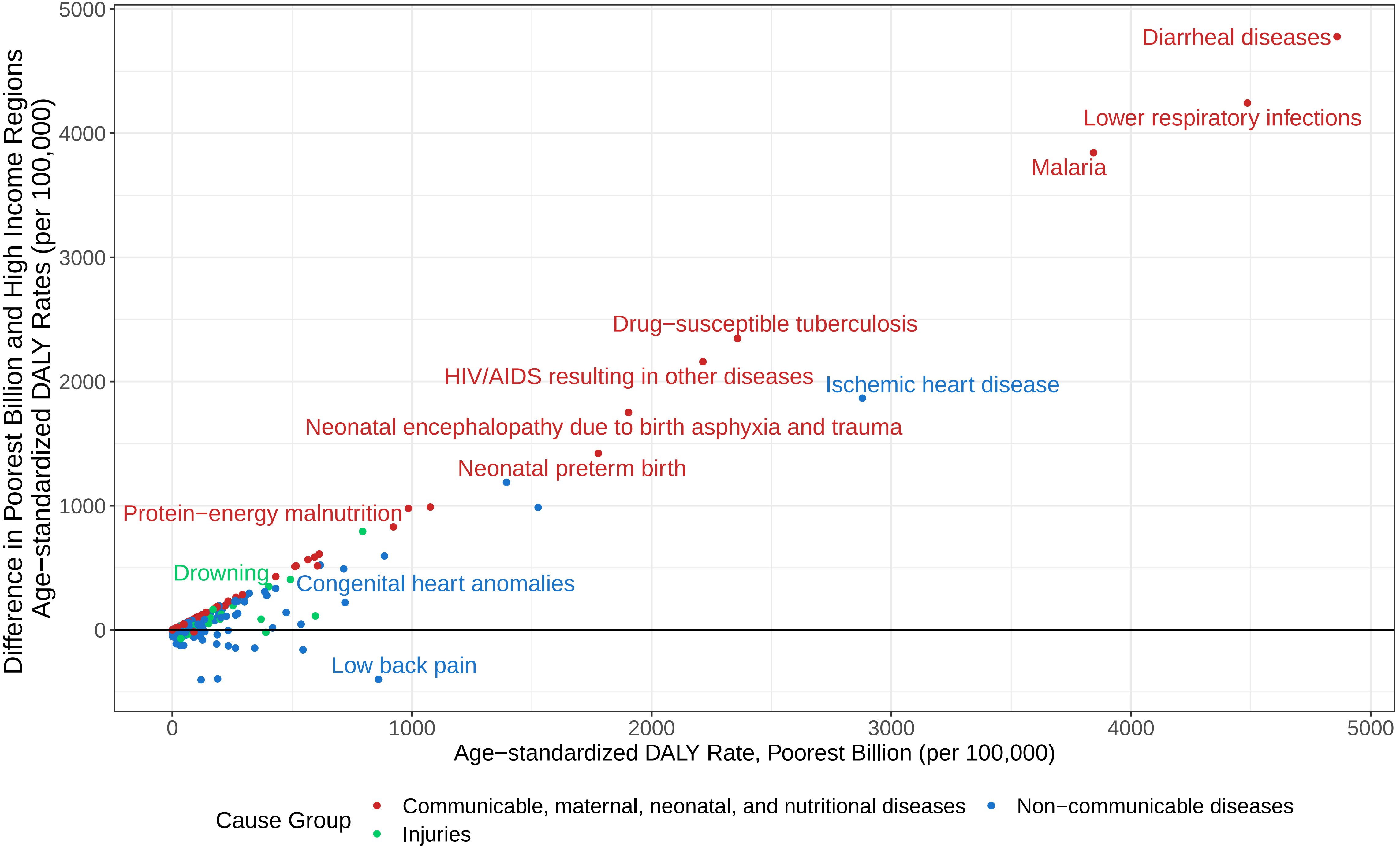

Appendix Figure 6c. Difference between Crude DALY Rates in Poorest Billion and High−income Regions, Poorest Billion Aggregation

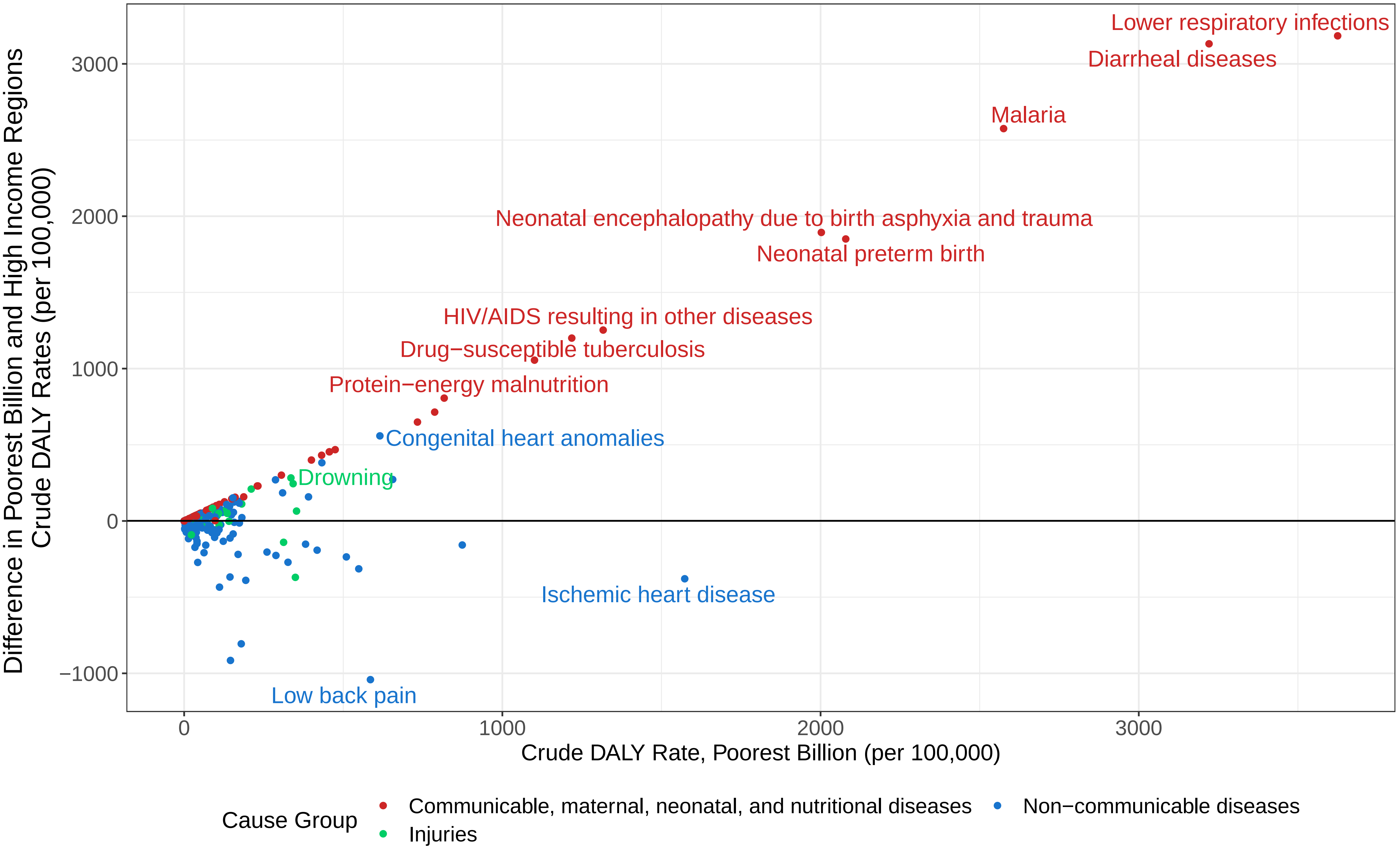

Appendix Figure 6d. Difference between Age−standardized DALY Rates in Poorest Billion and High−income Regions, Poorest Billion Aggregation

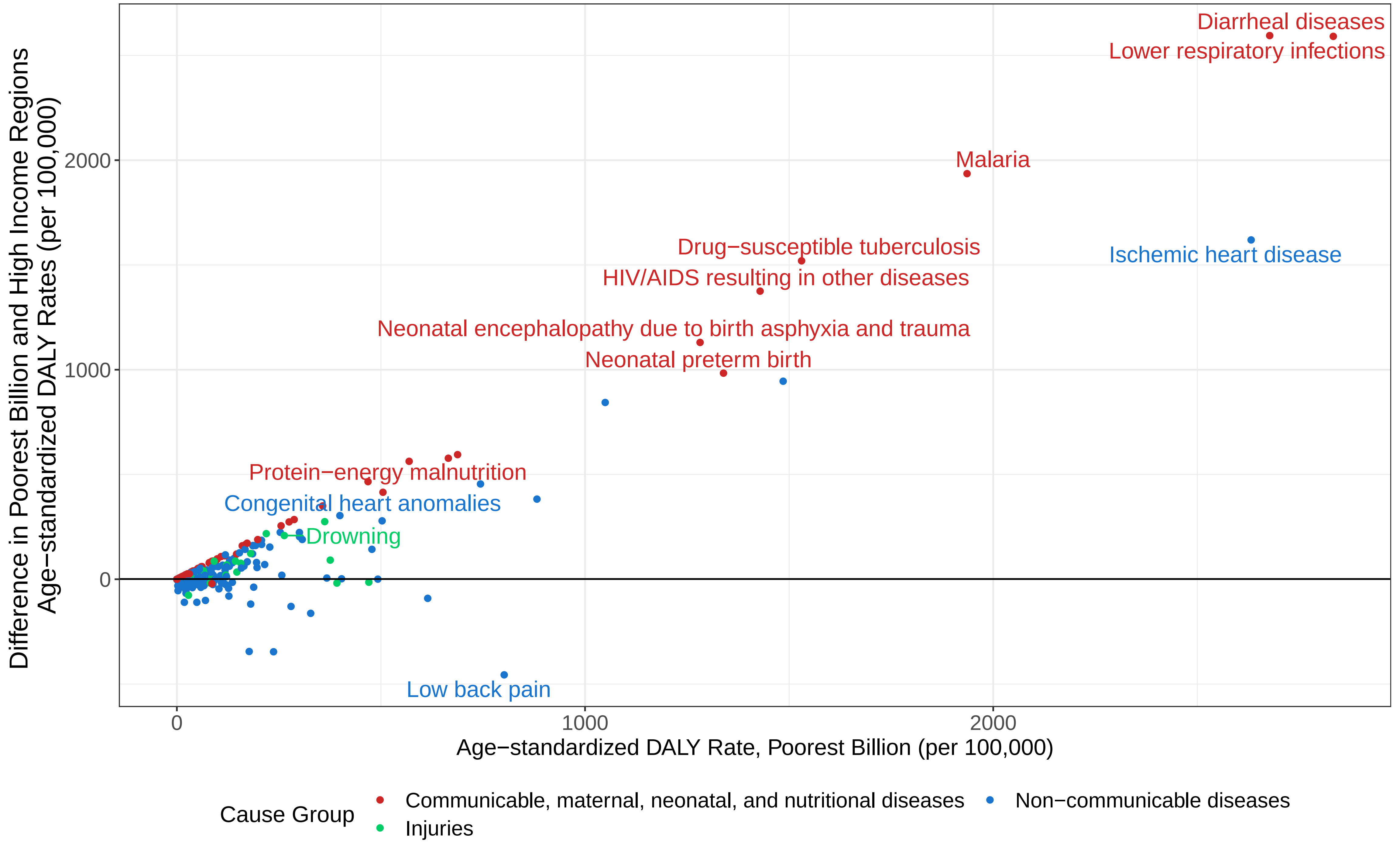

Appendix Figure 6e. Difference between Crude DALY Rates in Poorest Billion and High−income Regions, Country Aggregation

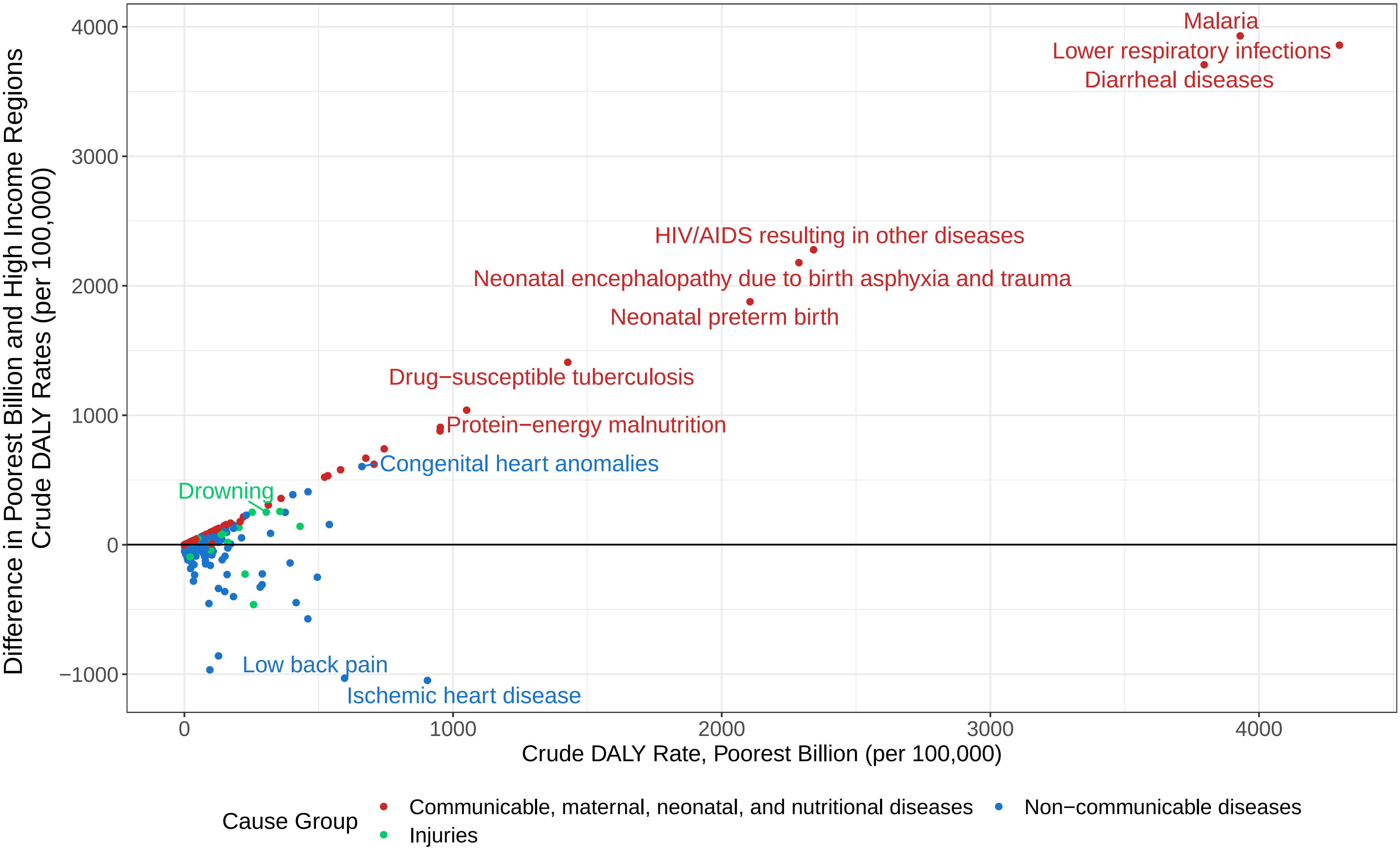

Appendix Figure 6f. Difference between Age−standardized DALY Rates in Poorest Billion and High−income Regions, Country Aggregation

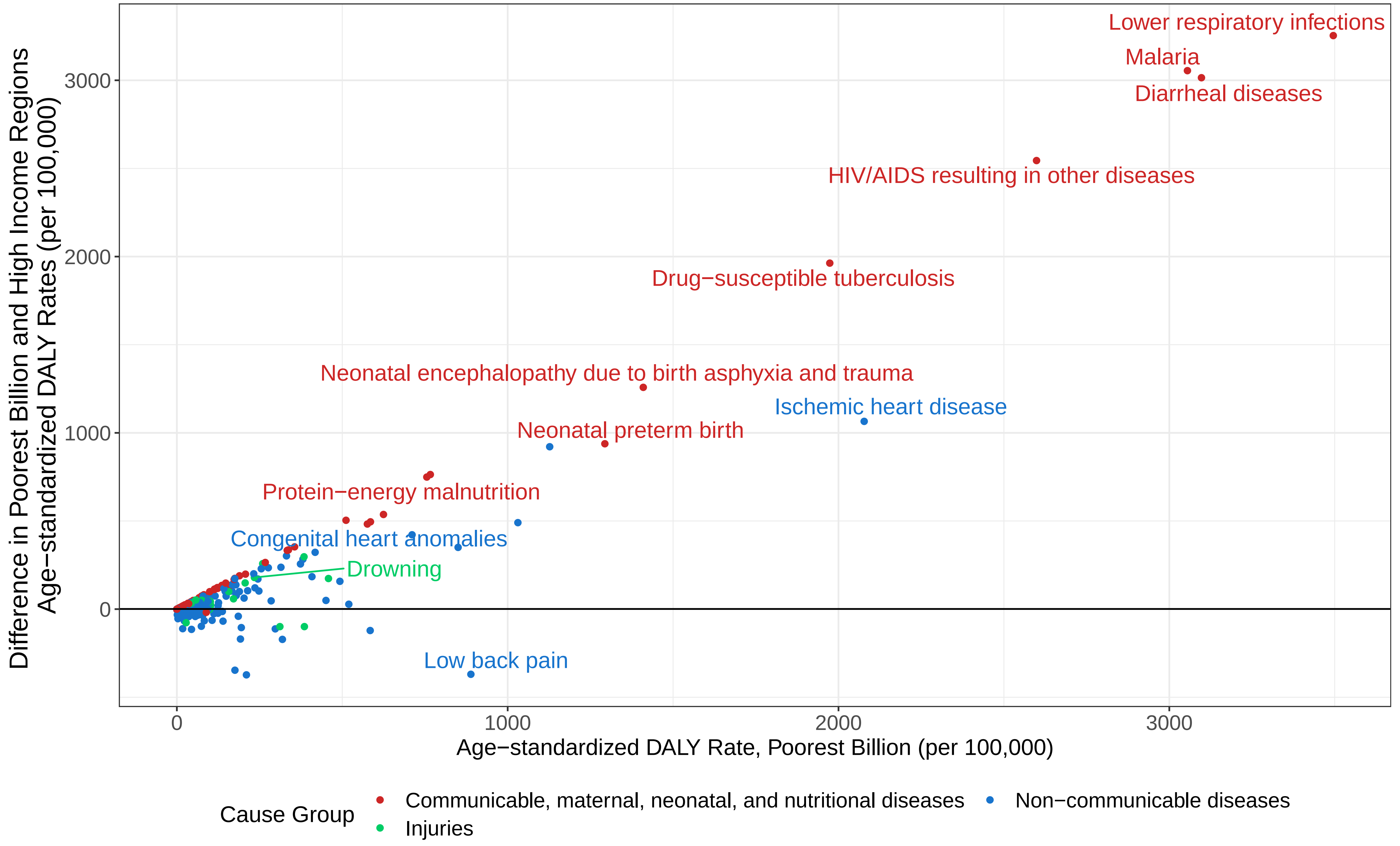

Appendix Figure 6g. Difference between Crude DALY Rates in Low−income and High−income Regions

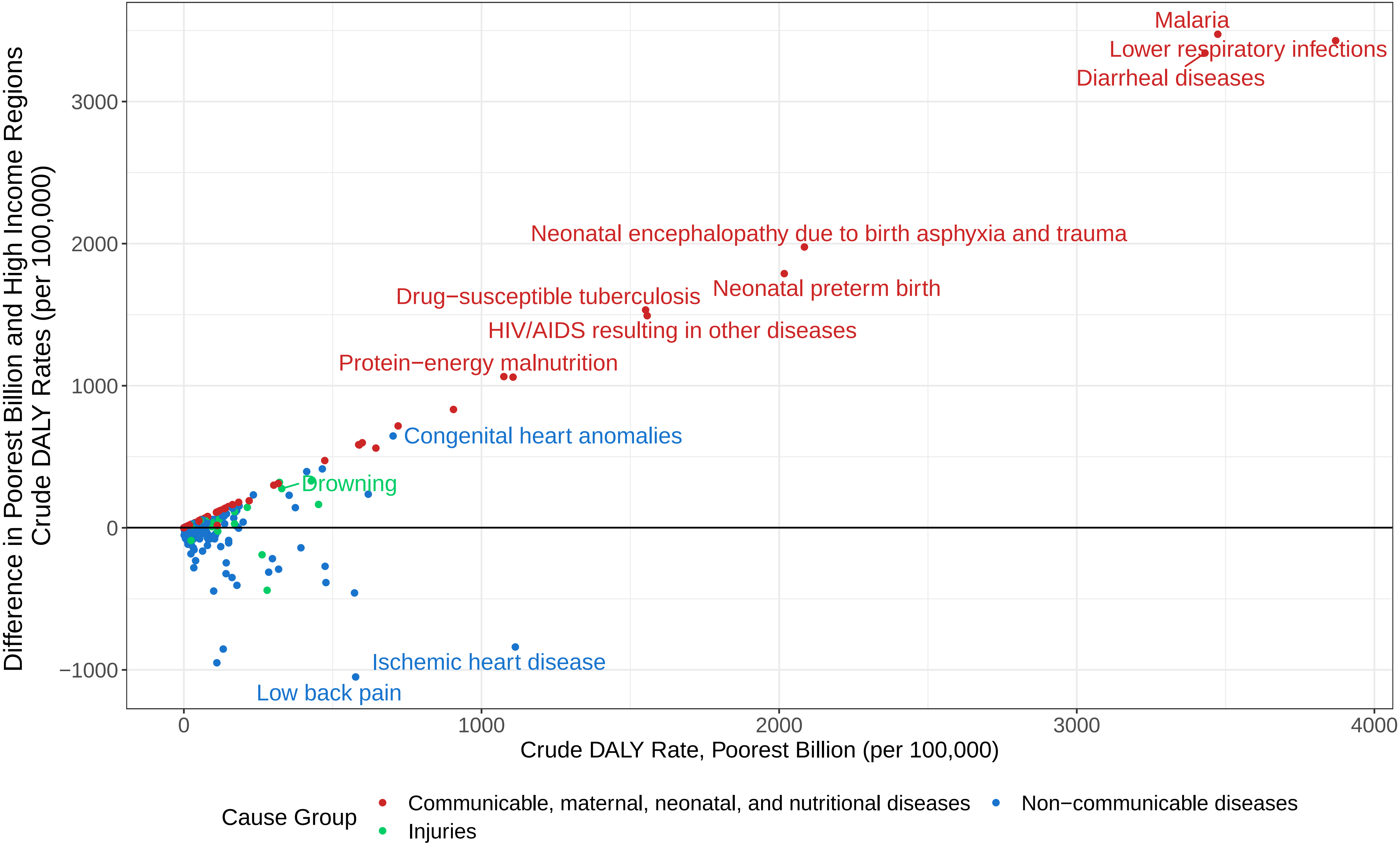

Appendix Figure 6h. Difference between Age−standardized DALY Rates in Low−income and High−income Regions

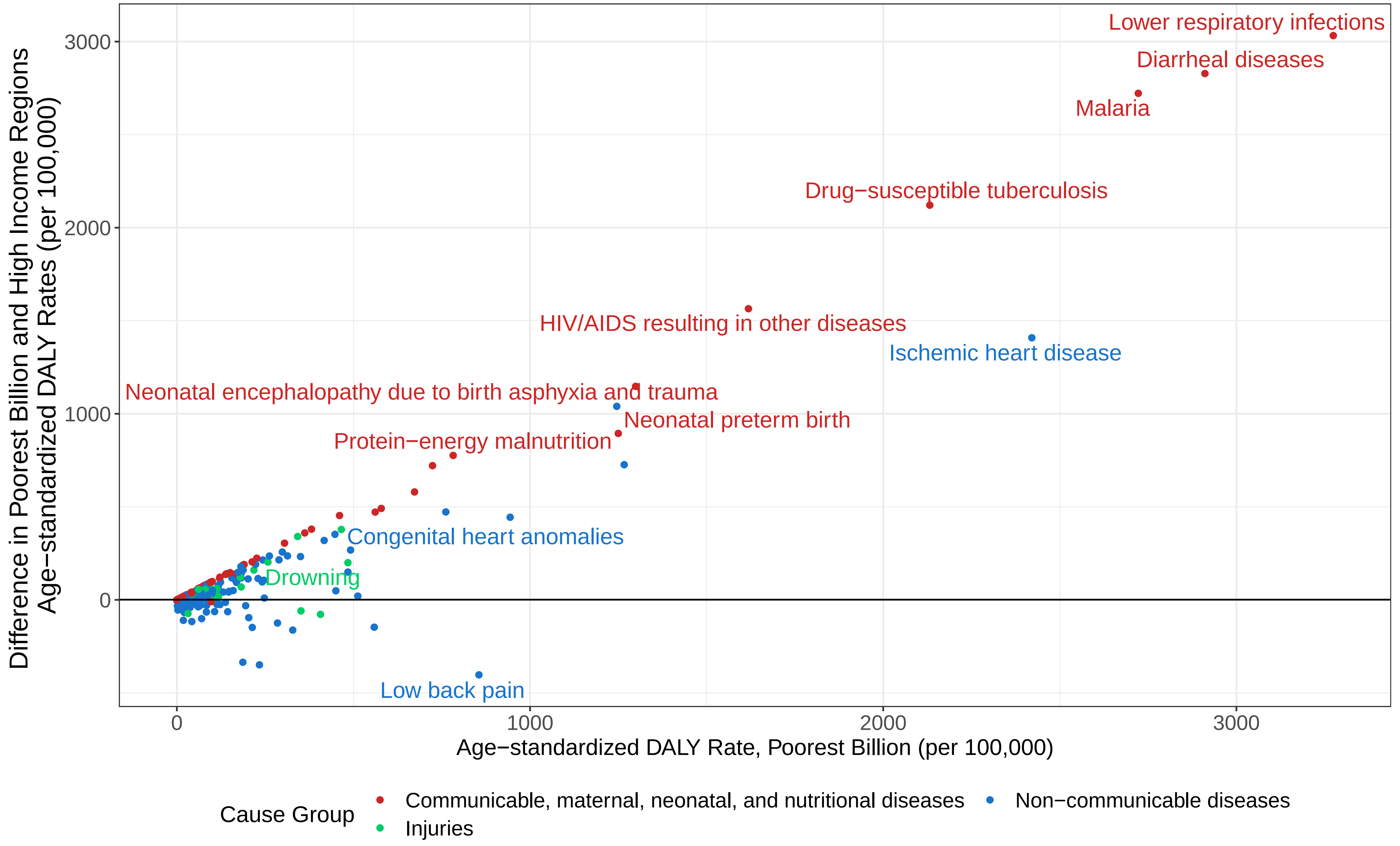

Appendix Figure 7a. Age−specific Percent of DALYs by Cause, Selective Ecological Approach

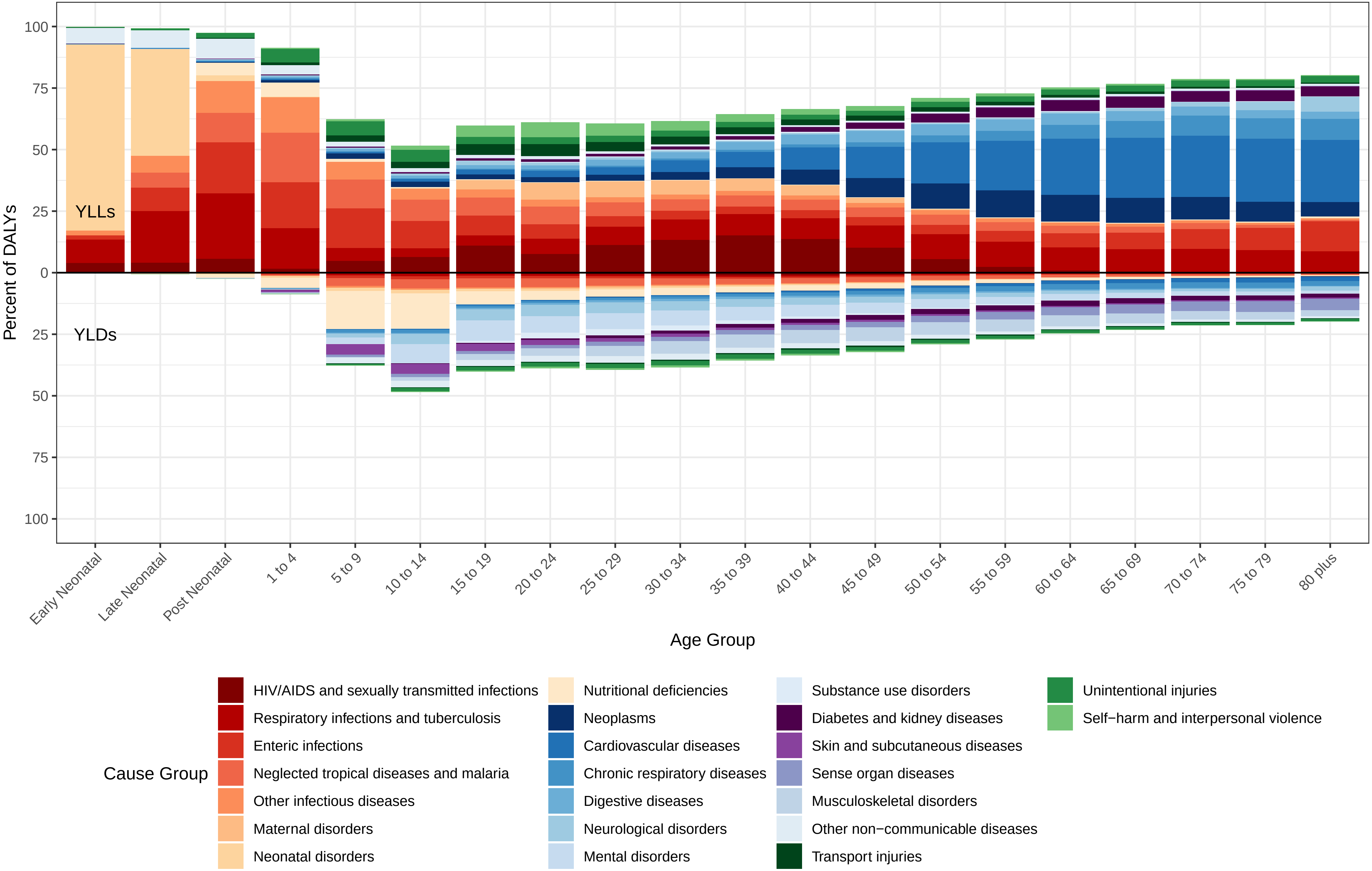

Appendix Figure 7b. Age−specific Percent of DALYs by Cause, High−income Regions

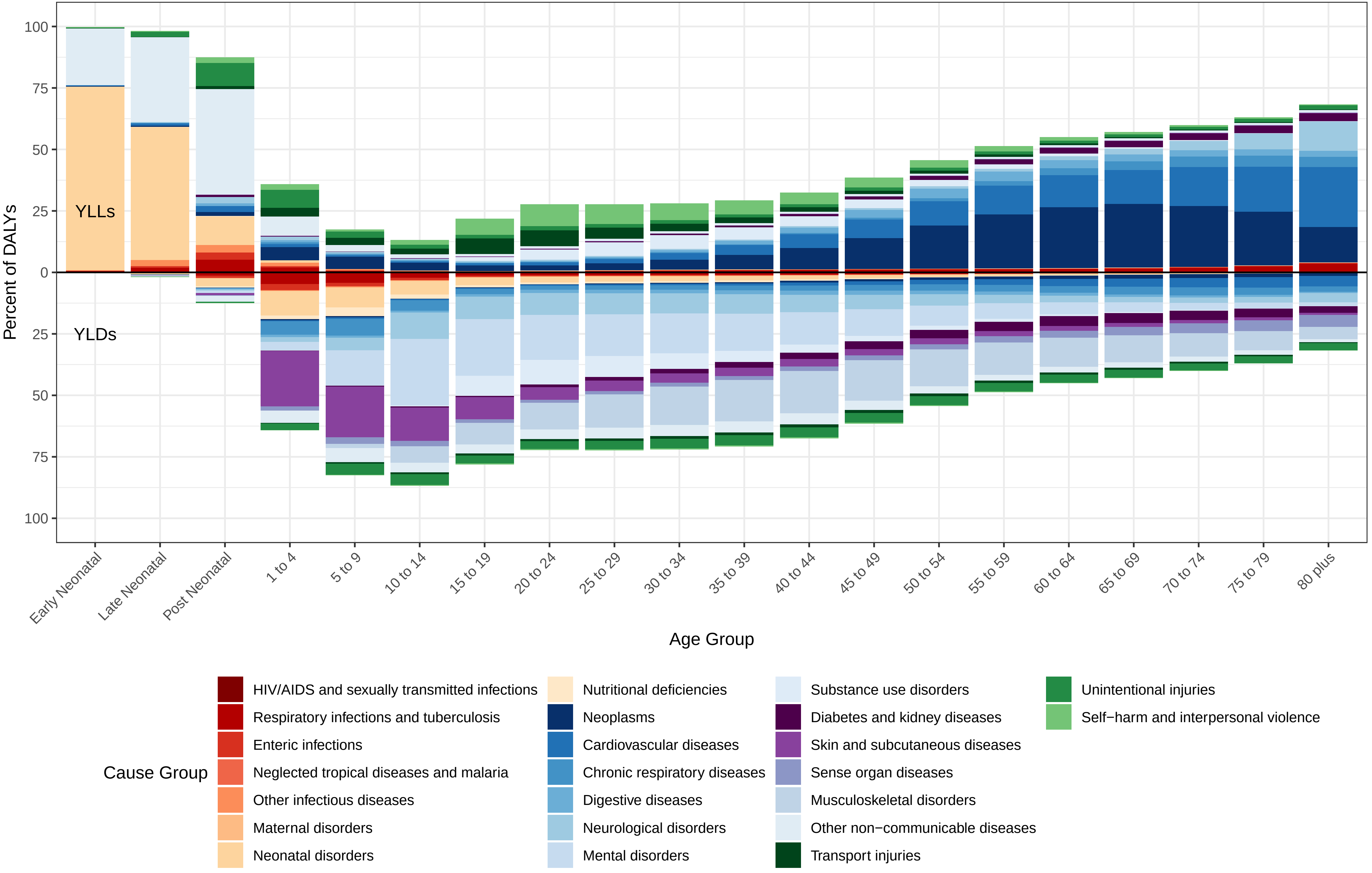

Appendix Figure 7c. Age−specific Percent of DALYs by Cause, Full Ecological Approach

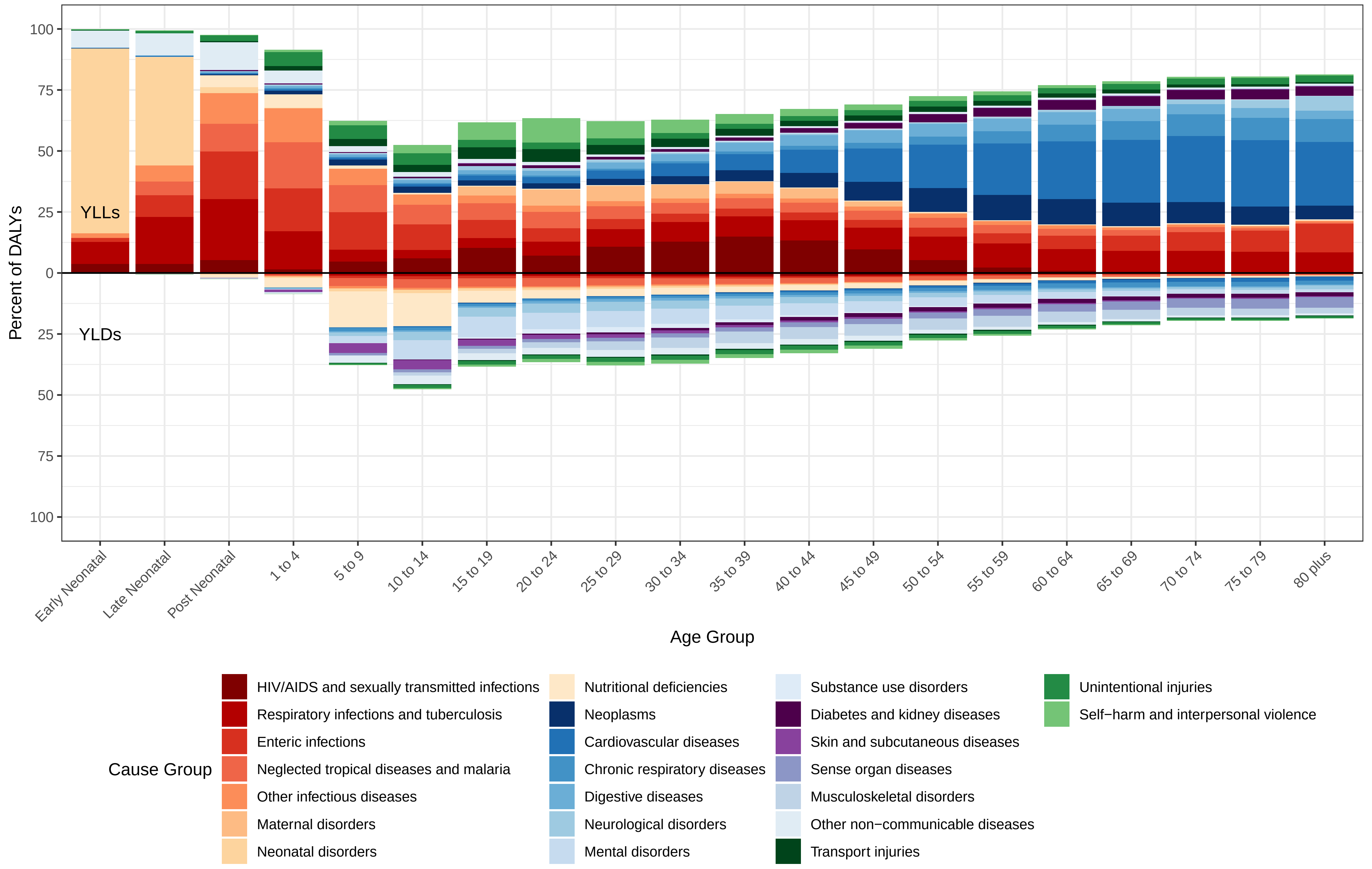

Appendix Figure 7d. Age−specific Percent of DALYs by Cause, Poorest Billion Aggregation

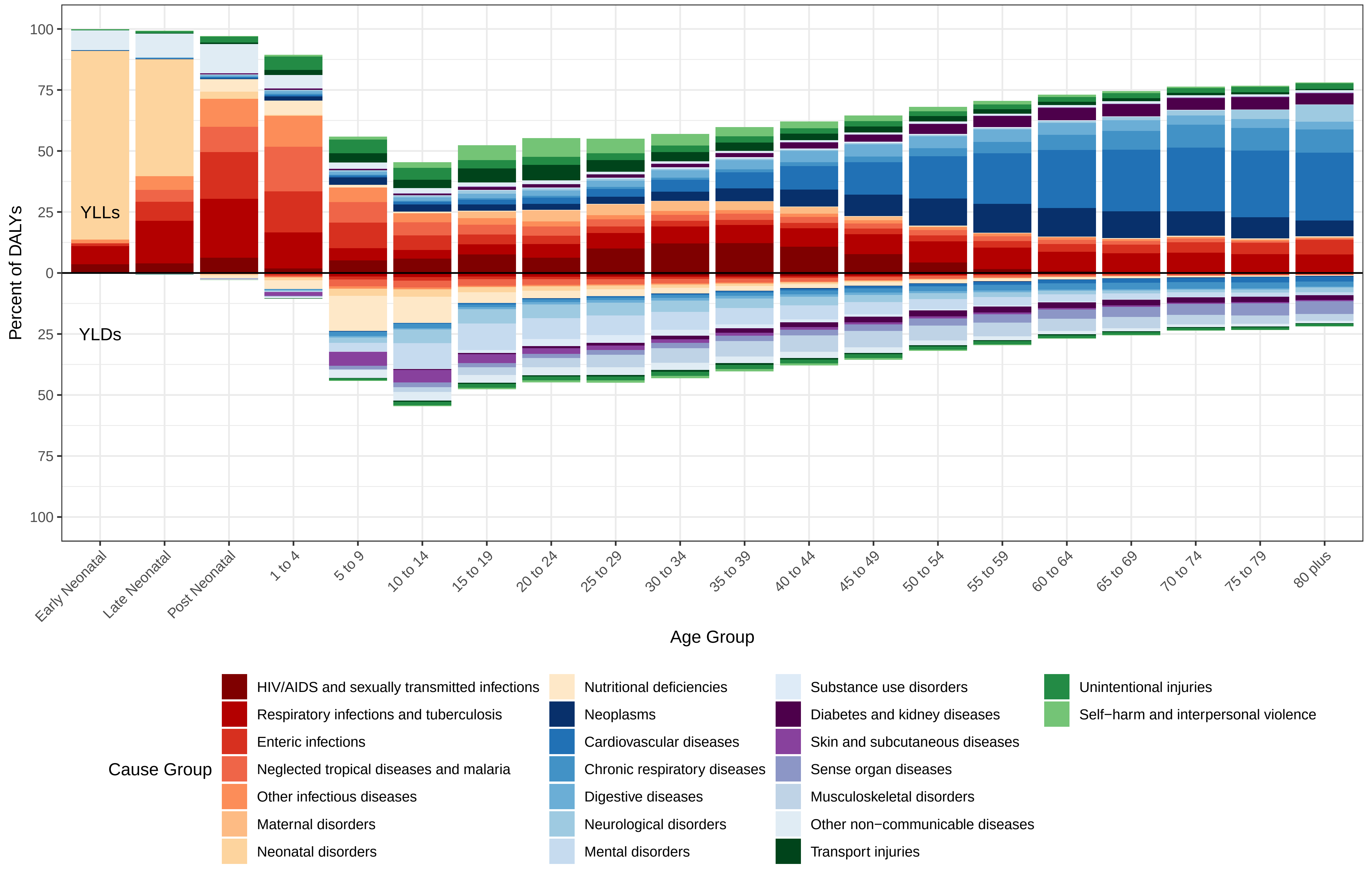

Appendix Figure 7e. Age−specific Percent of DALYs by Cause, Country Aggregation

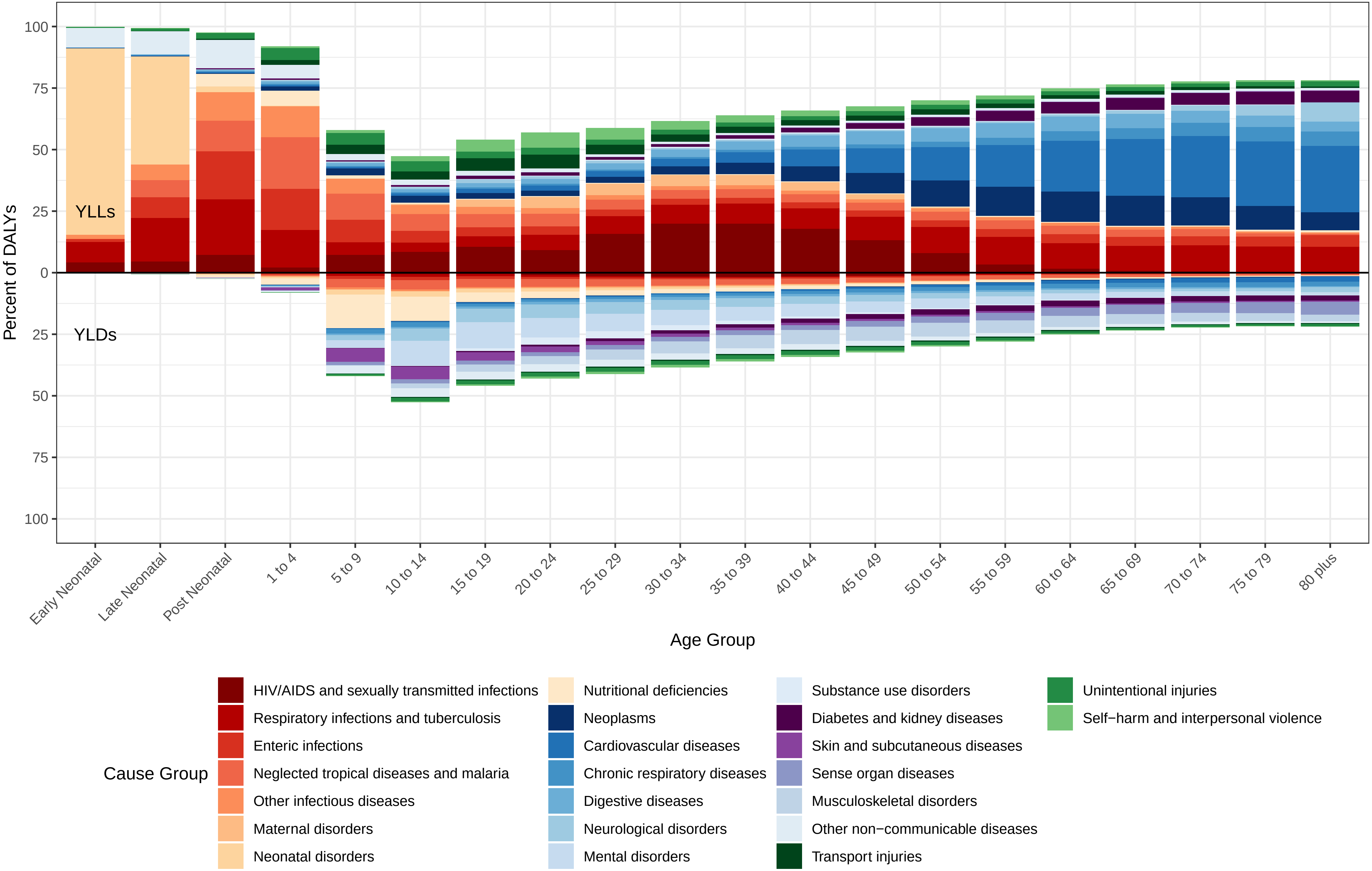

Appendix Figure 7f. Age−specific Percent of DALYs by Cause, Low−income Countries

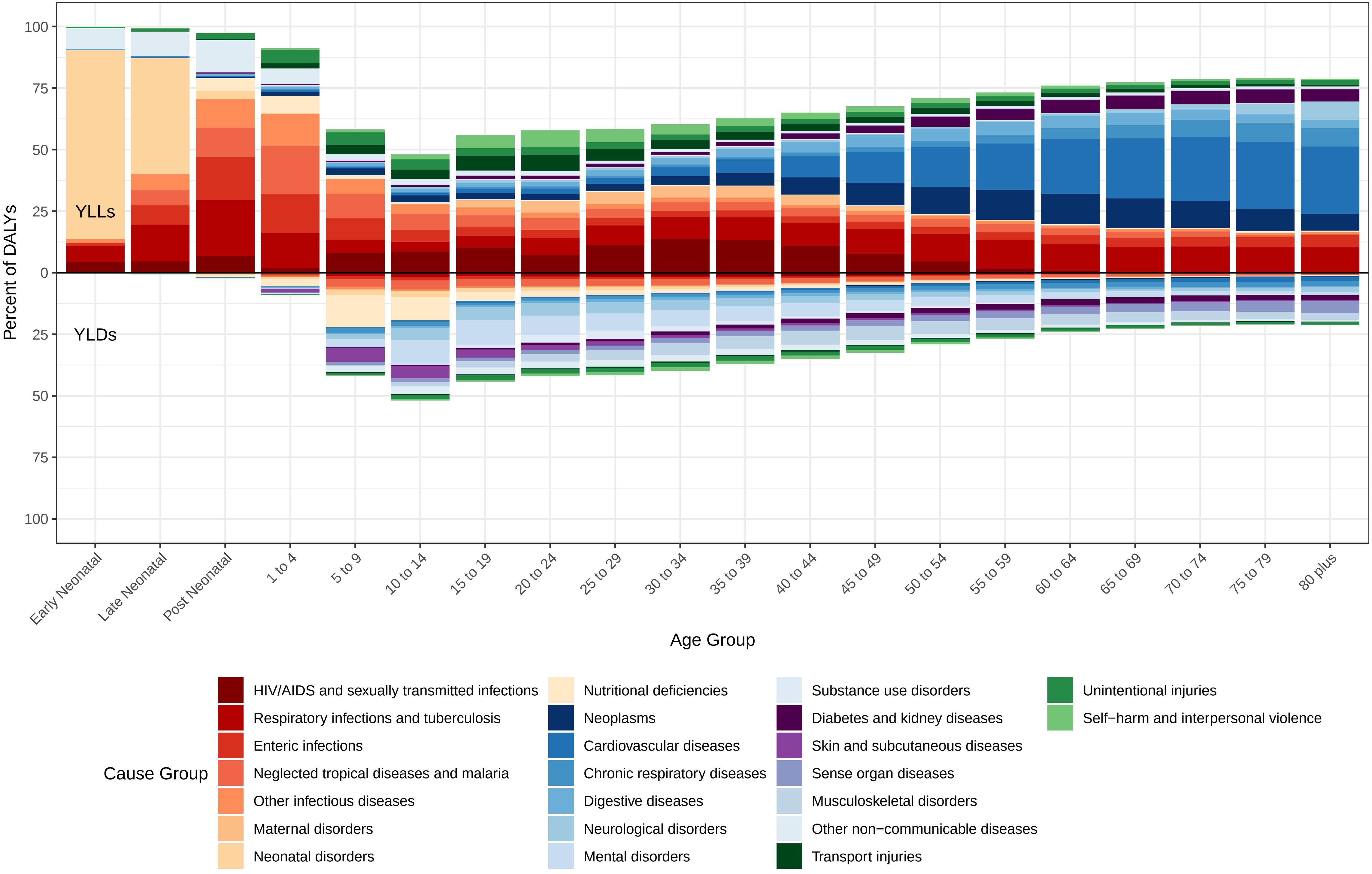

Appendix Figure 7g. Age−specific Percent of DALYs by Cause, Lower−middle−income Countries

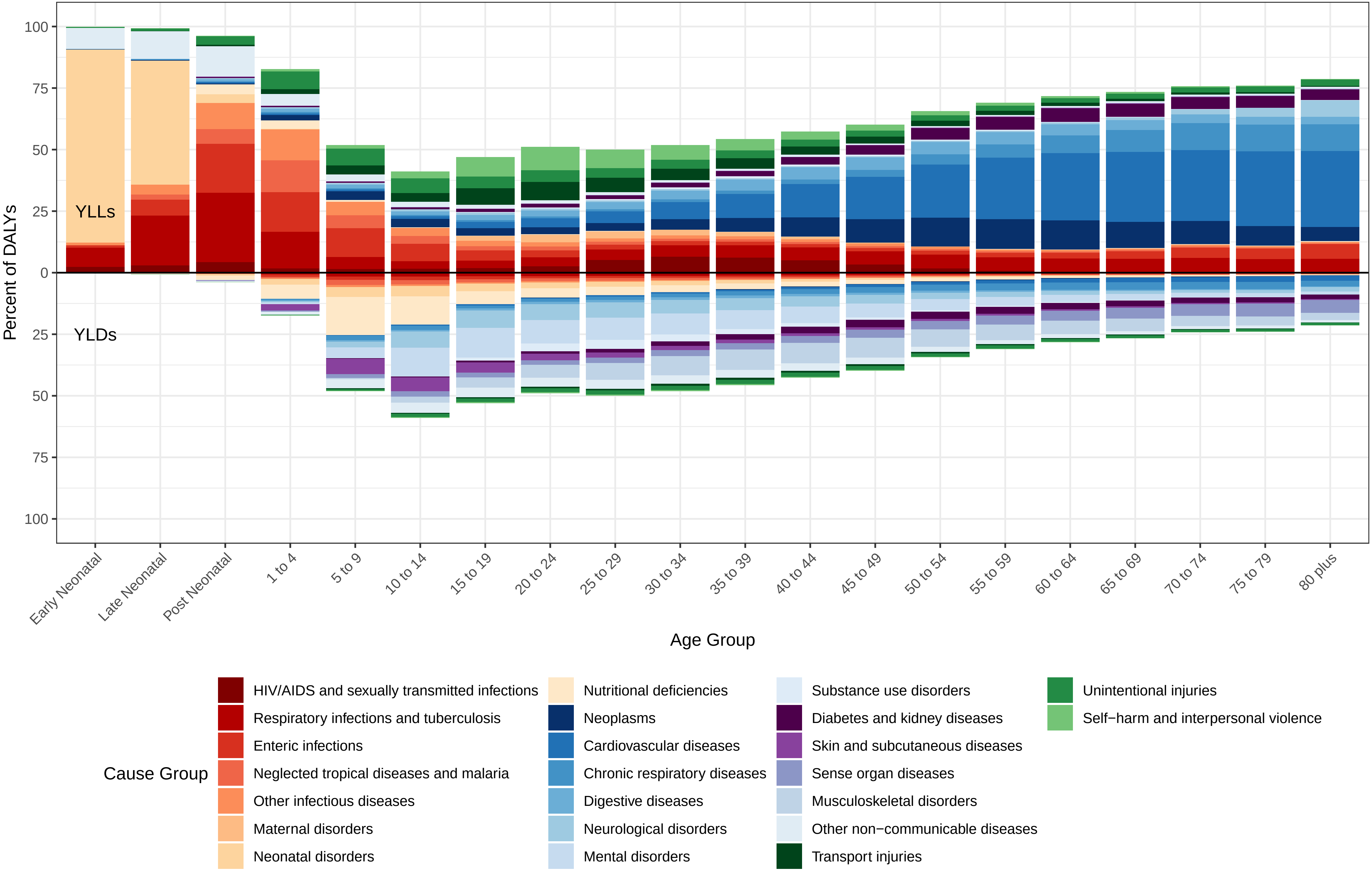

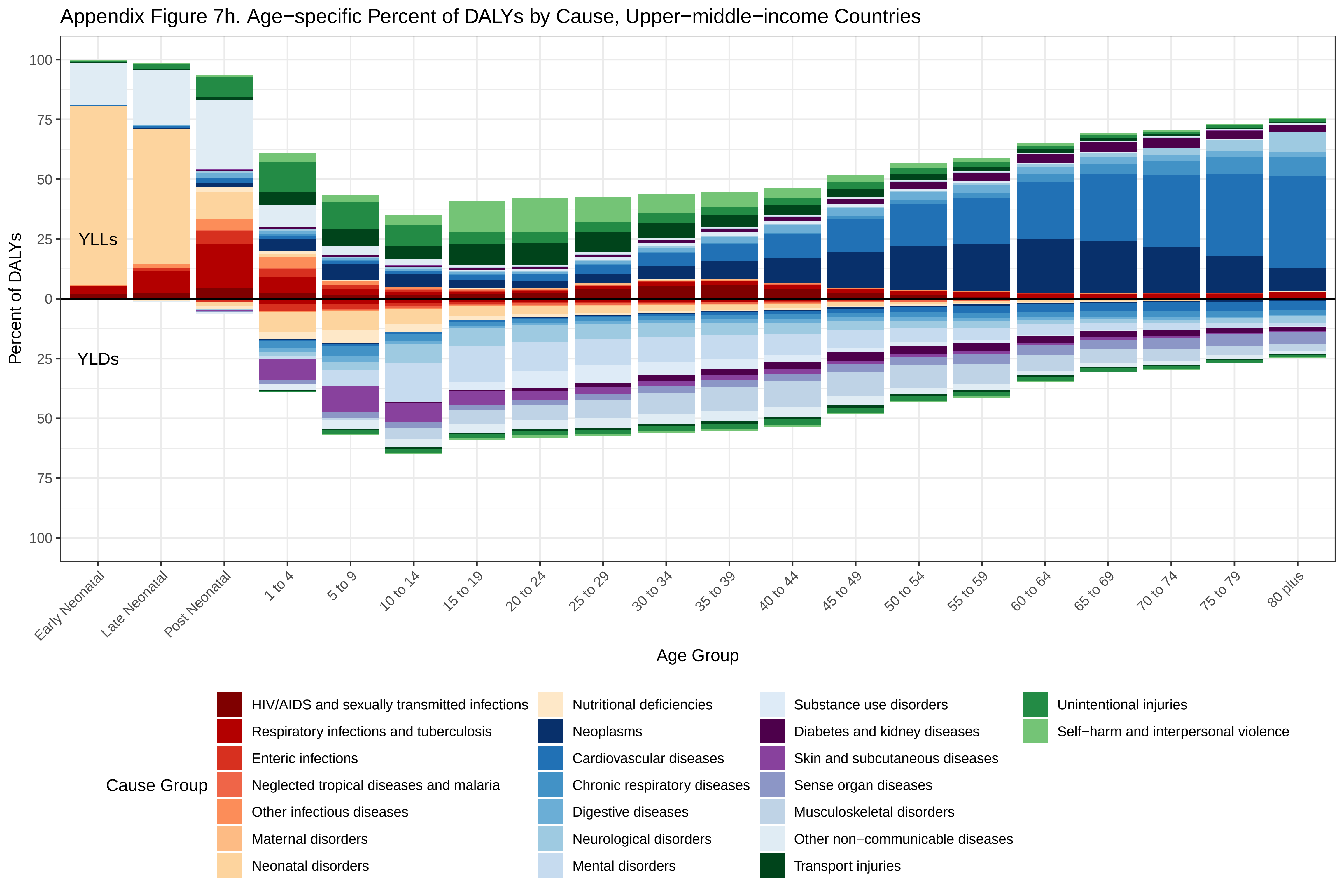
Appendix Figure 7h. Age−specific Percent of DALYs by Cause, Upper−middle−income Countries
Percent of DALYs
YLLs
YLDs
100
75
50
25
0
25
50
75
100
Early Neonatal
Late Neonatal
Post Neonatal
1 to 4
5 to 9
10 to 14
15 to 19
20 to 24
25 to 29
30 to 34
35 to 39
40 to 44
45 to 49
50 to 54
55 to 59
60 to 64
65 to 69
70 to 74
75 to 79
80 plus
Age Group
Cause Group
HIV/AIDS and sexually transmitted infections
Respiratory infections and tuberculosis
Enteric infections
Neglected tropical diseases and malaria
Other infectious diseases
Maternal disorders
Neonatal disorders
Nutritional deficiencies
Neoplasms
Cardiovascular diseases
Chronic respiratory diseases
Digestive diseases
Neurological disorders
Mental disorders
Substance use disorders
Diabetes and kidney diseases
Skin and subcutaneous diseases
Sense organ diseases
Musculoskeletal disorders
Other non−communicable diseases
Transport injuries
Unintentional injuries
Self−harm and interpersonal violence

Appendix Figure 7i. Age−specific Percent of DALYs by Cause, High−income Countries

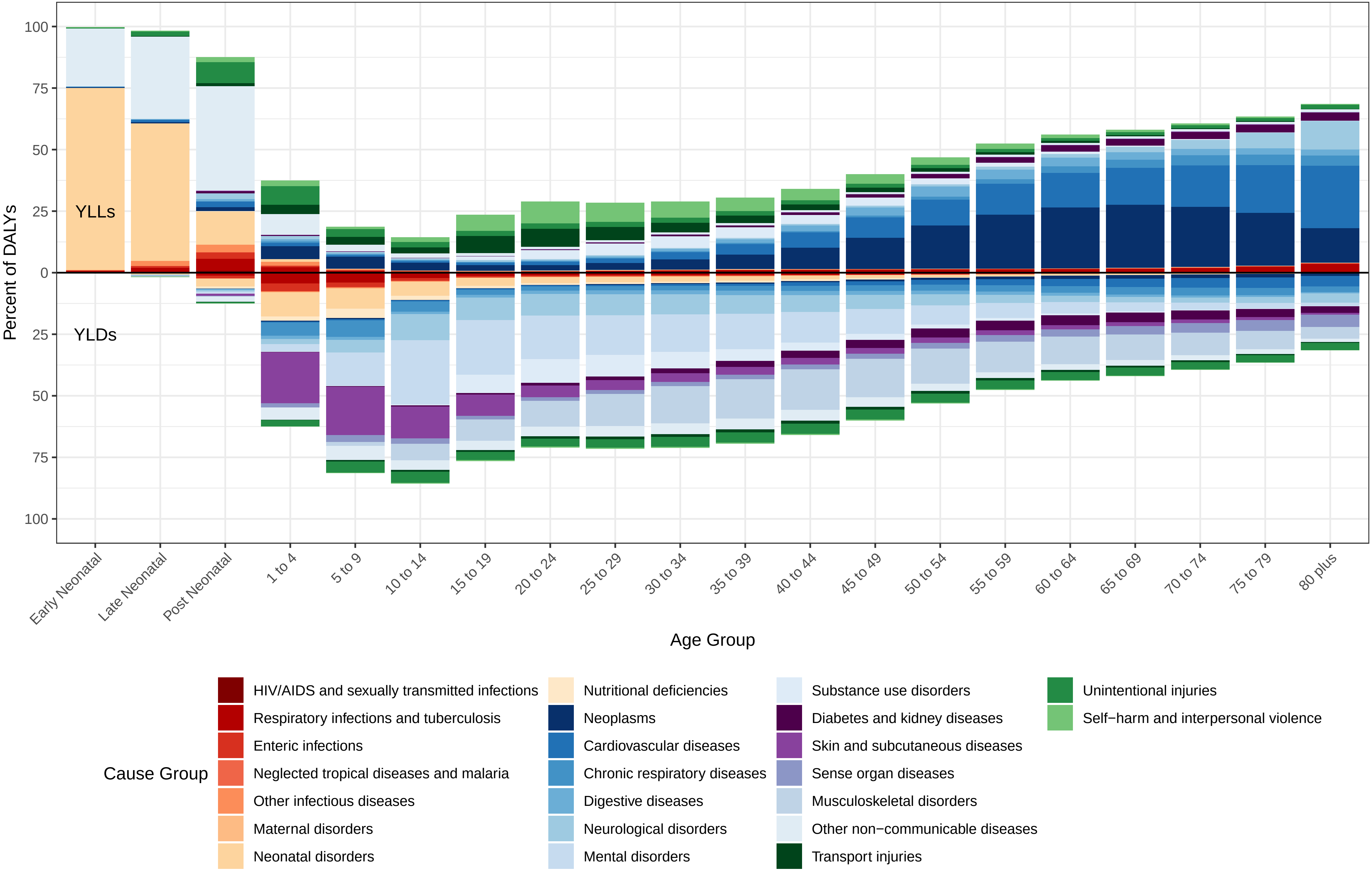

Appendix Figure 8a. Difference between Crude DALY Rates in Poorest Billion and High−income Regions, Selective Ecological Approach

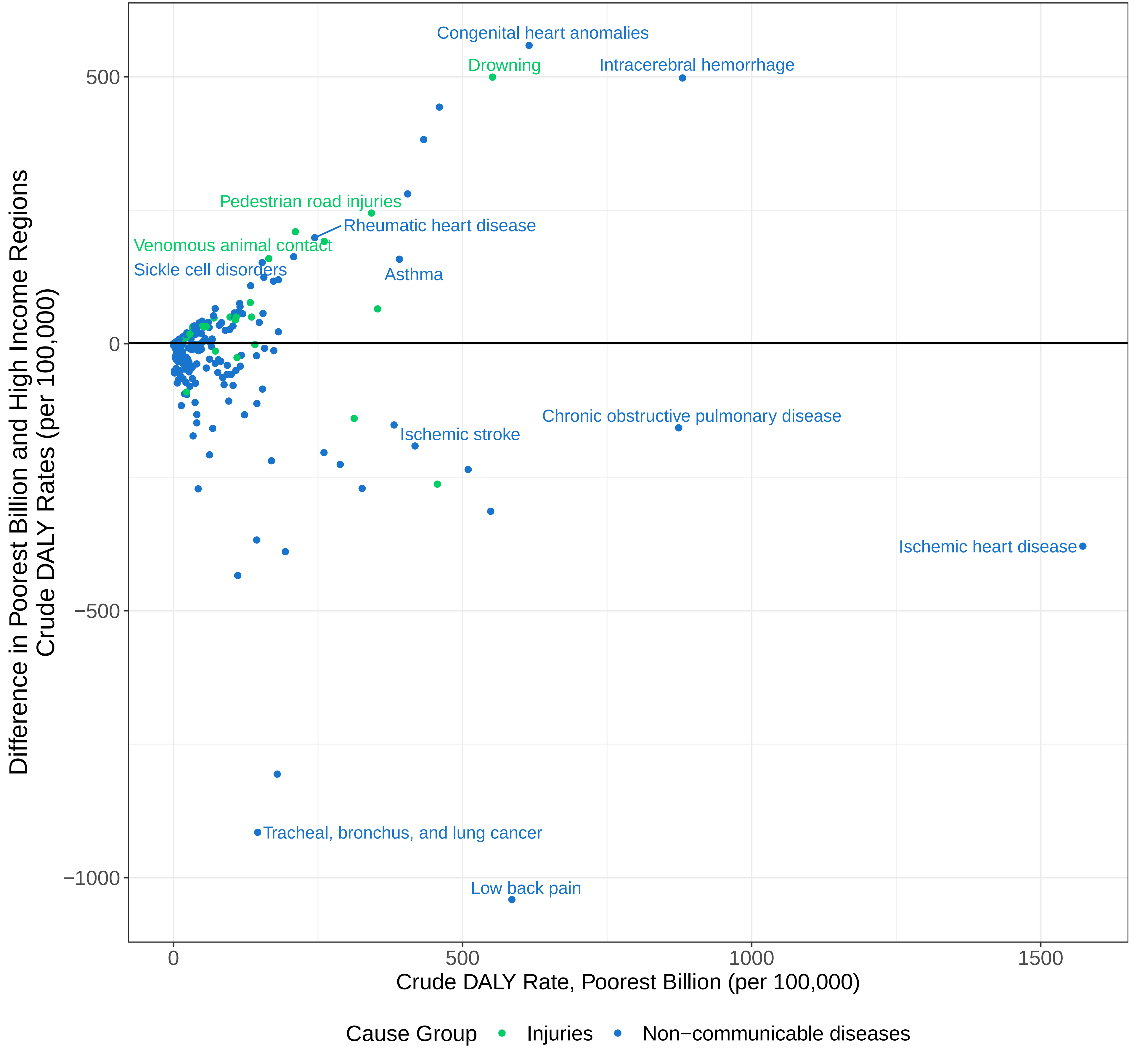

Appendix Figure 8b. Difference between Age−standardized DALY Rates in Poorest Billion and High−income Regions, Selective Ecological Approach

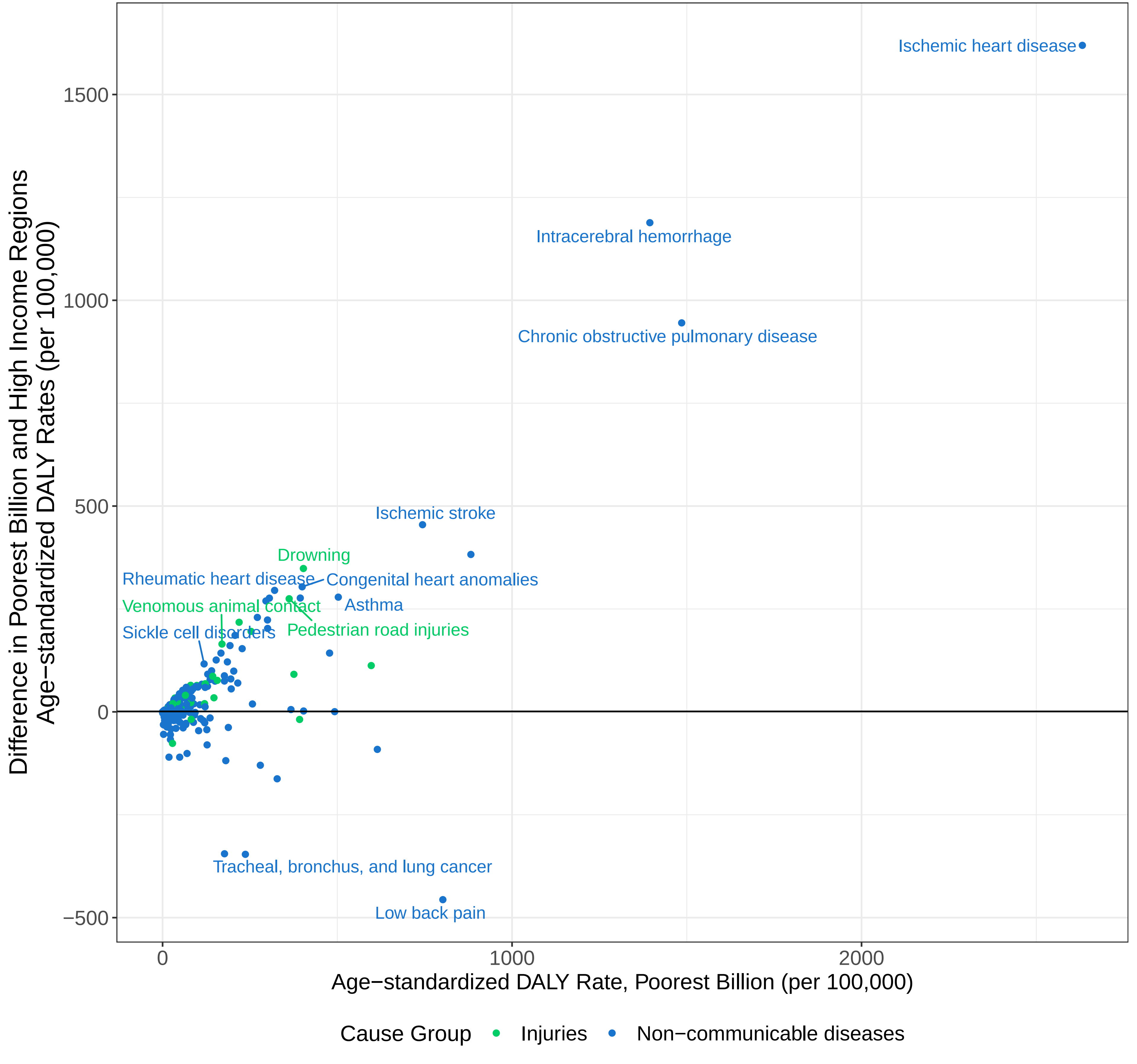

Appendix Figure 8c. Difference between Crude DALY Rates in Poorest Billion and High−income Regions, Full Ecological Approach

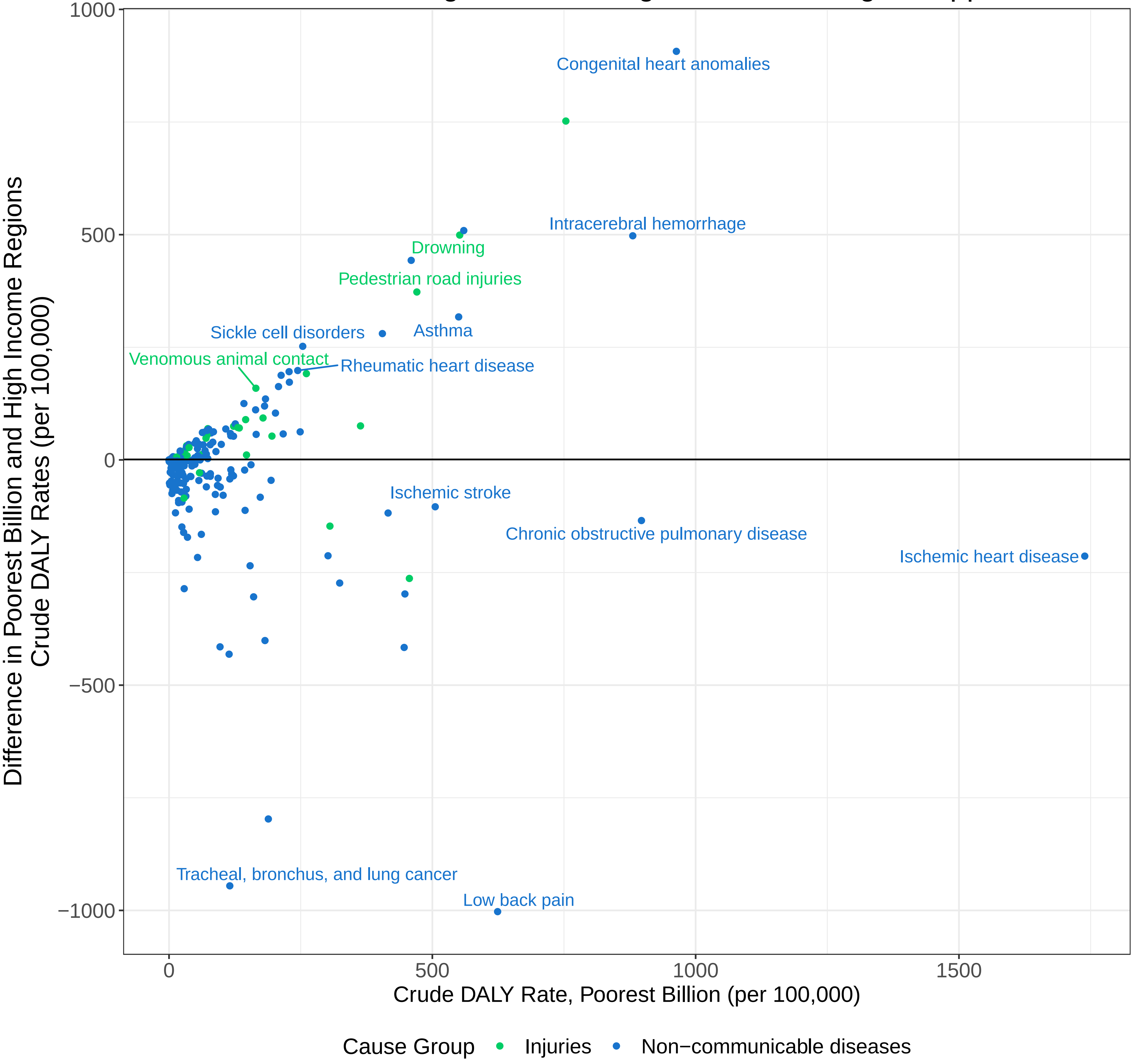

Appendix Figure 8d. Difference between Age−standardized DALY Rates in Poorest Billion and High−income Regions, Full Ecological Approach

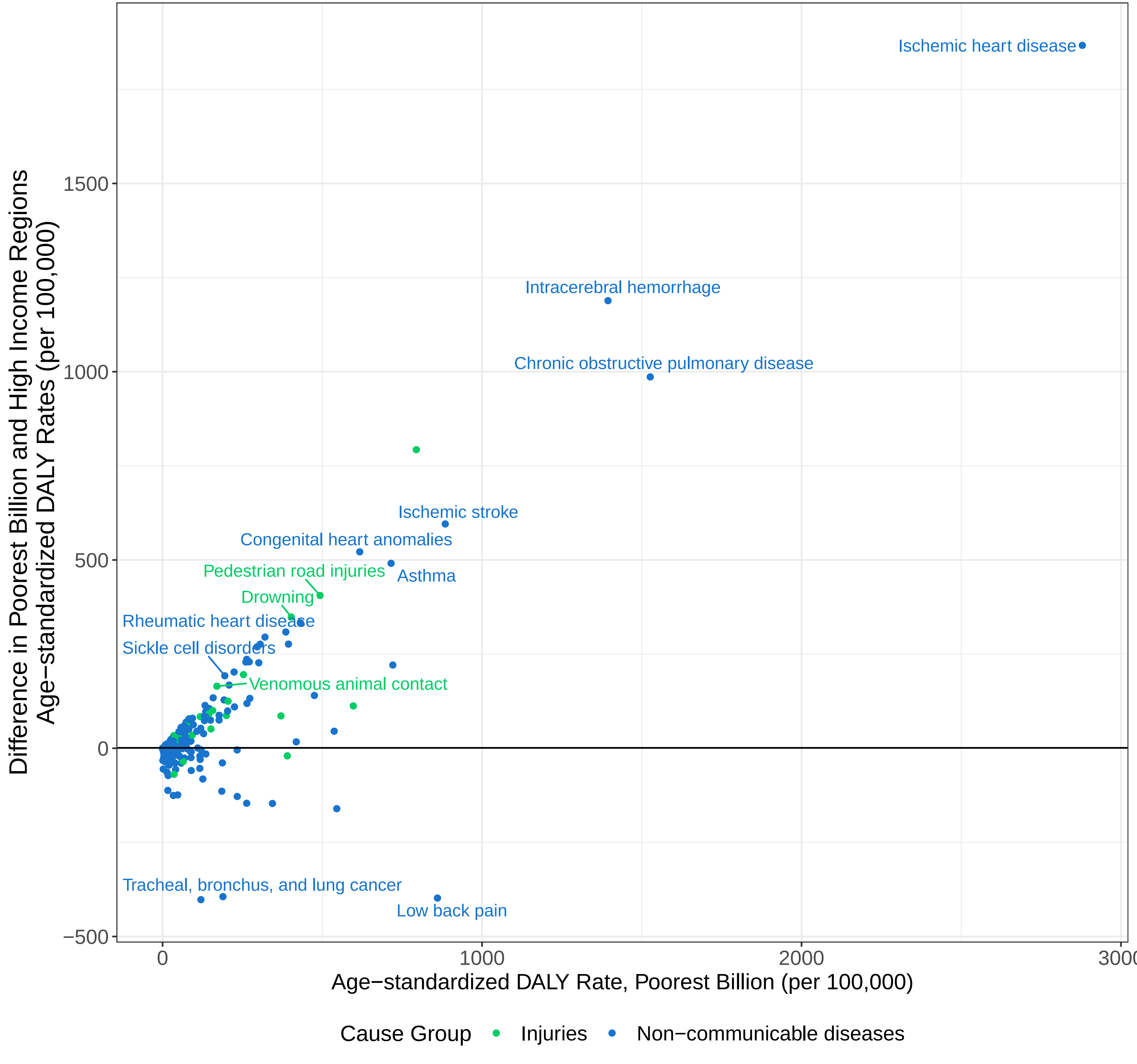

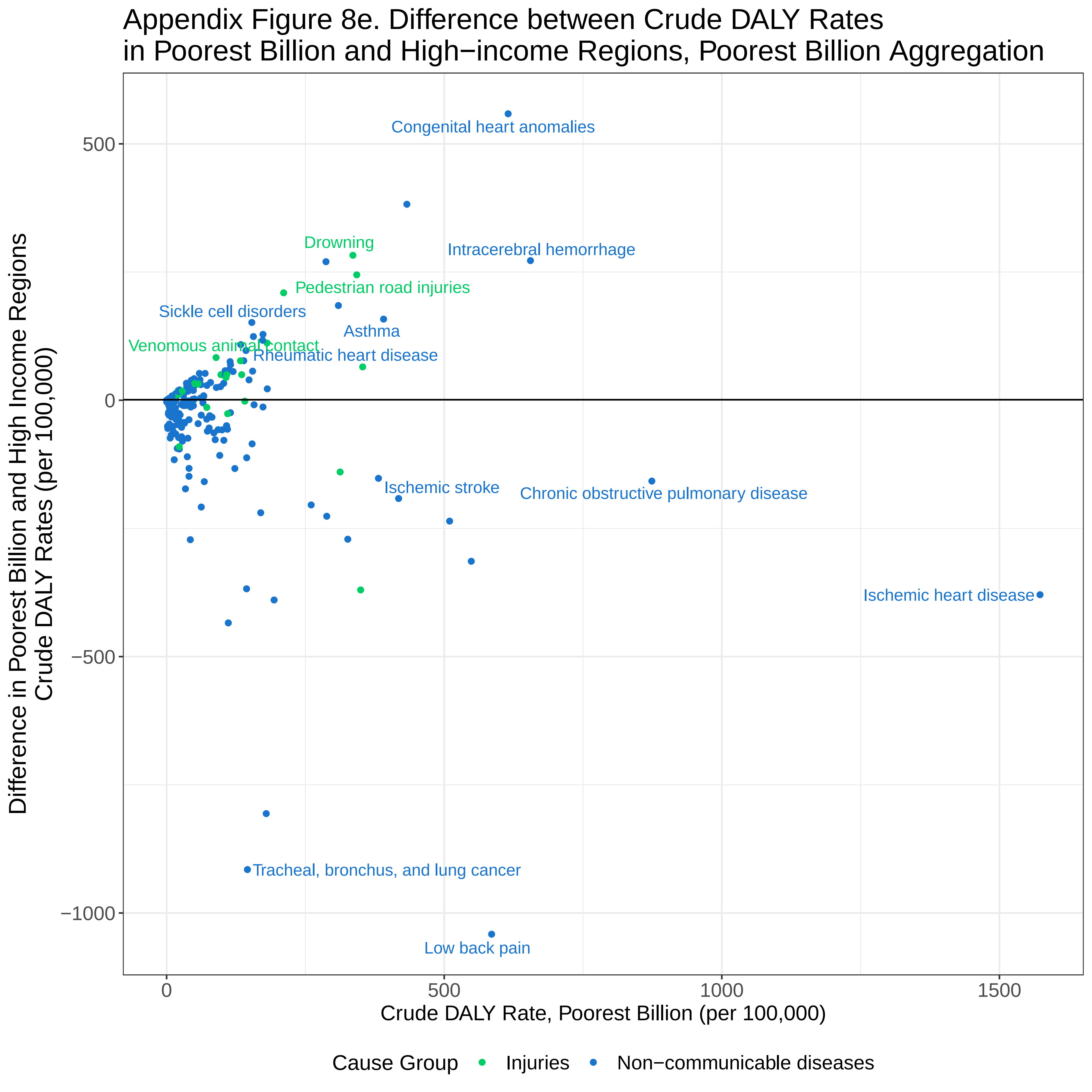

Appendix Figure 8e. Difference between Crude DALY Rates
in Poorest Billion and High−income Regions, Poorest Billion Aggregation
Congenital heart anomalies
Drowning
Intracerebral hemorrhage
Pedestrian road injuries
Sickle cell disorders
Asthma
Venomous animal contact
Rheumatic heart disease
Ischemic stroke
Chronic obstructive pulmonary disease
Ischemic heart disease
Tracheal, bronchus, and lung cancer
Low back pain
500
0
−500
−1000
0
500
1000
1500
Difference in Poorest Billion and High Income Regions
Crude DALY Rates (per 100,000)
Crude DALY Rate, Poorest Billion (per 100,000)
Cause Group
Injuries
Non−communicable diseases

Appendix Figure 8f. Difference between Age−standardized DALY Rates in Poorest Billion and High−income Regions, Poorest Billion Aggregation

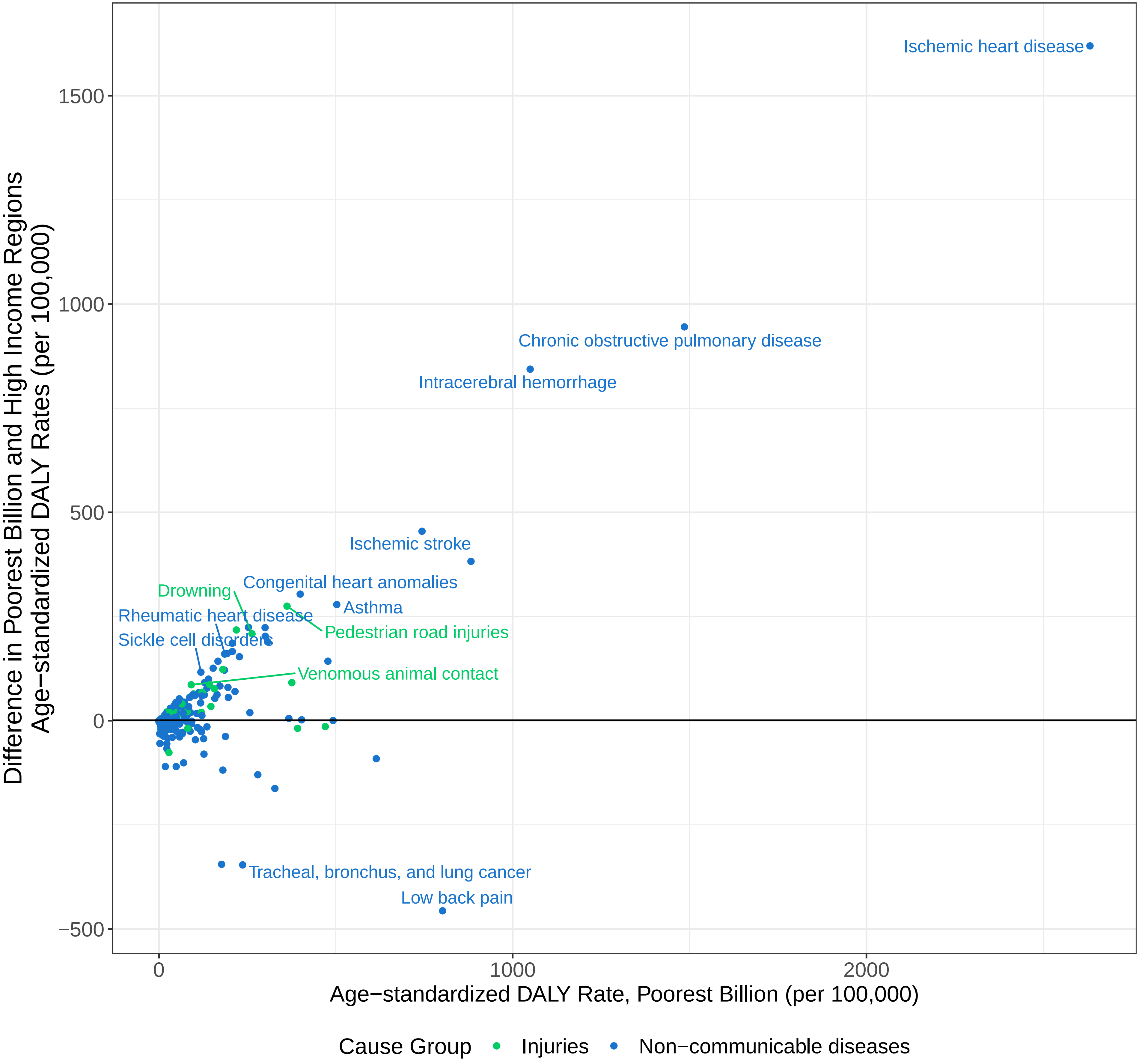

Appendix Figure 8g. Difference between Crude DALY Rates in Poorest Billion and High−income Regions, Country Aggregation

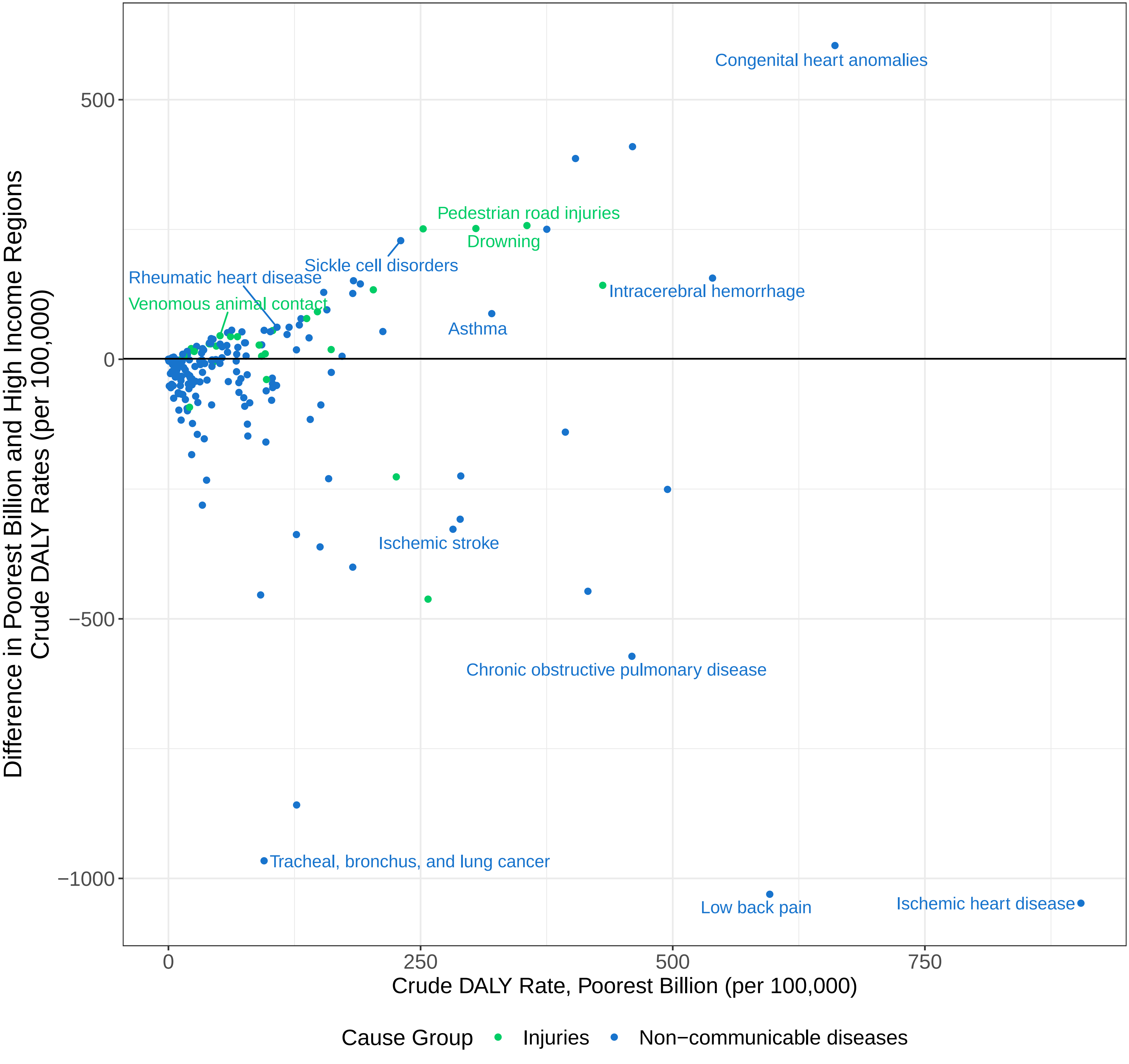

Appendix Figure 8h. Difference between Age−standardized DALY Rates in Poorest Billion and High−income Regions, Country Aggregation

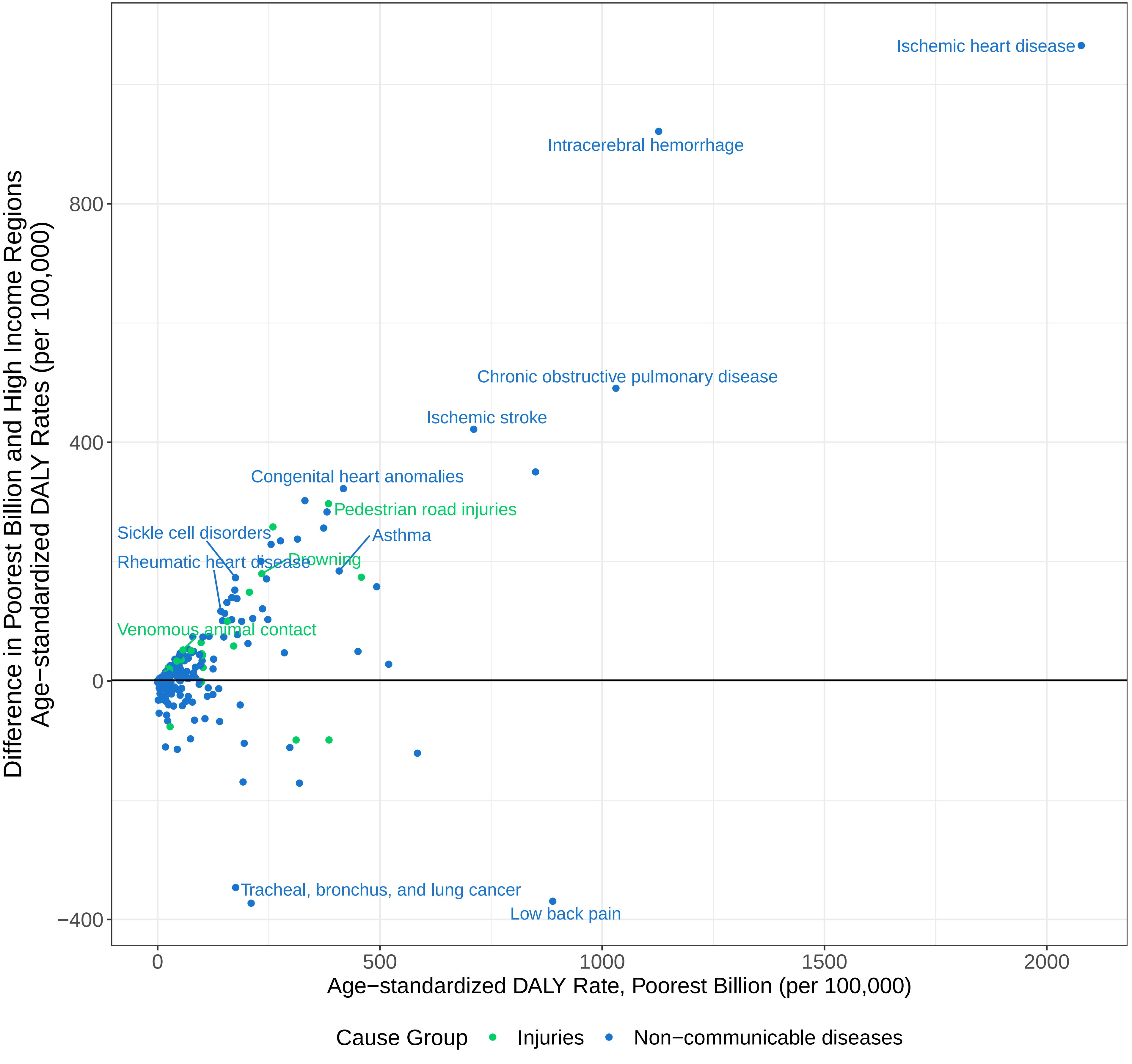

Appendix Figure 8i. Difference between Crude DALY Rates in Low−income and High−income Regions

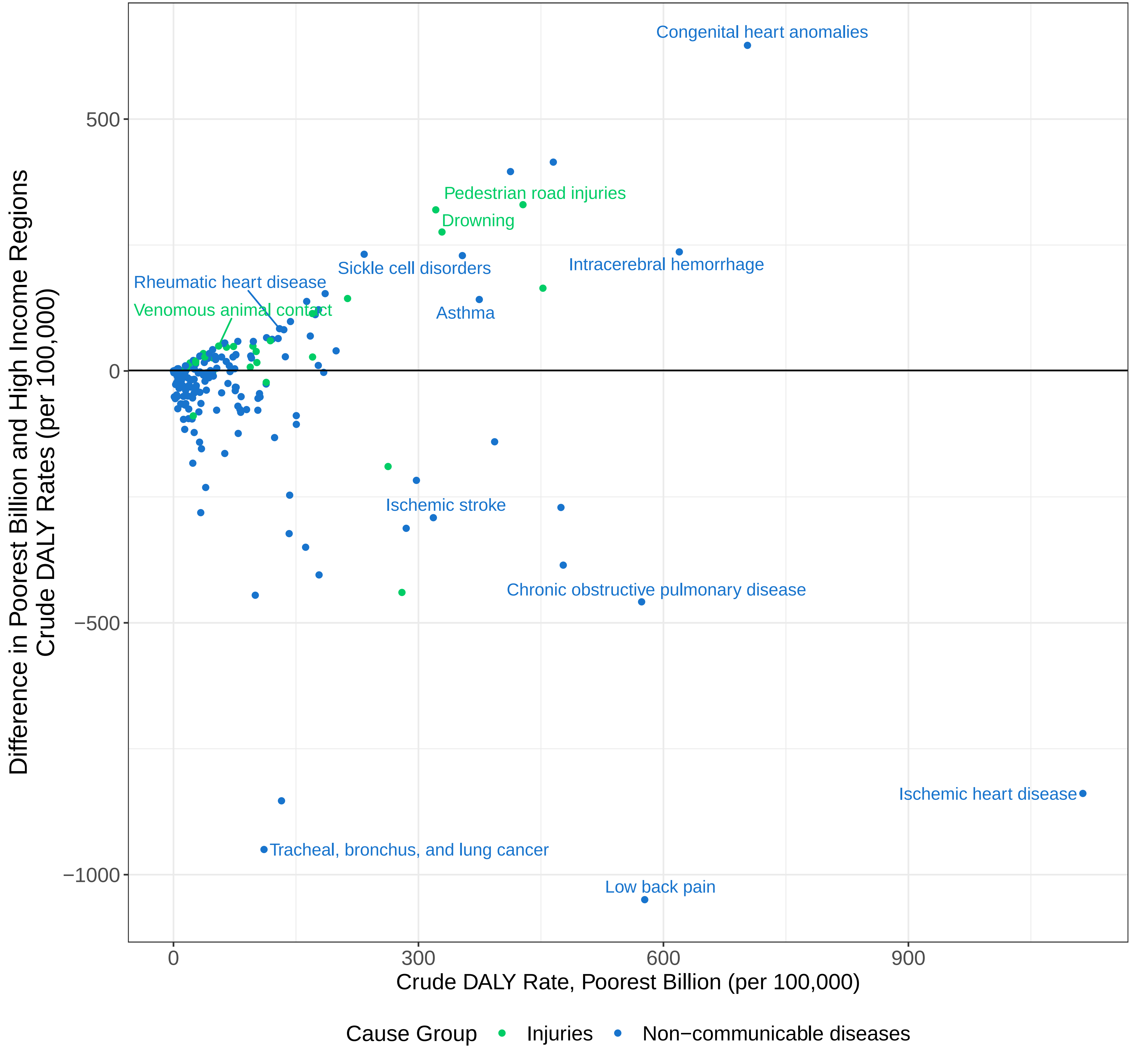

Appendix Figure 8j. Difference between Age−standardized DALY Rates in Low−income and High−income Regions

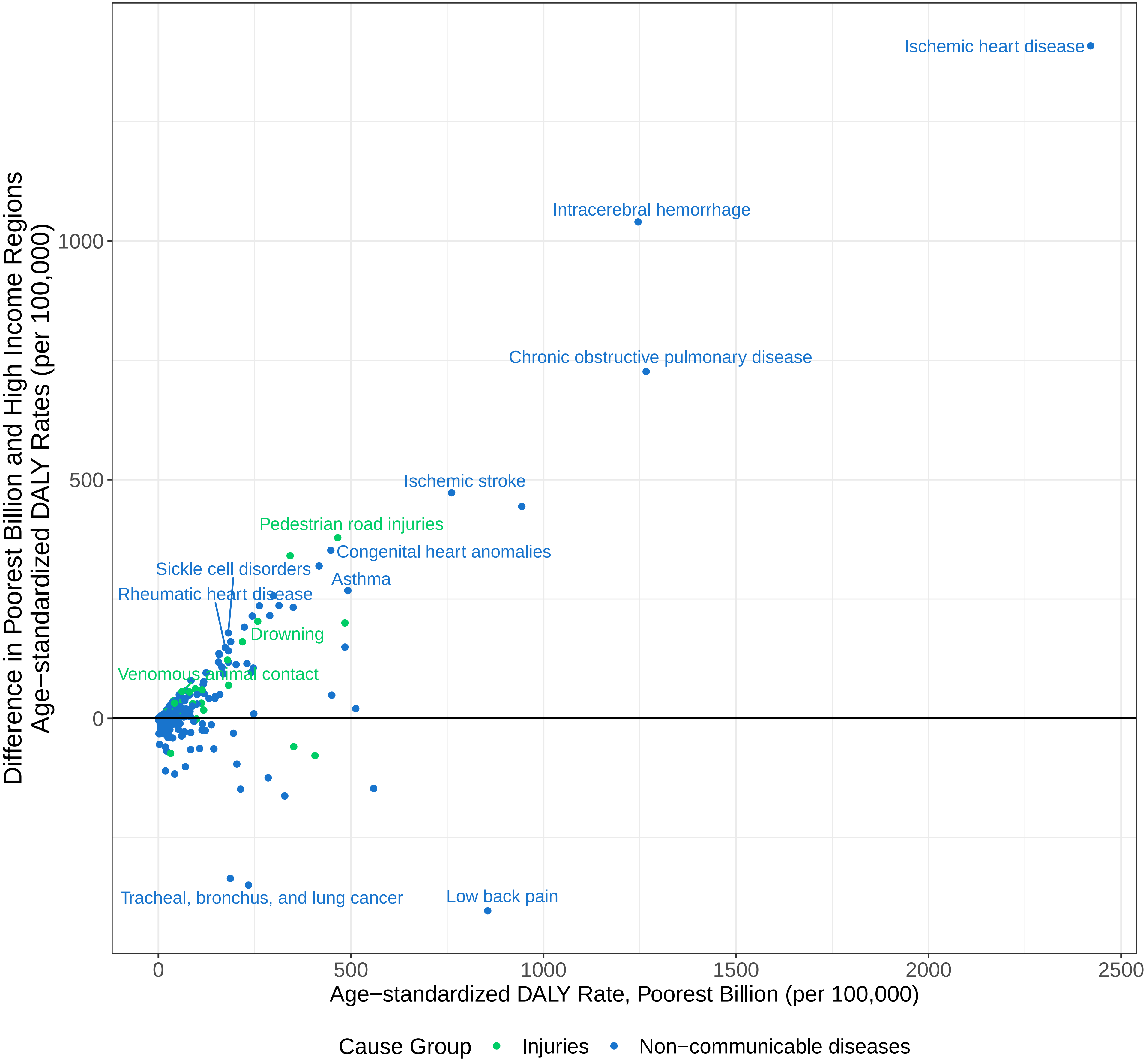

Appendix Figure 9a. Difference between Crude YLL Rates
in Poorest Billion and High−income Regions, Selective Ecological Approach

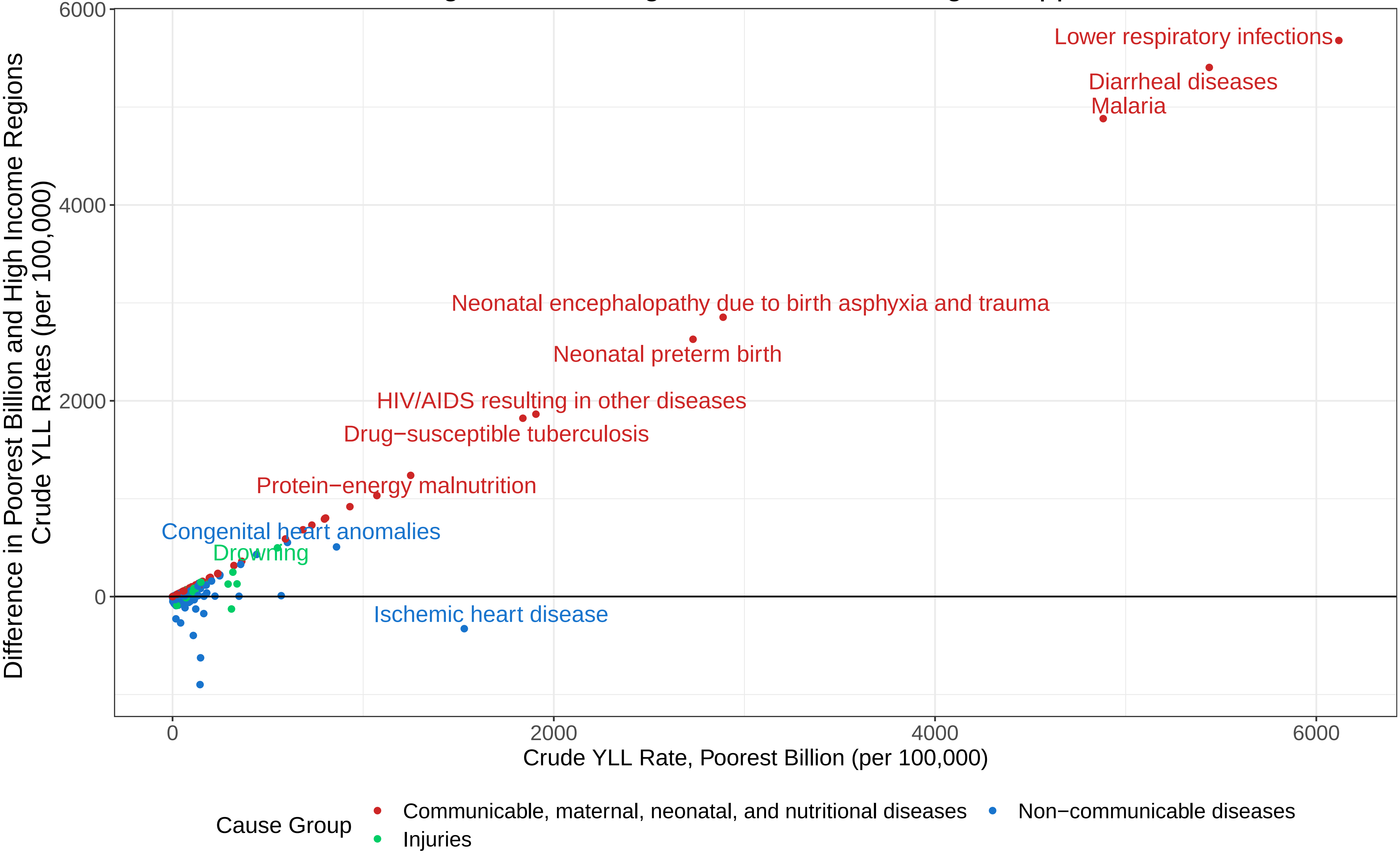

Appendix Figure 9b. Difference between Age−standardized YLL Rates in Poorest Billion and High−income Regions, Selective Ecological Approach

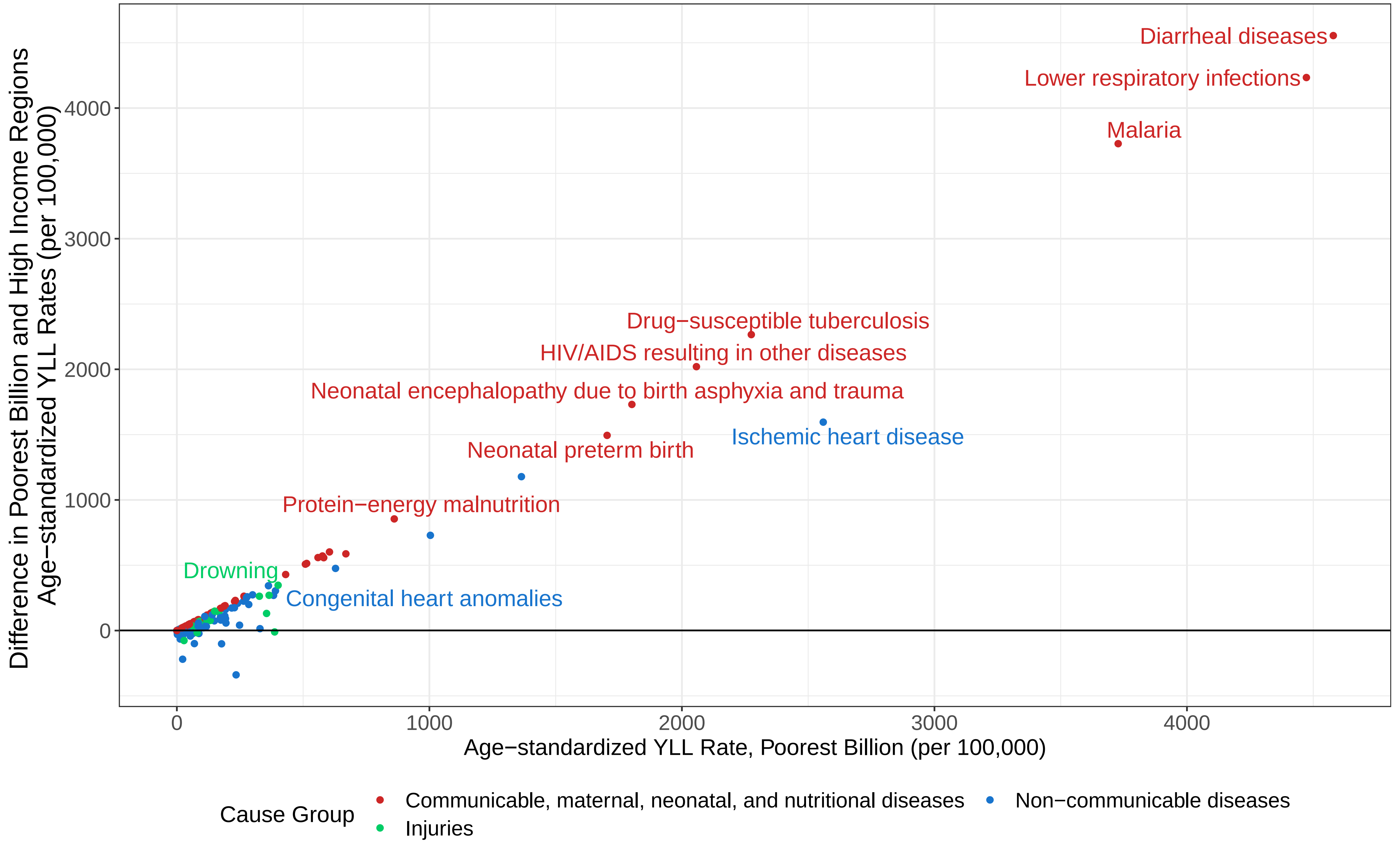

Appendix Figure 9c. Difference between Crude YLL Rates in Poorest Billion and High−income Regions, Full Ecological Approach

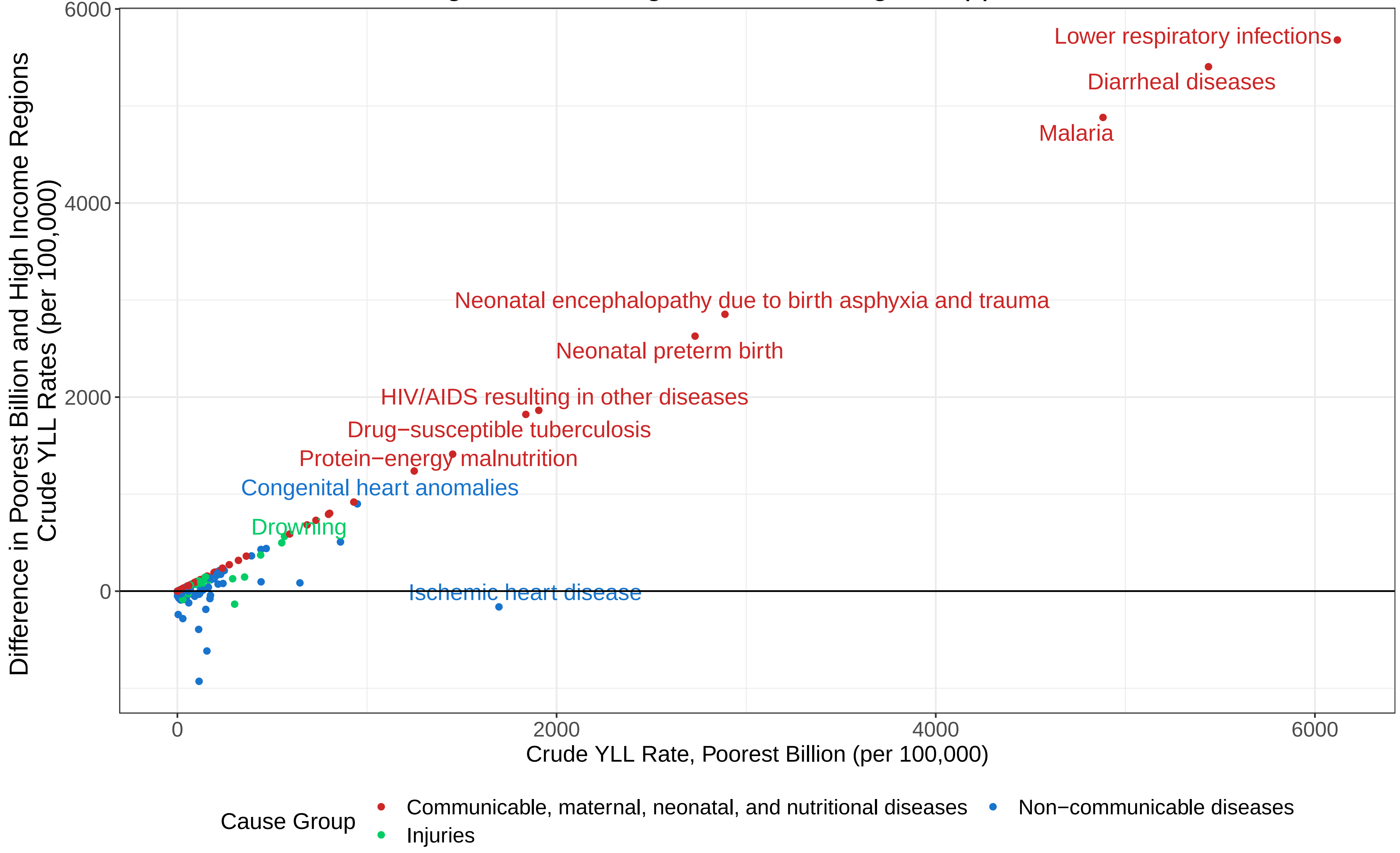

Appendix Figure 9d. Difference between Age−standardized YLL Rates in Poorest Billion and High−income Regions, Full Ecological Approach

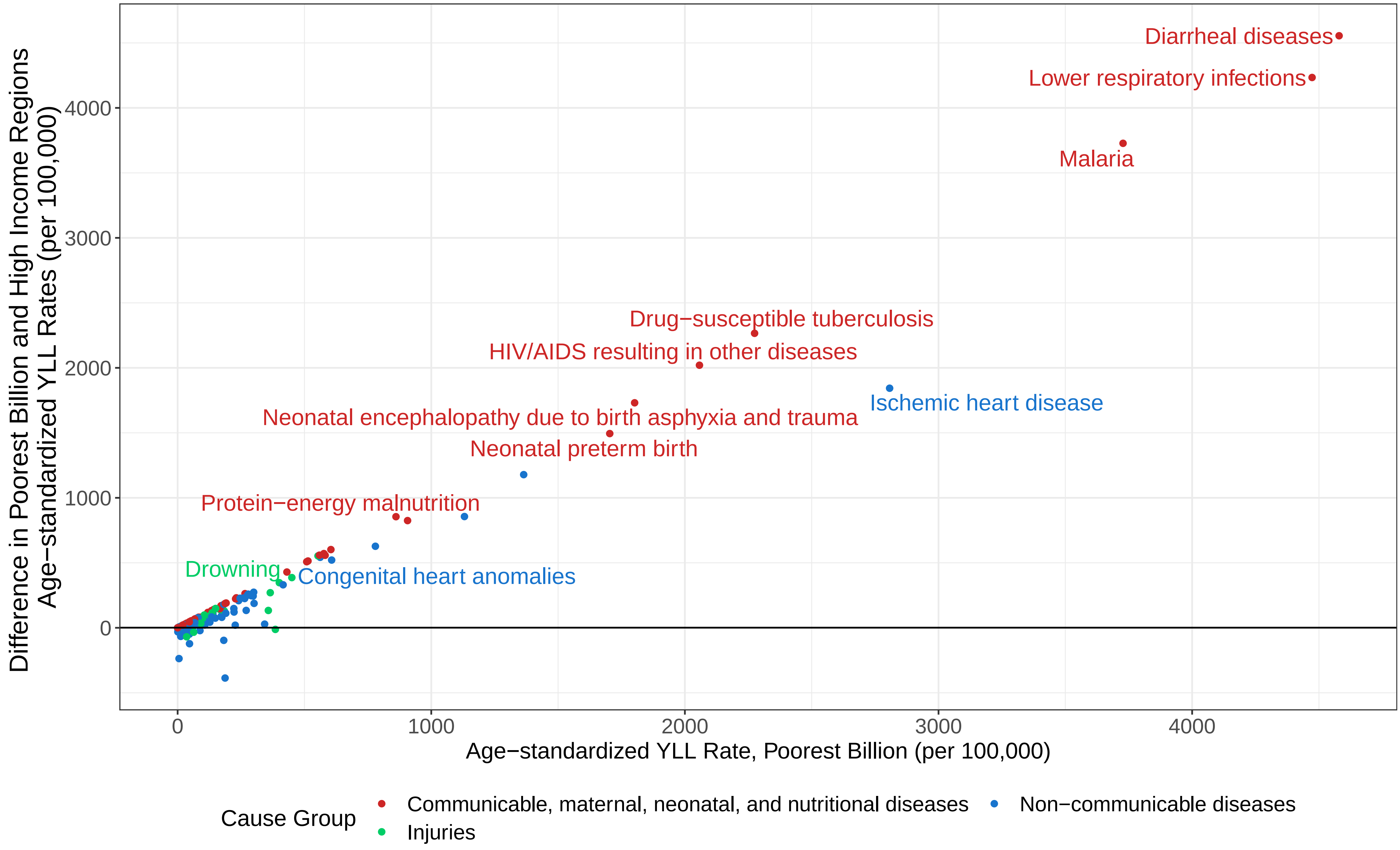

Appendix Figure 9e. Difference between Crude YLL Rates
in Poorest Billion and High−income Regions, Poorest Billion Aggregation

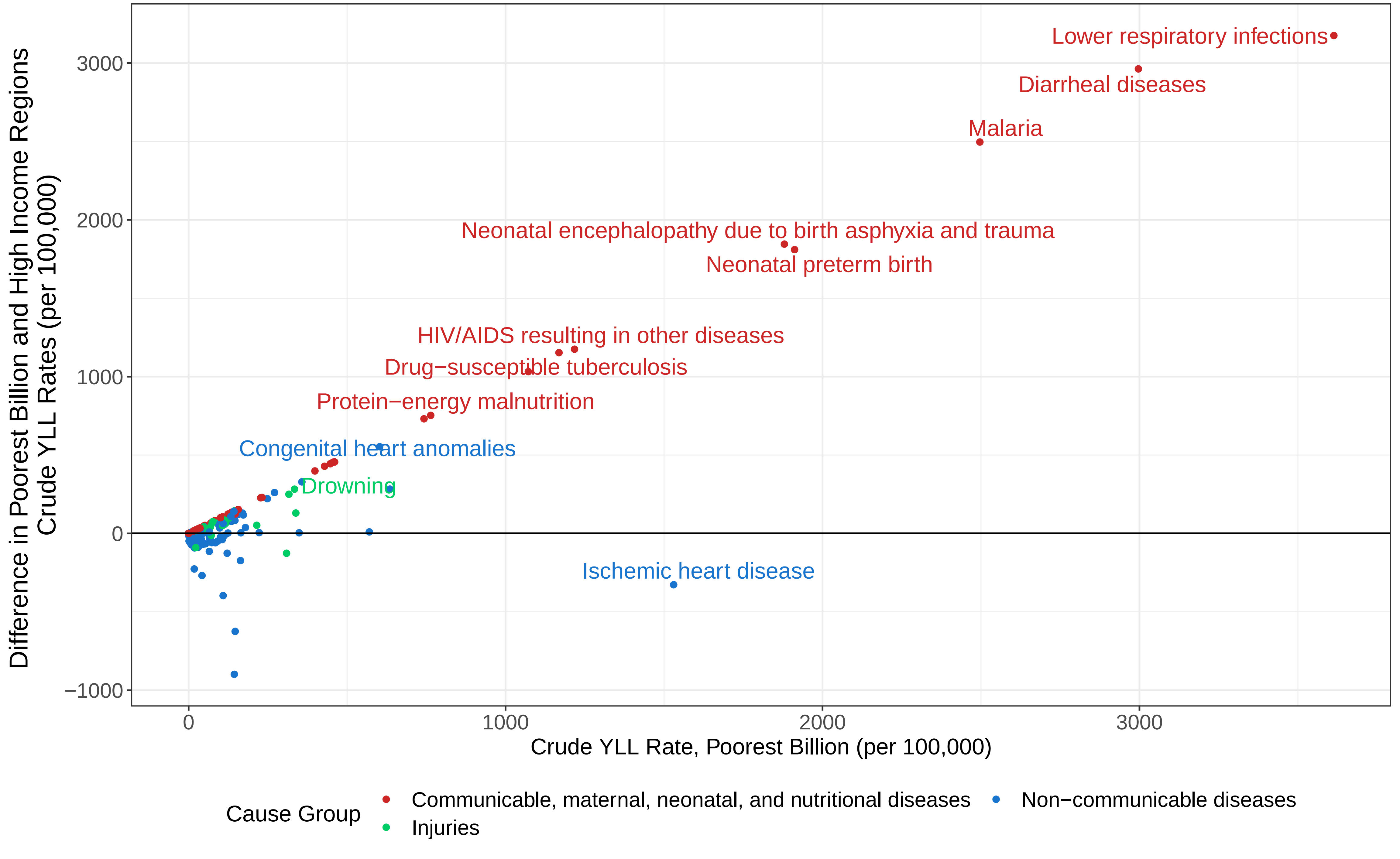

Appendix Figure 9f. Difference between Age−standardized YLL Rates in Poorest Billion and High−income Regions, Poorest Billion Aggregation

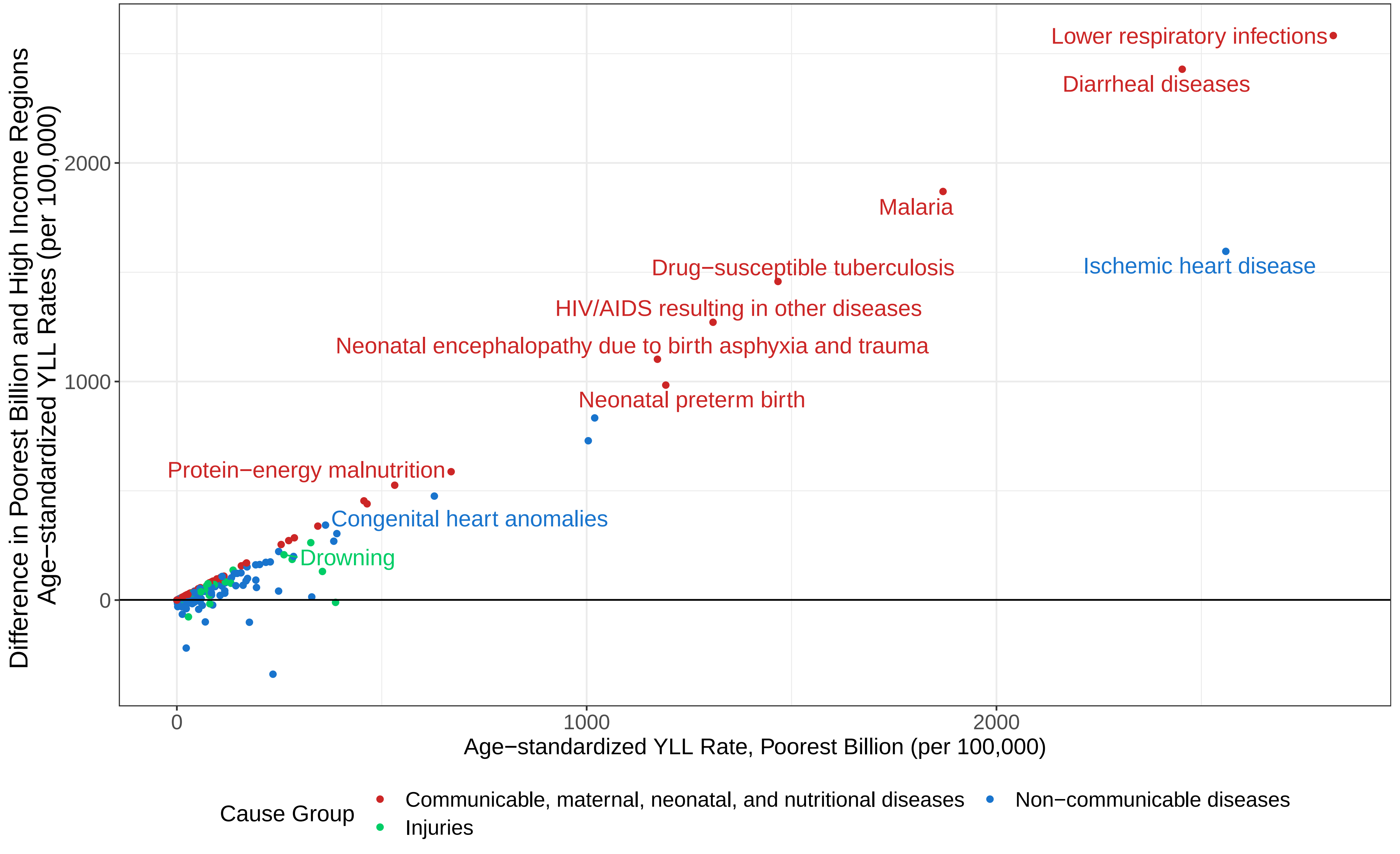

Appendix Figure 9g. Difference between Crude YLL Rates
in Poorest Billion and High−income Regions, Country Aggregation

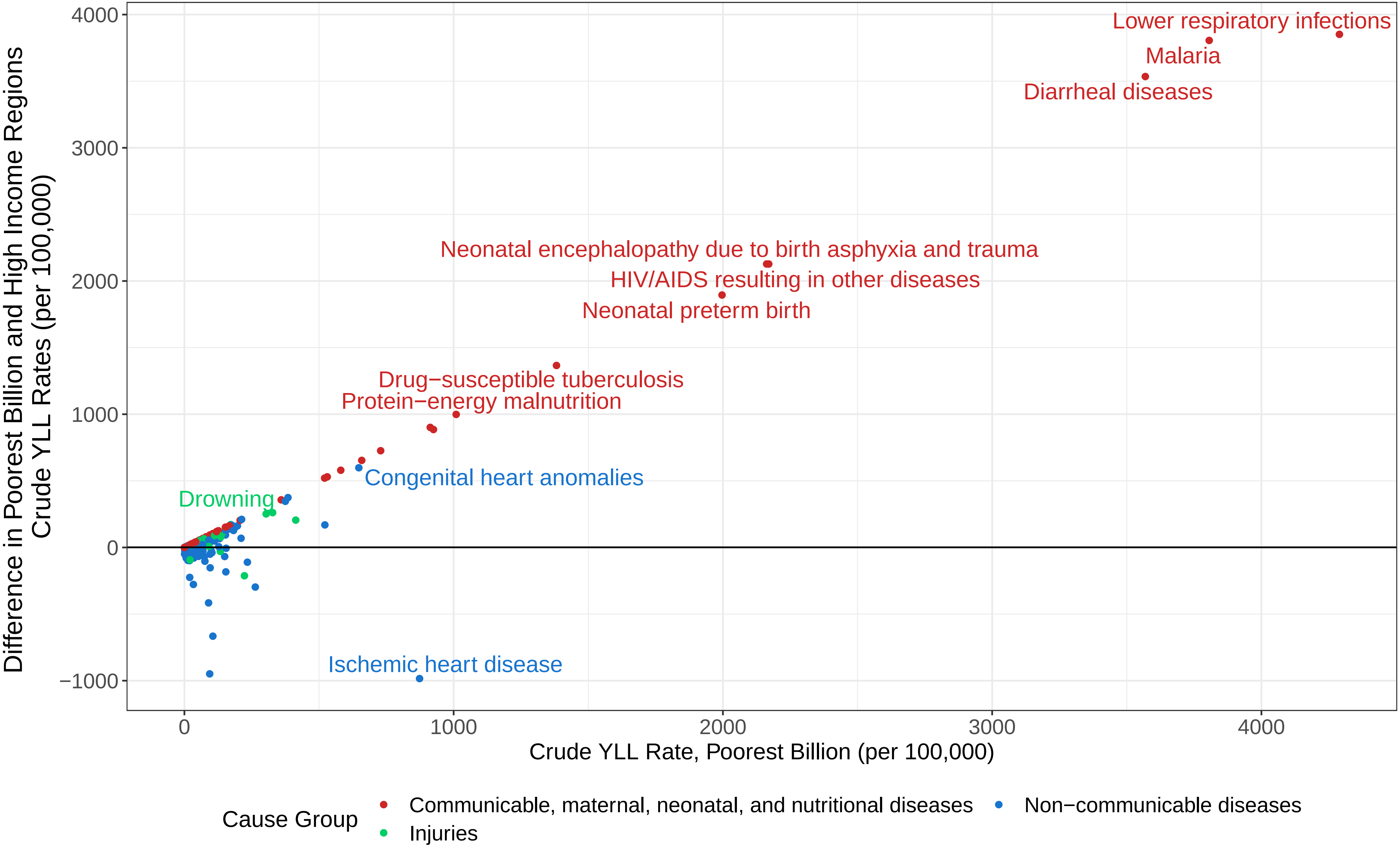

Appendix Figure 9h. Difference between Age−standardized YLL Rates in Poorest Billion and High−income Regions, Country Aggregation

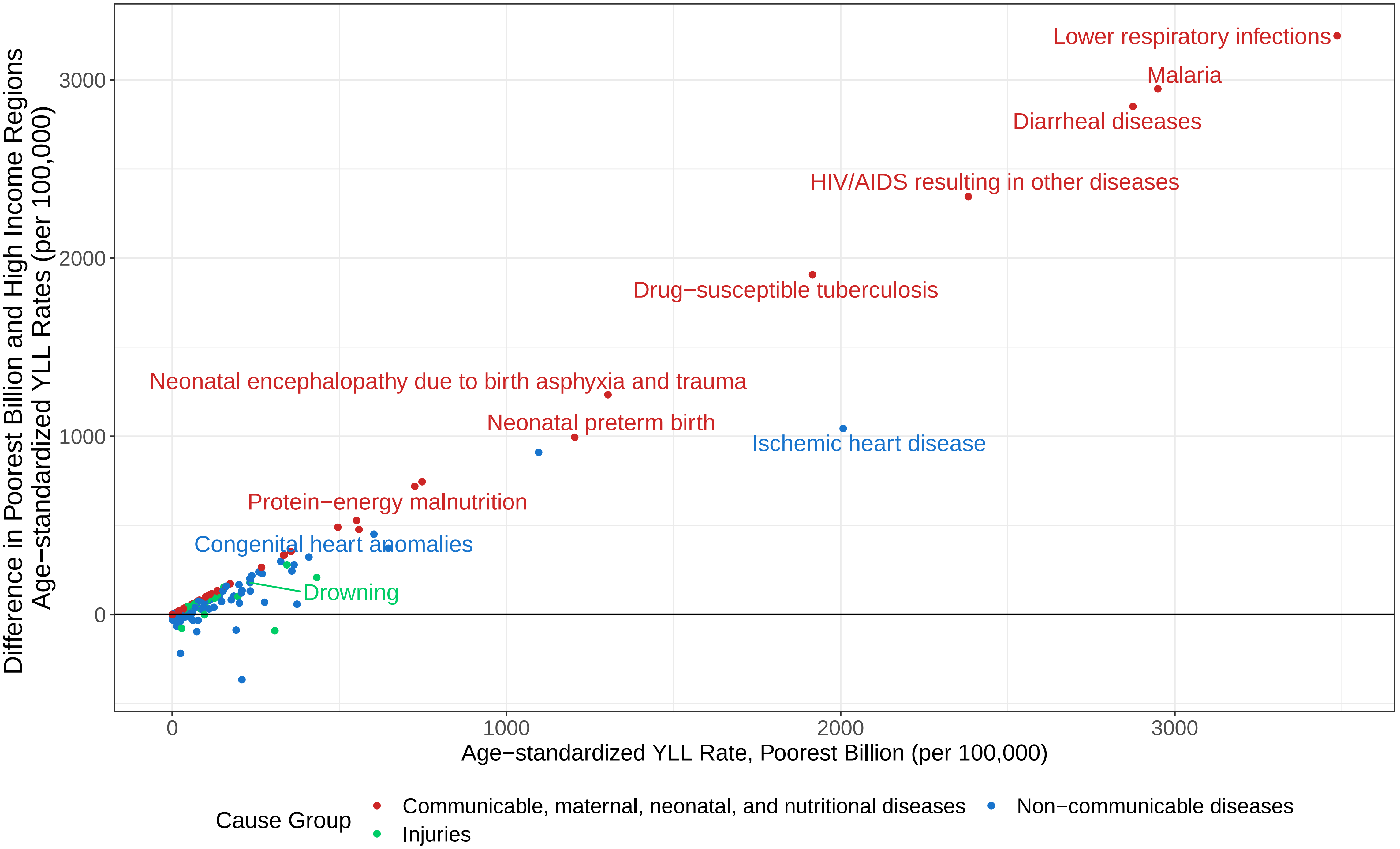

Appendix Figure 9i. Difference between Crude YLL Rates in Low−income and High−income Regions

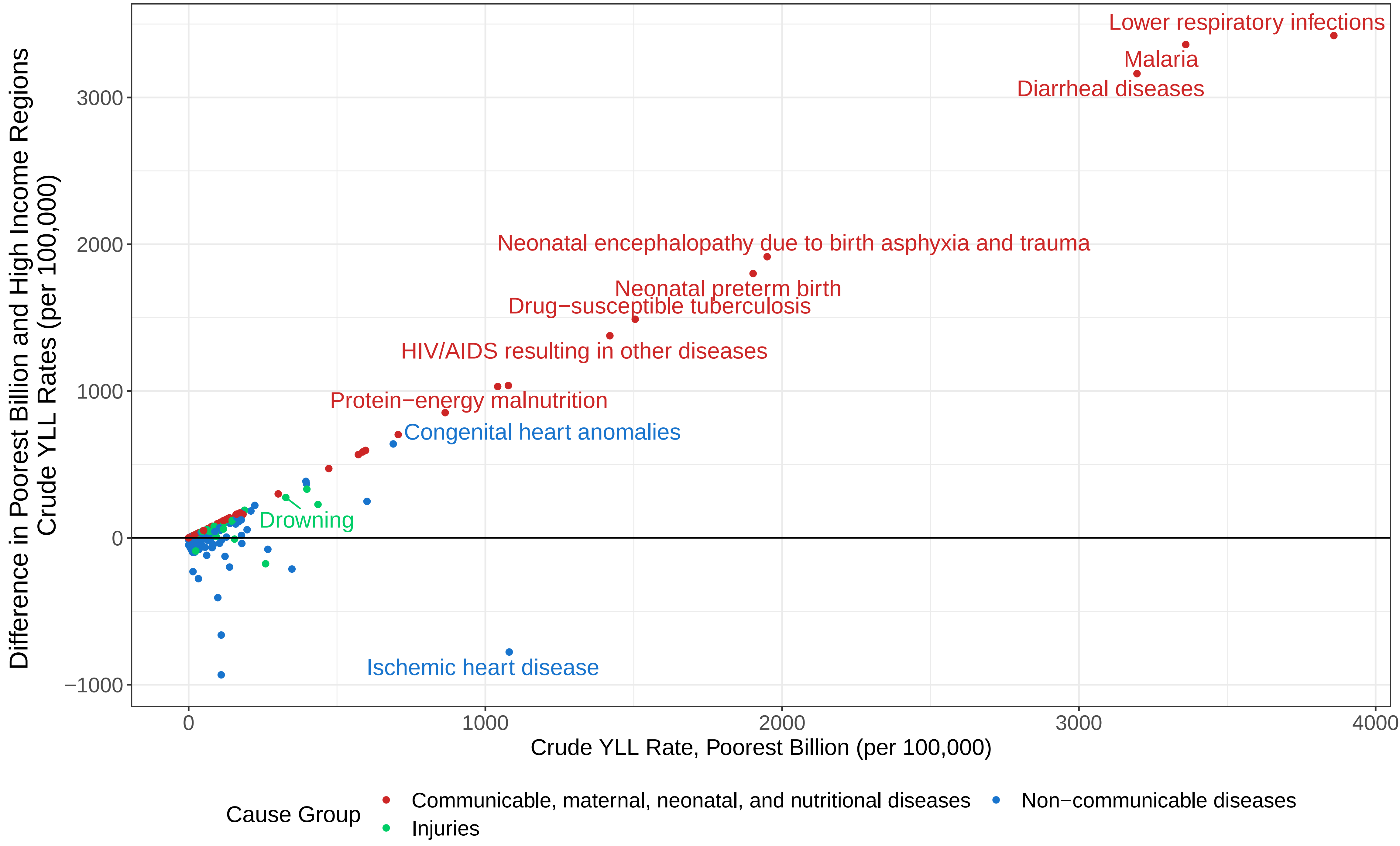

Appendix Figure 9j. Difference between Age−standardized YLL Rates in Low−income and High−income Regions

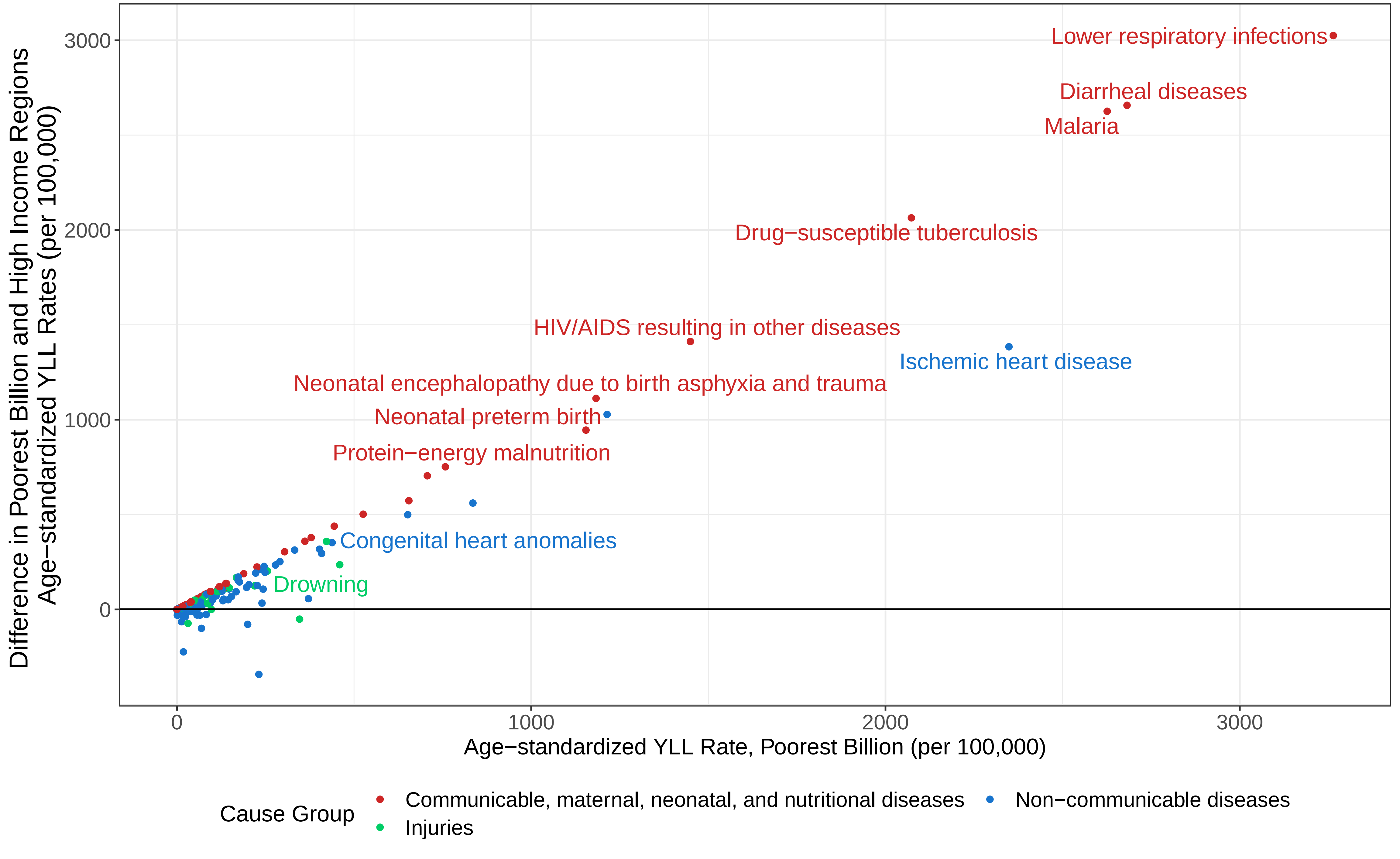

Appendix Figure 10a. Difference between Crude YLD Rates in Poorest Billion and High−income Regions, Selective Ecological Approach

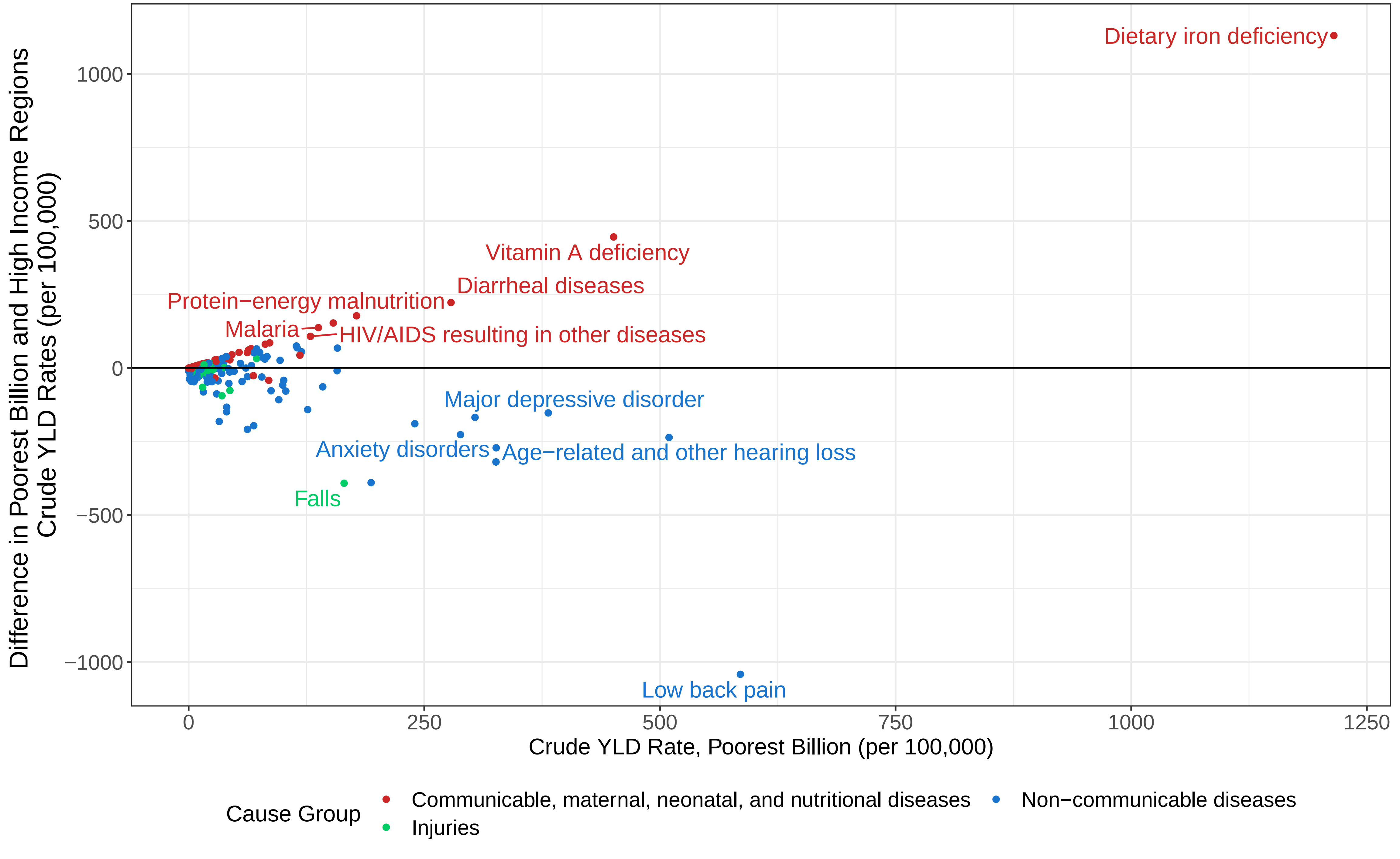

Appendix Figure 10b. Difference between Age−standardized YLD Rates in Poorest Billion and High−income Regions, Selective Ecological Approach

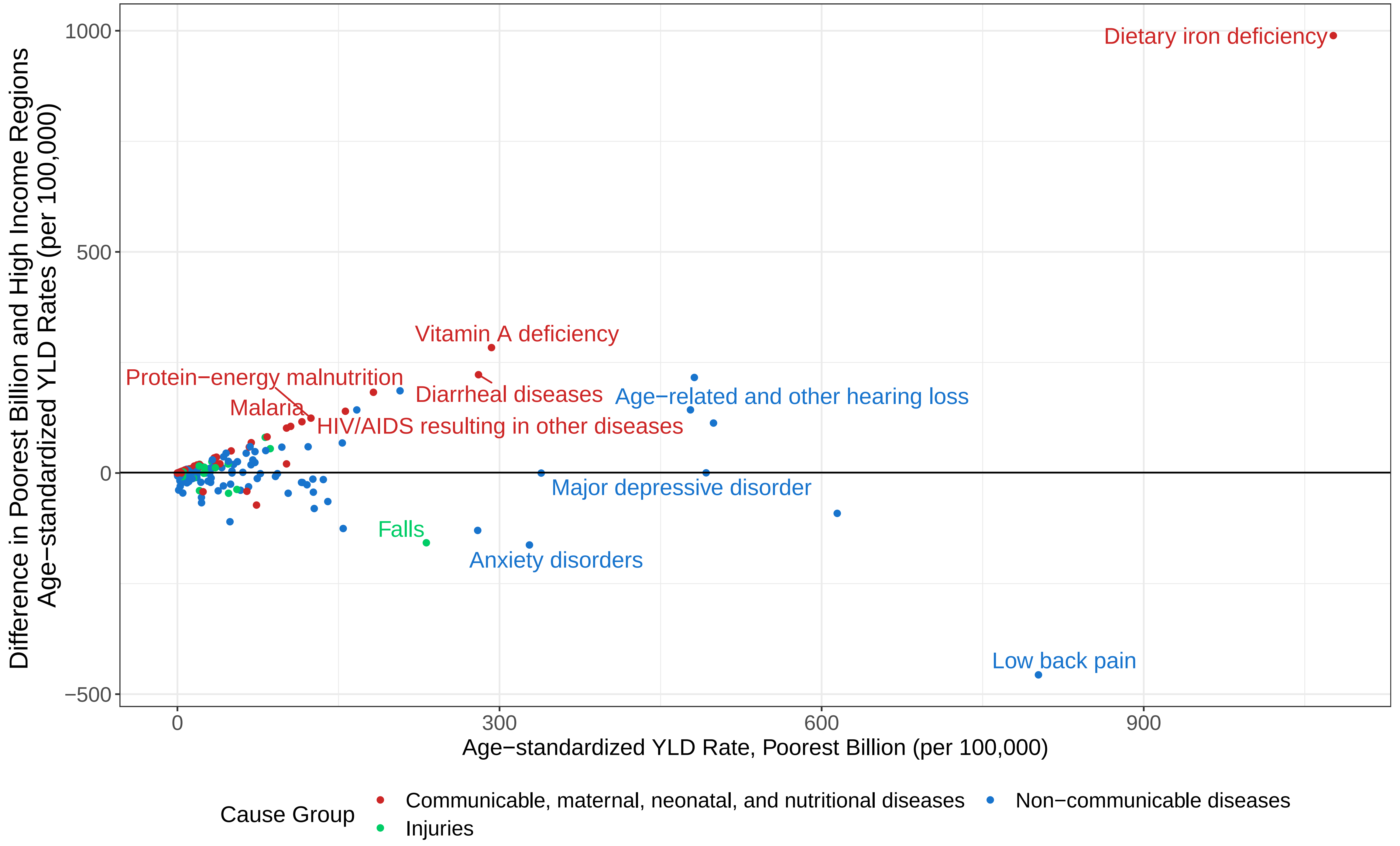

Appendix Figure 10c. Difference between Crude YLD Rates in Poorest Billion and High−income Regions, Full Ecological Approach

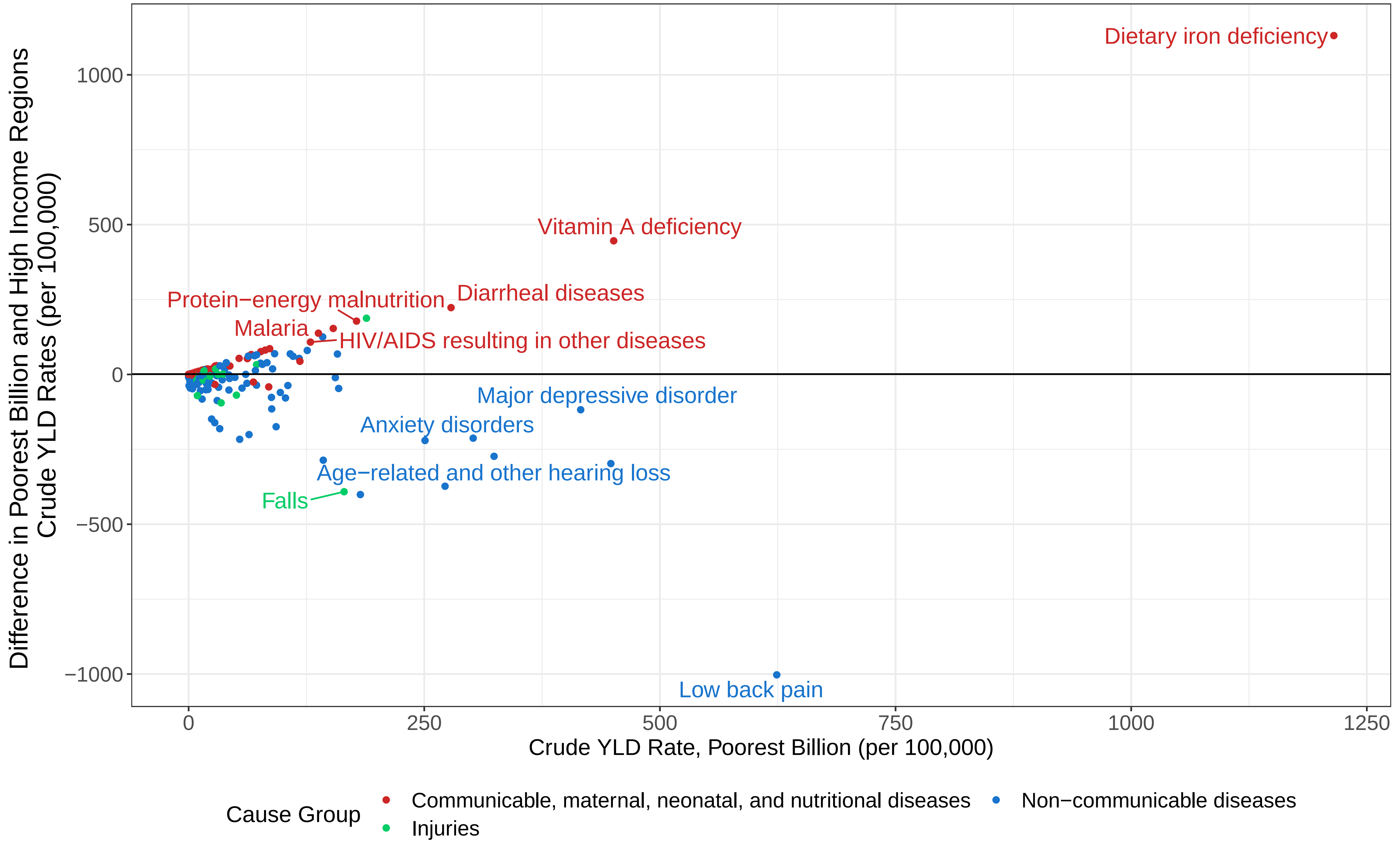

Appendix Figure 10d. Difference between Age−standardized YLD Rates in Poorest Billion and High−income Regions, Full Ecological Approach

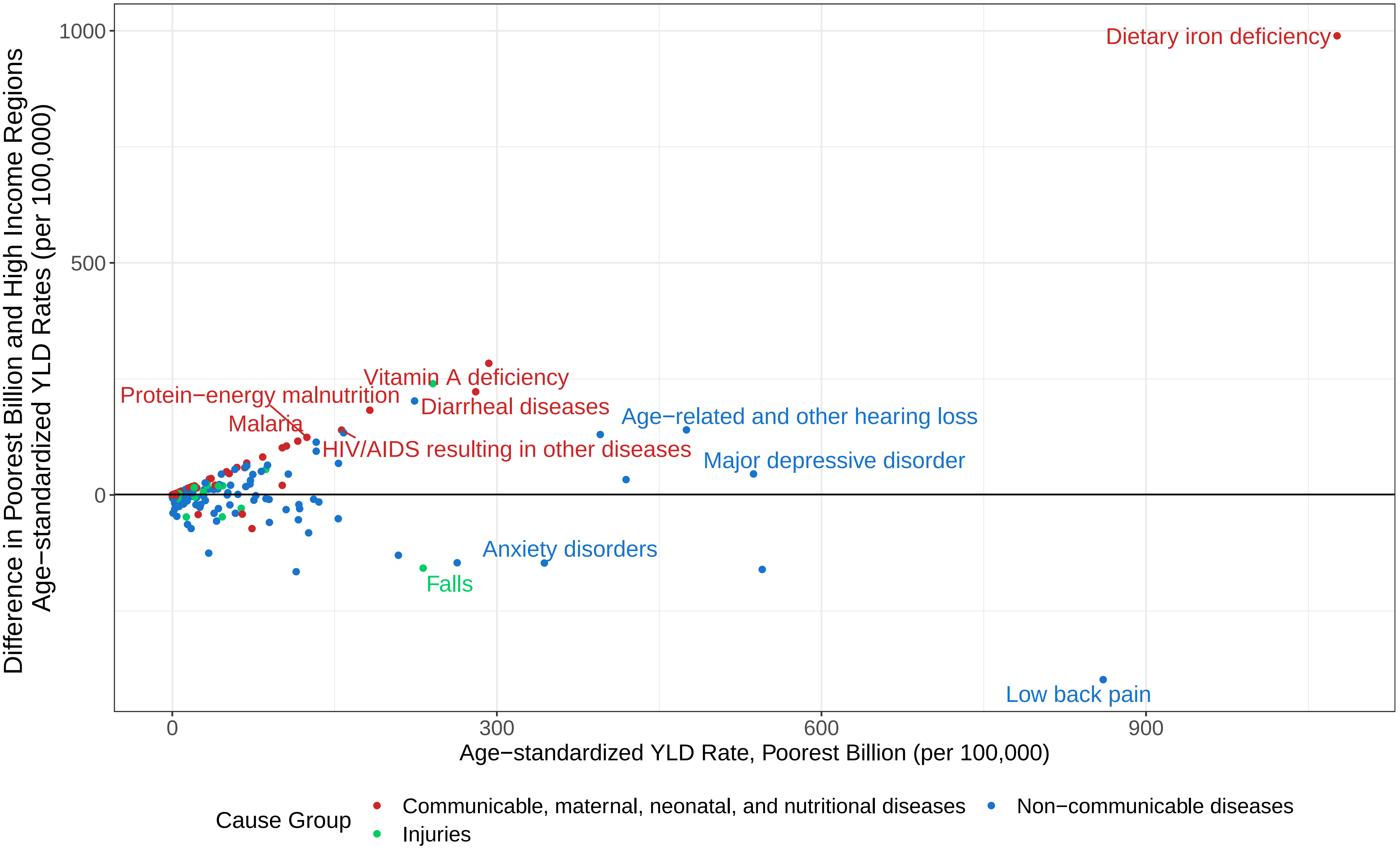

Appendix Figure 10e. Difference between Crude YLD Rates in Poorest Billion and High−income Regions, Poorest Billion Aggregation

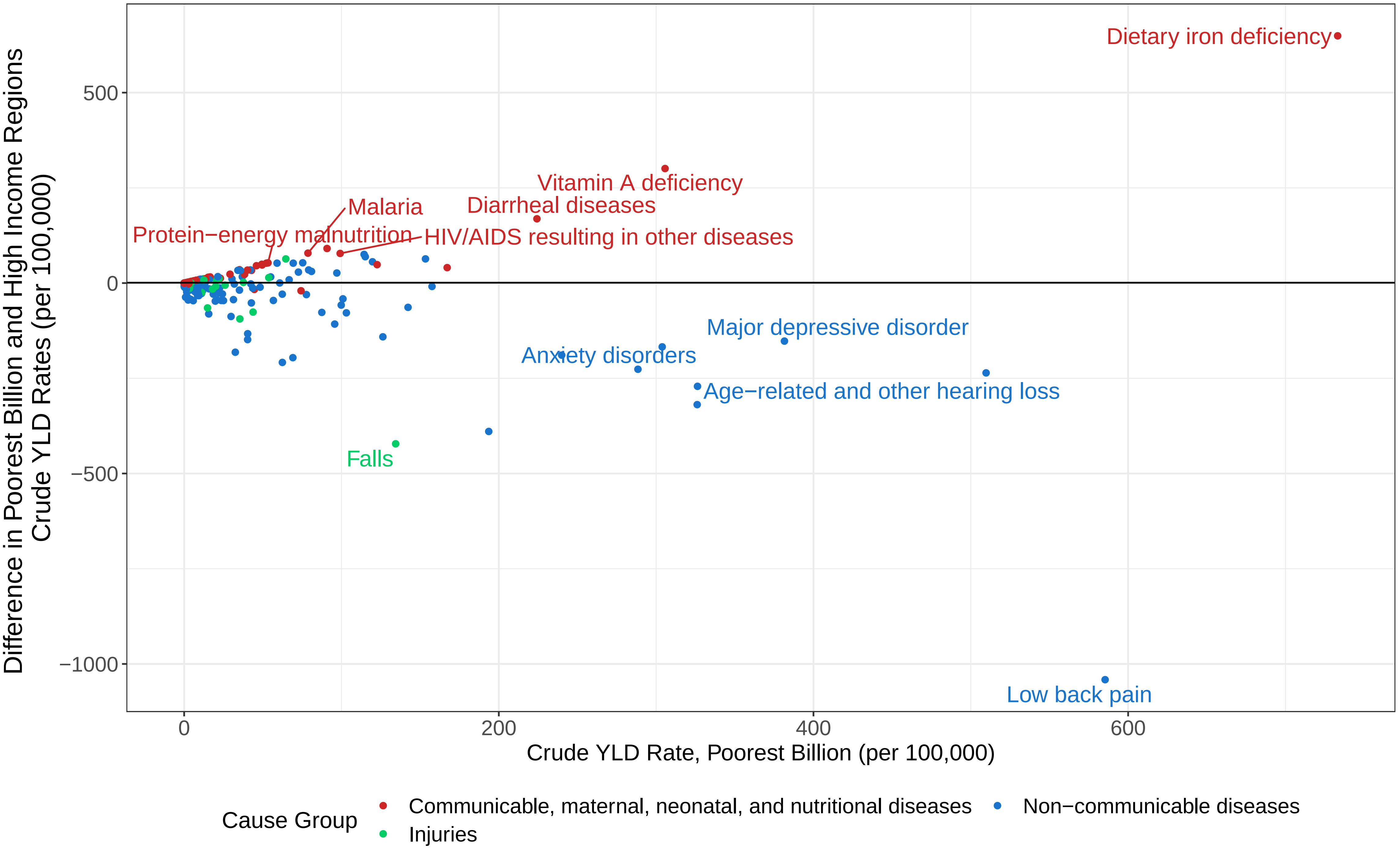

Appendix Figure 10f. Difference between Age−standardized YLD Rates in Poorest Billion and High−income Regions, Poorest Billion Aggregation

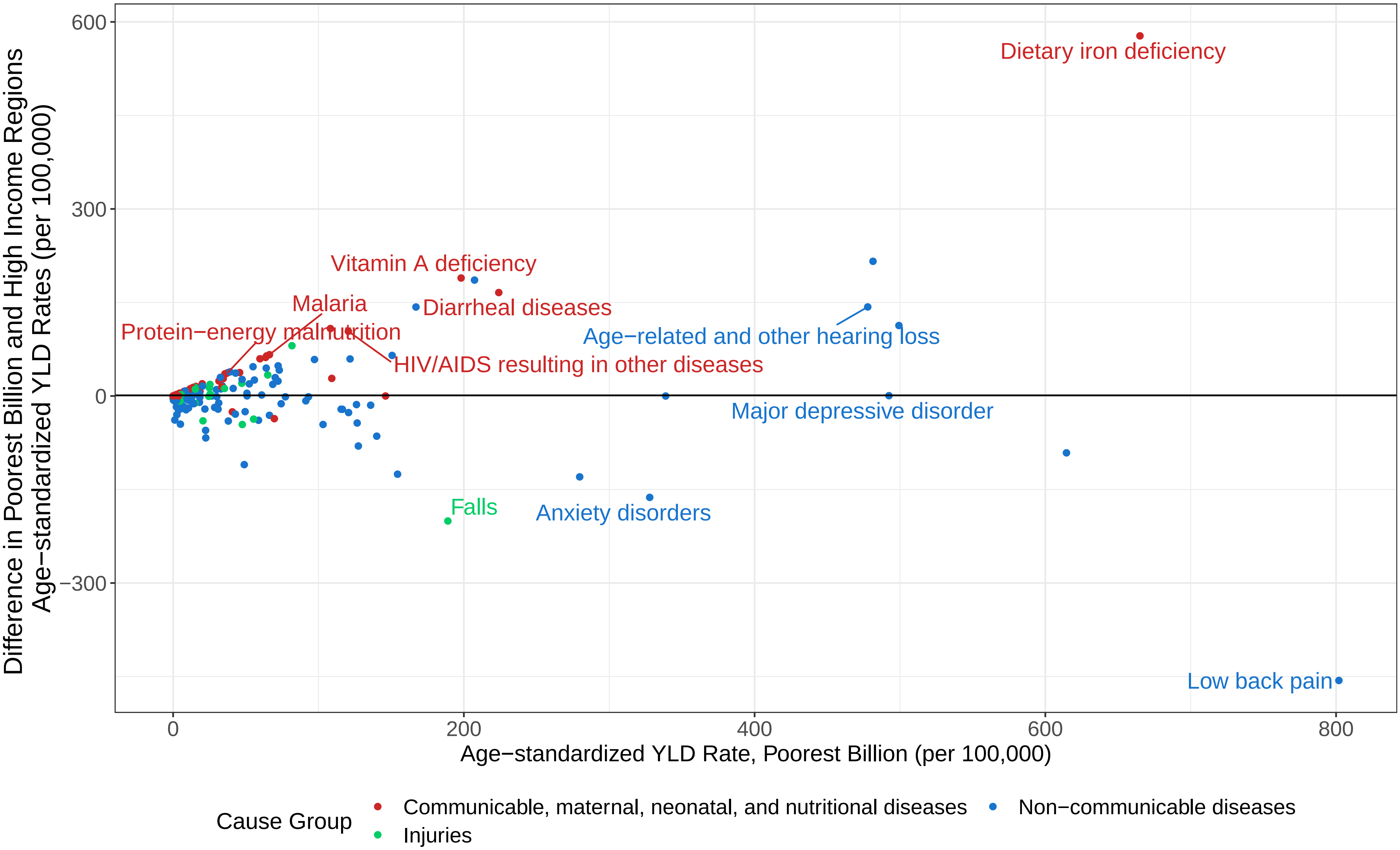

Appendix Figure 10g. Difference between Crude YLD Rates in Poorest Billion and High−income Regions, Country Aggregation

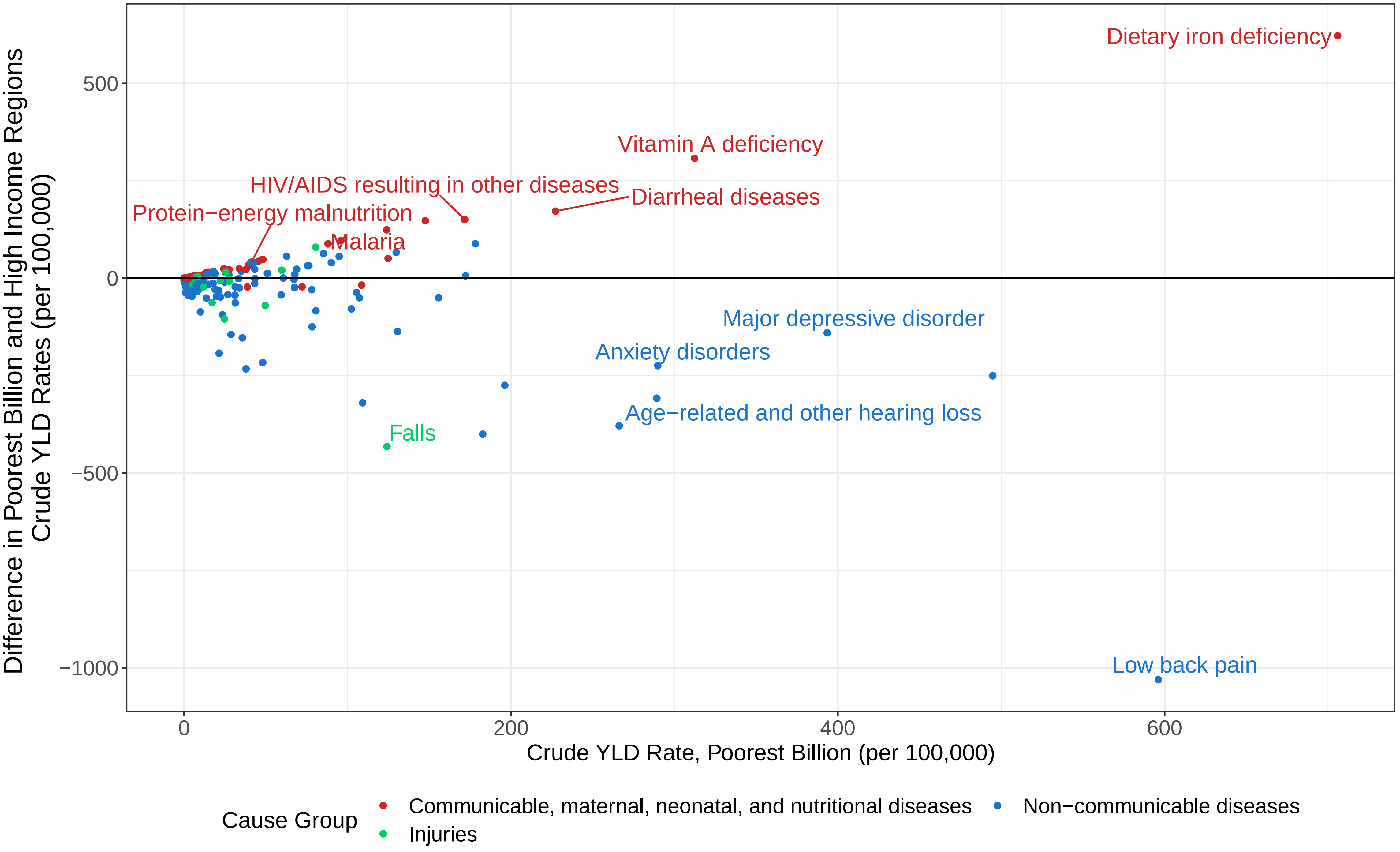

Appendix Figure 10h. Difference between Age−standardized YLD Rates in Poorest Billion and High−income Regions, Country Aggregation

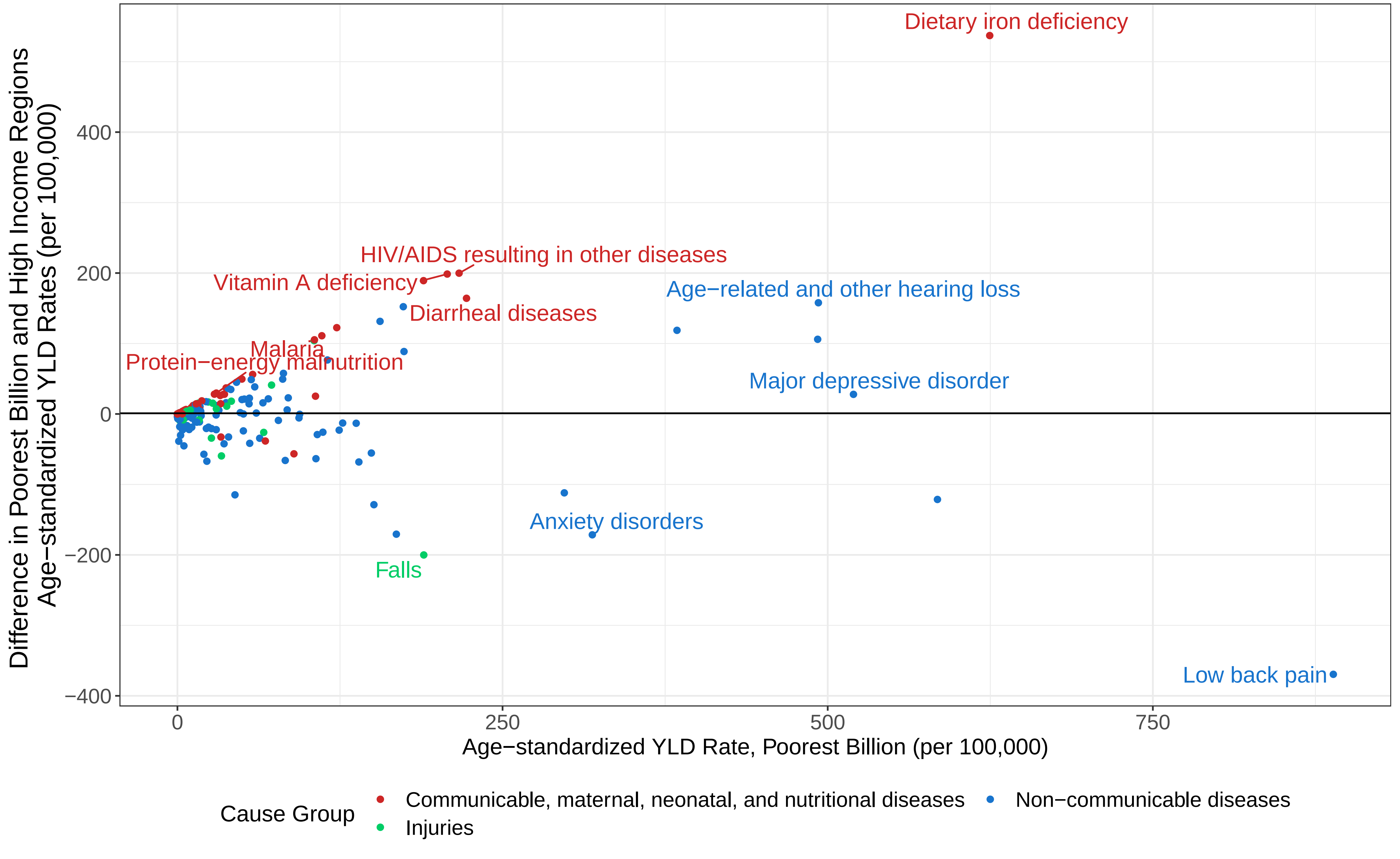

Appendix Figure 10i. Difference between Crude YLD Rates in Low−income and High−income Regions

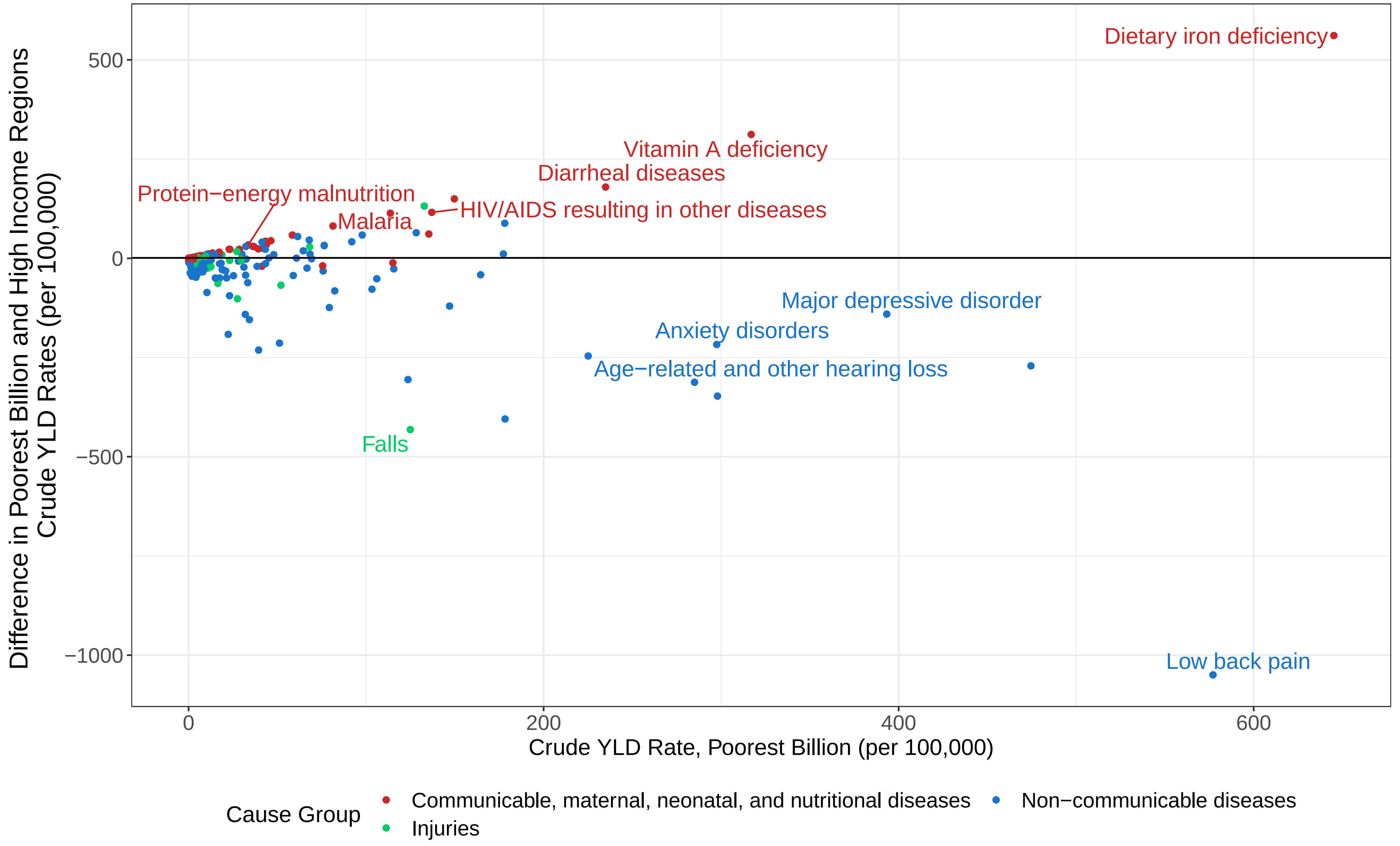

Appendix Figure 10j. Difference between Age−standardized YLD Rates in Low−income and High−income Regions

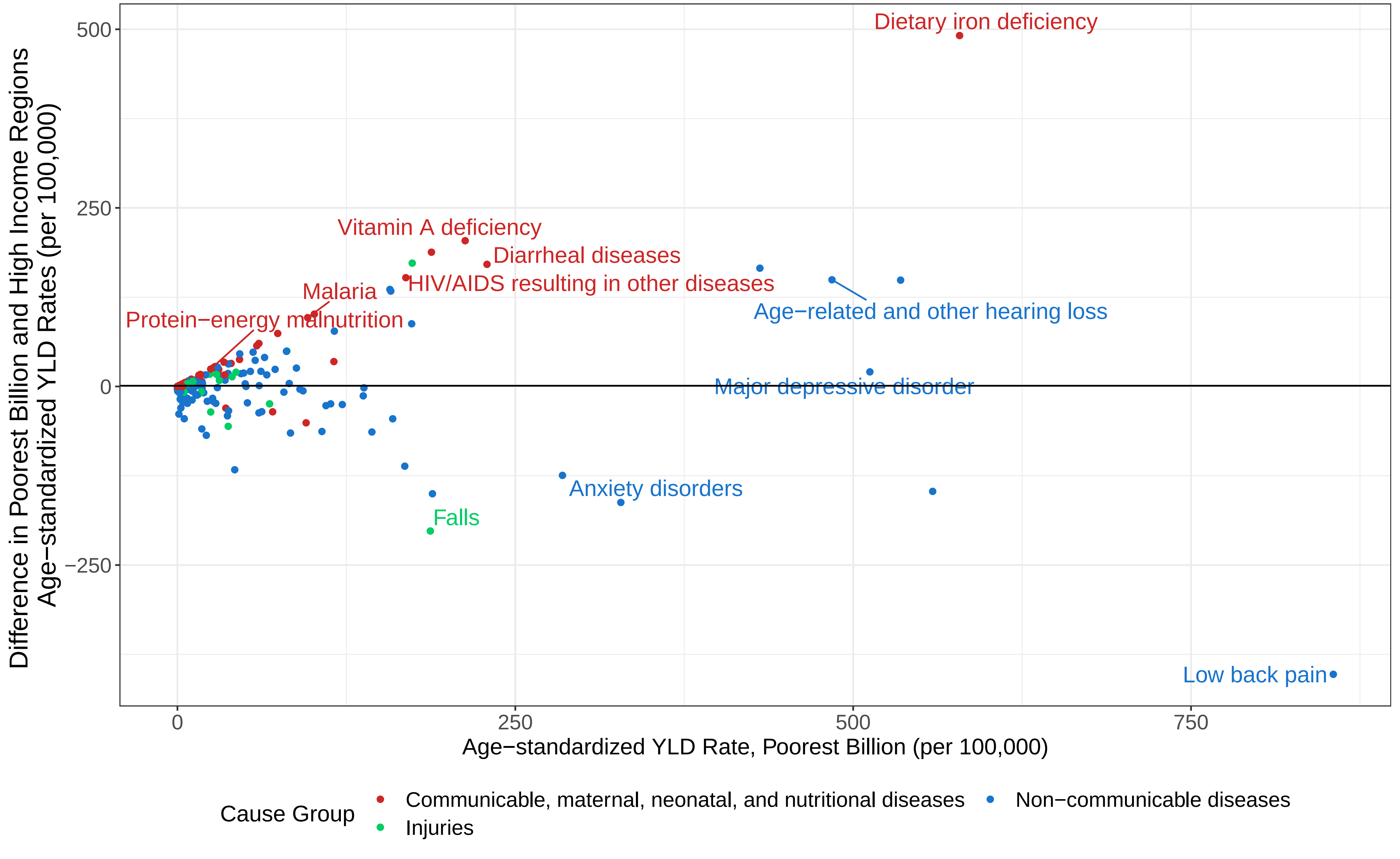

Appendix Figure 11a. Difference between Crude YLL Rates in Poorest Billion and High−income Regions, Selective Ecological Approach

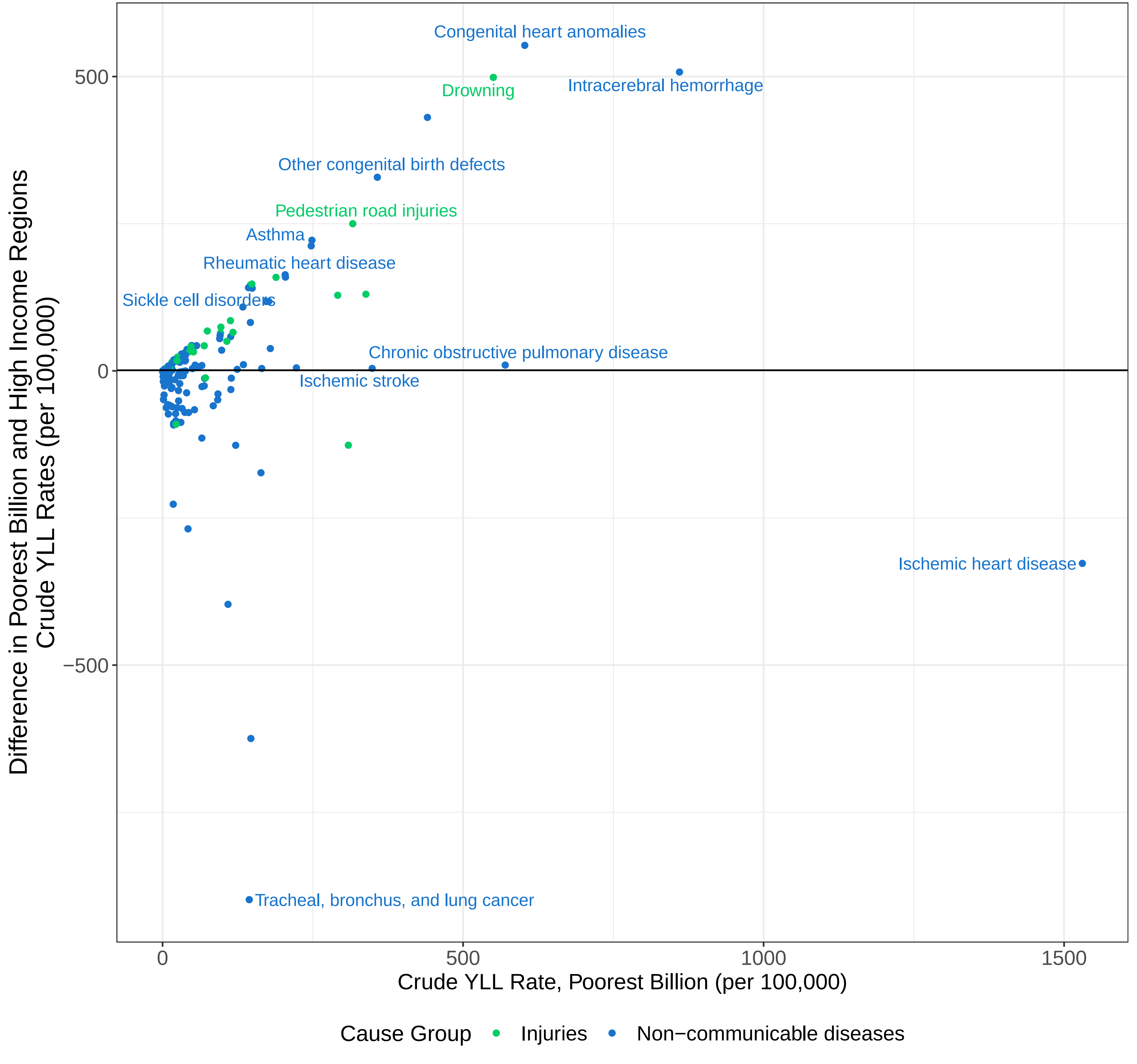

Appendix Figure 11b. Difference between Age−standardized YLL Rates in Poorest Billion and High−income Regions, Selective Ecological Approach

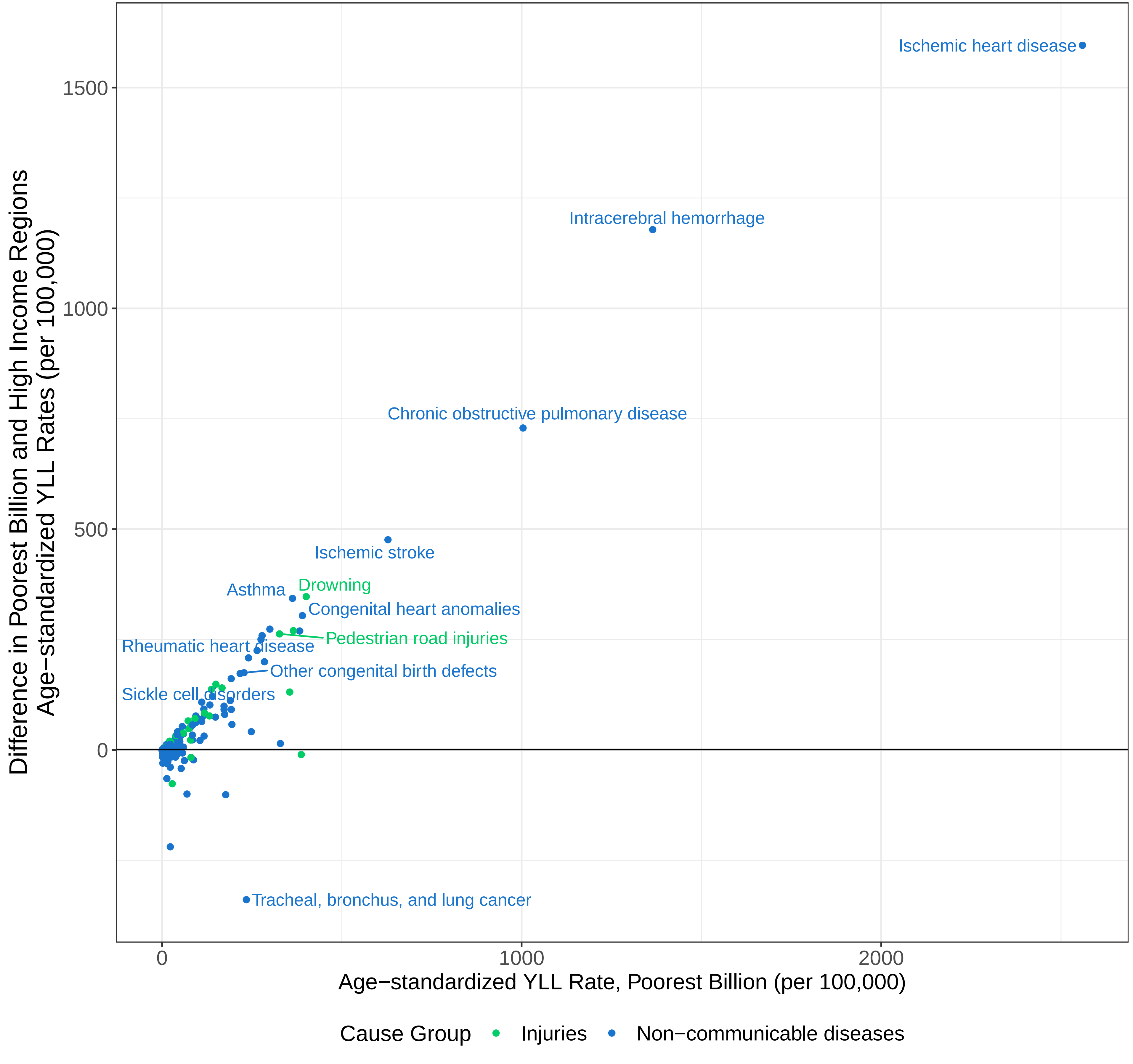

Appendix Figure 11c. Difference between Crude YLL Rates in Poorest Billion and High−income Regions, Full Ecological Approach

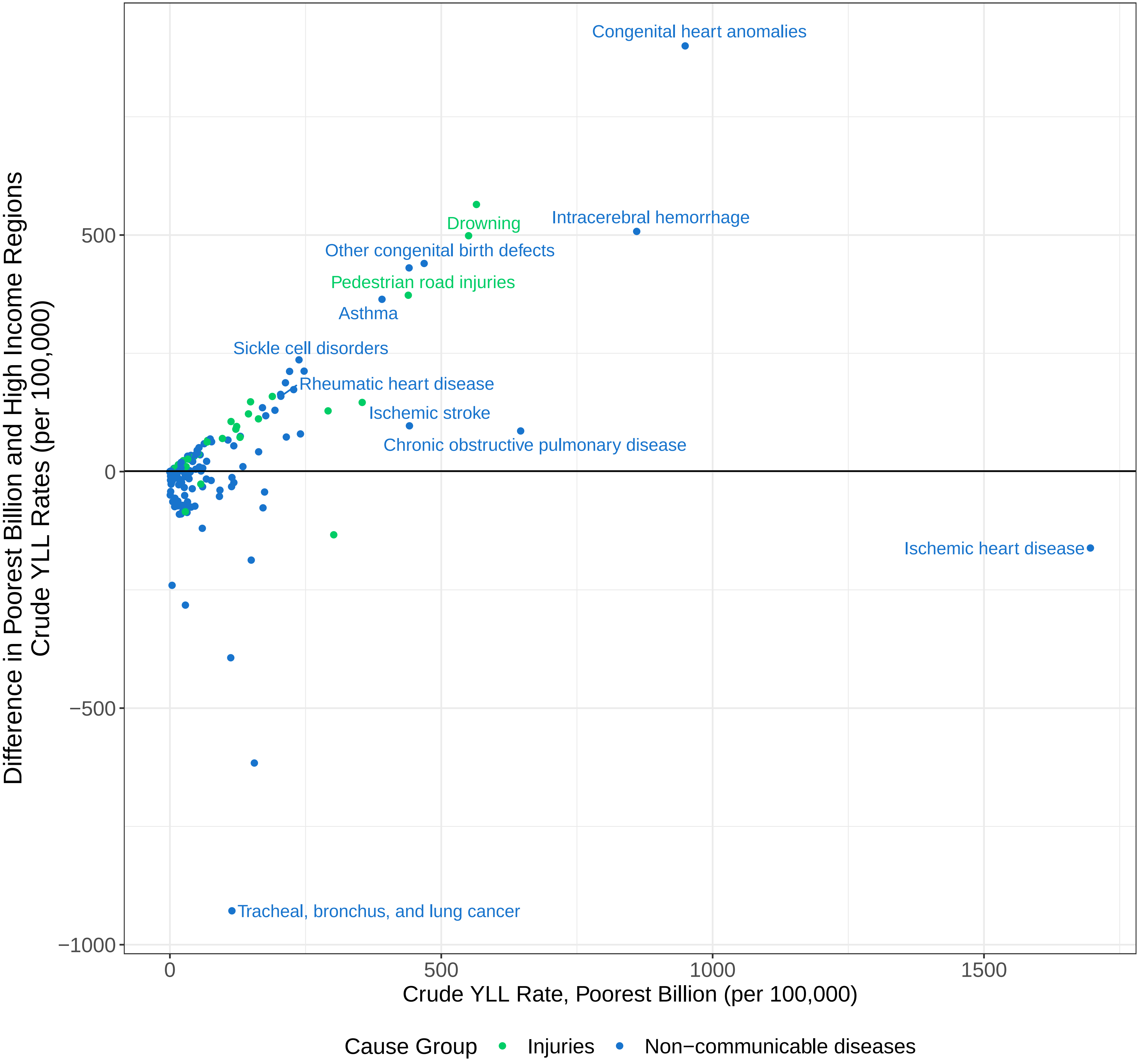

Appendix Figure 11d. Difference between Age−standardized YLL Rates in Poorest Billion and High−income Regions, Full Ecological Approach

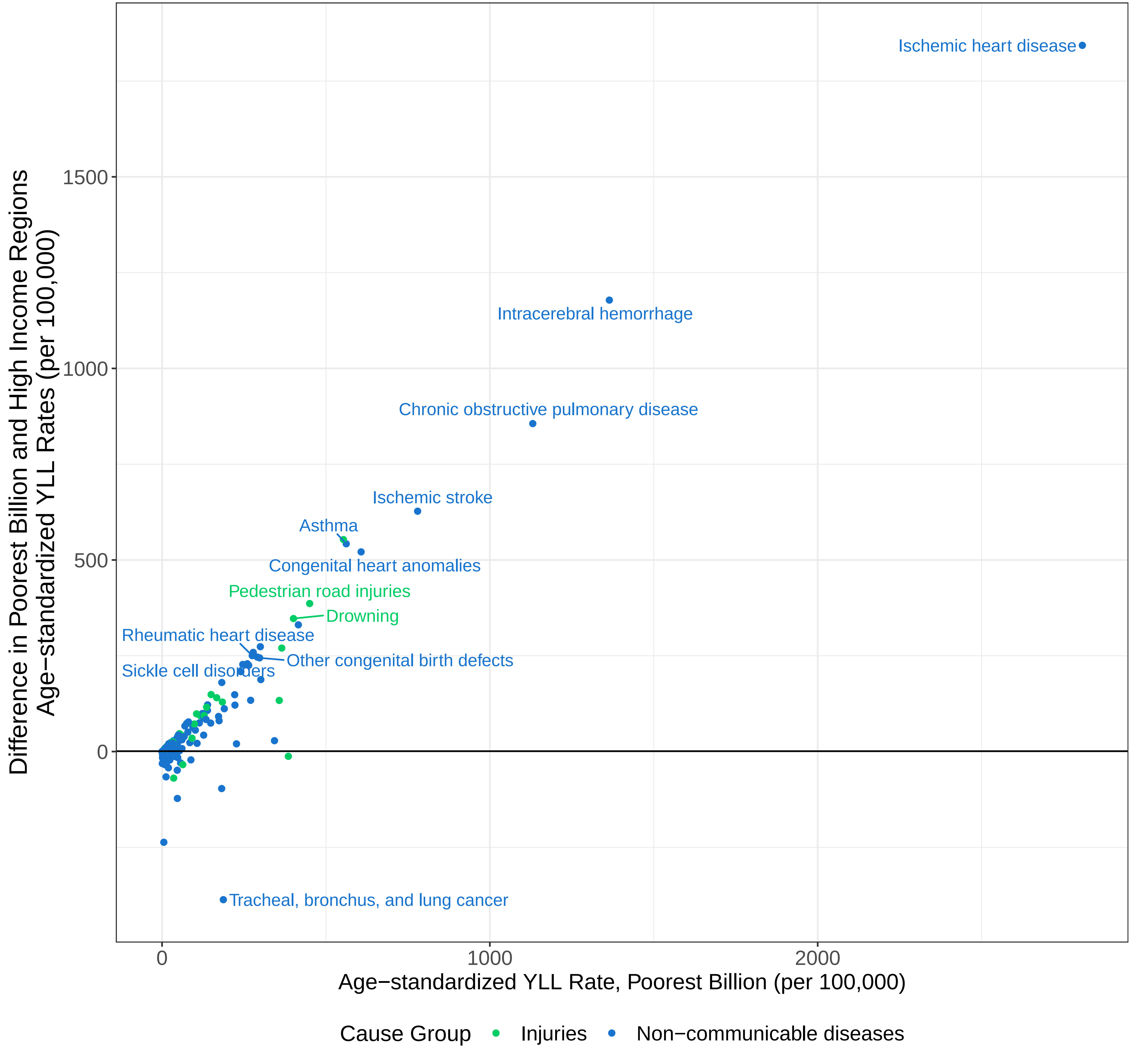

Appendix Figure 11e. Difference between Crude YLL Rates in Poorest Billion and High−income Regions, Poorest Billion Aggregation

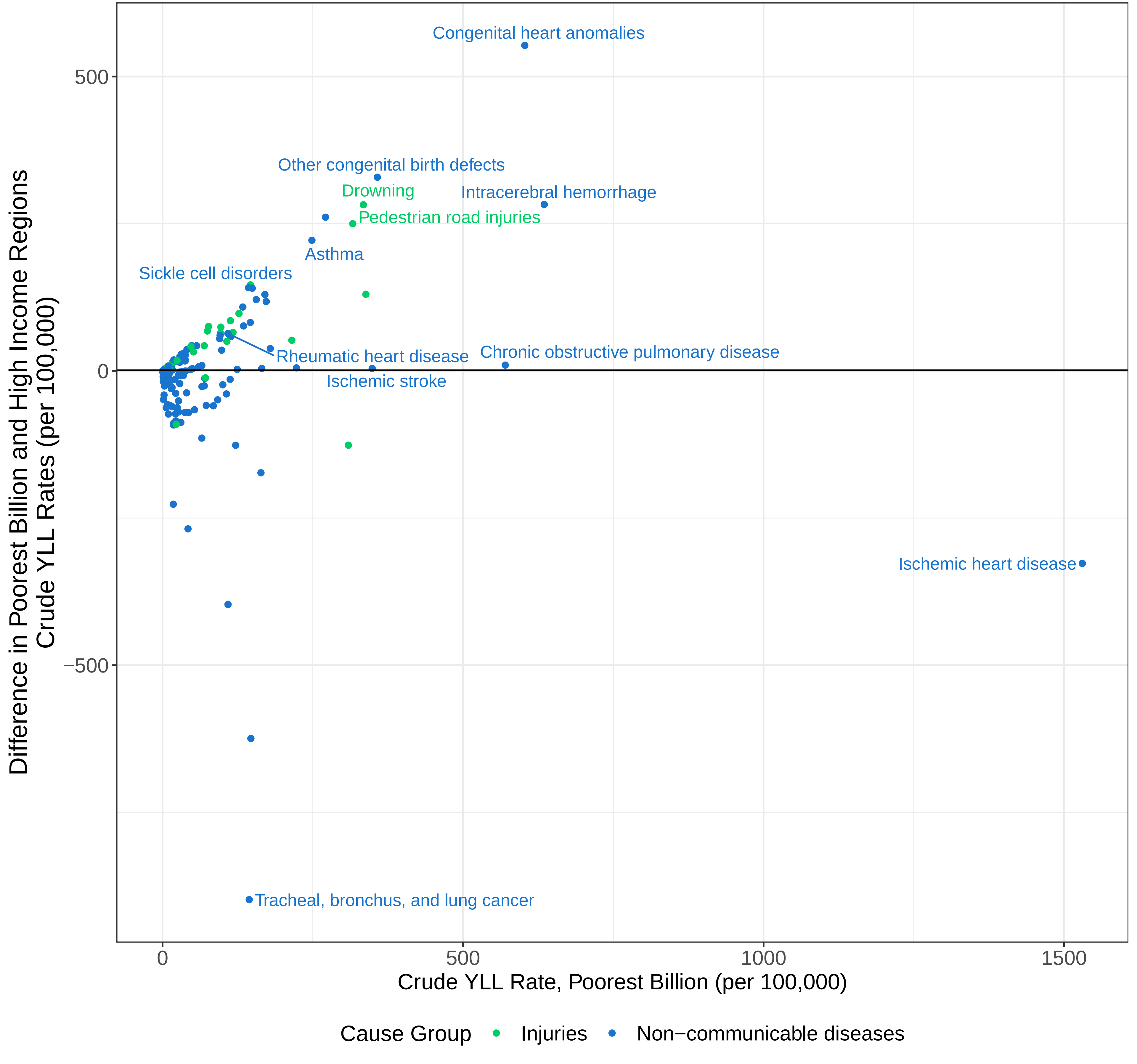

Appendix Figure 11f. Difference between Age−standardized YLL Rates in Poorest Billion and High−income Regions, Poorest Billion Aggregation

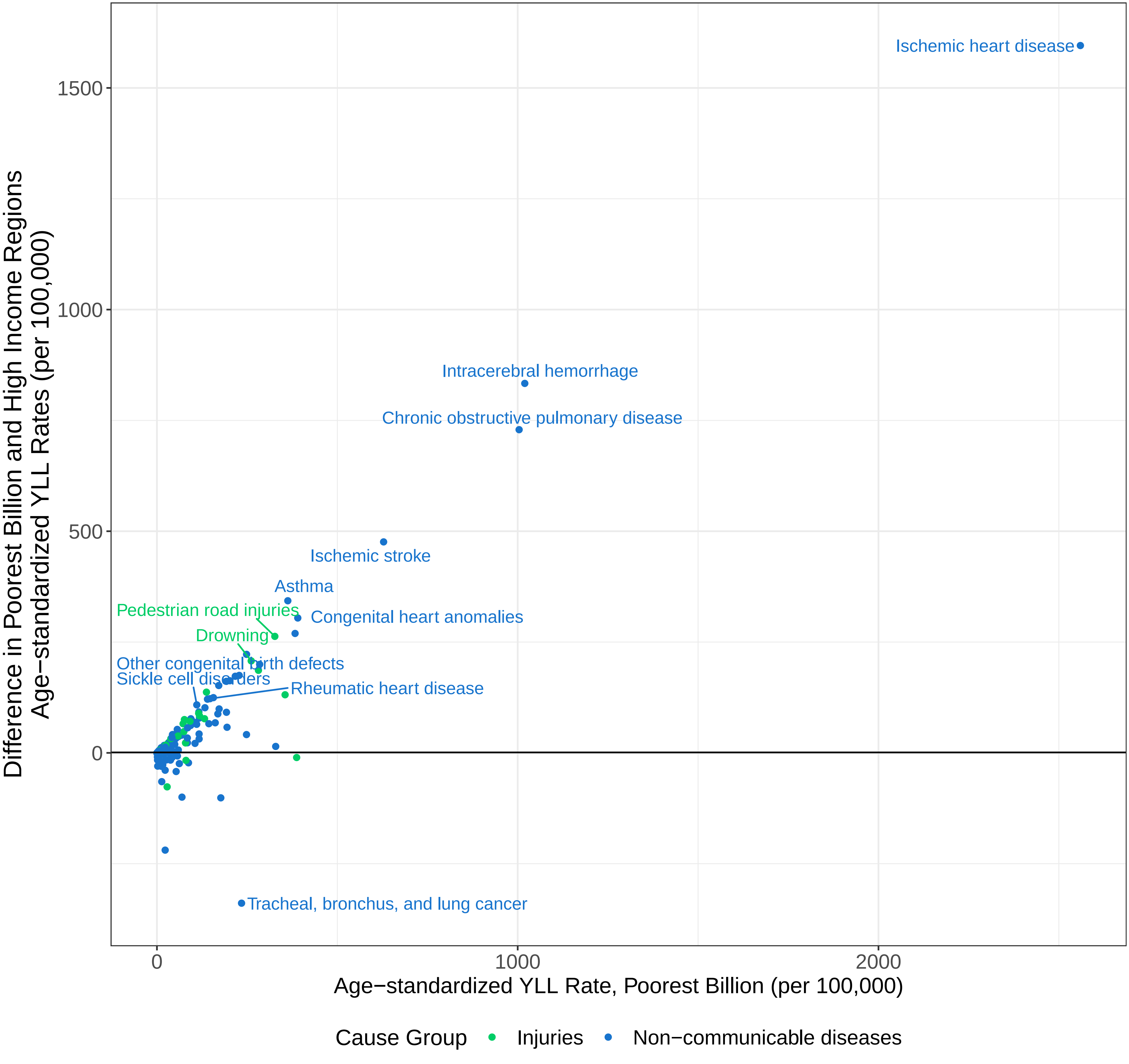

Appendix Figure 11g. Difference between Crude YLL Rates in Poorest Billion and High−income Regions, Country Aggregation

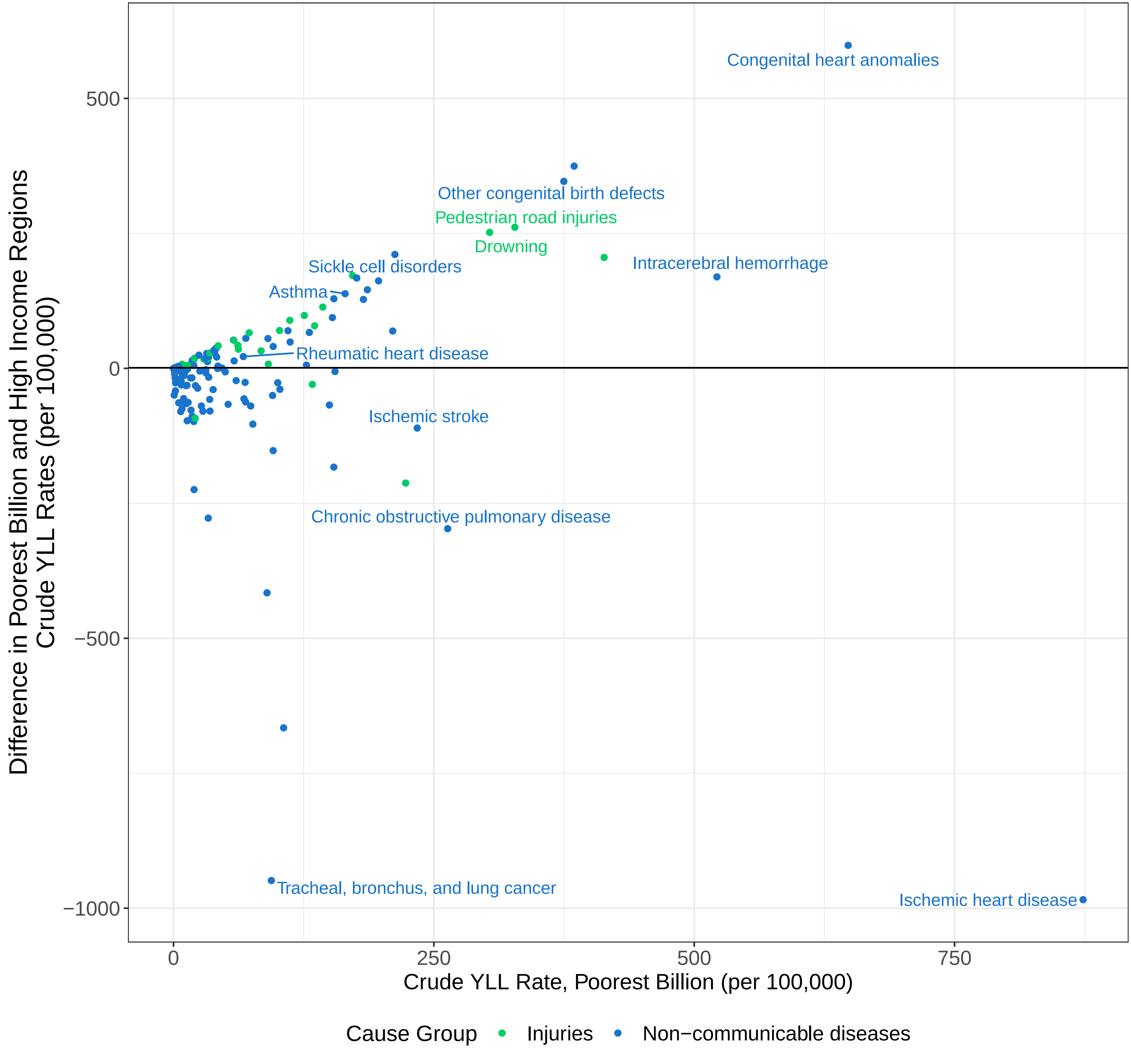

Appendix Figure 11h. Difference between Age−standardized YLL Rates in Poorest Billion and High−income Regions, Country Aggregation

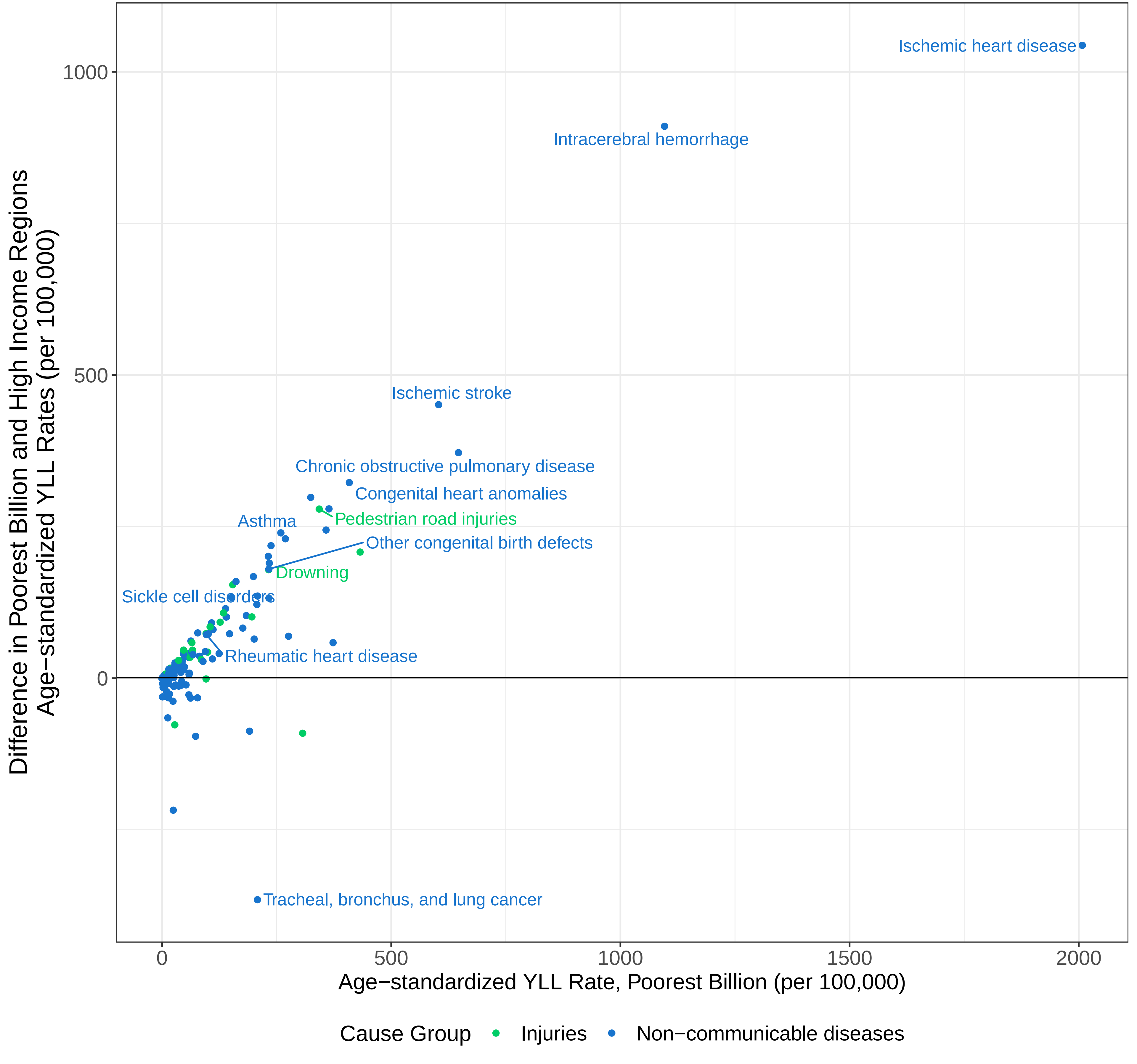

Appendix Figure 11i. Difference between Crude YLL Rates in Low−income and High−income Regions

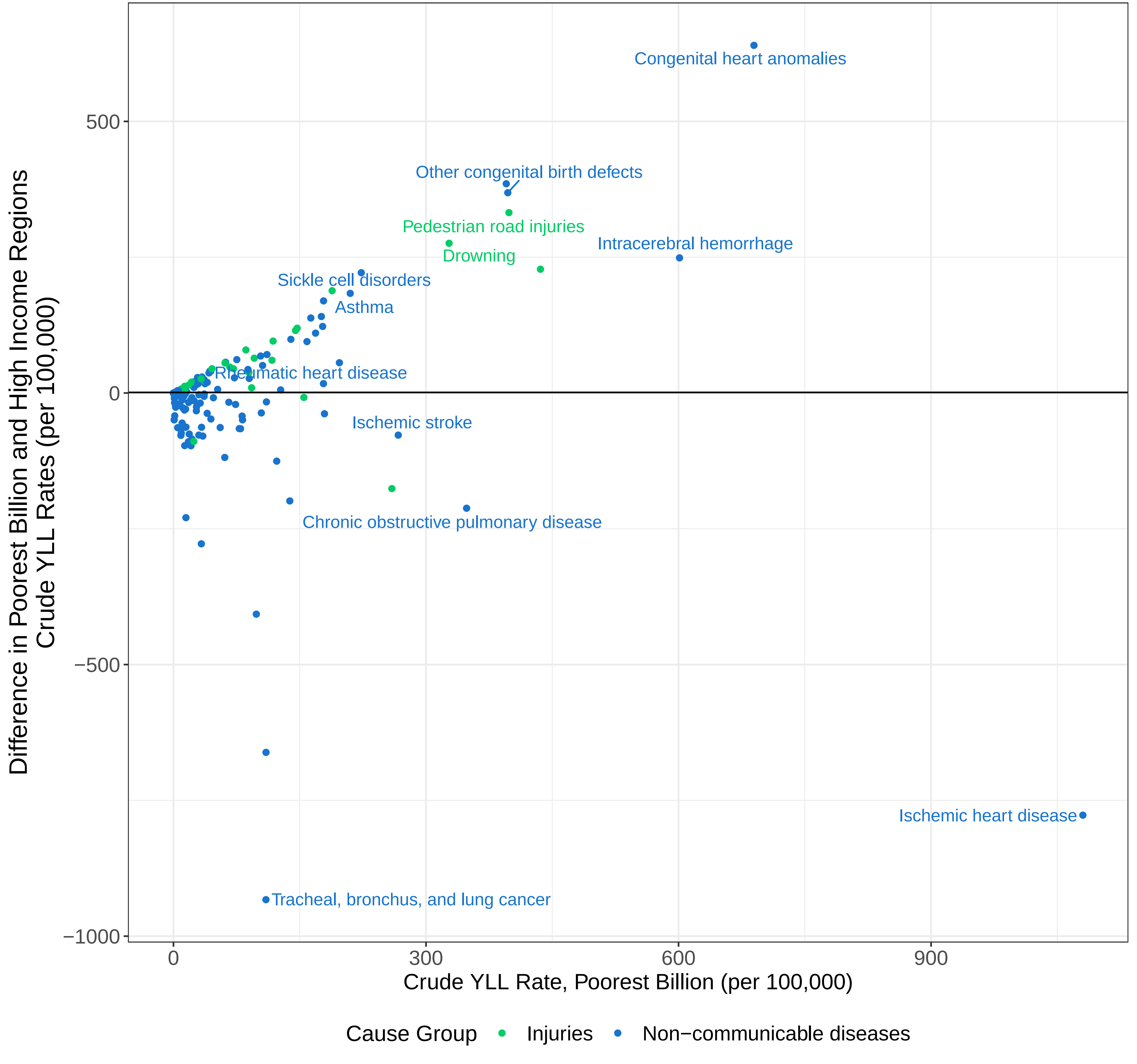

Appendix Figure 11j. Difference between Age−standardized YLL Rates in Low−income and High−income Regions

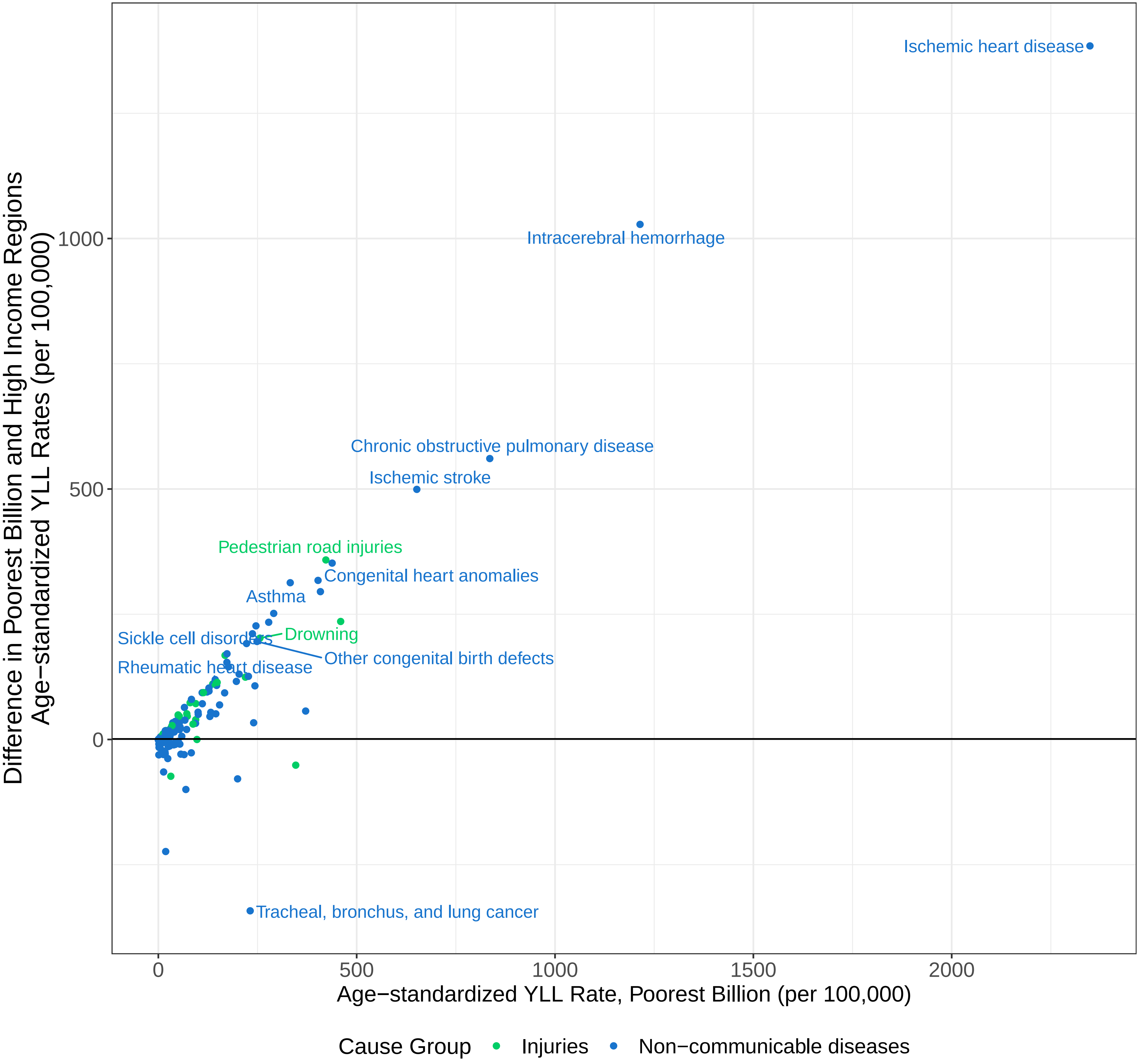

Appendix Figure 12a. Difference between Crude YLD Rates in Poorest Billion and High−income Regions, Selective Ecological Approach

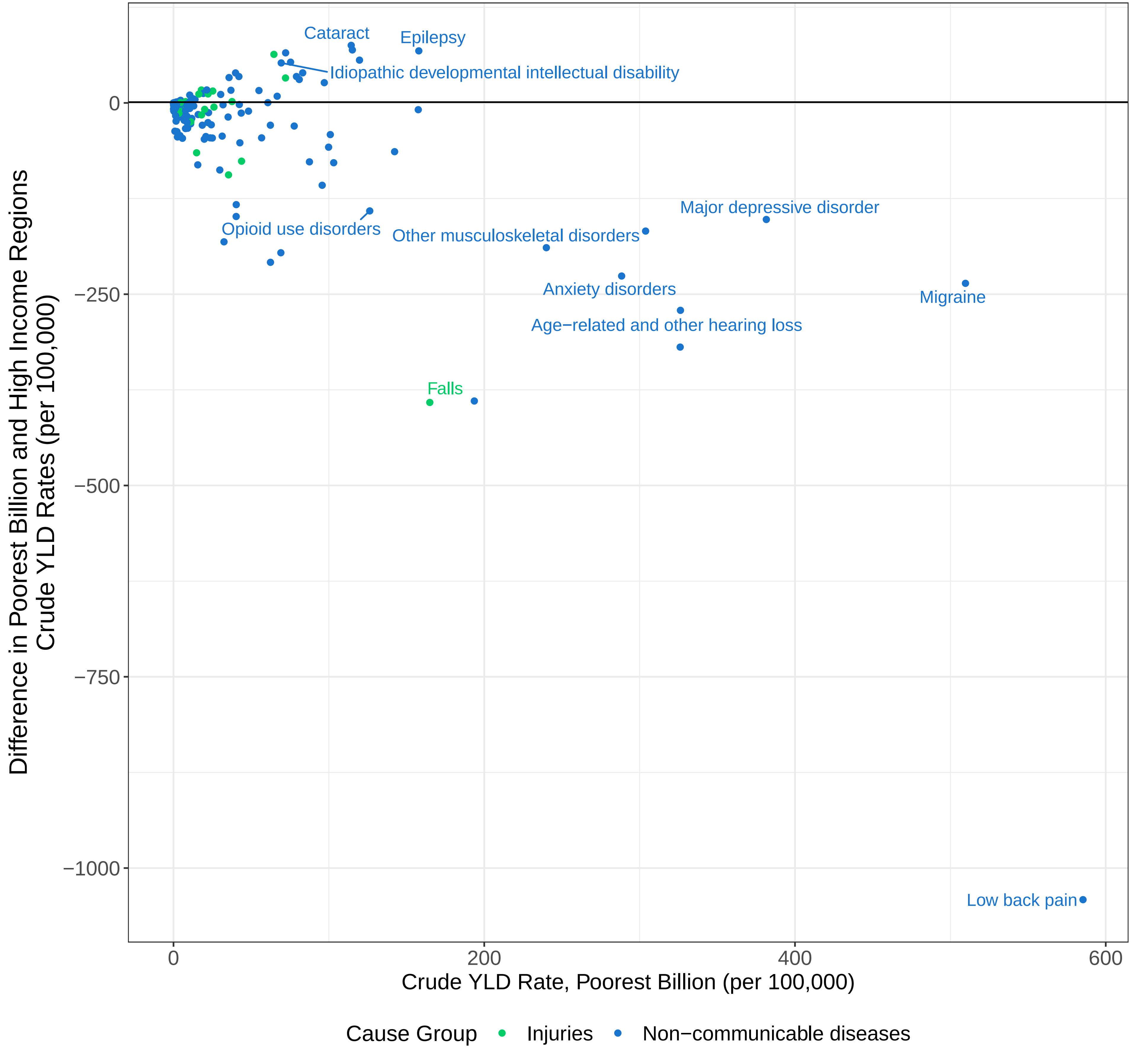

Appendix Figure 12b. Difference between Age−standardized YLD Rates in Poorest Billion and High−income Regions, Selective Ecological Approach

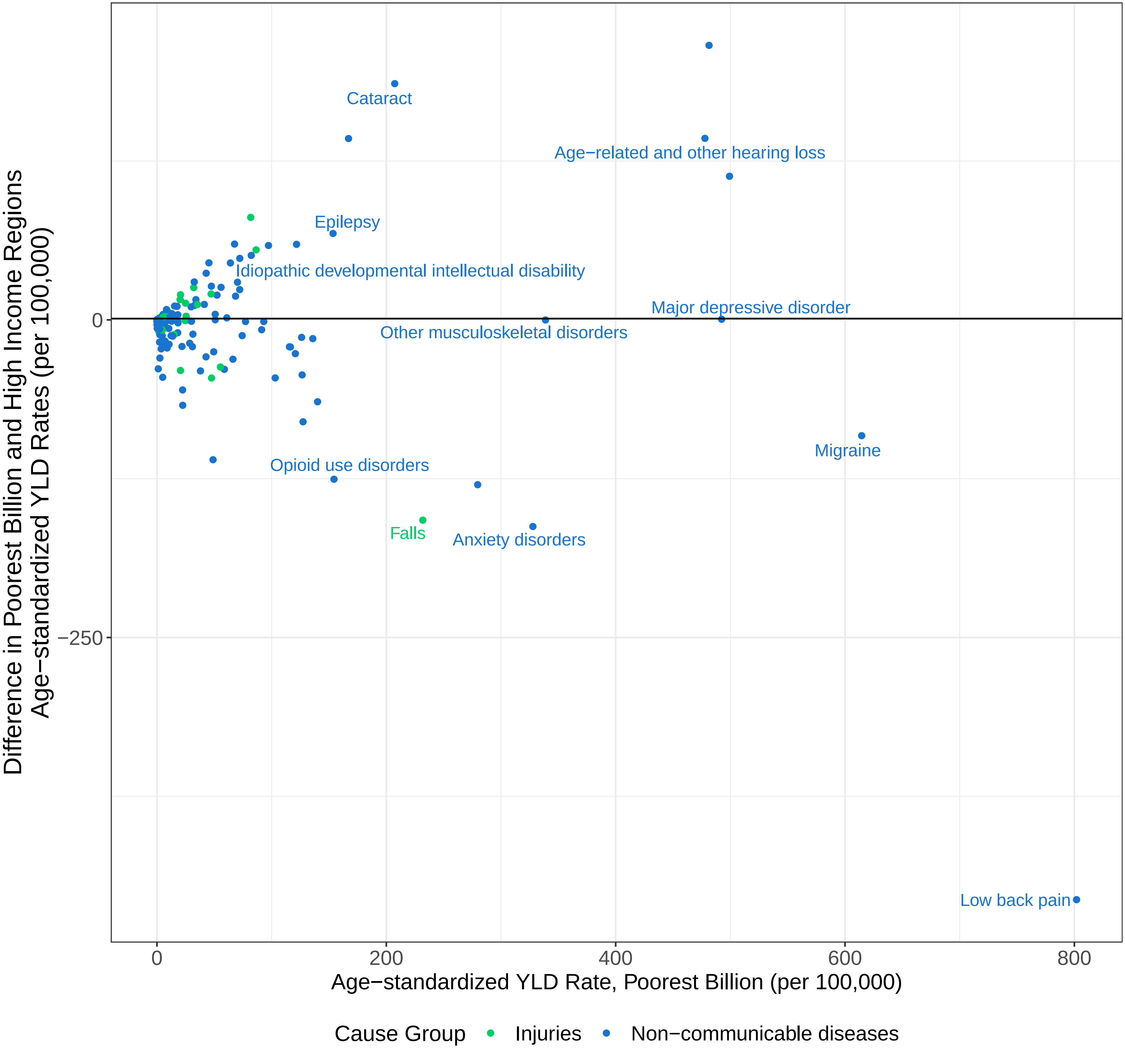

Appendix Figure 12c. Difference between Crude YLD Rates in Poorest Billion and High−income Regions, Full Ecological Approach

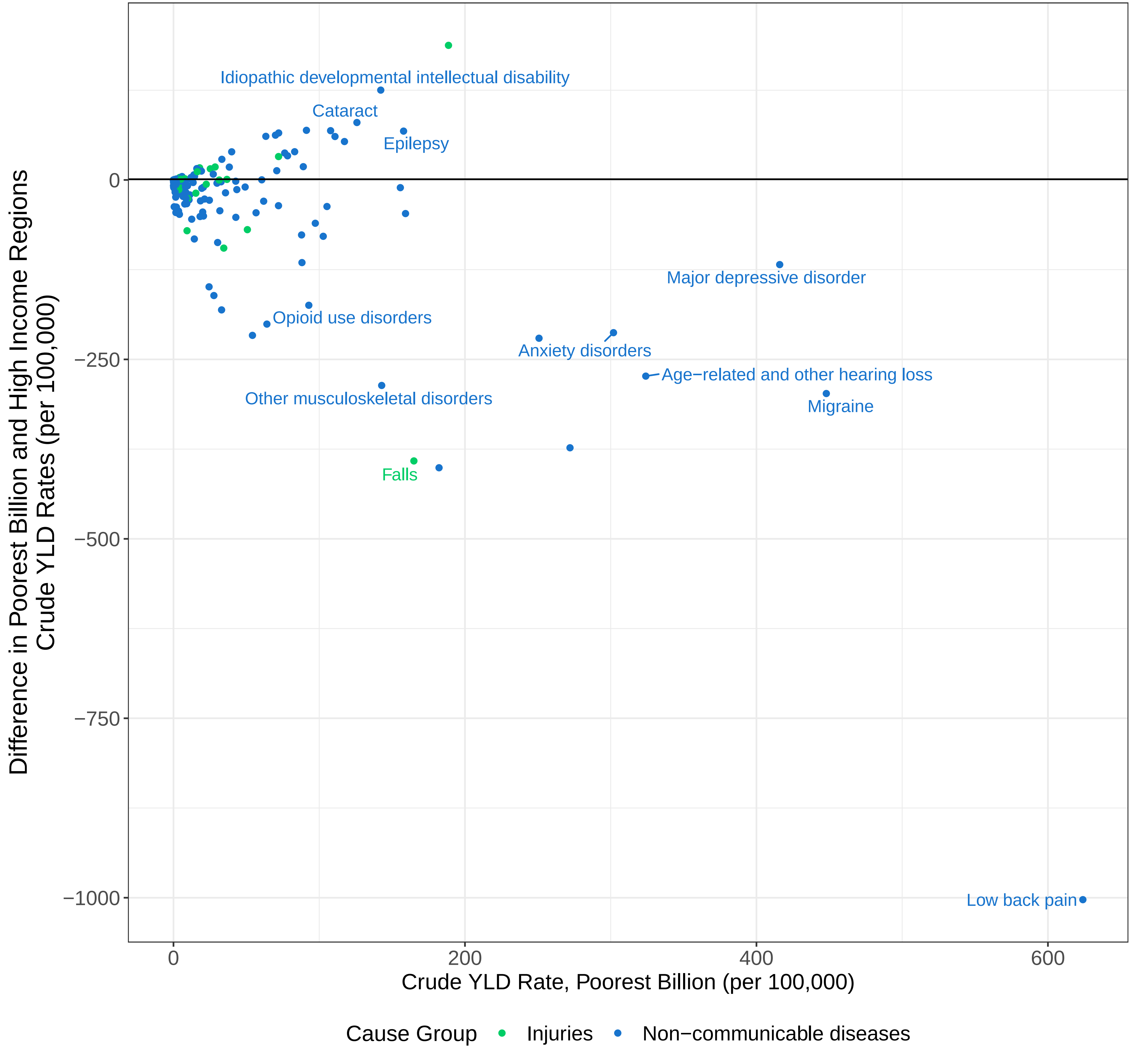

Appendix Figure 12d. Difference between Age−standardized YLD Rates in Poorest Billion and High−income Regions, Full Ecological Approach

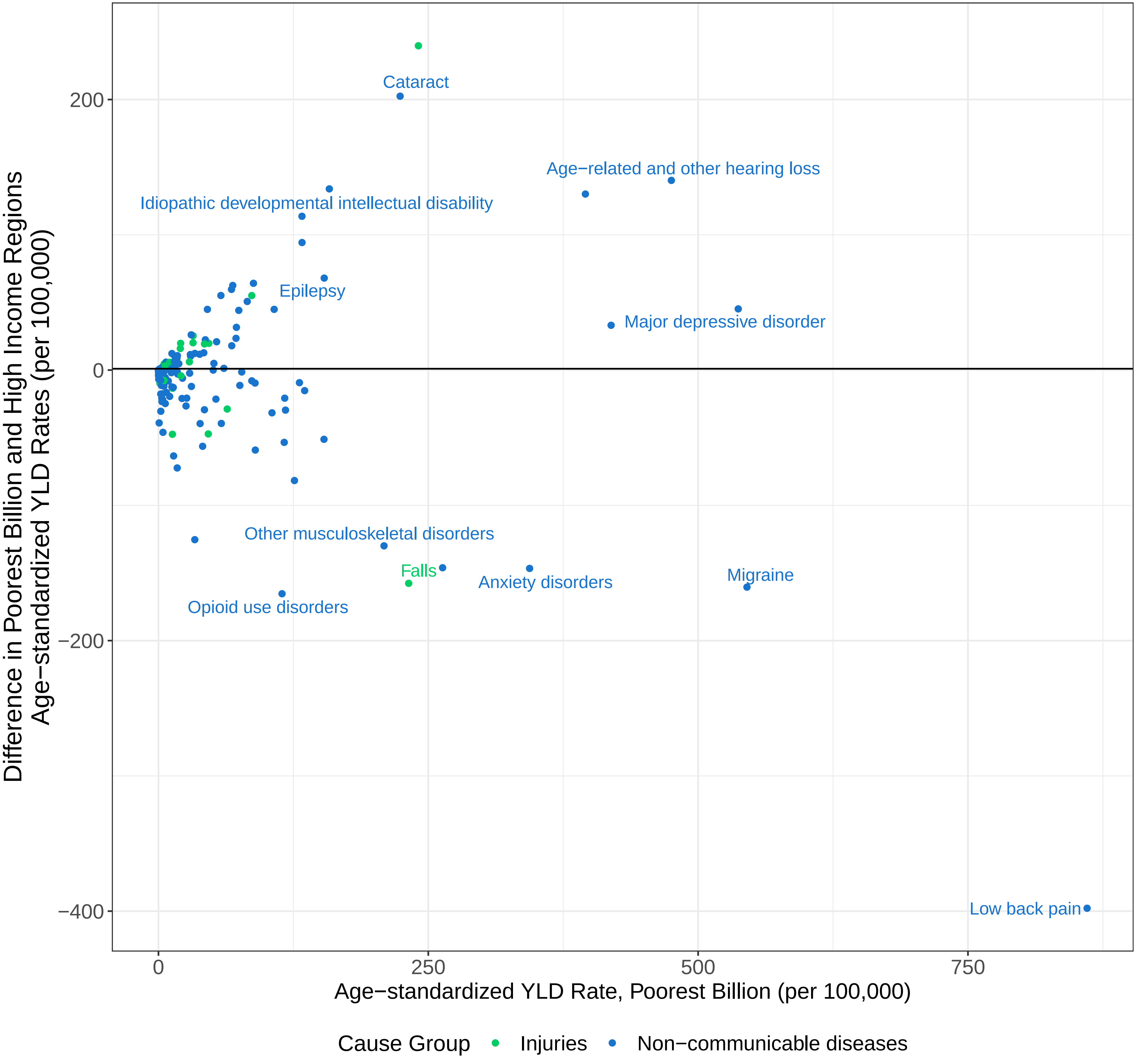

Appendix Figure 12e. Difference between Crude YLD Rates in Poorest Billion and High−income Regions, Poorest Billion Aggregation

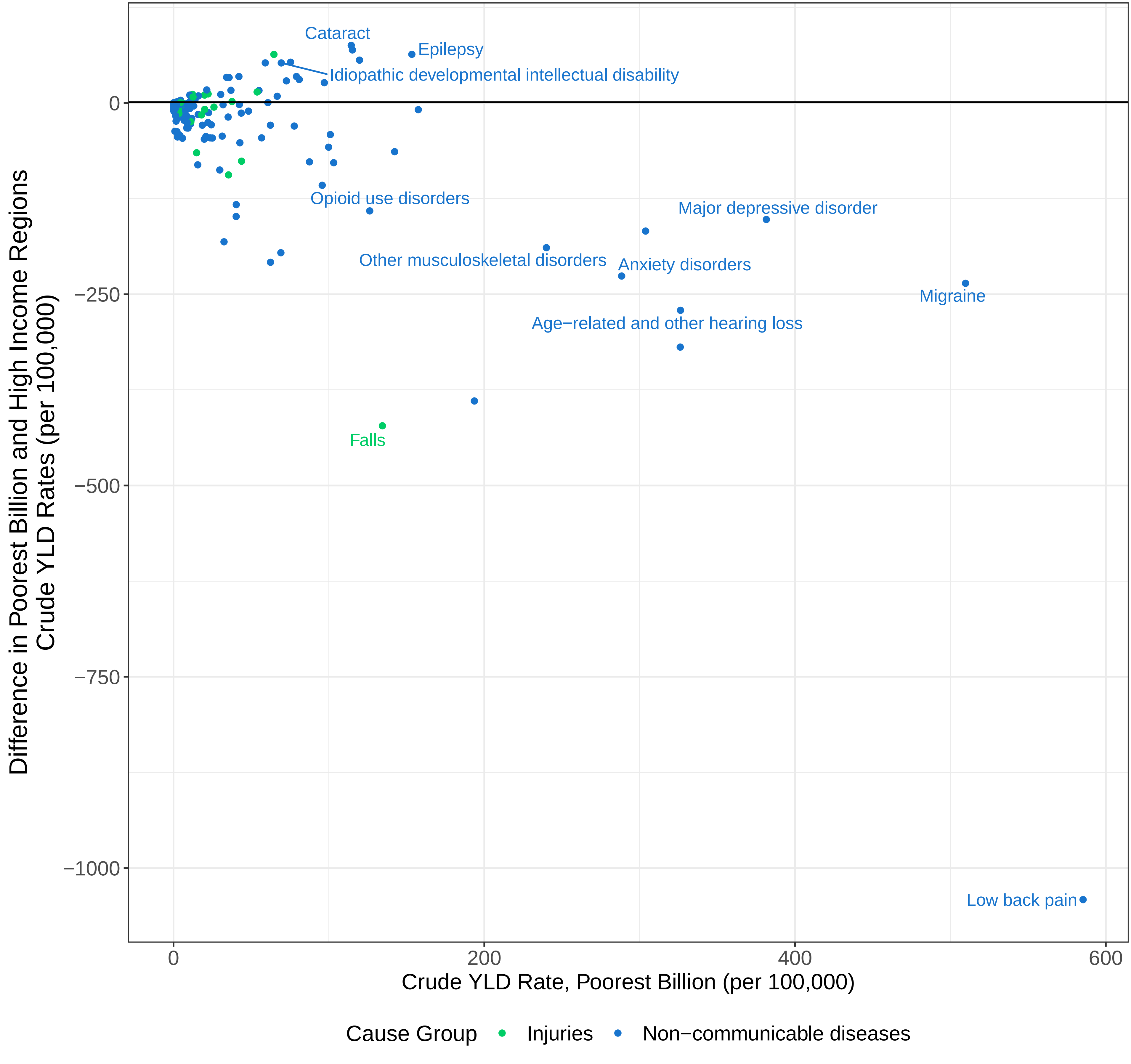

Appendix Figure 12f. Difference between Age−standardized YLD Rates in Poorest Billion and High−income Regions, Poorest Billion Aggregation

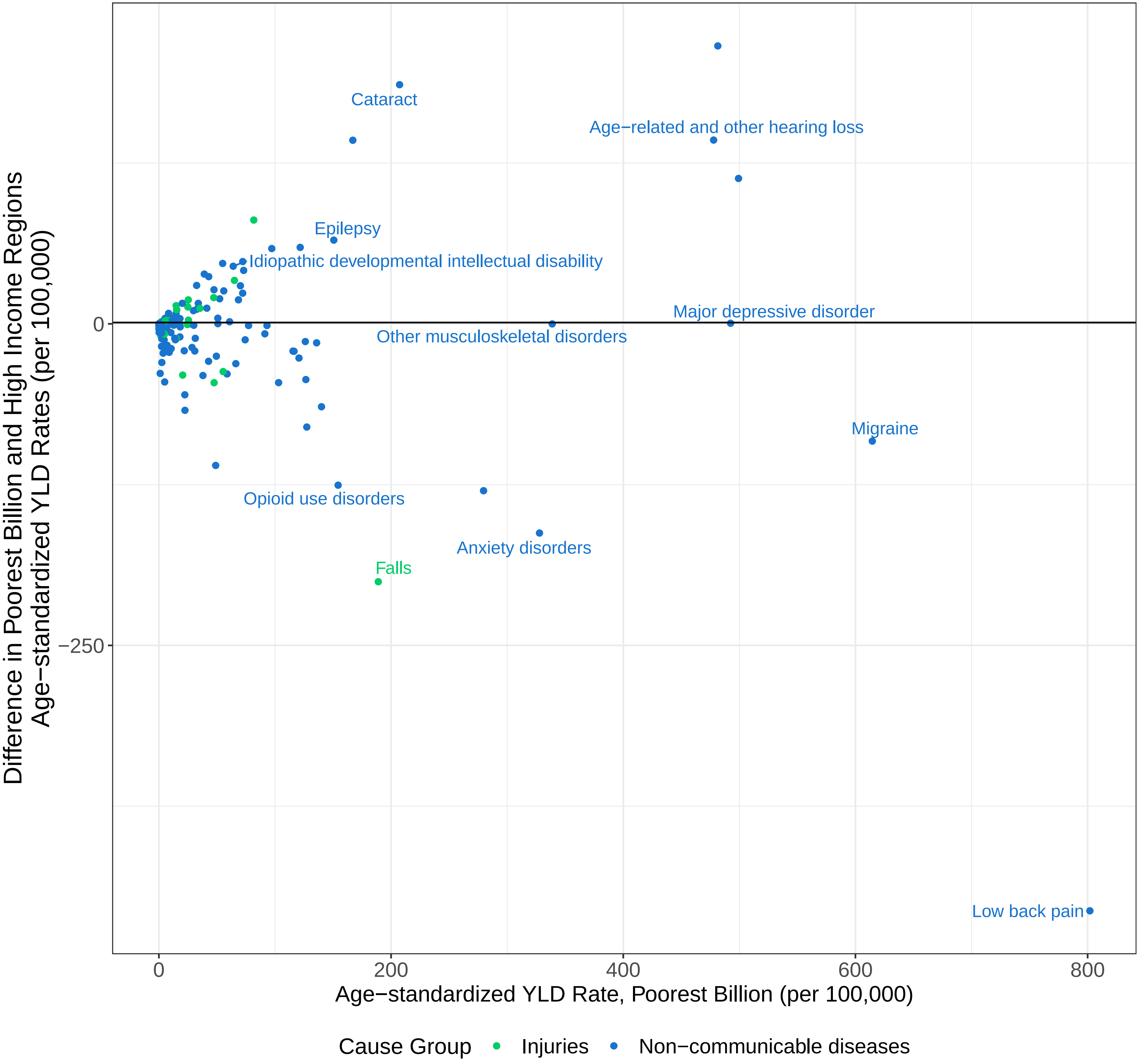

Appendix Figure 12g. Difference between Crude YLD Rates in Poorest Billion and High−income Regions, Country Aggregation

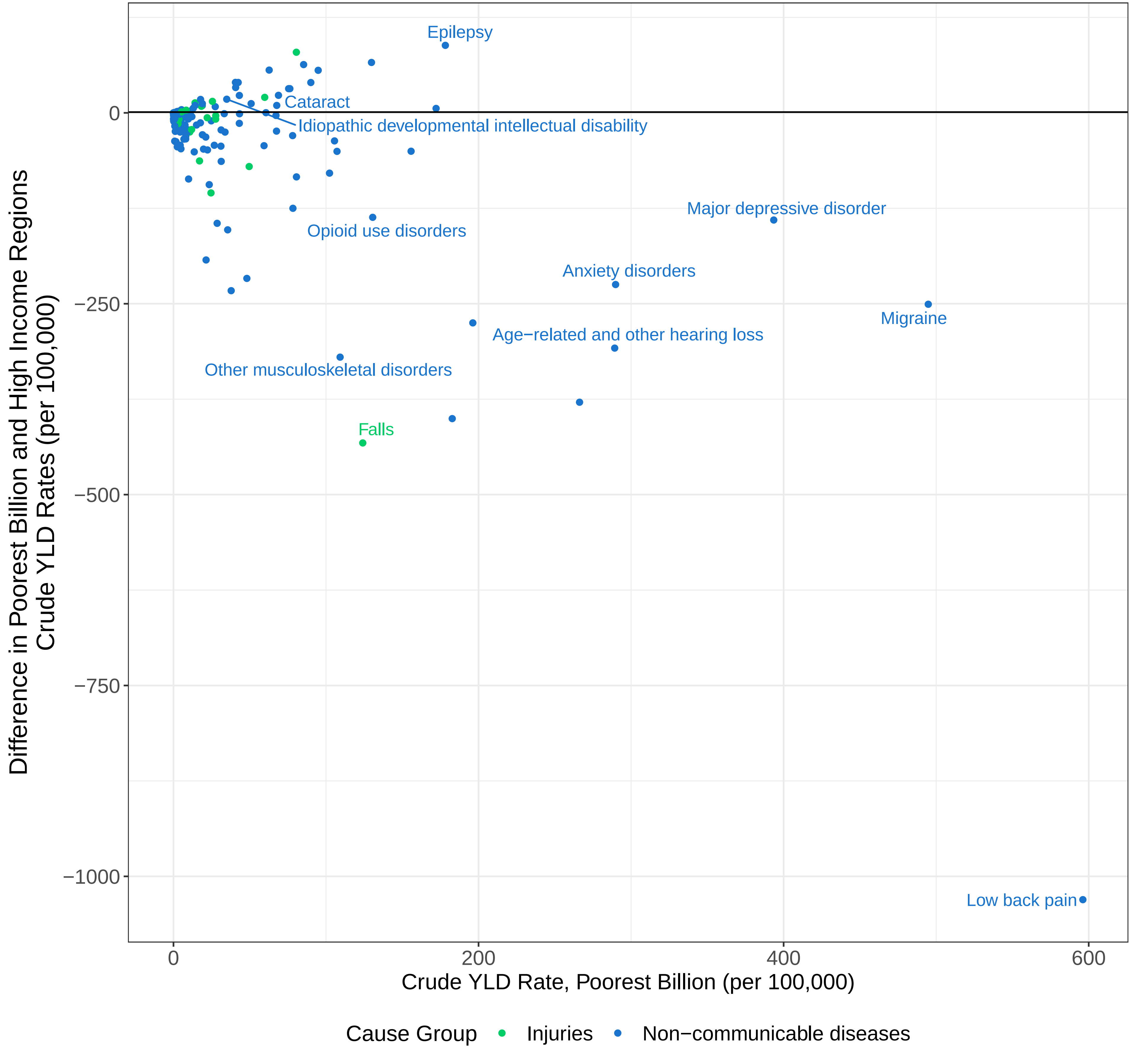

Appendix Figure 12h. Difference between Age−standardized YLD Rates in Poorest Billion and High−income Regions, Country Aggregation

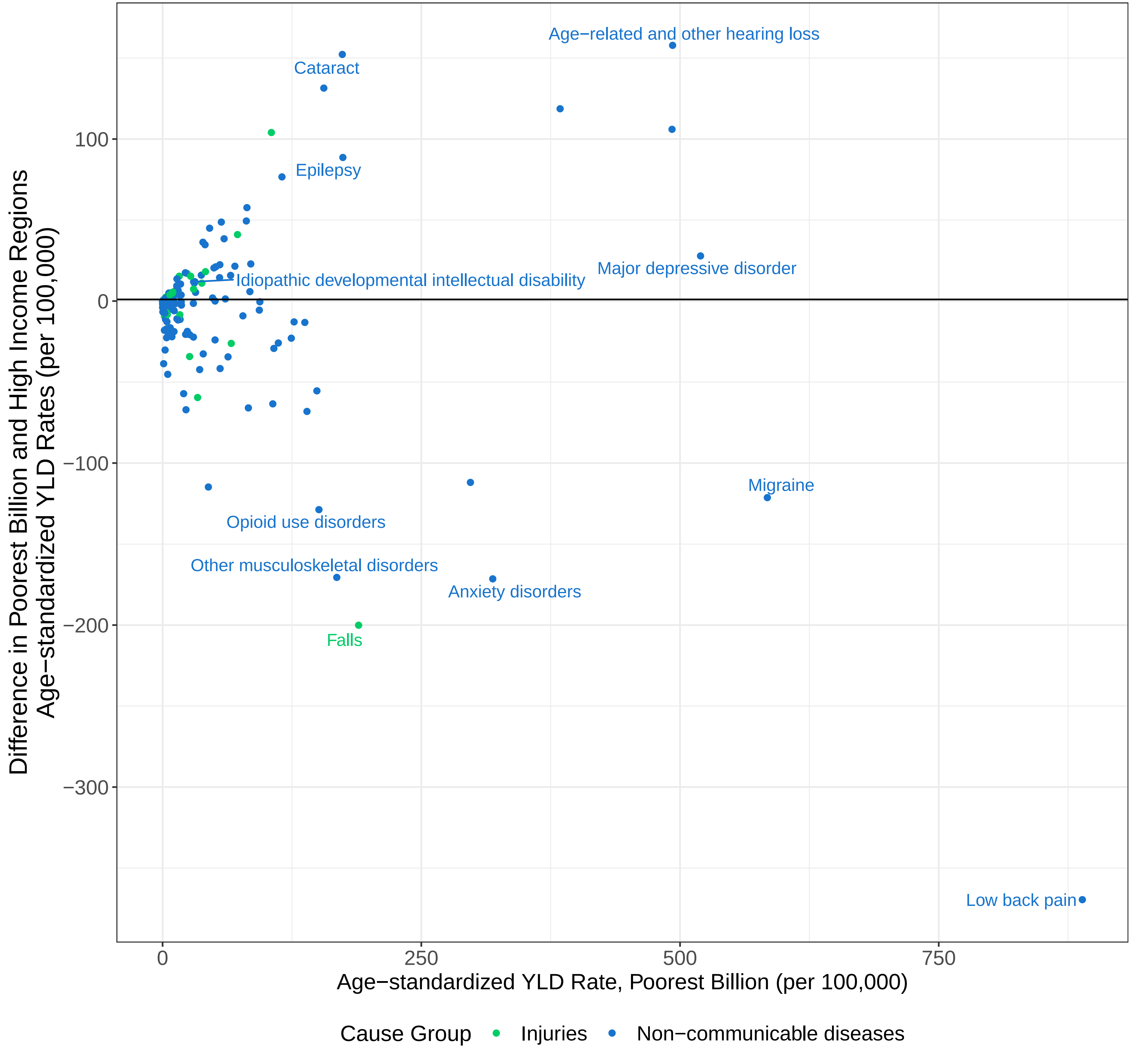

Appendix Figure 12i. Difference between Crude YLD Rates in Low−income and High−income Regions

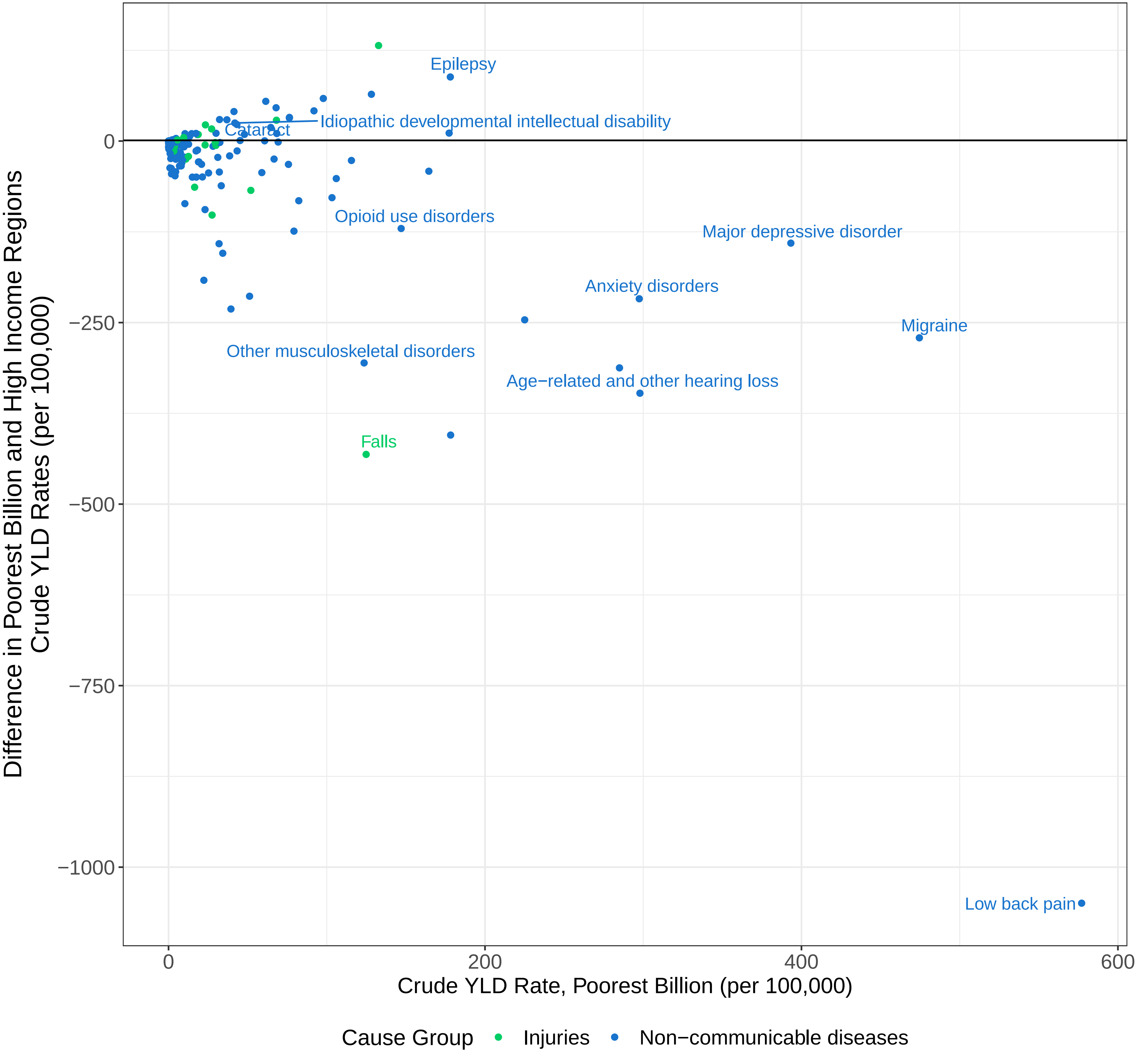

Appendix Figure 12j. Difference between Age−standardized YLD Rates in Low−income and High−income Regions

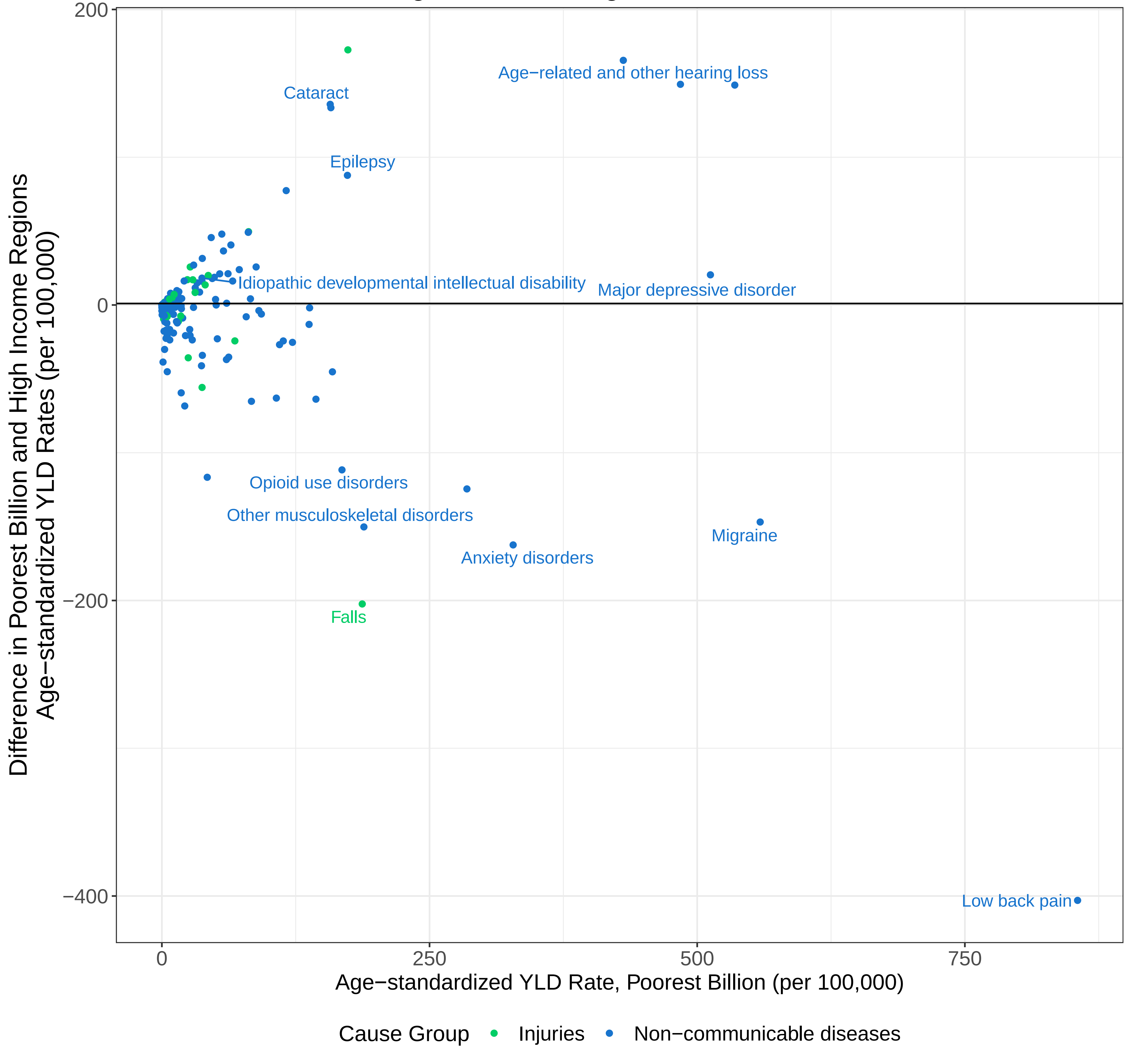

Comparison of Estimation Methods

In general, the characteristics of disease burden across the five methods were similar. Comparatively young populations and high rates of communicable, nutritional, and neonatal disease in these populations shaped the disease burden. DALYs were dominated by YLLs rather than YLDs. Age-standardized rates of all three major categories of diseases, including NCDs, were higher among the group representing the poorest.

Yet, there were some differences between approaches that can be described in terms of geography, population structure, epidemiology of specific conditions, and modeling strategy. Using a definition of the poorest billion based on household socioeconomic status rather than country averages had implications on the country composition of where the poorest live as well as their age distribution. Of the 873 million people experiencing extreme deprivation according to our poverty index, 374 million (43%) lived outside of the countries used in the Country Aggregation approach and 471 million (54%) lived in countries outside of the World Bank low-income countries. Poorer populations within each country tended to be younger, and the populations in lower-income countries also tended to be younger. The geographic distribution of the populations using the different approaches was similar but somewhat varied. The strategies using household-level poverty included a substantial population in India (197 million) that was excluded by the country-level approaches. These geographic variations in approaches had some effect on the rates of specific conditions like HIV/AIDS, neglected tropical diseases, sickle cell disorders, and cervical cancer, which are more prevalent in much of sub-Saharan Africa. Compared to the other approaches, the ecological analyses tended to show somewhat greater disparities between the poorest billion and HIRs because of the within-country effects that they assumed.

Example Analysis

Here, we show an example of how the Ecological Approach worked. In this example, we use diarrheal diseases in females aged 1 to 4 years. The logged death rates were regressed on the percent of the population in the poorest billion at the country level, with a random effect on region.

$$\log(Death\ rate_l) = \beta_0 + \beta_1 PPB_l + \gamma_r + \varepsilon$$

For this condition, the results from the regression are presented below in Appendix Table 7.

Appendix Table 7: Regression example results

	Estimate	Standard Error	t-value
β_0	1.9154	0.3586	5.342
β_1	2.5447	0.4625	5.502

These results are visualized in Appendix Figure 13, where country-level observations are shown as points and the resulting predictions from the model are shown as lines. Note that the y-axis is log-transformed.

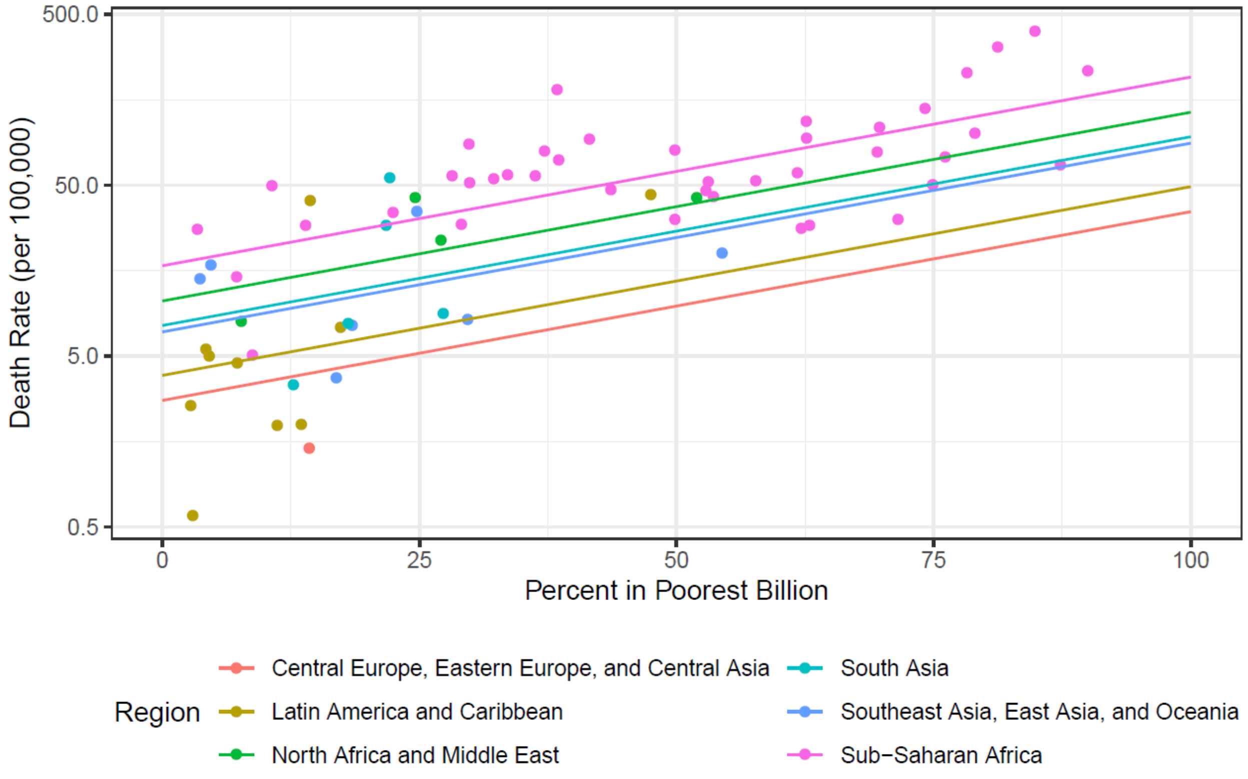

Appendix Figure 13: Analysis example regression results

For the poorest billion living in a given country, estimates of rates were generated by taking the model predictions at 100% living in the poorest billion. For the non-poorest, estimates were generated by taking the model predicitons at 0% living in the poorest billion. For instance, in a country in Sub-Saharan Africa

in this example, the estimated death rate at 0% was 16.88 deaths per 100,000, and at 100%, the estimated death rate was 215.04 per 100,000.

Then, these rates were scaled such that the population-weighted average rate equalled the national-level rate. For instance, in Madagascar, 74.2% of females in the age group in question were thought to be among the poorest billion and the national-level death rate from diarrheal disease in that age and sex group was 139.48 per 100,000. We solved for the scalar (k in the equation below) that made the population-weighted average rate in the poorest billion and non-poorest equal the national-level rate.

$$k\left(Rate_{l,a,s,c}^{pb} * Pop_{l,a,s}^{pb} + Rate_{l,a,s,c}^{npb} * Pop_{l,a,s}^{npb}\right) = Rate_{l,a,s,c}^{GBD} * Pop_{l,a,s}^{GBD}$$

In the case of deaths from diarrheal disease in females aged 1 to 4 in Madagascar, this scalar was about 0.85. So, among females aged 1 to 4 in extreme poverty in Madagascar, the predicted death rate from diarrheal diseases was about 183 per 100,000 (about 215.04*0.85), and among those not living in extreme poverty, the predicted death rate was about 14 per 100,000 (about 16.88*0.85). This procedure was conducted for each country in which we produced results to obtain estimates of rates by poorest billion/non-poorest within each country. The regressions were conducted for each age, sex, and cause group. As described earlier in the appendix (pp 13-15), this procedure was conducted for every health condition in the Full Ecological approach. For the Selective Ecological approach, this procedure was conducted for conditions showing consistency between associations from these regressions and the results from the expert survey (see p 14-15). For other conditions, national-level rates were assumed.
